# Prediction of Absorption Spectrum Shifts in Dyes Adsorbed on Titania

**DOI:** 10.1038/s41598-019-53534-2

**Published:** 2019-11-18

**Authors:** Vishwesh Venkatraman, Amsalu Efrem Yemene, John de Mello

**Affiliations:** Department of Chemistry, Norwegian University of Science and Technology, 7491 Trondheim, Norway

**Keywords:** Cheminformatics, Computational chemistry

## Abstract

Dye adsorption on metal-oxide films often results in small to substantial absorption shifts relative to the solution phase, with undesirable consequences for the performance of dye-sensitized solar cells and optical sensors. While density functional theory is frequently used to model such behaviour, it is too time-consuming for rapid assessment. In this paper, we explore the use of supervised machine learning to predict whether dye adsorption on titania is likely to induce a change in its absorption characteristics. The physicochemical features of each dye were encoded as a numeric vector whose elements are the counts of molecular fragments and topological indices. Various classification models were subsequently trained to predict the type of absorption shift i.e. blue, red or unchanged (|Δ*λ*| ≤ 10 nm). The models were able to predict the nature of the shift with a good likelihood (~80%) of success when applied to unseen data.

## Introduction

The light-harvesting properties of dye-sensitized metal oxides find a number of applications in photonic devices and chemical probes. They constitute a significant area of current research, and are key components of many devices including dye-sensitized solar cells^1^, photo-electrochemical water splitters^2^ and optical filters^3–5^. General requirements of the constituent dyes include broad absorption spectra (preferably extending into the near-infrared portion of the solar spectrum) accompanied by large extinction coefficients^1^. To meet these objectives, numerous organic^6^ and metal-based^7^ dyes have been designed^8^, employing varying donors (D), *π*-bridges, and acceptors (A) including but not limited to the following configurations: D-*π*-A, D-A-*π*-A^9^ and D-D-*π*-A^10^. Each dye is further chemisorbed onto a semiconducting metal oxide photoanode (usually TiO_2_^11^), to provide a mesoporous metal-oxide dye interface at which efficient separation of photo-generated electron-hole pairs can occur. The nature of the donors, acceptor/anchoring groups and the strength of the dye-semiconductor coupling, all have a significant impact on the photostability and photochemical behaviour.

Broad absorption spectra are desirable for light harvesting. However, it is often seen (particularly for metal-free dyes) that the UV-vis absorption peaks of the dyes adsorbed on TiO_2_ photoelectrodes are substantially shifted, compared to those in solution. While for some dyes, there is little or no change (for some a broadening of the peak is seen), peaks in other cases can be shifted by 100 nm or more^12,13^ in either direction, greatly complicating the design and selection of candidate dyes. Reasons attributed to such phenomena include the deprotonation of the carboxylic/cyanoacrylic anchoring group, *π*-stacking interactions, complexation with metal ions and dye aggregation^9,14–19^. On adsorption, deprotonation can result in a carboxylate-TiO_2_ unit that is a weaker electron acceptor than the native carboxylic acid^20,21^. Furthermore, during the sensitization process, significant dye aggregation can occur^22^. While J-aggregates lead to a red-shift, formation of H-aggregates causes a blue-shift, leading to a damping of the absorption efficiency. The aggregation behaviour is however very dye-specific. Complexation with metal ions such as aluminium, iron, tin, titanium and chromium are also seen to induce red-shifts particularly for anthocyanin dyes^23–25^ owing to the suspected formation of a quinoidal structure^14^. In the case of catechol anchoring groups^26^, absorption shifts have been attributed to the increased dipole moment of the Ti-ligand complex via an induced charge transfer dipole under excitation^27^. Solvatochromism also has a significant impact on the relative spectral shifts. For instance, it has been shown that in polar solvents, the electron-withdrawing power of the carboxylic acid decreases as a result of a partial deprotonation in the excited state^28^. The use of different sensitization solvents is also seen to affect the adsorption characteristics of dyes^29,30^.

So far, understanding the origin of these spectral changes and their possible effects, has largely been based on comparative studies of the dye in solution and in its adsorbed state. Selecting a dye purely on the basis of its solution-phase properties has until now been a unreliable task. Theoretical investigations have focused on analysing the aggregation behaviour^31–35^ and impact of the anchoring groups^29^, using density functional theory (DFT) and *ab initio* methods^36^. Calculations of excited-state properties using time-dependent DFT (TD-DFT) methods^35,37,38^ are generally seen to agree well with the experimental measurements^39,40^. However, such tasks require considerable time and are therefore, not suitable for rapid screening tasks involving a large number of molecules. An additional challenge is to identify the dye-oxide binding mode which will change depending on the structure of the dye and the binding groups. For example, the —COOH group can form monodentate ester-like, bidentate chelate or bidentate bridging linkages^41,42^. In the absence of any prior knowledge, multiple combinations must be tested, thereby adding to the computational effort.

Machine learning (ML) approaches capable of identifying embedded correlations between structure (represented appropriately) and property have been successfully used in materials science and computational chemistry^43–47^. We therefore ask the question: based only on the knowledge of a dye molecule's chemical structure and its absorption spectrum in a given solvent, can we use data-driven ML techniques to predict the type of absorption shift? To this purpose, the UV-Vis absorption peaks in solution and on a metal oxide were extracted from literature for ~2000 dyes. The change in the maximum absorption wavelength from solution-phase to metal-oxide-supported was used to categorise the dyes as blue-shifted, red-shifted or unchanged. Supervised machine learning models were then trained to distinguish between the classes using descriptors such as molecular fragment counts and topological indices that are easily calculated from the dye structure. Our results show that using cheaply derived structure descriptors, the classification models can achieve 80% success in predicting the type of absorption shift.

## Methods

### Data curation

**Figure 1:**
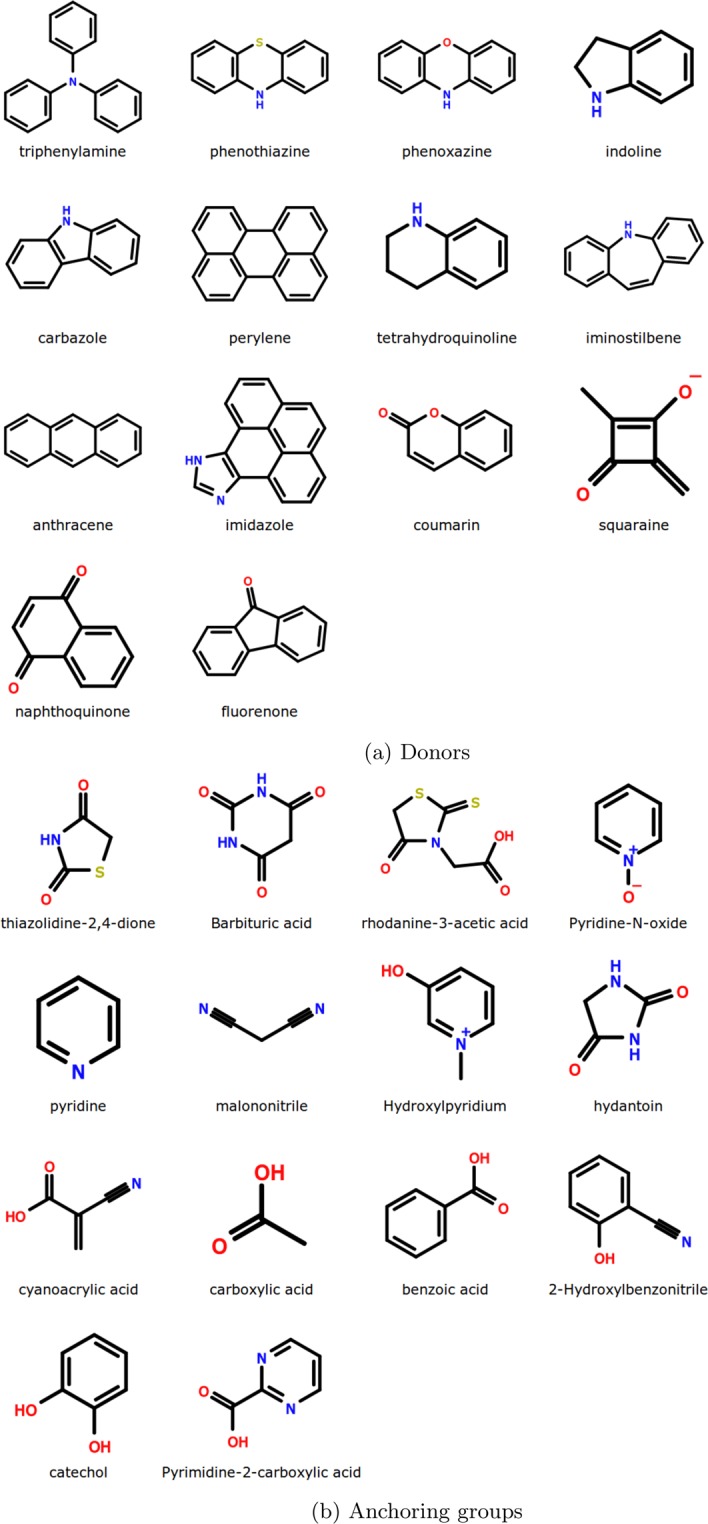
Prominent donors and anchoring groups present in the dyes included in the data set.

Absorption data in solution and on TiO_2_ for ~2000 metal-free dyes were extracted from around 500 literature articles. Other metal oxides such as ZnO, NiO and SnO_2_ were not considered as the available data was too limited. For some dyes, the reported absorption peaks (from different studies) in the same solvent were found to be significantly different and were therefore omitted. We further considered only those cases for which values were recorded in pure solvents and without any additives such as chenodeoxycholic acid. In the end, a total of 1961 observations corresponding to 1861 unique dyes were obtained. For these compounds, the difference in the absorption maxima (*λ*) i.e. $\Delta \lambda ={\lambda }_{{\rm{\max }}}^{{\rm{soln}}}-{\lambda }_{{\rm{\max }}}^{{{\rm{TiO}}}_{2}}$ ranged between −220 to +190 nm (see Fig. F1 in the Supplementary Material-II). The structures spanned various donor classes such as triphenylamines, phenothiazines, carbazoles, coumarins etc. with varying numbers and types of anchoring groups — catechol, hydroxylpyridium^48^, cyanoacrylic, pyrimidine (see Fig. 1). In the assembled data, the dyes were divided into ten separate groups based on the solvent (see Table 1). The molecular structures (SMILES format), absorption properties and associated references are provided in Table S1 in the Supplementary Material-I.

**Table 1:** Number of data points (*N*_*Obs*_) grouped according to the solvent in which the spectra were measured. The third column shows range of the shift (in nm) calculated as ${\lambda }_{{\rm{\max }}}^{{\rm{soln}}}-{\lambda }_{{\rm{\max }}}^{{{\rm{TiO}}}_{2}}$.

Solvent	*N*_*Obs*_	Range (nm)	*P*′	Distribution		
				B:NR	B:N:R	NB:R
Chloroform (CHCl3)	258	−94–134	4.1	141:117	141:67:50	208:50
Dichloromethane (DCM)	753	−219–190	3.1	429:324	429:188:136	617:136
Tetrahydrofuran (THF)	493	−116–140	4	205:288	205:117:171	322:171
Ethanol (EtOH)	174	−207–102	5.2	47:127	47:51:76	98:76
Methanol (MeOH)	39	−21–122	5.1	15:24	15:12:12	27:12
Acetonitrile (MeCN)	55	−109–108	5.8	22:33	22:7:26	29:26
Toluene	36	−22–64	2.4	16:20	16:11:9	27:9
1,4-dioxane	19	−71–33	4.8	3:16	3:3:13	6:13
Dimethylformamide (DMF)	114	−92–115	6.4	20:94	20:35:59	55:59
Dimethylsulfoxide (DMSO)	20	−38–62	7.2	11:9	11:3:6	14:6
Overall	1915	−219–190	—	909:1052	909:494:558	1403:558

The relative polarities (*P*′) for the solvents are taken from Snyder^104^. The last column shows the distribution of the categories formed by combining dyes that show no/little change (*N*), red-shift (*R*) or blue-shift (*B*) after adsorption on TiO_2_.

### Nature of the shift

**Figure 2:**
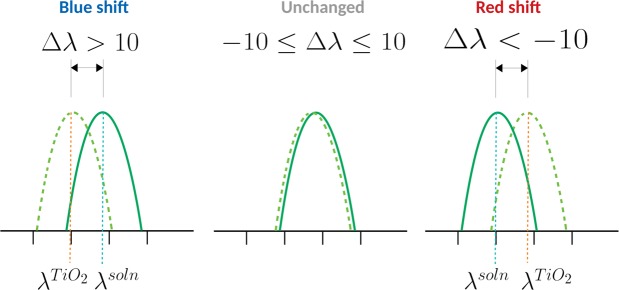
Nature of the spectral shift based on the positions of the absorption peaks corresponding to the solution phase (${\lambda }_{{\rm{\max }}}^{{\rm{soln}}}$) and solid-state maxima (${\lambda }_{{\rm{\max }}}^{{{\rm{TiO}}}_{2}}$). The difference between the two values: $\Delta \lambda ={\lambda }_{{\rm{\max }}}^{{\rm{soln}}}-{\lambda }_{{\rm{\max }}}^{{{\rm{TiO}}}_{2}}$ is used to determine whether there is a blue shift, red shift or no change upon adsorption.

**Figure 3:**
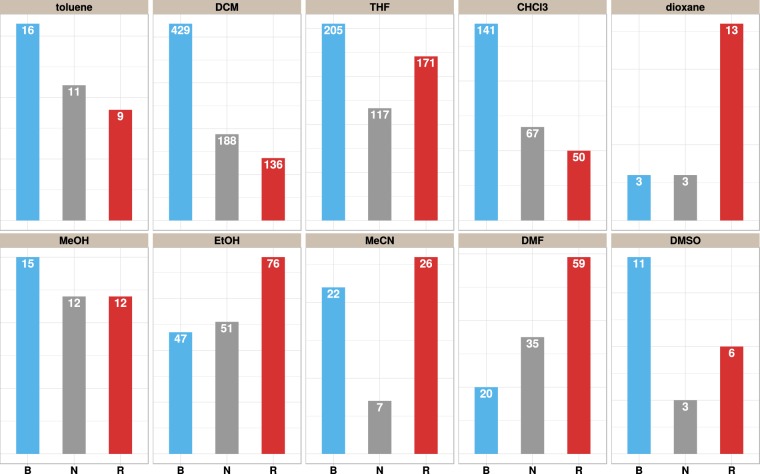
Distribution of the absorption shifts with respect to the solvents. The letter “B” indicates a blue shift, while “N” and “R” correspond to no change (|Δ*λ*| ≤ 10) or a red shift respectively. The solvents are sorted in increasing order of polarity from left to right. Polarity values are listed in Table 1. Additional plots are provided in the Supplementary Material-II.

The nature of the spectral shift was determined (see Fig. 2) by thresholding the difference between the solution phase and solid-state maxima ($\Delta \lambda ={\lambda }_{{\rm{\max }}}^{{\rm{soln}}}-{\lambda }_{{\rm{\max }}}^{{{\rm{TiO}}}_{2}}$) with respect to the following criteria:

$${S}_{B:NR}=\{\begin{array}{ll}{\rm{B}}, & {\rm{if}}\,\Delta \lambda  > 10\,{\rm{nm}}\\ {\rm{NR}}, & {\rm{otherwise}}\end{array}$$

$${S}_{B:N:R}=\{\begin{array}{ll}{\rm{N}}, & {\rm{if}}-\,10\,{\rm{nm}}\le \Delta \lambda \le 10\,{\rm{nm}}\\ {\rm{R}}, & {\rm{if}}\,\Delta \lambda  < -\,10\,{\rm{nm}}\\ {\rm{B}}, & {\rm{otherwise}}\end{array}$$

$${S}_{BN:R}=\{\begin{array}{ll}{\rm{BN}}, & {\rm{if}}\,\Delta \lambda  < -\,10\,{\rm{nm}}\\ {\rm{R}}, & {\rm{otherwise}}\end{array}$$

where *B*, *R*, and *N* indicate a blue shift, red shift and little or no change respectively. Instead of using a strict cut-off of 0, deviations of 10 nm or less were designated *N*. Figure 3 provides a solvent-wise distribution of the experimentally derived categories. For a majority of the cases associated with weakly polar solvents such as DCM, THF and CHCl3, a blue-shifted absorption is seen, while red-shifted behaviour becomes more prominent as polarity increases. This may be attributed to a more efficient solvation of the dyes in the polar solvents^49^.

The categories can be further grouped into *NR* (no change + red shift) or *BN* (blue-shift + no change). In the context of machine learning, a balanced distribution of the instances across the classes (50% to class A and 50% to class B) is preferred. Owing to the presence of higher number of instances for a given category, classification learners run the risk of predicting everything as one or the other class^50^. To this end, we analysed three different schemes (I) the first where three different groups *B*, *N* and *R* are established, (II) the second merges the red-shifted dyes with those indicating little or no change — *B*/*NR* and lastly, (III) merging the blue-shifted and no change — *BN*/*R*. When considering three independent groups — *B*,*N*,*R*, the class distributions (2:1:1) are slightly skewed towards the blue. For the second case (*B*:*NR*), a near 1:1 ratio is seen for most solvents is seen, with the exception of DMF for which a majority of the cases belong to the *NR* category. The converse holds true for the third case (*NB*:*R*), where the distribution of the categories is found to be significantly skewed (2.5:1) in favour of *NB* for a majority of the solvents. Consequently, in this paper, we focus mainly on the *B*:*NR* cases where the data is well balanced and more likely to yield a more effective classification.

### Molecular descriptors

For a statistical structure-property relationship to be established, the molecules need to be represented in a way that captures their physicochemical characteristics. In cheminformatics, these representations are typically referred to as molecular descriptors^51^ i.e. a vector of numbers that captures the chemical information in a computer-interpretable form. Machine learning approaches use these vectors to infer a predictive model from the training data. Here, we have employed the following schemes to encode each dye:

#### Atom-bond sequences

The first set of descriptors are extracted by enumerating all the shortest paths (successive connected atom-bond sequences) between each pair of atoms^52^. The descriptor calculation was carried out using the ISIDA Fragmentor2017 software^53^. The minimum and maximum length of the atom-bond sequences were set to 3 and 6 respectively.

#### Topological indices

The second set of descriptors includes constitutional indices (number of hetero atoms and aromatic rings, hydrogen bond acceptors and donors) as well as topological indices (derived from chemical graph representations) that take into account the connectivity along with atom and bond labels. Popular descriptors include the electrotopological state^54^ (EState) indices that encode the topology and electronic environment of molecular fragments. Other variables include MOE-type descriptors^55^ that are based on an approximate accessible van der Waals surface area calculation for each atom, along with some other atomic property. Here, we have included properties such as *logP* (octanol/water), molar refractivity, and partial charge within a binned range (corresponding to a subdivision of the molecular surface area). The descriptors were computed using the open source cheminformatics toolkit RDKit^56^. For a preset bin size (*k*), the calculated descriptors include SlogP–VSA_*k*_ (capture hydrophobic and hydrophilic effects), SMR–VSA_*k*_ (polarizability) and PEOE–VSA_*k*_ (capture electrostatic interactions). The descriptors were computed using the open source cheminformatics toolkit RDKit^56^.

The descriptors were selected to capture relevant features of the dye's chemical structure and without resorting to DFT or other computationally intensive calculations. Each structure was therefore described by a vector of length 2060 (solvent polarity was added as an additional descriptor) with computations taking less than 3 minutes to calculate all descriptors for the entire data set.

### Machine learning

In order to identify machine learning models capable of discriminating between the different types of shifts (*B*/*N*/*R*), six popular classification schemes were explored: linear discriminant analysis^57^ (LDA), *k*-nearest neighbours (*k*-NN), kernel-based support vector machines^58^ (SVM), and tree-based models such as classification and regression trees^59^ (CART), random forests^60^ (RF) and gradient boosting machines^61^ (GBM). Linear discriminant analysis works by identifying a linear combination of the variables (projection onto a smaller subspace) that best separates the classes. The *k*-NN algorithm classifies an object based on a majority vote of its *k* nearest neighbours that are identified by calculating the Euclidean distance from the point of interest (the class of which is to be determined) to all the points in training set. Support vector machines perform classification by finding the hyperplane that maximizes the margin between the classes^58^. For a two-dimensional space, the hyperplane is a line that divides a plane into two parts such that each class lies on either side. The vectors that define this hyperplane are the support vectors. The tree-based models output a series of if-then-else statements where the features are systematically checked to determine a final result. While the CART approach produces a single tree, both random forests and GBM are ensemble approaches where the outcome is the combination of the the decisions from multiple models. The difference lies in the way the trees are built: RF builds deep independent trees, while GBM creates successive models with each tree improving on the previous i.e. they seek to improve the result based on the current estimate.

### Statistical modelling

Analysis of the data started with the removal of descriptor columns with little or no variation and those containing missing values (due to an inability to calculate one or more descriptors). The data was then split randomly into independent calibration (75%) and test (25%) sets. The presence of highly correlated variables (multicollinearity) can affect predictive performance. Following previous studies^62,63^, a pair-wise squared correlation cut-off of 0.90 was applied to the training set, whereby only one (arbitrarily determined) among the correlated pair of variables was retained. This reduced the number of variables from 2000 to around 200. In order to select the best model parameters (e.g. number of trees for RF, depth of the tree, number of neighbours to be considered (*k*-NN)), a five-fold cross-validation was employed, followed by randomization tests to reduce the risk of overfitting. A grid search was carried out to identify the optimal parameter combinations for the ML models used. The modelling was carried out using *R*^64^. Owing to the class imbalance in the data, models trained using performance metrics such as the accuracy are biased towards the more frequent class (sensitive to class skews), and may suffer from a lack of generalizability. We have therefore assessed classification performance using the balanced accuracy^65,66^ which is defined as the average accuracy obtained on all classes:

1 $$BACC=\frac{1}{m}\mathop{\sum }\limits_{i}^{m}\frac{{k}_{i}}{{n}_{i}}$$

where *k*_*i*_ is the number of correct predictions in class *i*, *m* is the number of classes and *n*_*i*_ is the number of examples in class *i*. Other metrics such as the average accuracy (the average per-class effectiveness of a classifier), sensitivity (the true positive rate - TPR) and specificity (the true negative rate - TNR) are also reported for comparison^67^.

2 $$ACC=\frac{\mathop{\sum }\limits_{i}^{m}\,\frac{t{p}_{i}+t{n}_{i}}{t{p}_{i}+t{n}_{i}+f{p}_{i}+f{n}_{i}}}{m}$$

3 $$TPR=\frac{\mathop{\sum }\limits_{i}^{m}\frac{t{p}_{i}}{t{p}_{i}+f{n}_{i}}}{m}$$

4 $$TNR=\frac{\mathop{\sum }\limits_{i}^{m}\frac{t{n}_{i}}{t{n}_{i}+f{p}_{i}}}{m}$$

where, for an individual class *C*_*i*_ − *tp*_*i*_, *fp*_*i*_, *tn*_*i*_ and *fn*_*i*_ are the true positive, false positive, true negative and false negative counts respectively.

## Results and Discussion

### Manual analysis of the data

**Figure 4:**
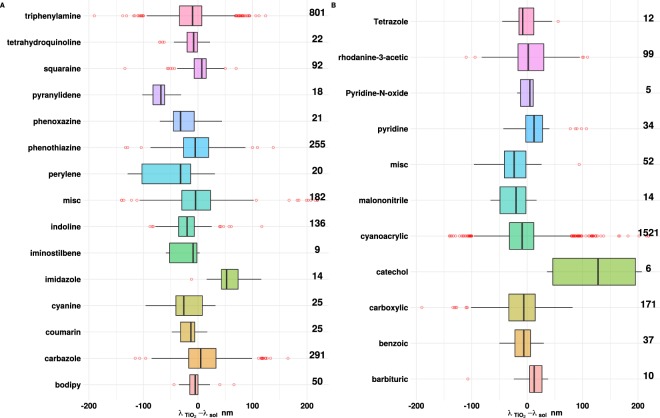
Box plots showing the distribution of the absorption shifts (irrespective of the solvent) based on the (A) class of the dyes and (B) the anchoring groups used. (**A**) The “misc” category includes various dyes based on pyrazoline, naphthoquinone, N,N-dialkylaniline^105^, julolidine^106^, bithiazole^107^, cyclohexadiene^108^ etc. (**B**) The “misc” category includes dyes with anchoring groups that include thiazolidine^73^, aldehyde, hydantoin^109^, isophorone^71^, phosphonic acid and pyrimidine^110^. Numbers on the right are the counts of cases found in each category.

In order to ascertain if there were any noticeable patterns associated with the absorption shifts, the experimental data was analysed with respect to the class of the dye, the conjugated spacers used, and the number and types of anchoring groups. Figure 4A,B summarize the data in terms of the dye class and type of anchoring groups, respectively. For simplicity, we have ignored the solvent medium allowing for a broader analysis. Examination of Fig. 4A, shows that dyes based on imidazole^12,68^ exclusively show red shifts, while those based on pyranylidene^69^ exclusively show blue shifts. All other dye classes exhibit both blue and red shifts.

Analysis of the anchoring groups (Fig. 4B) suggests that, those containing catechol, pyridine, and barbituric acid are largely red-shifted. The particularly large red shifts in the catechol group are attributable to their ability to strongly adsorb on to the TiO_2_ surface^70^ as well as the increased dipole moment of the surface-bound metal-ligand complex^26,27^. For some of the other groups such as isophorone^71^, malononitrile^72^, thiazolidine^73^ and benzoic acid^74^, a majority of the cases are blue-shifted. The impact of multiple anchoring groups^75^ was also studied (see Fig. F2 in the Supplementary Material-II). The number of dyes containing multiple anchoring groups was low (~170). Nonetheless, for the cases studied, there was no significant correlation between the number of anchoring groups and the size or direction of the spectral shift. While for some cases, little or no change was observed, others showed moderate to large shifts in either direction^76–80^. Wu *et al*.^77^ observed the maximum absorption peak of three triphenylamine dyes in acetonitrile/tert-butanol (1:1, V/V) showed a red-shift with an increasing anchoring group number. Compared to the spectra in solution, the peaks did not show any change on the TiO_2_ film, which was attributed to the cancelling effect of J-type aggregation and deprotonation. For squarylium dyes in particular, Connell *et al*.^81^, have shown that the position of dye anchoring points can influence hydrophobicity and contact angle of dyes adsorbed to TiO_2_ surfaces, which in turn can affect the absorption properties. In order examine the impact of mixed solvents, a total of 146 dyes in 12 different solvent mixtures were analysed. The absorption behaviour for dyes in mixed solvents is shown in Fig. F3 in the Supplementary Material-II. With the exception of solvent mixtures — methanol(MeOH)/chloroform(CHCl3), tert-butanol/acetonitrile(MeCN), ethanol(EtOH)-dichloromethane(DCM) and tetrahydrofuran(THF)-DCM, others had fewer than 10 instances. The dyes using tert-butanol/MeCN and MeOH/CHCl3 as solvents exhibited a greater tendency to blue-shift. For dyes in EtOH-DCM all three categories were equally represented while, for those in THF-DCM, a higher tendency to blue-shift was observed.

Several *π*-conjugated systems such as furan, thiophene and fused aromatic rings have been incorporated into the D-*π*-A architecture as *π*-linkers^82^. These units not only affect the light absorption regions of the DSSCs, but also influence the electron injection into the TiO_2_ surface. For the dyes investigated in this study, a majority of the structures contained thiophene^83^ and its derivatives (such as thienothiophene^84^, indacenodithiophene^85^, dithienopyrrole^86^) as the *π*-bridge. Figure F4 in Supplementary Material-II provides a box-plot of the absorption shifts for the various *π*-linkers (over 40 categories identified) used in the dyes. The conjugated spacers based on vinylene, ethynylene, furan, thiazole, thiophene and other fused aromatic segments (indole^87^, fluorene^88^, benzothiadiazole^89^) showed similar peak shifts in both directions. Other groups such as diphenylquinoxaline^90^, 1-chlorobuta-1,3-diene^91^, dithienobenzotriazole^92^ and dithienobenzofurazan^93^ found in a limited number of cases were largely associated with red-shifted peaks. On the other hand, those containing linkers based on fused thiophene derivatives such as dithienopyrrolobenzotriazole^94^, cyclopentadithiophene^95^, thienothienopyrrole^96^, silolodithiophene^97^ were blue shifted by more than 50 nm compare to the solution.

In conclusion, while for some choices of the dye class, anchoring groups and *π*-spacers we can identify clear patterns, in most cases there is no obvious pattern that can be discerned to predict the nature of the shift. In order to consider more formally, the effect of the structure on the adsorption behaviour, we employ machine learning the problem of predicting the type of the spectral shift and infer which features influence a set of observations.

### Classification performance

Table 2 summarizes the performance of the ML models across the calibration and test sets. In most cases, values for the two sets closely match one other, suggesting that the models generalize well. A comparative evaluation shows that both RF and SVM outperform other models on all classification tasks. The best performance is seen for the case *B*:*NR*, where the RF model achieves a cross-validated *BACC* = 0.76 during training and a slightly higher value of 0.80 on the test set containing 484 data points. On the same data set however, the LDA model performs only marginally better than random and achieves only a 50% accuracy on the other sets. Other models (*k*-NN, RF, SVM, GBM) are relatively more successful in separating classes by non-linear boundaries. Although the other binary classification problem *NB*:*R* has a moderate class imbalance ($\sim \mathrm{2.5:1}$), RF classification accuracies are only slightly lower with *BACC* of 0.72 on the calibration and 0.73 on the test set.

**Table 2:** Balanced accuracies obtained by the ML models for the calibration and test data.

Method	B:N:R		NB:R		B:NR	
	TRAIN	TEST	TRAIN	TEST	TRAIN	TEST
GBM	0.68	0.71	0.73	0.72	0.73	0.72
RF	**0.71**	**0.76**	**0.76**	**0.80**	**0.76**	**0.80**
CART	0.61	0.61	0.65	0.65	0.65	0.65
*k*-NN	0.65	0.70	0.71	0.66	0.71	0.66
LDA	0.52	0.53	0.60	0.59	0.60	0.59
SVM	**0.71**	**0.73**	**0.77**	**0.79**	**0.77**	**0.79**

**Figure 5:**
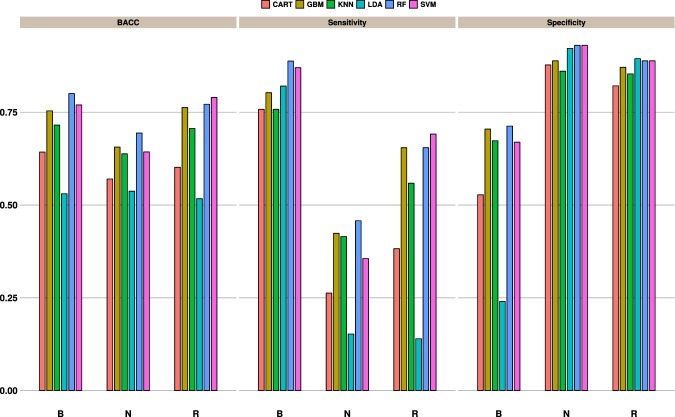
Bar plots showing the multiclass prediction performance on the test data. For each model, the per-class balanced accuracy, sensitivity and specificity are compared.

In the case of the multiclass *B*:*N*:*R* problem, a *BACC* = 0.71 for the calibration data is obtained. Corresponding values for the test set are somewhat higher at 0.76. To better understand the classification performance, the ML predictions for the test set was examined on a class-wise basis. Values of the per class balanced accuracy (*BACC*), sensitivity (*TPR*) and specificity (*TNR*) are shown as bar plots in Fig. 5. For the blue-shifted (*B*) cases, all models show a high sensitivity, albeit with a fairly high rate of false alarms (decreased specificity). Given that there are twice as many cases of blue-shifted dyes, the classifier favours the majority class. A common practice to address the class imbalance problem is to balance them artificially where, for instance, cases from the minority class are replicated or alternatively by ignoring cases from the majority class. However, for the data sets in this study, no visible improvement in performance was observed when such schemes were used.

### Descriptor analysis

**Figure 6:**
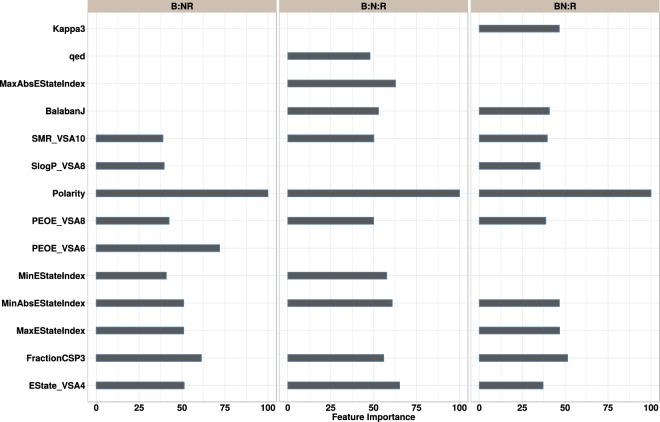
Variable importance plots for the RF model computed for each task: *B*/*NR*, *B*/*N*/*R* and *NB*/*R*. Only the top 10 most important variables are shown for each task. The bars show the contribution of the matching feature to the prediction. A missing bar for a given variable indicates that the said variable was ranked lower.

Compared to the CART scheme which uses a single tree, the tree-based ensemble models such as RF and GB are somewhat “black box” in nature given that they use multiple trees to arrive at a given outcome. We have therefore attempted to interpret the models by way of variable importance plots (shown in Fig. 6) that provide a qualitative understanding of the contribution that each input variable makes to the model^98,99^. We focus on the RF models that show the best performance. To this end, we examined the 10 most significant variables in the RF model trained for each task. The variable contributions are scaled to have a maximum value of 100 and those with higher values are expected to have a high predictive power. Given that the data contains various solvents, the relative polarity (*P*′) is a major contributor. To assess the impact of a single descriptor, we calculated the accuracy of the classifier obtained by setting a threshold (typically the mean) on the value of the variable^98^. For the test set, the constitutional descriptor “FractionCSP3” (fraction of sp3-hybridized carbon atoms) yielded a single variable classifier with an accuracy of 54% and 58% for the entire data. The BalabanJ descriptor is indicative of a large degree of branching of the molecule. This agrees with the experimental data which shows that the large size of branched dyes can lead to a poor dye loading on the TiO_2_ surface^28,100^. The E-state descriptors (MinEStateIndex, MaxEStateIndex, MinAbsEStateIndex, MaxAbsEStateIndex) for each atom in a given molecule reflect the steric and electronic effects of the surrounding atoms.

### External validation

**Figure 7:**
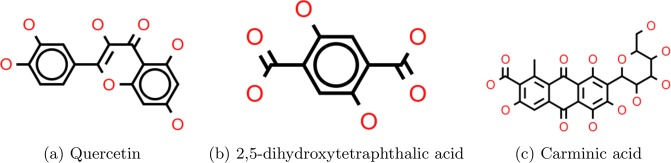
Structures of 3 dyes quercetin, 2,5-dihydroxytetraphthalic acid and carminic acid (purchased from Sigma-Aldrich).

In order to test the performance of the ML models on unseen data, we examined the absorption behaviour for 3 dyes quercetin, 2,5-dihydroxytetraphthalic acid and carminic acid (purchased from Sigma-Aldrich) in ethanol and THF (see Fig. 7). Experimental details are provided in the Supplementary Material. While two of the dyes (T01, T02) show negligible change on adsorption, carminic acid (T03) shows a very small blue shift. However, based on the selected criteria (|Δ*λ*| ≤ 10) they are categorized as *NR*. The RF predictions for the dyes are listed in Table 3, which shows that all instances are correctly classified. We also investigated electronic absorption spectra of the isolated dye as well as those adsorbed on titania using a (TiO_2_)_9_ cluster^101^. The Gaussian 09^102^ calculations were carried out using the B3LYP functional and the 6-31G(d,p) basis set for the C, H, O and N atoms and the effective core potential LANL2DZ basis set for the Ti atoms. Solvent effects were considered using the using the conductor-like polarizable continuum model^103^ (CPCM) along with the CAM-B3LYP functional. Computation times varied between 6–10 hours per structure. Although TD-DFT was not able to accurately predict the absorption peaks, its performance with respect to identifying the nature of the shift is comparable with that of the ML approach, albeit at a much higher computational cost.

**Table 3:** Comparison of the experimental and machine learning (RF) predictions for dyes (T01: quercetin, T02: 2,5-dihydroxytetraphthalic acid and T03: carminic acid shown in Fig. 7) in different solvents.

Dye	Solvent	${{\boldsymbol{\lambda }}}_{{\bf{s}}{\bf{o}}{\bf{l}}{\bf{n}}}^{{\bf{m}}{\bf{a}}{\bf{x}}}$	${{\boldsymbol{\lambda }}}_{{\bf{T}}{\bf{i}}{{\bf{O}}}_{{\bf{2}}}}^{{\bf{m}}{\bf{a}}{\bf{x}}}$	Shift	ML	DFT
T01	Ethanol	373	376	NR	NR	NR
	THF	370	376	NR	NR	NR
T02	Ethanol	372	370	NR	NR	NR
	THF	376	370	NR	NR	NR
T03	Ethanol	495	487	NR	NR	NR
	THF	497	487	NR	NR	NR

The UV-VIS absorption spectra for the dyes in ethanol and THF and on TiO2 are shown in Figs F5–F7 in the Supplementary Material-II.

The predictive performance of the RF model was also tested on an additional unseen data set. A second round of literature search was undertaken that yielded an additional set of 60 data points corresponding to 34 diverse dyes that included triphenylamine, indoline, bodipy, julolidine, and pyrenoimidazole based donors. Solvents in this list included dichloromethane (14 cases), THF (19), acetonitrile (6), toluene (9), DMF (6) and methanol (6). Table 4 summarizes the performance of the RF model on the second test set. The evaluation metrics are similar to those seen for the initial test set and reinforce the initial assessment of generalizability of the models.

**Table 4:** Classification performance for the RF model on a second independent test set.

Category	BACC	ACC	Sensitivity	Specificity
B:NR	0.81	0.83	0.77	0.85
NB:R	0.81	0.75	0.94	0.67
B:N:R	0.71	0.73	0.56	0.86

Overall, the ML models, trained using only “two-dimensional” information consisting of atom types and connectivity, are capable of identifying dyes that have a propensity to blue- or red-shift on adsorption. A similar theoretical assessment using TD-DFT approaches requires the calculations to be carried out on both the isolated dye molecule isolated and adsorbed on TiO_2_ clusters^32^. While the descriptor calculations are completed in less than a second, evaluations using DFT/TD-DFT approaches took more than 6–8 hours per structure. On the other hand, despite not being provided with the details about contributing factors such as the adsorption mode and strength of dye-cluster coupling, the ML models are able to deduce the nature of the absorption shift with reasonable accuracy (~70–80%).

In this work, we have outlined a data-driven approach that we believe could serve as a useful tool to exclude dyes adsorbed on TiO_2_ that are likely to exhibit undesirable photosensitization behaviour. We have shown that the approach can indicate the exact nature of the spectral shift in 70–80% of the dyes inspected. The predictive models afford a higher reliability than any experienced human expert. In addition, the advantages in terms of speed and versatility will certainly outweigh any possible gains that may be achieved with the more time-consuming DFT-based approaches. The models can be easily integrated into material screening frameworks that allow for the rapid computational assessment of candidate structures.

## Supplementary information

Supplementary Information

## Data Availability

The data used in the article were manually extracted from literature. The molecular structures (in SMILES format), values of the spectral shift and corresponding references have been made available in the Supplementary Information. Scripts used in the calculation of the molecular descriptors are available upon request.
