# Supplementary material for: Prediction of Absorption Spectrum Shifts in Dyes Adsorbed on Titania
Source: Sci Rep. 2019 Nov 18;9:16983. doi: 10.1038/s41598-019-53534-2 (PMC6861231; doi:10.1038/s41598-019-53534-2)
Supplement: Supplementary file 1 — Supplementary Information [file 41598_2019_53534_MOESM1_ESM.pdf]

Supplementary Information I

Prediction of Absorption Spectrum Shifts in Dyes Adsorbed on Titania

Vishwesh Venkatraman*, Amsalu Efrem Yemene, and John de Mello

Department of Chemistry, Norwegian University of Science and Technology (NTNU), 7491 Trondheim, Norway

Table S1: Molecular structures (SMILES) of the dyes, solvent, size of the spectral shift (difference between the absorption maxima in solution and dye-metal oxide) and associated references.

SMILES	SOLVENT	SHIFT (nm)	DOI
N#C/C(=C\ c1ccc(cc1)n1c2ccccc2c2c1cccc2)/C(=O)O	acetonitrile	-10	10.1021/ol9022936
N#C/C(=C\c1ccc(s1)/C=C/ c1ccc(cc1)n1c2ccc(cc2c2c1ccc(c2)C(C)(C)C)C(C)(C)C)/C(=O)O	acetonitrile	18	10.1021/ol9022936
N#C/C(=C\c1ccc2c(c1)c1ccccc1n2CC)/ C(=O)O	acetonitrile	-23	10.3390/ijms11010329
N#C/C(=C\c1ccc2c(c1)CCc1c(N2CC)cccc1)/ C(=O)O	acetonitrile	-46	10.3390/ijms11010329
CCN1c2ccc(cc2Sc2c1cccc2)/C=C(/C(=O)O)\ C#N	acetonitrile	-26	10.3390/ijms11010329
CCN1c2ccc(cc2Sc2c1cccc2)/C=C/C(=O)O	acetonitrile	-31	10.3390/ijms11010329
OC(=O)c1ccc(cc1)c1ccccc1c1cc2cc3c4c(c2oc1=O)C(C)(C)CCN4CCC3(C)C	acetonitrile	-15	10.1039/C6RA17930D
OC(=O)c1ccc(cc1)c1cccc(c1)c1cc2cc3c4c(c2oc1=O)C(C)(C)CCN4CCC3(C)C	acetonitrile	-15	10.1039/C6RA17930D
OC(=O)c1ccc(cc1)c1ccc(cc1)c1cc2cc3c4c(c2oc1=O)C(C)(C)CCN4CCC3(C)C	acetonitrile	-17	10.1039/C6RA17930D
N#C/C(=C\ c1ccc(cc1)c1ccc(cc1)N(c1ccc(cc1)N1c2ccccc2Sc2c1cccc2)c1ccc(cc1)N1c2ccccc2Sc2c1cccc2)/C(=O)O	acetonitrile	0	10.1039/C2JM34682F
N#C/C(=C\ c1ccc(s1)c1ccc(cc1)N(c1ccc(cc1)N1c2ccccc2Sc2c1cccc2)c1ccc(cc1)N1c2ccccc2Sc2c1cccc2)/C(=O)O	acetonitrile	-12	10.1039/C2JM34682F
N#C/C(=C\ c1ccc(o1)c1ccc(cc1)N(c1ccc(cc1)N1c2ccccc2Sc2c1cccc2)c1ccc(cc1)N1c2ccccc2Sc2c1cccc2)/C(=O)O	acetonitrile	-2	10.1039/C2JM34682F
O=C1OC(=O)c2c3c1ccc1c3c(cc2)c2c3c1cccc3c(cc2)N(C)C	acetonitrile	101	10.1039/B203260K
CCCCCCCCN(c1ccc2c3c1cccc3c1c3c2ccc2c3c(cc1)C(=O)OC2=O)CCCCCCCC	acetonitrile	108	10.1039/B203260K
CCCCCCCCN(c1ccc2c3c1cccc3c1c3c2ccc2c3c(cc1)c(=O)n(c2=O)CC(=O)O)CCCCCCCC	acetonitrile	-31	10.1039/B203260K
N#CC(=Cc1ccc(s1)C=Cc1ccc(cc1)N(c1ccccc1)c1ccccc1)C(=O)O	acetonitrile	32	10.1039/b603002e
N#C/C(=C/c1ccc(s1)/C=C/	acetonitrile	-15	10.1021/jp067872t

SMILES	SOLVENT	SHIFT (nm)	DOI
c1ccc(s1)c1cc2cc3c4c(c2oc1=O)C(C)(C)CCN4CCC3(C)C)/C(=O)O			
CCCCCCCCCCCCN1c2ccccc2C(/C/1=C\C1=C(O)C(=O)C1=O)(C)C	acetonitrile	-19	10.1016/j.dyepig.2019.02.028
CCCCCCCCCCCC[N+]1=C(CC2=C([O-])C(=O)/C/2=C(/C(=O)OCC)\C#N)C(c2c1cccc2)(C)C	acetonitrile	-18	10.1016/j.dyepig.2019.02.028
CCOC(=O)C[N+]1=C(CC2=C([O-])C(=O)/C/2=C(/C(=O)OCC)\C#N)C(c2c1cccc2)(C)C	acetonitrile	-26	10.1016/j.dyepig.2019.02.028
CCCCCCCCCCCCn1c2N(C)C=CC(c2c2c1cccc2)CC1=C(C(=O)C1=O)OCC	acetonitrile	7	10.1016/j.dyepig.2019.02.028
CCCCC(CC1(CC(CCCC)CC)c2cc(sc2c2c1cc(s2)c1ccc(c2c1nsn2)c1ccc(cc1)N(c1ccc(cc1)c1ccc(cc1OCC(CCCC)CC)OCC(CCCC)CC)c1ccc(cc1)c1ccc(cc1OCC(CCCC)CC)OCC(CCCC)CC)c1ccc(cc1)/C=C(/C(=O)O)\C#N)CC	acetonitrile	-27	10.1016/j.dyepig.2019.02.028
COc1cc(ccc1/C=C/1\SC(=S)N(C1=O)CC(=O)O)N(c1ccccc1)c1ccccc1	acetonitrile	52	10.1016/j.orgel.2018.11.041
COc1c(/C=C/2\SC(=S)N(C2=O)CC(=O)O)cc2c3c1CCCN3CCC2	acetonitrile	73	10.1016/j.orgel.2018.11.041
COc1cc2c(cc1/C=C/1\SC(=S)N(C1=O)CC(=O)O)c1c(n2CC)cccc1	acetonitrile	74	10.1016/j.orgel.2018.11.041
CCCCCCc1cc(sc1c1ccc(cc1)N(c1ccc2c(c1)n(CCCCCC)c(=S)n2CCCCCC)c1ccc2c(c1)n(CCCCCC)c(=S)n2CCCCCC)c1sc(c(c1)CCCCCC)/C=C(/C(=O)O)\C#N	acetonitrile	8	10.1016/j.solener.2019.04.090
CCCCCCn1c2cc(ccc2n(c1=S)CCCCCC)N(c1ccc2c(c1)n(CCCCCC)c(=S)n2CCCCCC)c1ccc(cc1)c1sc(cc1c1ccc(cc1)CCCC)c1cc(c(s1)/C=C(/C(=O)O)\C#N)c1ccc(cc1)CCCC	acetonitrile	11	10.1016/j.solener.2019.04.090
CCCCCCn1c2cc(ccc2n(c1=S)CCCCCC)N(c1ccc2c(c1)n(CCCCCC)c(=S)n2CCCCCC)c1ccc(cc1)c1sc(cc1c1ccc(cc1)OCC)c1cc(c(s1)/C=C(/C(=O)O)\C#N)c1ccc(cc1)OCC	acetonitrile	14	10.1016/j.solener.2019.04.090
OC(=O)c1ccc2c(c1)C(C)(C)C(=[N+]2C)/C=C/1\C(=C(C1=O)c1cc2c3c(c1)C(C)(C)c1c4N3c3c(C2(C)C)cccc3C(c4ccc1)(C)C)[O-]	acetonitrile	19	10.1039/C6TA08651A
CCCCCC[N+]1=C(/C=C/2\C(=C(C2=O)c2cc3c4c(c2)C(C)(C)c2c5N4c4c(C3(C)C)cccc4C(c5ccc2)(C)C)[O-])C(c2c1ccc(c2)C(=O)O)(C)C	acetonitrile	32	10.1039/C6TA08651A
CCCCCC[N+]1=C(/C=C/2\	acetonitrile	38	10.1039/C6TA08651A

SMILES	SOLVENT	SHIFT (nm)	DOI
C(=C(C2=O)c2cc3c4c(c2)C(C)(C)c2c5N4c4c(C3(C)C)cccc4C(c5ccc2)(C)C)[O-])C(c2c1ccc(c2)C(=O)O)(CCCCCCCCCC)CCCCCCCCCC			
CCCCCC[N+]1=C(/C=C/2\C(=C(C2=O)c2ccc(cc2)N(c2ccccc2)c2ccccc2)[O-])C(c2c1ccc(c2)C(=O)O)(C)C	acetonitrile	8	10.1039/C6TA08651A
CCCCCCc1cc(sc1c1sc(c(c1)CCCCCC)c1ccc2c(c1)c1ccccc1n2CC)c1sc(cc1CCCCCC)/C=C(/C(=O)O)\C#N	chloroform	14	10.1021/am402036j
CCCC[C@H](Cc1cc(sc1c1sc(c(c1)C[C@H](CCCC)CC)c1ccc2c(c1)c1ccccc1n2CC)c1sc(cc1C[C@H](CCCC)CC)/C=C(\C(=O)O)/C#N)CC	chloroform	13	10.1021/am402036j
CCCCCCc1cc(sc1c1sc(c(c1)CCCCCC)/C=C(/C(=O)O)\C#N)c1sc(cc1CCCCCC)c1ccc2c(c1)c1ccccc1n2CC	chloroform	17	10.1021/am402036j
CCCC[C@@H](Cc1cc(sc1c1sc(c(c1)C[C@H](CCCC)CC)/C=C(/C(=O)O)\C#N)c1sc(cc1C[C@H](CCCC)CC)c1ccc2c(c1)c1ccccc1n2CC)CC	chloroform	16	10.1021/am402036j
N#C/C(=C\c1ccc(s1)c1cc2c(s1)cc(s2)c1ccc(cc1)N(c1ccc2c(c1)C(C)(C)c1c2cccc1)c1ccc2c(c1)C(C)(C)c1c2cccc1)/C(=O)O	chloroform	28	10.1021/am405686z
N#C/C(=C\c1sc(c2c1OCCO2)c1cc2c(s1)cc(s2)c1ccc(cc1)N(c1ccc2c(c1)C(C)(C)c1c2cccc1)c1ccc2c(c1)C(C)(C)c1c2cccc1)/C(=O)O	chloroform	-4	10.1021/am405686z
CCCCCCCCN1C(=NC2=C3C=CC=CC3=C3C=CC=CC3=C12)C1=CC=C(C=C1)C1=C(C)C2=C(CCCCC)C3=C(C)C(C=C(C#N)C(O)=O)=C(C)N3[B-](F)(F)[N+]2=C1C	chloroform	9	10.1016/j.dyepig.2012.01.011
CCCCCCCCN1C(=NC2=C3C=CC=CC3=C3C=CC=CC3=C12)C1=CC=C(S1)C1=C(C)C2=C(CCCCC)C3=C(C)C(C=C(C#N)C(O)=O)=C(C)N3[B-](F)(F)[N+]2=C1C	chloroform	9	10.1016/j.dyepig.2012.01.011
CCCCCCCCN1C(=NC2=C3C=CC(OC)=CC3=C3C=C(OC)C=CC3=C12)C1=CC=C(C=C1)C1=C(C)C2=C(CCCCC)C3=C(C)C(C=C(C#N)C(O)=O)=C(C)N3[B-](F)(F)[N+]2=C1C	chloroform	9	10.1016/j.dyepig.2012.01.011
CCCCCCCCN1C(=NC2=C3C=CC(OC)=CC3=C3C=C(OC)C=CC3=C12)C1=CC=C(S1)C1=C(C)C2=C(CCCCC)C3=C(C)C(C=C(C#N)C(O)=O)=C(	chloroform	9	10.1016/j.dyepig.2012.01.011

SMILES	SOLVENT	SHIFT (nm)	DOI
C)N3[B-](F)(F)[N+]2=C1C			
CCCC[C@@H](Cn1c(c2ccc(cc2)c2cc3n(C[C@H](CCCC)CC)c4c5c3c3c2cccc3c2c5c(cc4)ccc2)c2c(c1=O)c(n(c2=O)C[C@H](CCCC)CC)c1ccc(cc1)c1ccc(s1)/C=C(/C(=O)O)\C#N)CC	chloroform	12	10.1039/C4CC08539F
CCCC[C@@H](Cn1c(c2ccc(cc2)c2cc3n(C[C@H](CCCC)CC)c4c5c3c3c2cccc3c2c5c(c(c4)c3ccc(cc3)OC)ccc2)c2c(c1=O)c(n(c2=O)C[C@H](CCCC)CC)c1ccc(cc1)c1ccc(s1)/C=C(\C(=O)O)/C#N)CC	chloroform	14	10.1039/C4CC08539F
CCCC[C@@H](Cn1c(c2ccc(cc2)c2cc3n(C[C@H](CCCC)CC)c4c5c3c3c2cccc3c2c5c(c(c4)OC)ccc2)c2c(c1=O)c(n(c2=O)C[C@H](CCCC)CC)c1ccc(cc1)c1ccc(s1)/C=C(/C(=O)O)\C#N)CC	chloroform	17	10.1039/C4CC08539F
CCCC[C@H](Cn1c(c2ccc(cc2)c2cc3n(C[C@H](CCCC)CC)c4c5c3c3c2cccc3c2c5c(c(c4)OCCCCCC)ccc2)c2c(c1=O)c(n(c2=O)C[C@H](CCCC)CC)c1ccc(cc1)c1ccc(s1)/C=C(/C(=O)O)\C#N)CC	chloroform	17	10.1039/C4CC08539F
N#C/C(=C\c1ccc(s1)c1ccc(s1)c1ccc(s1)c1cc2c(s1)c1[nH]c3c(c1cc2)cccc3)/C(=O)O	chloroform	-14	10.1039/C4qo00285g
N#C/C(=C\c1ccc(s1)c1ccc(s1)c1ccc(s1)c1sc2c(c1)ccc1c2n(c2c1cccc2)c1cc(c(cc1C(C)(C)C)OC)C(C)(C)C)/C(=O)O	chloroform	-12	10.1039/C4qo00285g
CCCCCCc1cc(sc1c1cc(c(s1)c1sc2c(c1)cc(c1c2n(CC)c2c1cccc2)c1ccccc1)CCCCCC)c1sc(cc1CCCCCC)/C=C(/C(=O)O)\C#N	chloroform	-51	10.1039/C4qo00285g
CCCCCCCCN1C2=CC=CC=C2C2=C1C=CC(=C2)C1=C(C)C2=C(CCCCC)C3=C(C)C(\C=C(/C#N)C(O)=O)=C(C)N3[B-](F)(F)[N+]2=C1C	chloroform	35	10.1039/C7RA04402J
CCCCCC1=C2C(C)=C(C(C)=[N+]2[B-](F)(F)N2C(C)=C(\C=C(/C#N)C(O)=O)C(C)=C12)C1=CC2=C(C=C1)N(C1=CC=CC=C21)C1=CC=C(OC)C=C1	chloroform	27	10.1039/C7RA04402J
CCCCCCCCN1C2=CC=CC=C2C2=C1C=C(C=C2)C1=C(C)C2=C(CCCCC)C3=C(C)C(\C=C(/C#N)C(O)=O)=C(C)N3[B-](F)(F)[N+]2=C1C	chloroform	30	10.1039/C7RA04402J

SMILES	SOLVENT	SHIFT (nm)	DOI
CCCCCC1=C2C(C)=C(C(C)=[N+]2[B-](F)(F)N2C(C)=C(\C=C(/C#N)C(O)=O)C(C)=C12)C1=CC2=C(C=C1)C1=CC=CC=C1N2C1=CC=C(OC)C=C1	chloroform	19	10.1039/C7RA04402J
N#C/C(=C\c1sc(cc1C)c1ccc2c(c1)ccn2c1ccc(cc1)N(c1ccc(cc1)C)c1ccc(cc1)C)/C(=O)O	chloroform	22	10.1016/j.jpowsour.2013.09.106
N#CSc1cn(c2c1cc(cc2)c1cc(c(s1)/C=C(/C(=O)O)\C#N)C)c1ccc(cc1)N(c1ccc(cc1)C)c1ccc(cc1)C	chloroform	28	10.1016/j.jpowsour.2013.09.106
N#C/C(=C\c1ccc(s1)c1cnc(c2c1nsn2)c1ccc2c(c1)[C@@H]1CCC[C@@H]1N2c1ccc(cc1)C)/C(=O)O	chloroform	60	10.1021/jp501173b
CCCC[C@H](C[C@@]1(C[C@H](CCCC)CC)c2cc(sc2c2c1cc(s2)c1ccc(s1)/C=C(/C(=O)O)\C#N)c1cnc(c2c1nsn2)c1ccc2c(c1)[C@H]1CCC[C@H]1N2c1ccc(cc1)C)CC	chloroform	35	10.1021/jp501173b
CCCCCCc1cc(sc1/C=C(/C(=O)O)\C#N)c1ccc(s1)c1ccc2c(c1)Sc1c(N2C[C@H](CCCC)CC)cccc1	chloroform	44	10.1016/j.dyepig.2015.05.012
CCCCCCc1cc(sc1/C=C(/C(=O)O)\C#N)c1ccc(c2c1nsn2)c1ccc2c(c1)Sc1c(N2C[C@H](CCCC)CC)cccc1	chloroform	87	10.1016/j.dyepig.2015.05.012
CCCCCCc1cc(sc1/C=C(/C(=O)O)\C#N)c1ccc(c2c1nsn2)c1ccc2c(c1)Sc1c(N2c2ccc(cc2)N(c2ccc(cc2)C)c2ccc(cc2)C)cccc1	chloroform	25	10.1016/j.dyepig.2015.05.012
CCCCCC1C2C(C)=C(C(C)=[N+]2[B-](F)(F)N2C(C)=C(C(C)=C12)C1=CC=C(S1)\C=C(\C#N)C(O)=O)C1=CC=C2N(CCC)C3=C(SC2=C1)C=CC=C3	chloroform	1	10.1016/j.orgel.2014.05.024
CCCCCC1C2C(C)=C(C(C)=[N+]2[B-](F)(F)N2C(C)=C(C(C)=C12)C1=CC=C(S1)C=C1SC(=S)N(CC(O)=O)C1=O)C1=CC=C2N(CCC)C3=C(SC2=C1)C=CC=C3	chloroform	-4	10.1016/j.orgel.2014.05.024
CCCCCC1C2C(C)=C(C(C)=[N+]2[B-](F)(F)N2C(C)=C(C(C)=C12)C1=CC=C(O1)\C=C(\C#N)C(O)=O)C1=CC=C2N(CCC)C3=C(SC2=C1)C=CC=C3	chloroform	4	10.1016/j.orgel.2014.05.024
CCCCCC1C2C(C)=C(C(C)=[N+]2[B-](F)(F)N2C(C)=C(C(C)=C12)C1=CC=C(O1)C=C1SC(=S)N(CC(O)=O)C1=O)C1=CC=C2N(CCC)C3=C(SC2=C1)C=CC=C3	chloroform	-1	10.1016/j.orgel.2014.05.024

SMILES	SOLVENT	SHIFT (nm)	DOI
CCCCCC1=C2C(=C(C(=[N+]2[B-](n2c1c(C)c(c2C)/C=C(/C(=O)O)\C#N)(F)F)C)c1ccc2c(c1)Sc1c(N2CCC)cccc1)C	chloroform	2	10.1016/j.jpowsour.2014.05.079
CCCCCC1=C2C(=C(C(=[N+]2[B-](n2c1c(C)c(c2C)/C=C(/C(=O)O)\C#N)(F)F)C)c1ccc2c(c1)Sc1c(N2CCCCCC)cccc1)C	chloroform	1	10.1016/j.jpowsour.2014.05.079
CCCCCC1=C2C(=C(C(=[N+]2[B-](n2c1c(C)c(c2C)/C=C(/C(=O)O)\C#N)(F)F)C)c1ccc2c(c1)Sc1c(N2CCCCCCN2c3ccccc3Sc3c2ccc(c3)C2=C(C)C3=C(CCCCC)c4n([B-]([N+]3=C2C)(F)F)c(c(c4C)/C=C(/C(=O)O)\C#N)C)cccc1)C	chloroform	5	10.1016/j.jpowsour.2014.05.079
CCCCCC1=C2C(=C(C(=[N+]2[B-](n2c1c(C)c(c2C)c1ccc(s1)/C=C(/C(=O)O)\C#N)(F)F)C)c1ccc2c(c1)Sc1c(N2CCCCCCN2c3ccccc3Sc3c2ccc(c3)C2=C(C)C3=C(CCCCC)c4n([B-]([N+]3=C2C)(F)F)c(c(c4C)/C=C(/C(=O)O)\C#N)C)cccc1)C	chloroform	7	10.1016/j.jpowsour.2014.05.079
CCCCc1nc2c3cc(sc3c3c(c2nc1CCCCC)cc(s3)/C=C(/C(=O)O)\C#N)c1ccc(cc1)N(c1ccccc1)c1ccccc1	chloroform	44	10.1021/acsami.5b06404
CCCCCCOc1ccc(cc1)N(c1ccc(cc1)OCCCCCC)c1ccc(cc1)c1sc2c(c1)c1nc(CCCCC)c(nc1c1c2sc(c1)/C=C(/C(=O)O)\C#N)CCCCC	chloroform	70	10.1021/acsami.5b06404
CCCCCCOc1ccc(cc1)N(c1ccc(cc1)OCCCCCC)c1ccc(cc1)c1sc(c2c1OCCO2)c1sc2c(c1)c1nc(CCCCC)c(nc1c1c2sc(c1)/C=C(/C(=O)O)\C#N)CCCCC	chloroform	56	10.1021/acsami.5b06404
CCCCCCOc1ccc(cc1)N(c1ccc(cc1)OCCCCCC)c1ccc(cc1)c1ccc(c2c1nsn2)c1sc2c(c1)c1nc(CCCCC)c(nc1c1c2sc(c1)/C=C(/C(=O)O)\C#N)CCCCC	chloroform	10	10.1021/acsami.5b06404
CCCCc1nc2c3cc(sc3c3c(c2nc1CCCCC)cc(s3)/C=C(/C(=O)O)\C#N)c1ccc(c2c1nsn2)c1ccc2c(c1)[C@@H]1CCC[C@@H]1N2c1ccc(cc1)C	chloroform	28	10.1021/acsami.5b06404
CCCCCCCCOc1c(/C=C/c2ccc(cc2)N(c2ccc(cc2)C)c2ccc(cc2)C)cc(cc1/C=C/c1ccc(cc1)N(c1ccc(cc1)C)c1ccc(cc1)C)c1ccc(s1)c1c(F)c(F)c(c2c1nsn2)c1ccc(cc1)C(=O)O	chloroform	4	10.1016/j.dyepig.2017.06.027
CCCC[C@@H](Cn1c(c2ccc(s2)c2cc(/C=C/c3ccc(cc3)N(c3ccc(cc3)C)c3ccc(cc3)C)c(c(c2)/C=C/	chloroform	12	10.1016/j.dyepig.2017.06.027

SMILES	SOLVENT	SHIFT (nm)	DOI
c2ccc(cc2)N(c2ccc(cc2)C)c2ccc(cc2)C)OCCCCCCCC)c2c(c1=O)c(n(c2=O)C[C@H](CCCC)CC)c1ccc(s1)c1ccc(cc1)C(=O)O)CC			
CCCCCCCCc1nc2c(ccc(c2nc1CCCCCCC)c1ccc(s1)/C=C(/C(=O)O)\C#N)c1ccc(s1)c1ccc(cc1)N(c1ccccc1)c1ccccc1	chloroform	34	10.1016/j.dyepig.2017.05.054
CCCCCCCCc1ccc(s1)c1nc2c(nc1c1ccc(s1)CCCCCCCCC)c(ccc2c1ccc(s1)/C=C(/C(=O)O)\C#N)c1ccc(s1)c1ccc(cc1)N(c1ccccc1)c1ccccc1	chloroform	24	10.1016/j.dyepig.2017.05.054
CCCCCCCCc1sc2c(c1)c1cc(sc1c1c2nc2c(ccc(c2n1)c1ccc(s1)/C=C(/C(=O)O)\C#N)c1ccc(s1)c1ccc(cc1)N(c1ccccc1)c1ccccc1)CCCCCCCC	chloroform	3	10.1016/j.dyepig.2017.05.054
CCCCCCn1c2ccc(cc2c2c1cccc2)c1c(/C=C(/C(=O)O)\C#N)sc(c1c1ccc2c(c1)c1ccccc1n2CCCCCC)/C=C(/C(=O)O)\C#N	chloroform	-94	10.1016/j.orgel.2017.07.019
N#C/C(=C/c1sc(c(c1c1ccc(cc1)N(c1ccccc1)c1ccccc1)c1cccc(c1)N(c1ccccc1)c1ccccc1)/C=C(/C(=O)O)\C#N)/C(=O)O	chloroform	-94	10.1016/j.orgel.2017.07.019
Cc1ccc(cc1)C1(c2ccc(cc2)C)c2cc(ccc2c2c1cc(s2)c1ccc(s1)c1ccc(cc1)C(=O)O)N(c1ccc(cc1)C)c1ccc(cc1)C	chloroform	11	10.1016/j.dyepig.2017.01.026
Cc1ccc(cc1)C1(c2ccc(cc2)C)c2cc(ccc2c2c1cc(s2)c1ccc(s1)c1ccc(c2c1nsn2)c1ccc(cc1)C(=O)O)N(c1ccc(cc1)C)c1ccc(cc1)C	chloroform	4	10.1016/j.dyepig.2017.01.026
CCCC[C@@H](Cn1c(=O)c2c(c1c1ccc(s1)c1sc3c(c1)C(c1c3ccc(c1)N(c1ccc(cc1)C)c1ccc(cc1)C)(c1ccc(cc1)C)c1ccc(cc1)C)c(=O)n(c2c1ccc(s1)c1ccc(cc1)C(=O)O)C[C@@H](CCCC)CC)CC	chloroform	6	10.1016/j.dyepig.2017.01.026
CCCCCC1=C2C(C)=C(C(C)=[N+]2[B-](F)(F)N2C(C)=C(\C=C(\C#N)C(O)=O)C(C)=C12)C1=CC=C(C=C1)N(C1=CC=CC=C1)C1=CC=CC=C1	chloroform	16	10.1016/j.dyepig.2017.02.017
CCCCCC1=C2C(C)=C(C(C)=[N+]2[B-](F)(F)N2C(C)=C(C(C)=C12)C1=CC=C(O1)\C=C(/C#N)C(O)=O)C1=CC2=C(C=C1)C1=CC(OC)=CC=C1N2CCC	chloroform	12	10.1016/j.dyepig.2017.02.017
CCCCCC1=C2C(C)=C(C(C)=[N+]2[B-](F)(F)N2C(C)=C(C(C)=C12)C1=CC=C(O1)\C=C(/C#N)C(O)=O)C1=CC=C(C=C1)N(C1=CC=CC=	chloroform	12	10.1016/j.dyepig.2017.02.017

SMILES	SOLVENT	SHIFT (nm)	DOI
C1)C1=CC=CC=C1			
CCCCOc1ccc(cc1)N(c1ccc(cc1)OCCCC)c1ccc(cc1)c1ccc(s1)/C=C(/C(=O)O)\C#N	chloroform	62	10.1016/j.dyepig.2010.03.034
CCCCOc1ccc(cc1)N(c1ccc(cc1)c1ccc(s1)/C=C(/C(=O)O)\C#N)c1ccc(cc1)c1ccc(s1)/C=C(/C(=O)O)\C#N	chloroform	43	10.1016/j.dyepig.2010.03.034
N(C1=CC=C(C=C1)C1=CC=C(S1)C=C(C(=O)O)C#N)(C1=CC=C(C=C1)C1=CC=C(S1)C=C(C(=O)O)C#N)C1=CC=C(C=C1)C1=CC=C(S1)C=C(C(=O)O)C#N	chloroform	24	10.1016/j.dyepig.2010.03.034
N#C/C(=C\[C@@H]1COc2c(O1)c(sc2COCCCCCCCCCOCc1sc(c2c1OC[C@H](O2)/C=C(/C(=O)O)\C#N)c1ccc(cc1)N(c1ccccc1)c1ccccc1)c1ccc(cc1)N(c1ccccc1)c1ccccc1)/C(=O)O	chloroform	82	10.1039/C4TA03451A
N#C/C(=C\c1sc(c2c1O[C@@H](COCCCCCCCCCOC[C@H]1COc3c(O1)c(sc3/C=C(/C(=O)O)\C#N)c1ccc(cc1)N(c1ccccc1)c1ccccc1)CO2)c1ccc(cc1)N(c1ccccc1)c1ccccc1)/C(=O)O	chloroform	78	10.1039/C4TA03451A
N#C/C(=C\[C@@H]1COc2c(O1)c(COCCCCCCCCCOCc1sc(c3c1O[C@@H](CO3)/C=C(/C(=O)O)\C#N)c1ccc(cc1)N(c1ccccc1)c1ccccc1)sc2c1ccc(cc1)N(c1ccccc1)c1ccccc1)/C(=O)O	chloroform	74	10.1039/C4TA03451A
COC[C@@H]1COc2c(O1)c(sc2/C=C(/C(=O)O)\C#N)c1ccc(cc1)N(c1ccccc1)c1ccccc1	chloroform	84	10.1039/C4TA03451A
COC[C@@H]1COc2c(O1)c(/C=C(/C(=O)O)\C#N)sc2c1ccc(cc1)N(c1ccccc1)c1ccccc1	chloroform	81	10.1039/C4TA03451A
CCCCCCCCCC[C@]1(CCCCCCCC)C(=[N+](c2c1cccc2)CCCCCC)/C=C/1\C(=C(C1=O)/C=C/1\N(C)c2c(C1(C)C)cc(cc2)c1ccc(cc1)/C=C(/C(=O)O)\C#N)[O-]	chloroform	-15	10.1021/acsami.7b08346
CCCCCCCCCC[C@]1(CCCCCCCC)c2cc(ccc2N(/C/1=C/C1=C([O-])/C(=C\C2=[N+](C)c3c(C2(C)C)cccc3)/C1=O)CCCCCCC)c1ccc(cc1)/C=C(/C(=O)O)\C#N	chloroform	-13	10.1021/acsami.7b08346
CCCCCCCCCC[C@@]1(CCCCCCCC)C(=[N+](c2c1cccc2)CCCCCC)/C=C/1\C(=C(C1=O)/C=C\1/N(C)c2c(C1(C)C)cc(cc2)c1ccc(s1)/C=C(/C(=O)O)\C#N)[O-]	chloroform	-13	10.1021/acsami.7b08346

SMILES	SOLVENT	SHIFT (nm)	DOI
CCCCCCCCCC[C@]1(CCCCCCCC)c2cc(ccc2N(/C/1=C/C1=C([O-])/C(=C\C2=[N+](C)c3c(C2(C)C)cccc3)/C1=O)CCCCCC)c1ccc(s1)/C=C(/C(=O)O)\C#N	chloroform	-15	10.1021/acsami.7b08346
CCCCCCCCCC[C@]1(CCCCCCCC)C(=[N+](c2c1cccc2)CCCCCCCCCCC)/C=C/1\C(=C(C1=O)/C=C/1\N(C)c2c(C1(C)C)cc(cc2)c1ccc(s1)/C=C(/C(=O)O)\C#N)[O-]	chloroform	-15	10.1021/acsami.7b08346
CCN(c1ccc2c(c1)ccc(n2)/C=C(/C(=O)O)\C#N)CC	chloroform	18	10.1016/j.saa.2017.09.002
N#C/C(=C\c1ccc2c(n1)ccc(c2)n1c2ccc(cc2c2c1ccc(c2)OC)OC)/C(=O)O	chloroform	14	10.1016/j.saa.2017.09.002
CCCCOc1ccc(cc1)N(c1ccc2c(c1)ccc(n2)/C=C(\C(=O)O)/C#N)c1ccc(cc1)OCCCC	chloroform	6	10.1016/j.saa.2017.09.002
O=C1NC(=O)/C(=C\c2ccc(s2)c2ccc(c3c2nsn3)c2ccc(cc2)N(c2ccccc2)c2ccccc2)/N1	chloroform	8	10.1002/adfm.201601305
COc1ccc(cc1)N(c1ccc(cc1)OC)c1ccc(cc1)c1ccc(c2c1nsn2)c1ccc(s1)/C=C/1\NC(=O)NC1=O	chloroform	3	10.1002/adfm.201601305
COc1ccc(cc1)N(c1ccc(cc1)OC)c1ccc(cc1)c1ccc(c2c1nsn2)c1ccc(s1)/C=C(/C(=O)O)\C#N	chloroform	3	10.1002/adfm.201601305
CCCCCCc1cc(sc1c1ccc(cc1)N(c1ccccc1)c1ccccc1)c1cc(c(s1)/C=C(/C(=O)O)\C#N)CCCCCC	chloroform	-12	10.1021/sc500234a
CCCCCCOc1ccc(cc1)N(c1ccc(cc1)OCCCCCC)c1ccc(cc1)c1sc(cc1CCCCCC)c1cc(c(s1)/C=C(/C(=O)O)\C#N)OCCCCC	chloroform	-14	10.1021/sc500234a
CCCCCCc1cc(sc1c1ccc(cc1)N(c1ccccc1)c1ccccc1)c1ccc2c(c1)c(c1ccc(cc1)C(C)(C)C)c1c(c2c2ccc(cc2)C(C)(C)C)cc(cc1)c1sc(c(c1)OCCCCC)/C=C(/C(=O)O)\C#N	chloroform	-12	10.1021/sc500234a
CCCCCCOc1ccc(cc1)N(c1ccc(cc1)OCCCCCC)c1ccc(cc1)c1sc(cc1CCCCCC)c1ccc2c(c1)c(c1ccc(cc1)C(C)(C)C)c1c(c2c2ccc(cc2)C(C)(C)C)cc(cc1)c1cc(c(s1)/C=C(/C(=O)O)\C#N)OCCCCC	chloroform	-17	10.1021/sc500234a
CCCCCCCCCCCCn1c(cc2c1ccc(c2)c1ccc(s1)/C=C/1\SC(=S)N(C1=O)CC(=O)O)c1ccc(s1)/C=C/1\SC(=S)N(C1=O)CC(=O)O	chloroform	-56	10.1007/s10854-015-4251-1
CCCCCCCCCCCCn1c(cc2c1ccc(c2)c1ccc(s1)/	chloroform	10	10.1007/s10854-015-4251-1

SMILES	SOLVENT	SHIFT (nm)	DOI
C=C(/C(=O)O)\C#N)c1ccc(s1)/C=C(/C(=O)O)\C#N			
CCCCCCc1cc(sc1c1ccc(cc1)N(c1ccc(cc1)C)c1ccc(cc1)C)c1ccc2c(c1)C(=O)c1c2ccc(c1)c1cc(c(s1)/C=C(/C(=O)O)\C#N)CCCCCC	chloroform	-12	10.1016/j.jpowsour.2013.08.034
CCCCCCc1cc(sc1c1ccc(cc1)N(c1ccc(cc1)C)c1ccc(cc1)C)c1ccc2c(c1)C(=C(C#N)C#N)c1c2ccc(c1)c1cc(c(s1)/C=C(/C(=O)O)\C#N)CCCCCC	chloroform	-2	10.1016/j.jpowsour.2013.08.034
CCCCCCCCOc1ccc(cc1)C1(c2ccc(cc2)OCCCCCCCC)c2ccsc2c2c1cc1c(c2)[nH]c2c1cc1c(c2)c2c(C1(c1ccc(cc1)OCCCCCCCC)c1ccc(cc1)OCCCCCCCC)cc(s2)c1ccc(c2c1nsn2)c1ccc(cc1)C(=O)O	chloroform	4	10.1021/acsomega.7b01387
CCCCCCCCOc1ccc(cc1)C1(c2ccc(cc2)OCCCCCCCC)c2ccsc2c2c1cc1c(c2)[nH]c2c1cc1c(c2)c2c(C1(c1ccc(cc1)OCCCCCCCC)c1ccc(cc1)OCCCCCCCC)cc(s2)c1c(F)c(F)c(c2c1nsn2)c1ccc(cc1)C(=O)O	chloroform	11	10.1021/acsomega.7b01387
CCCCCCCCOc1ccc(cc1)C1(c2ccc(cc2)OCCCCCCCC)c2ccsc2c2c1cc1c(c2)[nH]c2c1cc1c(c2)c2c(C1(c1ccc(cc1)OCCCCCCCC)c1ccc(cc1)OCCCCCCCC)cc(s2)c1cnc(c2c1nsn2)c1ccc(cc1)C(=O)O	chloroform	17	10.1021/acsomega.7b01387
C(#N)C(C(=O)O)=CC=1SC(=CC1)C1=CC=C(C=C1)N(C1=CC=CC=C1)C1=CC=CC=C1	chloroform	-2	10.1016/j.dyepig.2009.04.005
CCCCCCc1cc(sc1c1ccc(cc1)N(c1ccccc1)c1ccccc1)/C=C(/C(=O)O)\C#N	chloroform	-4	10.1016/j.dyepig.2009.04.005
C(#N)C(C(=O)O)=CC1=CC=C(S1)C=1SC(=CC1)C=1SC(=CC1)C1=CC=C(C=C1)N(C1=CC=CC=C1)C1=CC=CC=C1	chloroform	37	10.1016/j.dyepig.2009.04.005
CCCCCCc1cc(sc1c1ccc(s1)c1sc(cc1CCCCCC)/C=C(/C(=O)O)\C#N)c1ccc(cc1)N(c1ccccc1)c1ccccc1	chloroform	34	10.1016/j.dyepig.2009.04.005
CCCCCCc1cc(sc1c1ccc(s1)c1sc(cc1CCCCCC)c1ccc(cc1)N(c1ccccc1)c1ccccc1)c1sc(c(c1)CCCCCC)c1ccc(s1)c1sc(cc1CCCCCC)/C=C(/C(=O)O)\C#N	chloroform	-25	10.1016/j.dyepig.2009.04.005
CCCCCCc1cc(sc1c1ccc(cc1)N(c1ccc(cc1)C)c1ccc(cc1)C)c1ccc2c(c1)C(=O)c1c2ccc(c1)c1cc(c(s1)C=C(C#N)C#N)CCCCCC	chloroform	3	10.1016/j.jpowsour.2013.08.034
N#C/C(=C/c1ccc2c(c1)sc(n2)C#Cc1ccc(cc1)N(c1ccccc1)c1ccccc1)/C(=O)O	chloroform	13	10.1021/jp509971q

SMILES	SOLVENT	SHIFT (nm)	DOI
N#C/C(=C/c1ccc2c(c1)sc(n2)/C=C/ c1ccc(cc1)N(c1ccccc1)c1ccccc1)/C(=O)O	chloroform	24	10.1021/jp509971q
N#C/C(=C\c1ccc2c(c1)sc(n2)/C=C/ c1ccc(cc1)N(c1ccc(cc1)OC)c1ccc(cc1)OC)/ C(=O)O	chloroform	26	10.1021/jp509971q
N#C/C(=C\ c1ccc(cc1)c1ccc(cc1)N(c1cccc(c1)c1cccs1)c 1cccc(c1)c1cccs1)/C(=O)O	chloroform	-5	10.1039/C2CP42993D
N#C/C(=C\ c1ccc(s1)c1ccc2c(c1)c1cc(ccc1n2CC)c1ccc(s 1)c1ccc(cc1)N(c1ccc(cc1)c1cccs1)c1ccc(cc1)c1cccs1)/C(=O)O	chloroform	-80	10.1039/C2CP42993D
CCn1c2ccc(cc2c2c1ccc(c2)c1ccc(s1)c1ccncc 1)c1ccc(s1)c1ccc(cc1)N(c1ccc(cc1)c1cccs1)c 1ccc(cc1)c1cccs1	chloroform	-78	10.1039/C2CP42993D
CCCCCc1nc2c3cc(sc3c3c(c2nc1CCCCC)cc(s3)/C=C(/C(=O)O)\C#N)c1ccc2c(c1) [C@H]1CCC[C@H]1N2c1ccc(cc1)C	chloroform	10	10.1016/ j.dyepig.2018.04.067
CCCCCc1nc2c(ccc(c2nc1CCCCC)c1ccc2c(c1) [C@H]1CCC[C@H]1N2c1ccc(cc1)C)c1cc2c(s 1)c1sc(cc1c1c2nc(CCCCC)c(n1)CCCCC)/ C=C(/C(=O)O)\C#N	chloroform	21	10.1016/ j.dyepig.2018.04.067
CCCCCc1nc2c(ncc(c2nc1CCCCC)c1ccc2c(c1) [C@H]1CCC[C@H]1N2c1ccc(cc1)C)c1cc2c(s 1)c1sc(cc1c1c2nc(CCCCC)c(n1)CCCCC)/ C=C(/C(=O)O)\C#N	chloroform	20	10.1016/ j.dyepig.2018.04.067
CCCCCCCCCCCCC1(CCCCCCCCCCCC)c2cc3c(cc2c2c1cccc2)ccc([n+]3C)C=C1C(=C(C1=O)C =C1N(C)c2c(C1(C)C)ccc(c2)C(=O)O)[O-]	chloroform	47	10.1002/chem.201803062
CCCCCCCCCCCCC1(CCCCCCCCCCCC)c2cc3c(cc2c2c1cccc2)ccc([n+]3C)C=C1C(=C(C1=O)C =C1N(C)c2c(C1(C)C)cc1c(c2)cc(cc1)C(=O)O) [O-]	chloroform	54	10.1002/chem.201803062
CCCCCCCCCCCCC1(CCCCCCCCCCCC)c2cc3c(cc2c2c1cccc2)ccc([n+]3C)C=C1C(=C(C1=O)C =c1ccc2c(n1C)ccc(c2)C(=O)O)[O-]	chloroform	134	10.1002/chem.201803062
N#C/C(=C/ c1ccc(s1)c1ccc(cc1)n1c2ccc(cc2c2c1ccc(c2) C(C)(C)C)C(C)(C)C)/C(=O)O	chloroform	-17	10.1021/jp1055842
N#C/C(=C\ c1ccc(s1)c1ccc(s1)c1ccc(cc1)n1c2ccc(cc2c2 c1ccc(c2)C(C)(C)C)C(C)(C)C)/C(=O)O	chloroform	0	10.1021/jp1055842
N#C/C(=C/1\C=C(/C=C/ c2ccc(cc2)N(c2ccc(cc2)n2c3ccccc3c3c2cccc	chloroform	15	10.1016/ j.dyepig.2011.11.005

SMILES	SOLVENT	SHIFT (nm)	DOI
3)c2ccc(cc2)n2c3ccccc3c3c2cccc3)CC(C1)(C)C)/C(=O)O			
N#C/C(=C\1/CCCC(=C1)/C=C/c1ccc2c(c1)C1CCCC1N2c1ccc(cc1)C)/C(=O)O	chloroform	67	10.1016/j.dyepig.2011.11.005
N#C/C(=C\c1ccc(cc1)N(c1ccc(cc1)c1ccc(cc1)N(c1ccccc1)c1ccccc1)c1ccc(cc1)c1ccc(cc1)N(c1ccccc1)c1ccccc1)/C(=O)O	chloroform	37	10.1016/j.dyepig.2013.04.023
N#C/C(=C\c1ccc(s1)c1ccc(cc1)N(c1ccc(cc1)c1ccc(cc1)N(c1ccccc1)c1ccccc1)c1ccc(cc1)c1ccc(cc1)N(c1ccccc1)c1ccccc1)/C(=O)O	chloroform	14	10.1016/j.dyepig.2013.04.023
N#C/C(=C\c1cc(c(s1)c1ccc(cc1)N(c1ccc(cc1)c1ccc(cc1)N(c1ccccc1)c1ccccc1)c1ccc(cc1)c1ccc(cc1)N(c1ccccc1)c1ccccc1)c1ccc(cc1)N(c1ccccc1)c1ccccc1)/C(=O)O	chloroform	-5	10.1016/j.dyepig.2013.04.023
N#C/C(=C\c1cc2c(s1)c1c(C2(CC)CC)cc(s1)c1ccc(cc1)/C=C\C(c1ccc(cc1)N(C)C)c1ccc(cc1)N(C)C)/C(=O)O	chloroform	71	10.1016/j.dyepig.2011.09.019
N#C/C(=C\c1cc2c(s1)c1c(C2(CC)CC)cc(s1)c1ccc(cc1)N(c1ccccc1)c1ccccc1)/C(=O)O	chloroform	61	10.1016/j.dyepig.2011.09.019
N#C/C(=C\c1cc2c(s1)c1c(C2(CC)CC)cc(s1)c1sc(c2c1OCCO2)c1ccc(cc1)N(c1ccccc1)c1ccccc1)/C(=O)O	chloroform	79	10.1016/j.dyepig.2011.09.019
N#C/C(=C\1/C=C(/C=C/c2ccc(cc2)N(c2ccccc2)c2ccccc2)CC(C1)(C)C)/C(=O)O	chloroform	6	10.1021/jo800159t
N#C/C(=C\1/C=C(/C=C/c2ccc(cc2)N(c2ccc(cc2)N(c2ccccc2)c2ccccc2)c2ccccc2)CC(C1)(C)C)/C(=O)O	chloroform	21	10.1021/jo800159t
N#C/C(=C\1/C=C(/C=C/c2ccc(cc2)N(c2ccc(cc2)N(c2ccccc2)c2ccccc2)c2ccc(cc2)N(c2ccccc2)c2ccccc2)CC(C1)(C)C)/C(=O)O	chloroform	43	10.1021/jo800159t
N#C/C(=C\1/C=C(/C=C/c2ccc(cc2)N(c2ccc(cc2)n2c3ccccc3c3c2cccc3)c2ccc(cc2)n2c3ccccc3c3c2cccc3)CC(C1)(C)C)/C(=O)O	chloroform	-25	10.1021/jo800159t
CCCCC(COc1c(/C=C/	chloroform	95	10.1016/

SMILES	SOLVENT	SHIFT (nm)	DOI
c2sc(cc2CCCCCC)c2ccc(c3c2nsn3)c2scc(c2)CCCCCC)cc(cc1/C=C/c1sc(cc1CCCCCC)c1ccc(c2c1nsn2)c1scc(c1)CCCCCC)c1sc(c(c1)CCCCCC)/C=C(/C(=O)O)\C#N)CC			j.dyepig.2013.01.009
CCCCCCCCOc1c(/C=C/C=C/c2ccc(cc2)N(c2ccc(cc2)C)c2ccc(cc2)C)cc(cc1/C=C/C=C/c1ccc(cc1)N(c1ccc(cc1)C)c1ccc(cc1)C)c1sc(c(c1)CCCCCC)/C=C(/C(=O)O)\C#N	chloroform	-4	10.1016/j.dyepig.2013.01.009
CCCCn1c2ccc(cc2c2c1cccc2)c1cc(c(s1)c1cc2cc3c4c(c2oc1=O)C(C)(C)CCN4CCC3(C)C)/C=C(\C(=O)O)/C#N	chloroform	29	10.1039/c2jm32333h
CCCCC1(CCCC)c2ccccc2c2c1cc(cc2)c1cc(c(s1)c1cc2cc3C(C)(C)CCN4c3c(c2oc1=O)C(C)(C)CC4)/C=C(/C(=O)O)\C#N	chloroform	6	10.1039/c2jm32333h
N#C/C(=C/c1cc(sc1c1cc2cc3c4c(c2oc1=O)C(C)(C)CCN4CCC3(C)C)C)/C(=O)O	chloroform	3	10.1039/c2jm32333h
CCCCCCOc1ccc(cc1)N(c1ccc(cc1)OCCCCCC)c1ccc(cc1)c1ccc(s1)c1ccc(s1)c1ccc(s1)/C=C(/C(=O)O)\C#N	chloroform	80	10.1021/cm302250y
CCCCCCOc1ccc(cc1)N(c1ccc(cc1)OCCCCCC)c1ccc(cc1)c1ccc(s1)c1sc(c(c1)c1ccc(s1)c1cc(sc1c1ccc(s1)c1ccc(cc1)N(c1ccc(cc1)OCCCCCC)c1ccc(cc1)OCCCCCC)c1ccc(s1)C=C(C(=O)O)C#N)c1ccc(s1)C=C(C(=O)O)C#N	chloroform	100	10.1021/cm302250y
CCCCc1ccc(cc1)n1c(nc(c1c1ccc(cc1)OC)c1ccc(cc1)OC)c1ccc2c(c1)Sc1c(N2CC)ccc(c1)C=C(C(=O)O)C#N	chloroform	35	10.1021/acs.jpcc.8b05477
CCCCN1c2ccc(cc2C(C1=CC1=C([O-])C(=CC2=[N+](CCCC)c3c(C2)cccc3)C1=C(C#N)C#N)(C)C)C(=O)O	chloroform	-7	10.1039/C2TA00883A
CCCCN1c2ccccc2C(C1=CC1=C([O-])C(=CC2=[N+](CCCC)c3c(C2)cccc3)C1=C(C#N)C#N)(C)C	chloroform	7	10.1039/C2TA00883A
CCCCN1c2ccccc2C(C1=CC1=C([O-])C(=CC2=[N+](CCCC)c3c(C2)cccc3)C1=O)(C)C	chloroform	-1	10.1039/C2TA00883A
CCCCCCc1cc(sc1c1ccc(cc1)N(c1ccc(cc1)OCCCC)c1ccc(cc1)OCCCC)c1ccc2c(c1)cc[n+](c2)CC(=O)O	chloroform	37	10.1039/C2TA00905F
CCCCCCc1cc(sc1c1ccc(cc1)N(c1ccc(cc1)OCCCC)c1ccc(cc1)OCCCC)c1sc(cc1CCCCCC)c1sc(cc1CCCCCC)c1ccc2c(c1)cc[n+](c2)CC(=O)O	chloroform	17	10.1039/C2TA00905F

SMILES	SOLVENT	SHIFT (nm)	DOI
CCCCCCc1cc(sc1c1ccc(cc1)N(c1ccc(cc1)c1ccc(cc1OCCCC)OCCCC)c1ccc(cc1)c1ccc(cc1OCCCC)OCCCC)c1ccc2c(c1)cc[n+](c2)CC(=O)O	chloroform	39	10.1039/C2TA00905F
CCCCCCc1cc(sc1c1ccc(cc1)N(c1ccc(cc1)c1ccc(cc1OCCCC)OCCCC)c1ccc(cc1)c1ccc(cc1OCCCC)OCCCC)c1sc(cc1CCCCCC)c1sc(cc1CCCCCC)c1ccc2c(c1)cc[n+](c2)CC(=O)O	chloroform	31	10.1039/C2TA00905F
COC1=CC=C(C=C1)N(C1=CC=C(C=C1)/C=C(\C#N)/C1=CC=C(S1)C1=CC=C(S1)/C=C(/C(=O)O)\C#N)C1=CC=C(C=C1)OC	chloroform	65	10.1021/jp807191w
C(#N)C(C(=O)O)=CC=1SC(=C2OCCOC21)C=CC2=CC=C(C=C2)N(C2=CC=CC=C2)C2=CC=CC=C2	chloroform	52	10.1002/aenm.201200435
CC(C)(C)C1=CC2=C3N4C5=C(C=C(C=CC6=C7OCCOC7=C(S6)C=C(C#N)C(O)=O)C=C5C(C)(C)C3=C1)C(C)(C)C1=C4C(=CC(=C1)C(C)(C)C)C2(C)C	chloroform	66	10.1002/aenm.201200435
CCCCCCC1(CCCCCC)C2=C(SC(\C=C\C3=CC4=C5N6C7=C(C=C(C=C7C(C)(C)C5=C3)C(C)(C)C)C(C)(C)C3=CC(=CC(=C63)C4(C)C)C(C)(C)C)=C2)C2=C1C=C(S2)\C=C(\C#N)C(O)=O	chloroform	73	10.1002/aenm.201200435
CCCCCCc1cc(sc1c1ccc(s1)c1sc(cc1CCCCCC)/C=C(/C(=O)O)\C#N)c1ccc2c(c1)c1ccccc1n2CC	chloroform	52	10.1016/j.electacta.2018.08.068
CCCCCCc1cc(sc1c1ccc(s1)c1sc(cc1CCCCCC)/C=C(/C(=O)O)\C#N)c1ccc2c(c1)c1ccccc1o2	chloroform	36	10.1016/j.electacta.2018.08.068
CCCCCCc1cc(sc1c1ccc(s1)c1sc(cc1CCCCCC)/C=C(/C(=O)O)\C#N)c1ccc2c(c1)c1ccccc1s2	chloroform	37	10.1016/j.electacta.2018.08.068
CCCCCCn1c2ccc(cc2c2c1ccc(c2)/C=C(\C(=O)O)/C#N)c1ccc(cc1)/C=C(\C(=O)O)/C#N	chloroform	71	10.1039/c7pp00351j
CCCCCCn1c2ccc(cc2c2c1ccc(c2)/C=C/1\SC(=O)NC1=O)c1ccc(cc1)OC	chloroform	35	10.1039/c7pp00351j
CCCCCCn1c2ccc(cc2c2c1ccc(c2)C=C1C(=O)NC(=O)NC1=O)c1ccc(cc1)OC	chloroform	107	10.1039/c7pp00351j
CCCCCCN1c2ccccc2C(/C/1=C/C1=C([O-])/C(=C\C2=[N+](C)c3c(C2)cc(cc3)c2sc(c3c2OCC2(CO3)COc3c(OC2)c(sc3c2ccc3c(c2)CC(=[N+]3C)/C=C/2\C(=C(C2=O)/C=C/2\N(CCCCCC)c3c(C2(C)C)cccc3)[O-])/C=C(/	chloroform	-2	10.1002/cplu.201800450

SMILES	SOLVENT	SHIFT (nm)	DOI
C(=O)O)\C#N)/C=C(/C(=O)O)\C#N)/C1=O)(C)C			
CCCCCCN1c2ccccc2C(/C/1=C/C1=C([O-])/C(=C\C2=[N+](C)c3c(C2)cc(cc3)c2sc(c3c2OC[C@]2(CO3)COc3c(OC2)c(sc3c2ccc(cc2)N(c2ccc(cc2)OC)c2ccc(cc2)OC)/C=C(/C(=O)O)\C#N)/C=C(/C(=O)O)\C#N)/C1=O)(C)C	chloroform	15	10.1002/cplu.201800450
N#CC(=Cc1ccc(n1C)C=C1C=C(OC(=C1)c1ccccc1)c1ccccc1)C(=O)O	chloroform	51	10.1016/j.orgel.2014.09.003
N#CC(=Cc1ccc(n1C)C=C1C=C(OC(=C1)C(C)(C)C)C(C)(C)C)C(=O)O	chloroform	34	10.1016/j.orgel.2014.09.003
N#CC(=Cc1ccc(o1)C=C1C=C(OC(=C1)c1ccccc1)c1ccccc1)C(=O)O	chloroform	77	10.1016/j.orgel.2014.09.003
N#CC(=Cc1ccc(o1)C=C1C=C(OC(=C1)C(C)(C)C)C(C)(C)C)C(=O)O	chloroform	31	10.1016/j.orgel.2014.09.003
N#CC(=Cc1ncc(s1)C=C1C=C(OC(=C1)c1ccccc1)c1ccccc1)C(=O)O	chloroform	60	10.1016/j.orgel.2014.09.003
N#CC(=Cc1ncc(s1)C=C1C=C(OC(=C1)C(C)(C)C)C(C)(C)C)C(=O)O	chloroform	65	10.1016/j.orgel.2014.09.003
N#CC(=Cc1cnc(s1)C=C1C=C(OC(=C1)c1ccccc1)c1ccccc1)C(=O)O	chloroform	66	10.1016/j.orgel.2014.09.003
N#CC(=Cc1cnc(s1)C=C1C=C(OC(=C1)C(C)(C)C)C(C)(C)C)C(=O)O	chloroform	70	10.1016/j.orgel.2014.09.003
N#CC(=Cc1ccc(s1)C=C1C=C(OC(=C1)C(C)(C)C)C(C)(C)C)C(=O)O	chloroform	66	10.1021/ol203298r
CCCCCCc1c(C=C2C=C(OC(=C2)C(C)(C)C)C(C)(C)C)sc(c1CCCCCC)C=C(C(=O)O)C#N	chloroform	47	10.1021/ol203298r
CCCCCCc1c(sc(c1CCCCCC)c1ccc(cc1)N(c1ccccc1)c1ccccc1)C1=C/C(=C\2/C=C(OC2=O)c2sc(c(c2CCCCCC)CCCCCC)c2ccc(s2)C=C(C(=O)O)C#N)/C(=O)O1	chloroform	59	10.1021/acsomega.8b03560
CCCCCCSc1ccc(cc1)N(c1ccc(cc1)SCCCCCC)c1ccc(cc1)c1sc(c(c1CCCCCC)CCCCCC)C1=C/C(=C\2/C=C(OC2=O)c2sc(c(c2CCCCCC)CCCCCC)c2ccc(s2)C=C(C(=O)O)C#N)/C(=O)O1	chloroform	77	10.1021/acsomega.8b03560
CCCCCCSc1ccc(cc1)N(c1ccc(cc1)SCCCCCC)c1ccc(cc1)c1sc(c(c1CCCCCC)CCCCCC)C1=C/C(=C\2/C=C(OC2=O)c2sc(c(c2CCCCCC)CCCCCC)c2ccc(cc2)C(=O)O)/C(=O)O1	chloroform	93	10.1021/acsomega.8b03560
CCCCCCc1c(sc(c1CCCCCC)c1ccc(cc1)N(c1ccc(cc1)SCCCCCC)c1ccc(cc1)SCCCCCC)C1=C/	chloroform	116	10.1021/acsomega.8b03560

SMILES	SOLVENT	SHIFT (nm)	DOI
C(=C\2/ C=C(OC2=O)c2sc(c(c2CCCCCC)CCCCCC)C#C c2ccc(cc2)C(=O)O)/C(=O)O1			
CCCCCCN1C(=O)/C(=C/2\ C=C(N(C2=O)CCCCCC)c2sc(c(c2CCCCCC)CC CCCC)c2ccc(s2)C=C(C(=O)O)C#N)/ C=C1c1sc(c(c1CCCCCC)CCCCCC)c1ccc(cc1)N (c1ccccc1)c1ccccc1	chloroform	-4	10.1021/acsomega.8b03560
CCCCC(Cc1cc(sc1c1sc(c2c1nccn2)c1sc(cc1C C(CCCC)CC)C=C(C(=O)O)C#N)c1ccc(cc1)N(c 1ccccc1)c1ccccc1)CC	chloroform	16	10.1021/cm301520z
CCCCCCc1cc(sc1c1sc(c2c1nccn2)c1sc(cc1C CCCCC)C=C(C(=O)O)C#N)c1ccc(cc1)N(c1ccc cc1)c1ccccc1	chloroform	3	10.1021/cm301520z
CCCCCCOc1ccc(cc1)N(c1ccc(cc1)OCCCCCC) c1ccc(cc1)c1ccc(s1)c1sc(c2c1nccn2)c1ccc(s 1)C=C(C(=O)O)C#N	chloroform	20	10.1021/cm301520z
CCCCCCCCOc1ccc(cc1)N(c1ccc(cc1)OCCCCC CCC)c1ccc(cc1)c1ccc(s1)c1sc(c2c1nccn2)c1 ccc(s1)C=C(C(=O)O)C#N	chloroform	20	10.1021/cm301520z
CCCCCCCCOc1ccc(cc1)N(c1ccc(cc1)OCCCCC CCC)c1ccc(cc1)c1sc(c(c1)CCCCCC)c1sc(c2c1 nccn2)c1sc(cc1CCCCCC)C=C(C(=O)O)C#N	chloroform	4	10.1021/cm301520z
N#C/C(=C\ c1ccc(s1)c1ncc(c2c1nsn2)c1ccc(cc1)N(c1cc ccc1)c1ccccc1)/C(=O)O	dichloromethane	49	10.1016/ j.dyepig.2013.11.001
N#C/C(=C\ c1ccc(s1)c1ncc(c2c1nsn2)c1ccc(cc1)N(c1cc c2c(c1)C(C)(C)c1c2cccc1)c1ccc2c(c1)C(C) (C)c1c2cccc1)/C(=O)O	dichloromethane	31	10.1016/ j.dyepig.2013.11.001
CCCCCCOc1ccc(cc1)N(c1ccc(cc1)OCCCCCC) c1ccc(cc1)c1cnc(c2c1nsn2)c1ccc(s1)/C=C(/ C(=O)O)\C#N	dichloromethane	27	10.1016/ j.dyepig.2013.11.001
N#C/C(=C\ c1ccc(s1)c1ccc(c2c1nsn2)c1ccc(cc1)N1c2cc ccc2C=Cc2c1cccc2)/C(=O)O	dichloromethane	9	10.1016/j.tet.2014.02.074
N#C/C(=C\ c1ccc(cc1)c1ccc(c2c1nsn2)c1ccc(cc1)N1c2c cccc2C=Cc2c1cccc2)/C(=O)O	dichloromethane	3	10.1016/j.tet.2014.02.074
N#C/C(=C\ c1ccc(s1)c1ccc(c2c1nsn2)C#Cc1ccc(cc1)N1 c2ccccc2C=Cc2c1cccc2)/C(=O)O	dichloromethane	-3	10.1016/j.tet.2014.02.074
N#C/C(=C\ c1ccc(cc1)c1ccc(c2c1nsn2)C#Cc1ccc(cc1)N	dichloromethane	9	10.1016/j.tet.2014.02.074

SMILES	SOLVENT	SHIFT (nm)	DOI
1c2ccccc2C=Cc2c1cccc2)/C(=O)O			
N#C/C(=C\ c1ccc(s1)c1ccc(cc1)N1c2ccccc2C=Cc2c1cccc2)/C(=O)O	dichloromethane	52	10.1016/j.tet.2014.02.074
N#C/C(=C\ c1ccc(s1)c1ccc(cc1)N1c2ccccc2CCc2c1cccc2)/C(=O)O	dichloromethane	2	10.1016/ j.dyepig.2011.11.002
C1=CC=CC=2N(C3=C(C=CC21)C=CC=C3)C3=CC=C(C=C3)C3=CC=C(S3)C=C(C(=O)O)C#N	dichloromethane	52	10.1016/ j.dyepig.2011.11.002
N#C/C(=C/c1ccc(s1)/C=C/ c1ccc(cc1)N1c2ccccc2CCc2c1cccc2)/ C(=O)O	dichloromethane	1	10.1016/ j.dyepig.2011.11.002
N#C/C(=C/c1ccc(s1)/C=C/ c1ccc(cc1)N1c2ccccc2C=Cc2c1cccc2)/ C(=O)O	dichloromethane	59	10.1016/ j.dyepig.2011.11.002
CCCCCCC1(CCCCCC)c2cc(ccc2c2c1cc(cc2)/ C=C(/c1ccc(s1)c1ccc(s1)/C=C(/C(=O)O)\ C#N)\ C#N)n1c2ccc(cc2c2c1ccc(c2)c1ccc(s1)CCCCCC)c1ccc(s1)CCCCCC	dichloromethane	24	10.1016/ j.dyepig.2015.02.020
CCCCCCC1(CCCCCC)c2cc(ccc2c2c1cc(cc2)/ C=C(/c1ccc(s1)c1ccc(s1)/C=C(/C(=O)O)\ C#N)\ C#N)n1c2ccc(cc2c2c1ccc(c2)c1sc2c(c1)sc(c2)CCCCCC)c1sc2c(c1)sc(c2)CCCCCC	dichloromethane	32	10.1016/ j.dyepig.2015.02.020
CCCCCCC1(CCCCCC)c2cc(ccc2c2c1cc(cc2)/ C=C(/c1ccc(s1)c1ccc(s1)/C=C(/C(=O)O)\ C#N)\C#N)n1c2ccc(cc2c2c1ccc(c2)C(C)(C)C)C(C)(C)C	dichloromethane	32	10.1016/ j.dyepig.2015.02.020
CCCCC[C@H] (n1c2ccc(cc2c2c1c1ccccc1c(c2)c1ccc(s1)/ C=C(/C(=O)O)\C#N)OC)CC	dichloromethane	30	10.1021/am508400a
CCCCC[C@H] (n1c2ccc(cc2c2c1c1ccccc1c(c2)c1ccc(o1)/ C=C(/C(=O)O)\C#N)OC)CC	dichloromethane	10	10.1021/am508400a
CCCCCCCCc1cc(sc1c1ccc(s1)c1sc(cc1CCCCCCCC)/C=C(/C(=O)O)\ C#N)c1cc2c(c3c1cccc3)n(c1c2cc(OC)cc1) [C@H](CCCCC)CC	dichloromethane	16	10.1021/am508400a
CCCCCCn1c2ccc(cc2c2c1c1ccccc1C2(CCCCCC)CCCCCC)c1ccc(cc1)/C=C(/C(=O)O)\C#N	dichloromethane	-6	10.1016/ j.dyepig.2016.12.028
CCCCCCn1c2ccc(cc2c2c1c1ccccc1C2(CCCCCC)CCCCCC)c1ccc(cc1)/C=C/1\ SC(=S)N(C1=O)CC(=O)O	dichloromethane	-8	10.1016/ j.dyepig.2016.12.028

SMILES	SOLVENT	SHIFT (nm)	DOI
CCCCCCn1c2ccc(cc2c2c1c1ccccc1C2(CCCCCC)CCCCCC)c1ccc(cc1)/C=c/1\sc(=C(C#N)C#N)[nH]c1=O	dichloromethane	-14	10.1016/j.dyepig.2016.12.028
CCCCCCn1c2ccc(cc2c2c1c1ccccc1C2(CCCCCC)CCCCCC)c1ccc(s1)/C=c/1\sc(=C(C#N)C#N)[nH]c1=O	dichloromethane	-12	10.1016/j.dyepig.2016.12.028
CCCCN1c2ccc(cc2Sc2c1ccc(c2)n1c2ccc(cc2c2c1ccc(c2)C(C)(C)C)C(C)(C)C)/C=C/1\SC(=S)N(C1=O)CC(=O)O	dichloromethane	1	10.1016/j.tet.2016.12.019
CCCCCCN1c2ccc(cc2Sc2c1ccc(c2)n1c2ccc(cc2c2c1ccc(c2)C(C)(C)C)C(C)(C)C)/C=C/1\SC(=S)N(C1=O)CC(=O)O	dichloromethane	1	10.1016/j.tet.2016.12.019
CCCCN1c2ccc(cc2Sc2c1ccc(c2)/C=C/1\SC(=S)N(C1=O)CC(=O)O)n1c2ccc(cc2c2c1ccc(c2)n1c2ccc(cc2c2c1ccc(c2)C(C)(C)C)C(C)(C)C)n1c2ccc(cc2c2c1ccc(c2)C(C)(C)C)C(C)(C)C	dichloromethane	41	10.1016/j.tet.2016.12.019
CCCCCCN1c2ccc(cc2Sc2c1ccc(c2)/C=C/1\SC(=S)N(C1=O)CC(=O)O)n1c2ccc(cc2c2c1ccc(c2)n1c2ccc(cc2c2c1ccc(c2)C(C)(C)C)C(C)(C)C)n1c2ccc(cc2c2c1ccc(c2)C(C)(C)C)C(C)(C)C	dichloromethane	14	10.1016/j.tet.2016.12.019
CCCCn1c2ccc(cc2c2c1ccc(c2)/C=C(/C(=O)O)\C#N)/C=C(/C(=O)O)\C#N	dichloromethane	-19	10.1016/j.jphotochem.2014.09.007
CCCCn1c2ccc(cc2c2c1ccc(c2)N(c1ccc(cc1)OC)c1ccccc1)N(c1ccc(cc1)/C=C(/C(=O)O)\C#N)c1ccc(cc1)OC	dichloromethane	-19	10.1016/j.jphotochem.2014.09.007
CCCCn1c2ccc(cc2c2c1ccc(c2)N(c1ccc(cc1)/C=C(/C(=O)O)\C#N)c1ccc(cc1)OC)N(c1ccc(cc1)/C=C(/C(=O)O)\C#N)c1ccc(cc1)OC	dichloromethane	-16	10.1016/j.jphotochem.2014.09.007
CCCCCCCCCCCCn1c2ccc(cc2c2c1ccc(c2)c1ccc(s1)/C=C(/C(=O)O)\C#N)n1c2ccc(cc2c2c1ccc(c2)C(C)(C)C)C(C)(C)C	dichloromethane	20	10.1002/ejoc.201300373
CCCCCCCCCCCCn1c2ccc(cc2c2c1ccc(c2)n1c2ccc(cc2c2c1ccc(c2)C(C)(C)C)C(C)(C)C)c1ccc(s1)c1ccc(s1)/C=C(/C(=O)O)\C#N	dichloromethane	24	10.1002/ejoc.201300373
CCCCCCCCCCCCn1c2ccc(cc2c2c1ccc(c2)n1c2ccc(cc2c2c1ccc(c2)C(C)(C)C)C(C)(C)C)c1ccc(s1)c1ccc(s1)c1ccc(s1)/C=C(/C(=O)O)\C#N	dichloromethane	1	10.1002/ejoc.201300373
CCCCCCCCCCCCn1c2ccc(cc2c2c1ccc(c2)n1c2ccc(cc2c2c1ccc(c2)C(C)(C)C)C(C)	dichloromethane	-5	10.1002/ejoc.201300373

SMILES	SOLVENT	SHIFT (nm)	DOI
(C)C)c1ccc(s1)c1ccc(s1)c1ccc(cc1)/C=C(/C(=O)O)\C#N			
N#C/C(=C\c1ccc(s1)c1ccc(s1)c1ccc(s1)c1ccc(cc1)N(c1ccc(cc1)n1c2ccc(cc2c2c1ccc(c2)C(C)(C)C)C(C)(C)C)c1ccc(cc1)n1c2ccc(cc2c2c1ccc(c2)C(C)(C)C)C(C)(C)C)/C(=O)O	dichloromethane	40	10.1002/ejoc.201300373
CCCCn1c2ccc(cc2c2c1ccc(c2)/C=C(/C(=O)O)\C#N)c1ccc(cc1)OC	dichloromethane	18	10.1002/ejoc.201600353
CCCCn1c2ccc(cc2c2c1ccc(c2)c1ccc(cc1)OC)c1ccc(s1)/C=C(/C(=O)O)\C#N	dichloromethane	35	10.1002/ejoc.201600353
CCCCn1c2ccc(cc2c2c1ccc(c2)/C=C(/C(=O)O)\C#N)c1ccc(s1)c1ccc(cc1)OC	dichloromethane	24	10.1002/ejoc.201600353
CCCCn1c2ccc(cc2c2c1ccc(c2)c1ccc(s1)/C=C(/C(=O)O)\C#N)c1ccc(s1)c1ccc(cc1)OC	dichloromethane	15	10.1002/ejoc.201600353
CCCCn1c2ccc(cc2c2c1ccc(c2)/C=C(/C(=O)O)\C#N)c1sc(cc1c1ccc(cc1)OC)c1ccc(cc1)OC	dichloromethane	12	10.1002/ejoc.201600353
OC(=O)C(=C/C1=CC=C(C=C1)N1C2=CC=CC=C2C2=C1C=CC=C2)\C#N	dichloromethane	-38	10.1016/j.dyepig.2012.03.028
N#C/C(=C\c1ccc(cc1)N1c2ccccc2Sc2c1cccc2)/C(=O)O	dichloromethane	-41	10.1016/j.dyepig.2012.03.028
N#C/C(=C\c1ccc(cc1)N(c1ccccc1)c1ccccc1)/C(=O)O	dichloromethane	-22	10.1016/j.dyepig.2012.03.028
N#C/C(=C\c1ccc(cc1)c1cc2c3c(c1)c1ccccc1n3c1c2cccc1)/C(=O)O	dichloromethane	-54	10.1021/jp503455m
N#C/C(=C\c1ccc(cc1)c1ccc(cc1)c1cc2c3c(c1)c1ccccc1n3c1c2cccc1)/C(=O)O	dichloromethane	-118	10.1021/jp503455m
N#C/C(=C\c1ccc(s1)c1cc2c3c(c1)c1ccccc1n3c1c2cccc1)/C(=O)O	dichloromethane	-61	10.1021/jp503455m
N#C/C(=C\c1ccc(s1)c1ccc(cc1)c1cc2c3c(c1)c1ccccc1n3c1c2cccc1)/C(=O)O	dichloromethane	-99	10.1021/jp503455m
OC(=O)C(=C\C1=CC=C(S1)C1=CC=C(C=C1)N(C1=CC=CC=C1)C1=CC=CC=C1)\C#N	dichloromethane	-2	10.1021/jp503455m
CCCC[C@H](CN1c2cc(ccc2/C(=C/2\C(=O)N(c3c2ccc(c3)c2ccc(s2)/C=C(/C(=O)O)\C#N)C[C@@H](CCCC)CC)/	dichloromethane	19	10.1016/j.dyepig.2014.07.017

SMILES	SOLVENT	SHIFT (nm)	DOI
C1=O)c1ccc2c(c1)[C@@H]1CCC[C@@H]1N2c1ccc(cc1)C)CC			
CCCC[C@@H](CN1c2cc(ccc2/C(=C/2\C(=O)N(c3c2ccc(c3)c2ccc(o2)/C=C(/C(=O)O)\C#N)C[C@@H](CCCC)CC)/C1=O)c1ccc2c(c1)[C@@H]1CCC[C@@H]1N2c1ccc(cc1)C)CC	dichloromethane	13	10.1016/j.dyepig.2014.07.017
CCCC[C@H](CN1c2cc(ccc2/C(=C/2\C(=O)N(c3c2ccc(c3)c2ccc(cc2)/C=C(/C(=O)O)\C#N)C[C@@H](CCCC)CC)/C1=O)c1ccc2c(c1)[C@@H]1CCC[C@@H]1N2c1ccc(cc1)C)CC	dichloromethane	29	10.1016/j.dyepig.2014.07.017
CCCCCCCCN1c2cc(ccc2/C(=C\2/c3ccc(cc3N(C2=O)CCCCCCCC)c2ccc(s2)/C=C(/C(=O)O)\C#N)/C1=O)c1ccc(cc1)N(c1ccccc1)c1ccccc1	dichloromethane	21	10.1016/j.dyepig.2014.07.017
N#C/C(=C\c1ccc(s1)c1ccc(c2c1nc(c1ccccc1)c(n2)c1ccccc1)c1ccc2c(c1)[C@@H]1CCC[C@@H]1N2c1ccc(cc1)C)/C(=O)O	dichloromethane	39	10.1021/ja500280r
CCCCCCOc1cc(OCCCCCC)ccc1c1ccc(cc1)N1c2ccc(cc2[C@H]2[C@@H]1CCC2)c1ccc(c2c1nc(c1ccccc1)c(n2)c1ccccc1)c1ccc(s1)/C=C(/C(=O)O)\C#N	dichloromethane	20	10.1021/ja500280r
CCCCCCOc1cc(OCCCCCC)ccc1c1ccc(cc1)C(=Cc1ccc(cc1)N1c2ccc(cc2[C@H]2[C@@H]1CCC2)c1ccc(c2c1nc(c1ccccc1)c(n2)c1ccccc1)c1ccc(s1)/C=C(/C(=O)O)\C#N)c1ccc(cc1)c1ccc(cc1OCCCCCC)OCCCCCC	dichloromethane	26	10.1021/ja500280r
N#C/C(=C\c1ccc(s1)c1ccc2c(c1)[C@@H]1CCC[C@@H]1N2c1ccc(cc1)C)/C(=O)O	dichloromethane	41	10.1021/am3001049
CCCCCCCCN1C(=O)c2c(C1=O)c(ccc2c1ccc2c(c1)[C@H]1CCC[C@H]1N2c1ccc(cc1)C)c1ccc(s1)/C=C(/C(=O)O)\C#N	dichloromethane	53	10.1021/am3001049
CCCCCCCCn1nc2c(n1)c(ccc2c1ccc(s1)/C=C(/C(=O)O)\C#N)c1ccc2c(c1)[C@H]1CCC[C@H]1N2c1ccc(cc1)C	dichloromethane	25	10.1021/am3001049
N#C/C(=C\c1ccc(s1)c1sc(c2c1OCCO2)c1ccc2c(c1)[C@@H]1CCC[C@@H]1N2c1ccc(cc1)C)/C(=O)O	dichloromethane	32	10.1016/j.dyepig.2013.04.031

SMILES	SOLVENT	SHIFT (nm)	DOI
N#C/C(=C\c1ccc(s1)c1sc(c2c1OCCO2)c1ccc(cc1)N(c1ccccc1)c1ccccc1)/C(=O)O	dichloromethane	12	10.1016/j.dyepig.2013.04.031
CCCc1c(/C=C(/C(=O)O)\C#N)sc2c1sc(c2)c1ccc2c(c1)[C@@H]1CCC[C@@H]1N2c1ccc2c(c1)C(CCC)(CCC)c1c2cccc1	dichloromethane	48	10.1021/cm400196w
CCCCCCOc1ccc(cc1)N1[C@H]2CCC[C@H]2c2c1ccc(c2)c1cc2c(s1)c(c(s2)/C=C(/C(=O)O)\C#N)CCC	dichloromethane	72	10.1021/cm400196w
CCCc1c(/C=C(/C(=O)O)\C#N)sc2c1sc(c2)c1ccc2c(c1)[C@@H]1CCC[C@@H]1N2c1ccc(cc1)OC(C)(C)C	dichloromethane	38	10.1021/cm400196w
CCCc1c(/C=C(/C(=O)O)\C#N)sc2c1sc(c2)c1ccc2c(c1)[C@@H]1CCC[C@@H]1N2c1ccc2c(c1)C(CCC)(CCC)c1c2c2c(c3c1c1ccccc1C3(CCC)CCC)c1c(C2(CCC)CCC)cccc1	dichloromethane	52	10.1021/cm400196w
CCCCCCn1c2ccc(cc2c2c1cccc2)N1c2ccc(cc2[C@H]2[C@@H]1CCC2)c1ccc(c2c1nsn2)c1ccc(s1)/C=C(/C(=O)O)\C#N	dichloromethane	24	10.1021/acsami.5b08888
CCCCCCCCC[C@]1(CCCCCCCC)c2cc(sc2c2c1cc(s2)/C=C(/C(=O)O)\C#N)c1ccc(c2c1nsn2)c1ccc2c(c1)[C@H]1CCC[C@H]1N2c1ccc2c(c1)c1ccccc1n2CCCCCC	dichloromethane	24	10.1021/acsami.5b08888
CC1=CC=C(C=C1)N1C2CCCC2C2=CC(=CC=C12)C1=CC=C(C2=CC=C(S2)\C=C(\C#N)C(O)=O)C2=NSN=C12	dichloromethane	33	10.1021/acsami.5b08888
Cc1ccc(cc1)N1c2ccc(cc2[C@H]2[C@@H]1CCC2)c1ccc(c2c1nsn2)c1ccc(cc1)C(=O)O	dichloromethane	22	10.1039/C6TC03418G
Cc1ccc(cc1)N1c2ccc(cc2[C@H]2[C@@H]1CCC2)C#Cc1ccc(c2c1nsn2)c1ccc(cc1)C(=O)O	dichloromethane	29	10.1039/C6TC03418G
Cc1ccc(cc1)N1[C@H]2CCC[C@H]2c2c1ccc(c2)c1ccc(c2c1nsn2)C#Cc1ccc(cc1)C(=O)O	dichloromethane	34	10.1039/C6TC03418G
CCCCCCCCOc1cc(OCCCCCCCC)ccc1c1ccc(cc1)C(=Cc1ccc(cc1)N1c2ccc(cc2[C@H]2[C@@H]1CCC2)c1ncc(c2c1nc(c1ccccc1)c(n2)c1ccccc1)c1ccc(s1)/C=C(/C(=O)O)\C#N)c1ccc(cc1)c1ccc(cc1OCCCCCCCC)OCCCCCCCC	dichloromethane	17	10.1021/am507824h
CCCCCCCCOc1cc(OCCCCCCCC)ccc1c1ccc(cc	dichloromethane	9	10.1021/am507824h

SMILES	SOLVENT	SHIFT (nm)	DOI
1)N(c1ccc(cc1)c1ncc(c2c1nc(c1ccccc1)c(n2)c1ccccc1)c1ccc(s1)/C=C(/C(=O)O)\C#N)c1ccc(cc1)c1ccc(cc1OCCCCCCCC)OCCCCCCCC			
CCCCCCOc1cc(OCCCCCC)ccc1c1ccc2c(c1)Sc1c(N2CCCCCC)ccc(c1)/C=C(\C(=O)O)/C#N	dichloromethane	23	10.1039/c3cp52314d
CCCCCCN1c2ccc(cc2Sc2c1ccc(c2)/C=C/c1cc[n+](cc1)CC(=O)O)c1ccc(cc1OCCCCCC)OCCCCCC	dichloromethane	9	10.1039/c3cp52314d
CCCCCCOc1cc(OCCCCCC)ccc1c1ccc2c(c1)Sc1c(N2CCCCCC)ccc(c1)/C=C/C1=[N+](CCCCCC)c2c(C1(C)C)cc(cc2)C(=O)O	dichloromethane	59	10.1039/c3cp52314d
CCCCCCOc1ccc(cc1)c1ccc2c(c1)Sc1c(N2CC)ccc(c1)/C=C(/C(=O)O)\C#N	dichloromethane	20	10.1021/cm400800h
CCCCCCOc1ccc(cc1)c1ccc2c(c1)Sc1c(N2CCCCCC)ccc(c1)/C=C(/C(=O)O)\C#N	dichloromethane	26	10.1021/cm400800h
CCCCCCCCN1c2ccc(cc2Sc2c1ccc(c2)/C=C(/C(=O)O)\C#N)c1ccc(cc1)OCCCCCC	dichloromethane	10	10.1021/cm400800h
CCCCCCCCCCCCN1c2ccc(cc2Sc2c1ccc(c2)/C=C(/C(=O)O)\C#N)c1ccc(cc1)OCCCCCC	dichloromethane	13	10.1021/cm400800h
CCCCCCN1c2ccc(cc2Sc2c1ccc(c2)/C=C(/C(=O)O)\C#N)c1ccc2c(c1)c1ccccc1n2CCCCCC	dichloromethane	26	10.1016/j.jpowsour.2013.05.157
CCCCCCN1c2ccc(cc2Sc2c1ccc(c2)/C=C(/C(=O)O)\C#N)c1ccc2c(c1)c1ccccc1C2(CCCCCC)CCCCCC	dichloromethane	20	10.1016/j.jpowsour.2013.05.157
CCCCCCn1c2ccccc2c2c1cc(cc2)N1c2ccccc2Sc2c1ccc(c2)/C=C(/C(=O)O)\C#N	dichloromethane	133	10.1016/j.jpowsour.2013.05.157
CCCCCCC1(CCCCCC)c2cc(ccc2c2c1cccc2)N1c2ccccc2Sc2c1ccc(c2)/C=C(/C(=O)O)\C#N	dichloromethane	129	10.1016/j.jpowsour.2013.05.157
CCCCN1c2ccc(cc2Sc2c1cccc2)/C=C(/C(=O)O)\C#N	dichloromethane	27	10.1039/b707485a
CCCCN1c2ccc(cc2Sc2c1cccc2)/C=C/c1ccc(cc1)/C=C(/C(=O)O)\C#N	dichloromethane	41	10.1039/b707485a
CCCCN1c2ccc(cc2Sc2c1cccc2)/C=C\1/SC(=S)N(C1=O)CC(=O)O	dichloromethane	5	10.1039/b707485a
CCCCN1c2ccc(cc2Sc2c1cccc2)/C=C/c1ccc(cc1)/C=C\1/SC(=S)N(C1=O)CC(=O)O	dichloromethane	8	10.1039/b707485a
CCCCCCCCN1c2ccc(cc2Sc2c1ccc(c2)c1ccc(s1)/C=C(/C(=O)O)\C#N)c1ccc(cc1)N(c1ccc(cc1)OC)c1ccc(cc1)OC	dichloromethane	-16	10.1039/b918282a

SMILES	SOLVENT	SHIFT (nm)	DOI
CCCCCCCCN1c2ccc(cc2Sc2c1ccc(c2)c1ccc(s1)/C=C(/C(=O)O)\C#N)c1ccc(cc1)N(c1ccccc1)c1ccccc1	dichloromethane	-25	10.1039/b918282a
CCCCCCCCN1c2ccc(cc2Sc2c1ccc(c2)c1ccc(s1)/C=C(/C(=O)O)\C#N)c1ccc(cc1)C=C(c1ccccc1)c1ccccc1	dichloromethane	-31	10.1039/b918282a
CCCCCCN1c2ccc(cc2Sc2c1cccc2)c1ccc2c(c1)Sc1c(N2CCCCCC)ccc(c1)/C=C(/C(=O)O)\C#N	dichloromethane	29	10.1039/c2jm35556f
CCCCCCN1c2ccc(cc2Sc2c1ccc(c2)c1ccc2c(c1)Sc1c(N2CCCCCC)ccc(c1)/C=C(/C(=O)O)\C#N)c1ccc2c(c1)Sc1c(N2CCCCCC)cccc1	dichloromethane	32	10.1039/c2jm35556f
CCCCCCOc1ccc(cc1)N1c2ccccc2Sc2c1ccc(c2)/C=C(/C(=O)O)\C#N	dichloromethane	38	10.1039/c2jm35556f
CCCCCCOc1ccc(cc1)N1c2ccc(cc2Sc2c1cccc2)c1ccc2c(c1)Sc1c(N2c2ccc(cc2)OCCCCCC)ccc(c1)/C=C(/C(=O)O)\C#N	dichloromethane	34	10.1039/c2jm35556f
CCCCCCOc1ccc(cc1)N1c2ccc(cc2Sc2c1ccc(c2)c1ccc2c(c1)Sc1c(N2c2ccc(cc2)OCCCCCC)ccc(c1)/C=C(/C(=O)O)\C#N)c1ccc2c(c1)Sc1c(N2c2ccc(cc2)OCCCCCC)cccc1	dichloromethane	37	10.1039/c2jm35556f
CCCCCCCCCCCCN1c2ccccc2Oc2c1ccc(c2)/C=C(/C(=O)O)\C#N	dichloromethane	45	10.1039/b912746a
CCCCCCCCCCCCN1c2ccc(cc2Oc2c1ccc(c2)/C=C(/C(=O)O)\C#N)/C=C/c1ccc(cc1)N(c1ccccc1)c1ccccc1	dichloromethane	49	10.1039/b912746a
CCCCCCc1ccc(s1)c1ccc2c(c1)Sc1c(N2CCCCCC)ccc(c1)/C=C(/C(=O)O)\C#N	dichloromethane	5	10.1002/chem.201304897
CCCCCCc1sc(c2c1OCCO2)c1ccc2c(c1)Sc1c(N2CCCCCC)ccc(c1)/C=C(/C(=O)O)\C#N	dichloromethane	6	10.1002/chem.201304897
CCCCCCc1ccc(s1)c1ccc(s1)c1ccc2c(c1)Sc1c(N2CCCCCC)ccc(c1)/C=C(/C(=O)O)\C#N	dichloromethane	4	10.1002/chem.201304897
CCCCCCc1sc(c2c1OCCO2)c1ccc(s1)c1ccc2c(c1)Sc1c(N2CCCCCC)ccc(c1)/C=C(/C(=O)O)\C#N	dichloromethane	14	10.1002/chem.201304897
CCCCCCc1sc(c2c1OCCO2)c1sc(c2c1OCCO2)c1ccc2c(c1)Sc1c(N2CCCCCC)ccc(c1)/C=C(/C(=O)O)\C#N	dichloromethane	17	10.1002/chem.201304897
CCCCN1c2ccc(cc2Sc2c1cccc2)c1ccc(o1)/C=C(/C(=O)O)\C#N	dichloromethane	30	10.1021/ol2023517
CCCCN1c2ccc(cc2Sc2c1cccc2)c1ccc(s1)/C=C(/C(=O)O)\C#N	dichloromethane	32	10.1021/ol2023517
CCCCN1c2ccc(cc2Sc2c1cccc2)c1sc(c2c1OCC	dichloromethane	40	10.1021/ol2023517

SMILES	SOLVENT	SHIFT (nm)	DOI
O2)/C=C(/C(=O)O)\C#N			
C(CCC)N1C2=CC=CC=C2SC=2C=C(C=CC12)C=C(C(=O)O)C#N	dichloromethane	34	10.1016/j.dyepig.2012.12.007
C(#N)C(C(=O)O)=CC=1C=CC=2N(C3=CC=CC=C3SC2C1)C1=CC=C(C=C1)OC	dichloromethane	38	10.1016/j.dyepig.2012.12.007
CCCCN1c2cc(SCC)c(cc2Sc2c1cccc2)/C=C(/C(=O)O)\C#N	dichloromethane	44	10.1016/j.dyepig.2012.12.007
CCSc1cc2c(cc1/C=C(/C(=O)O)\C#N)Sc1c(N2c2ccc(cc2)OC)cccc1	dichloromethane	38	10.1016/j.dyepig.2012.12.007
CCCCCCc1cc(sc1c1ccc(cc1)N(c1ccccc1)c1ccccc1)c1cc[n+](cc1)CC(=O)O	dichloromethane	14	10.1021/jp311378b
CCCCCCc1cc(sc1c1ccc(cc1)N(c1ccc(cc1)OC)c1ccc(cc1)OC)c1cc[n+](cc1)CC(=O)O	dichloromethane	11	10.1021/jp311378b
CCCCCCc1cc(sc1c1ccc(cc1)N(c1ccc(cc1)OCCCCCC)c1ccc(cc1)OCCCCCC)c1cc[n+](cc1)CC(=O)O	dichloromethane	20	10.1021/jp311378b
OC(=O)C[n+]1ccc(cc1)/C=C/c1ccc(cc1)N(c1ccccc1)c1ccccc1	dichloromethane	23	10.1021/jp311378b
COc1ccc(cc1)N(c1ccc(cc1)OC)c1ccc(cc1)/C=C/c1cc[n+](cc1)CC(=O)O	dichloromethane	10	10.1021/jp311378b
CCCCCCc1cc(sc1c1ccc2c(c1)Sc1c(N2CCCCCC)cccc1)c1cc[n+](cc1)C(=O)O	dichloromethane	17	10.1021/jp311378b
CCCCCCN1c2ccc(cc2Sc2c1ccc(c2)c1sc(cc1CCCCCC)c1cc[n+](cc1)C(=O)O)c1ccc(cc1OCCCCCC)OCCCCCC	dichloromethane	20	10.1021/jp311378b
CCCCCCOc1cc(OCCCCCC)ccc1c1ccc(cc1)N(c1ccc(cc1)c1ccc(cc1OCCCCCC)OCCCCCC)c1ccc(cc1)/C=c/1\s/c(=C(/C(=O)O)\C#N)/nc1c1ccccc1	dichloromethane	7	10.1021/am403210v
CCCCCCN1c2ccc(cc2Sc2c1ccc(c2)/C=c/1\s/c(=C(/C(=O)O)\C#N)/nc1c1ccccc1)c1ccc(cc1OCCCCCC)OCCCCCC	dichloromethane	5	10.1021/am403210v
CCCCOc1ccc(cc1)N(c1ccc(cc1)OCCCC)c1ccc(cc1)c1ccc2c(c1)C1(OCCO1)C1(c3c2ccc(c3)c2ccc(s2)/C=C(/C(=O)O)\C#N)OCCO1	dichloromethane	20	10.1021/jp400011w
CCCCOc1ccc(cc1)N(c1ccc(cc1)OCCCC)c1ccc(cc1)c1ccc(s1)c1ccc2c(c1)C1(OCCO1)C1(c3c2ccc(c3)c2ccc(s2)/C=C(/C(=O)O)\C#N)OCCO1	dichloromethane	20	10.1021/jp400011w
CCCCN1c2ccc(cc2Sc2c1cccc2)c1ccc2c(c1)C1(OCCO1)C1(c3c2ccc(c3)c2ccc(s2)/C=C(/C(=O)O)\C#N)OCCO1	dichloromethane	29	10.1021/jp400011w
CCCCCCn1c(c2ccc(cc2)c2ccc(cc2)/C=C(/	dichloromethane	-6	10.1016/

SMILES	SOLVENT	SHIFT (nm)	DOI
C(=O)O)\C#N)c2c(c1=O)c(n(c2=O)CCCCCC)c1ccc2c(c1)Sc1c(N2CCCCCC)cccc1			j.jpowsour.2014.08.030
CCCCCCn1c(c2ccc(cc2)c2ccc3c(c2)Sc2c(N3CCCCCC)cccc2)c2c(c1=O)c(n(c2=O)CCCCCC)c1ccc(cc1)/C=C(/C(=O)O)\C#N	dichloromethane	-6	10.1016/j.jpowsour.2014.08.030
CCCCCCn1c(c2ccc(cc2)c2ccc3c(c2)Sc2c(N3CCCCCC)cccc2)c2c(c1=O)c(n(c2=O)CCCCCC)c1ccc(cc1)c1ccc(cc1)/C=C(/C(=O)O)\C#N	dichloromethane	-21	10.1016/j.jpowsour.2014.08.030
CCCCCCn1c(c2ccc(cc2)N(c2ccc(cc2)OCCCCCC)c2ccc(cc2)OCCCCCC)c2c(c1=O)c(n(c2=O)CCCCCC)c1ccc(cc1)c1ccc(cc1)/C=C(/C(=O)O)\C#N	dichloromethane	-4	10.1016/j.jpowsour.2014.08.030
CCCCCCn1c(c2ccc(cc2)c2ccc(cc2)N(c2ccc(cc2)OCCCCCC)c2ccc(cc2)OCCCCCC)c2c(c1=O)c(n(c2=O)CCCCCC)c1ccc(cc1)/C=C(/C(=O)O)\C#N	dichloromethane	-4	10.1016/j.jpowsour.2014.08.030
CCCCCCn1c(=O)c2c(c1c1ccc(cc1)c1ccc(cc1)N(c1ccc(cc1)OCCCCCC)c1ccc(cc1)OCCCCCC)c(=O)n(c2c1ccc(cc1)c1ccc(cc1)/C=C(/C(=O)O)\C#N)CCCCCC	dichloromethane	-16	10.1016/j.jpowsour.2014.08.030
CCCCN1c2ccc(cc2Sc2c1ccc(c2)c1ccc(cc1OCCCC)OCCCC)c1ccc(s1)c1ccc(s1)/C=C(/C(=O)O)\C#N	dichloromethane	19	10.1039/c4ra01858c
CCCCN1c2ccc(cc2Sc2c1ccc(c2)c1ccc(cc1OCCCC)OCCCC)c1ccc(c2c1nsn2)c1ccc(s1)/C=C(\C(=O)O)/C#N	dichloromethane	33	10.1039/c4ra01858c
N#C/C(=C\c1ccc(s1)c1ccc2c(c1)C(CC)(CC)c1c2ccc(c1)N1c2ccccc2Sc2c1cccc2)/C(=O)O	dichloromethane	-16	10.1021/am506149q
N#C/C(=C\c1ccc(s1)c1ccc(s1)c1ccc2c(c1)C(CC)(CC)c1c2ccc(c1)N1c2ccccc2Sc2c1cccc2)/C(=O)O	dichloromethane	-20	10.1021/am506149q
N#C/C(=C\c1ccc(cc1)c1ccc2c(c1)C(CC)(CC)c1c2ccc(c1)N1c2ccccc2Sc2c1cccc2)/C(=O)O	dichloromethane	-32	10.1021/am506149q
CCCCN1c2ccc(cc2Sc2c1cccc2)c1ccc2c(c1)C(CC)(CC)c1c2ccc(c1)c1ccc(s1)/C=C(/C(=O)O)\C#N	dichloromethane	-73	10.1021/am506149q
CCCCN1c2ccc(cc2Sc2c1cccc2)c1ccc2c(c1)C(CC)(CC)c1c2ccc(c1)c1ccc(s1)c1ccc(s1)/C=C(/C(=O)O)\C#N	dichloromethane	-17	10.1021/am506149q
CCCCCCCCN1c2ccc(cc2Oc2c1ccc(c2)/C=C(/	dichloromethane	50	10.1002/chem.201003730

SMILES	SOLVENT	SHIFT (nm)	DOI
C(=O)O)\C#N)c1ccc(cc1OCCCC)OCCCC			
CCCCCCOc1ccc(cc1)N1c2ccc(cc2Oc2c1ccc(c2)/C=C(/C(=O)O)\C#N)c1ccc(cc1OCCCC)OCCCC	dichloromethane	45	10.1002/chem.201003730
CCCCCCCCN1c2ccc(cc2Oc2c1cccc2)c1ccc(o1)/C=C(/C(=O)O)\C#N	dichloromethane	70	10.1002/chem.201003730
CCCCCCOc1ccc(cc1)N1c2ccccc2Oc2c1ccc(c2)/C=C(/C(=O)O)\C#N	dichloromethane	35	10.1002/chem.201003730
CCCCCCCCN1c2ccccc2Oc2c1ccc(c2)/C=C(/C(=O)O)\C#N	dichloromethane	43	10.1002/chem.201003730
CCCCN1c2ccc(cc2Sc2c1cccc2)/C=C/c1ccc(s1)/C=C(/C(=O)O)\C#N	dichloromethane	40	10.1016/j.dyepig.2009.06.014
C1(=CC=CC=C1)N(C1=CC=C(C=CC2=CC=C(S2)C=C(C(=O)O)C#N)C=C1)C1=CC=CC=C1	dichloromethane	60	10.1016/j.dyepig.2009.06.014
N#CC(=Cc1ccc(s1)C=Cc1ccc2c(c1)C(C)CC(N2C)(C)C)C(=O)O	dichloromethane	65	10.1016/j.dyepig.2009.06.014
CCCCCCCCN1c2ccc(cc2Sc2c1ccc(c2)n1c2ccc(cc2c2c1ccc(c2)C(C)(C)C)C(C)(C)C)c1ccc(cc1)/C=C(/C(=O)O)\C#N	dichloromethane	9	10.1039/c6ra06220b
CCCCCCCCN1c2ccc(cc2Sc2c1ccc(c2)n1c2ccc(cc2c2c1ccc(c2)C(C)(C)C)C(C)(C)C)c1ccc(s1)c1ccc(cc1)/C=C(/C(=O)O)\C#N	dichloromethane	1	10.1039/c6ra06220b
CCCCCCCCN1c2ccc(cc2Sc2c1ccc(c2)n1c2ccc(cc2c2c1ccc(c2)C(C)(C)C)C(C)(C)C)c1ccc(s1)c1ccc(s1)c1ccc(cc1)/C=C(/C(=O)O)\C#N	dichloromethane	7	10.1039/c6ra06220b
C(#N)C(C(=O)O)=CC=1C=CC=2N(C3=CC=CC=C3SC2C1)CCCCCC	dichloromethane	-15	10.1007/s13233-012-0017-2
C(#N)C(C(=O)O)=CC1=CC=C(S1)C=1SC(=CC1)C=CC=1C=CC=2N(C3=CC=CC=C3SC2C1)CCCCCC	dichloromethane	10	10.1007/s13233-012-0017-2
C(#N)C(C(=O)O)=CC=1SC(=CC1)C1=CC=C(C=C1)N(C1=CC=CC=C1)C1=CC=C(C=C1)C=CC=1C=CC=2N(C3=CC=CC=C3SC2C1)CCCCCC	dichloromethane	15	10.1007/s13233-012-0017-2
CCCCCC1C2C(C)=C(C(C)=[N+]2[B-](F)(F)N2C(C)=C(\C=C(/C#N)C(O)=O)C(C)=C12)C1=CC2=C(C=C1)N(CCC)C1=CC=C(C=C1S2)C1=C(C)C2C(CCCCC)C3=C(C)C(\C=C(/C#N)C(O)=O)=C(C)N3[B-](F)(F)[N+]2=C1C	dichloromethane	6	10.1016/j.dyepig.2015.02.001
CCCCCC1C2C(C)=C(C(C)=[N+]2[B-](F)(F)N2C(C)=C(C(C)=C12)C1=CC=C(O1)\C=C(/C#N)C(O)=O)C1=CC2=C(C=C1)N(CCC)C1=CC	dichloromethane	7	10.1016/j.dyepig.2015.02.001

SMILES	SOLVENT	SHIFT (nm)	DOI
=C(C=C1S2)C1=C(C)C2C(CCCCC)C3=C(C)C(=C(C)N3[B-](F)(F)[N+]2=C1C)C1=CC=C(O1)\C=C(\C#N)C(O)=O			
CCCCCC1C2C(C)=C(C(C)=[N+]2[B-](F)(F)N2C(C)=C(\C=C(\C#N)C(O)=O)C(C)=C12)C1=CC=C2N(CCC)C3=C(SC2=C1)C=CC=C3	dichloromethane	1	10.1016/j.dyepig.2015.02.001
N#C/C(=C/c1ccc2c(c1)Sc1c(N2c2ccc3c(c2)c2ccccc2n3CC)cccc1)/C(=O)O	dichloromethane	31	10.1039/c6cp05722e
CCCCn1c2ccccc2c2c1ccc(c2)N1c2ccccc2Sc2c1ccc(c2)/C=C(/C(=O)O)\C#N	dichloromethane	36	10.1039/c6cp05722e
CCCCCCCCn1c2ccccc2c2c1ccc(c2)N1c2ccccc2Sc2c1ccc(c2)/C=C(/C(=O)O)\C#N	dichloromethane	39	10.1039/c6cp05722e
CCCCn1c2ccc(cc2c2c1cccc2)N1c2ccc(cc2Sc2c1cccc2)/C=C/1\SC(=S)N(C1=O)CC(=O)O	dichloromethane	41	10.1039/c6cp05722e
CCCCCCn1c2ccccc2c2c1ccc(c2)N1c2ccccc2Sc2c1ccc(c2)/C=C/1\SC(=S)N(C1=O)CC(=O)O	dichloromethane	38	10.1039/c6cp05722e
CCCCCCCCn1c2ccccc2c2c1ccc(c2)N1c2ccccc2Sc2c1ccc(c2)/C=C/1\SC(=S)N(C1=O)CC(=O)O	dichloromethane	39	10.1039/c6cp05722e
CCCCCCN1c2ccc(cc2Sc2c1cccc2)/C=C(\C(=O)O)/C#N	dichloromethane	18	10.1016/j.electacta.2016.05.175
CCCCCCN1c2ccc(cc2Sc2c1ccc(c2)/C=C/1\SC(=C(S1)SC)SC)c1ccc(cc1)/C=C(/C(=O)O)\C#N	dichloromethane	9	10.1016/j.electacta.2016.05.175
CCCCCCN1c2ccc(cc2Sc2c1ccc(c2)/C=C(/C(=O)O)\C#N)/C=C/1\SC(=C(S1)SC)SC	dichloromethane	24	10.1016/j.electacta.2016.05.175
CCCCCCOc1ccc(cc1)N1c2ccc(cc2Sc2c1cccc2)/C=C/[C@@H]1C/C(=C(/C(=O)O)\C#N)/CC(C1)(C)C	dichloromethane	55	10.1039/c5ra17898c
N#C/C(=C/1\C[C@@H](/C=C/c2ccc(cc2)N(c2ccccc2)c2ccccc2)CC(C1)(C)C)/C(=O)O	dichloromethane	62	10.1039/c5ra17898c
N#C/C(=C/1\C[C@@H](/C=C/c2ccc(cc2)N(c2cccc3c2cccc3)c2ccccc2)CC(C1)(C)C)/C(=O)O	dichloromethane	71	10.1039/c5ra17898c
CCCCCCOc1ccc(cc1)N(c1ccc(cc1)OCCCCCC)c1ccc(cc1)/C=C/[C@@H]1C/C(=C(/C(=O)O)\C#N)/CC(C1)(C)C	dichloromethane	70	10.1039/c5ra17898c
CCCCCCn1c2ccc(cc2c2c1cccc2)c1cc(sc1c1sc(c(c1)c1ccc2c(c1)c1ccccc1n2CCCCCC)c1ccc2c(c1)c1ccccc1n2CCCCCC)/C=C(/C(=O)O)\	dichloromethane	14	10.1016/j.jphotochem.2013.12.019

SMILES	SOLVENT	SHIFT (nm)	DOI
C#N			
CCCCCCN1c2ccc(cc2Sc2c1cccc2)c1cc(sc1c1sc(c(c1)c1ccc2c(c1)Sc1c(N2CCCCCC)cccc1)c1ccc2c(c1)Sc1c(N2CCCCCC)cccc1)/C=C(\C(=O)O)/C#N	dichloromethane	62	10.1016/j.jphotochem.2013.12.019
N#C/C(=C\c1cc(c(s1)c1sc(c(c1)c1ccc(cc1)N(c1ccccc1)c1ccccc1)c1ccc(cc1)N(c1ccccc1)c1ccccc1)c1ccc(cc1)N(c1ccccc1)c1ccccc1)/C(=O)O	dichloromethane	66	10.1016/j.jphotochem.2013.12.019
CCCCC1(CCCC)c2cc(ccc2c2c1cc(cc2)c1ccc(s1)/C=C(/C(=O)O)\C#N)N1c2ccccc2Sc2c1ccc(c2)C=C1c2ccccc2c2c1cccc2	dichloromethane	-43	10.1007/s10854-016-5146-5
CCCCC1(CCCC)c2cc(ccc2c2c1cc(cc2)c1ccc(s1)c1ccc(s1)/C=C(/C(=O)O)\C#N)N1c2ccccc2Sc2c1ccc(c2)C=C1c2ccccc2c2c1cccc2	dichloromethane	-67	10.1007/s10854-016-5146-5
N#C/C(=C\c1ccc(s1)c1ccc2c(c1)C(CC)(CC)c1c2ccc(c1)N(c1ccccc1)c1ccccc1)/C(=O)O	dichloromethane	-2	10.1007/s10854-016-5146-5
N#C/C(=C\c1ccc(s1)c1ccc(s1)c1ccc2c(c1)C(CC)(CC)c1c2ccc(c1)N(c1ccccc1)c1ccccc1)/C(=O)O	dichloromethane	-18	10.1007/s10854-016-5146-5
N#C/C(=C\c1ccc(s1)c1ccc2c(c1)C(CC)(CC)c1c2ccc(c1)N(c1ccc(cc1)C=C1c2ccccc2c2c1cccc2)c1ccccc1)/C(=O)O	dichloromethane	-2	10.1007/s10854-016-5146-5
N#C/C(=C/c1ccc(s1)c1ccc(s1)c1ccc2c(c1)C(CC)(CC)c1c2ccc(c1)N(c1ccc(cc1)C=C1c2ccccc2c2c1cccc2)c1ccccc1)/C(=O)O	dichloromethane	-30	10.1007/s10854-016-5146-5
N#C/C(=C\c1ccc(s1)c1ccc2c(c1)C(CC)(CC)c1c2ccc(c1)N(c1ccc2c(c1)C(CC)(CC)c1c2cccc1)c1ccc2c(c1)C(CC)(CC)c1c2cccc1)/C(=O)O	dichloromethane	41	10.1016/j.dyepig.2014.07.036
N#C/C(=C\c1ccc(s1)c1ccc(s1)c1ccc2c(c1)C(CC)(CC)c1c2ccc(c1)N(c1ccc2c(c1)C(CC)(CC)c1c2cccc1)c1ccc2c(c1)C(CC)(CC)c1c2cccc1)/C(=O)O	dichloromethane	21	10.1016/j.dyepig.2014.07.036
N#C/C(=C\c1ccc(s1)c1ccc(s1)c1ccc2c(c1)C(CC)(CC)c1c2ccc(c1)N(c1ccc(cc1)C=C1c2ccccc2c2c1cccc2)c1ccccc1)/C(=O)O	dichloromethane	-30	10.1039/C3TA15456D

SMILES	SOLVENT	SHIFT (nm)	DOI
N#C/C(=C\c1ccc(s1)c1ccc2c(c1)C(CC)(CC)c1c2ccc(c1)N(c1ccc(cc1)C=C1c2ccccc2c2c1cccc2)c1ccc(cc1)C=C1c2ccccc2c2c1cccc2)/C(=O)O	dichloromethane	-10	10.1039/C3TA15456D
N#C/C(=C\c1ccc(s1)c1ccc(s1)c1ccc2c(c1)C(CC)(CC)c1c2ccc(c1)N(c1ccc(cc1)C=C1c2ccccc2c2c1cccc2)c1ccc(cc1)C=C1c2ccccc2c2c1cccc2)/C(=O)O	dichloromethane	-14	10.1039/C3TA15456D
N#CC(=Cc1ccc(cc1)N(c1ccc2c(c1)C(CC)(CC)c1c2ccc(c1)c1ccc(s1)/C=C(/C(=O)O)\C#N)c1ccccc1)C#N	dichloromethane	-2	10.1039/C3TA15456D
CCCCN1c2ccc(cc2Sc2c1ccc(c2)/C=C(/C(=O)O)\C#N)n1c2ccc(cc2c2c1ccc(c2)C(C)(C)C)C(C)(C)C	dichloromethane	30	10.1016/j.electacta.2017.06.151
CCCCCCN1c2ccc(cc2Sc2c1ccc(c2)/C=C(/C(=O)O)\C#N)n1c2ccc(cc2c2c1ccc(c2)C(C)(C)C)C(C)(C)C	dichloromethane	20	10.1016/j.electacta.2017.06.151
CCCCN1c2ccc(cc2Sc2c1ccc(c2)/C=C(/C(=O)O)\C#N)n1c2ccc(cc2c2c1ccc(c2)n1c2ccc(cc2c2c1ccc(c2)C(C)(C)C)C(C)(C)C)n1c2ccc(cc2c2c1ccc(c2)C(C)(C)C)C(C)(C)C	dichloromethane	22	10.1016/j.electacta.2017.06.151
CCCCCCN1c2ccc(cc2Sc2c1ccc(c2)/C=C(/C(=O)O)\C#N)n1c2ccc(cc2c2c1ccc(c2)n1c2ccc(cc2c2c1ccc(c2)C(C)(C)C)C(C)(C)C)n1c2ccc(cc2c2c1ccc(c2)C(C)(C)C)C(C)(C)C	dichloromethane	4	10.1016/j.electacta.2017.06.151
CCCCCCn1c2ccc(cc2c2c1c1ccccc1C2(CCCCCC)CCCCCC)C#Cc1ccc(cc1)/C=C(\C(=O)O)/C#N	dichloromethane	-1	10.1016/j.dyepig.2017.02.011
CCCCCCn1c2ccc(cc2c2c1c1ccccc1C2(CCCCCC)CCCCCC)C#Cc1ccc(s1)/C=C(/C(=O)O)\C#N	dichloromethane	-2	10.1016/j.dyepig.2017.02.011
CCCCCCn1c2ccc(cc2c2c1c1ccccc1C2(CCCCCC)CCCCCC)C#Cc1ccc(c2c1nsn2)c1ccc(cc1)C(=O)O	dichloromethane	6	10.1016/j.dyepig.2017.02.011
CCCC[C@H](Cn1c2cc(ccc2c2c1nc1c(ccc(c1n2)c1ccc(cc1)N(c1ccccc1)c1ccccc1)c1ccc(cc1)N(c1ccccc1)c1ccccc1)c1ccc(s1)/C=C(/C(=O)O)\C#N)CC	dichloromethane	16	10.1016/j.electacta.2017.02.166
CCCC[C@H](Cn1c2cc(ccc2c2c1nc1c(ccc(c1n2)c1ccc(cc1	dichloromethane	18	10.1016/j.electacta.2017.02.166

SMILES	SOLVENT	SHIFT (nm)	DOI
)N(c1ccccc1)c1ccccc1)c1ccc(cc1)N(c1ccccc1)c1ccccc1)c1ccc(o1)/C=C(/C(=O)O)\C#N)CC			
CCCC[C@H](Cn1c2nc3c(ccc(c3nc2c2c1cc(cc2)c1ccc(s1)/C=c/1\sc(=C(C#N)C#N)[nH]c1=O)c1ccc(cc1)N(c1ccccc1)c1ccccc1)c1ccc(cc1)N(c1ccccc1)c1ccccc1)CC	dichloromethane	2	10.1016/j.electacta.2017.02.166
CCCC[C@H](Cn1c2nc3c(ccc(c3nc2c2c1cc(cc2)c1ccc(o1)/C=c/1\sc(=C(C#N)C#N)[nH]c1=O)c1ccc(cc1)N(c1ccccc1)c1ccccc1)c1ccc(cc1)N(c1ccccc1)c1ccccc1)CC	dichloromethane	4	10.1016/j.electacta.2017.02.166
CCCCCCn1c2ccc(cc2c2c1cccc2)N(c1ccc(c(c1)C(=O)OC)c1cc2c(s1)c1c(n2CCCCCC)cc(s1)/C=C(/C(=O)O)\C#N)c1ccc(cc1)c1ccc2c(c1)C(CCCCCC)(CCCCCC)c1c2c2c(c3c1c1ccccc1C3(CCCCCC)CCCCCC)c1c(C2(CCCCCC)CCCCCC)cccc1	dichloromethane	17	10.1021/acssuschemeng.6b00706
CCCCCCn1c2ccc(cc2c2c1cccc2)N(c1ccc(cc1)c1cc2c(s1)c1c(n2CCCCCC)cc(s1)/C=C(/C(=O)O)\C#N)c1ccc(cc1)c1ccc2c(c1)C(CCCCCC)(CCCCCC)c1c2c2c(c3c1c1ccccc1C3(CCCCCC)CCCCCC)c1c(C2(CCCCCC)CCCCCC)cccc1	dichloromethane	40	10.1021/acssuschemeng.6b00706
CCCCCCOc1ccc(cc1)N(c1ccc(cc1)c1cc2c(s1)c1c(n2CCCCCC)cc(s1)/C=C(/C(=O)O)\C#N)c1ccc(cc1)c1ccc2c(c1)C(CCCCCC)(CCCCCC)c1c2c2c(c3c1c1ccccc1C3(CCCCCC)CCCCCC)c1c(C2(CCCCCC)CCCCCC)cccc1	dichloromethane	49	10.1021/acssuschemeng.6b00706
CCCCCCCCc1ccc(s1)c1nc2c(ccc(c2nc1c1ccc(s1)CCCCCCCC)c1ccc(cc1)/C=C(/C(=O)O)\C#N)c1cc(c(s1)c1ccc(cc1)N(c1ccccc1)c1ccccc1)CCCCCCCC	dichloromethane	-5	10.1016/j.dyepig.2017.07.022
CCCCCCCCc1ccc(s1)c1nc2c3cc(sc3c3c(c2nc1c1ccc(s1)CCCCCCCC)cc(s3)c1ccc(cc1)/C=C(/C(=O)O)\C#N)c1cc(c(s1)c1ccc(cc1)N(c1ccccc1)c1ccccc1)CCCCCCCC	dichloromethane	4	10.1016/j.dyepig.2017.07.022
CCCCCCCCc1ccc(s1)c1nc2c(sc(c2nc1c1ccc(s1)CCCCCCCC)c1ccc(cc1)/C=C(/C(=O)O)\C#N)c1cc(c(s1)c1ccc(cc1)N(c1ccccc1)c1ccccc1)CCCCCCCC	dichloromethane	3	10.1016/j.dyepig.2017.07.022
COc1ccc(cc1)N1c2ccc(cc2Oc2c1cccc2)c1ccc(o1)/C=C(/C(=O)O)\C#N	dichloromethane	35	10.1016/j.orgel.2017.01.035

SMILES	SOLVENT	SHIFT (nm)	DOI
CCCCCCCn1c2cc(/C=C/c3ccc(cc3)N(c3ccccc3)c3ccccc3)ccc2c2c1ccc(c2)/C=C(/C(=O)O)\C#N	dichloromethane	-2	10.1016/j.dyepig.2017.08.022
CCCCCCCn1c2ccc(cc2c2c1cc(/C=C/c1ccc(cc1)N(c1ccc(cc1)/C=C/c1ccc3c(c1)n(CCCCCCC)c1c3cc(cc1)/C=C(/C(=O)O)\C#N)c1ccccc1)cc2)/C=C(/C(=O)O)\C#N	dichloromethane	-23	10.1016/j.dyepig.2017.08.022
CCCCCCCn1c2cc(/C=C/c3ccc(cc3)N(c3ccc(cc3)/C=C/c3ccc4c(c3)n(CCCCCCC)c3c4cc(cc3)/C=C(/C(=O)O)\C#N)c3ccc(cc3)/C=C/c3ccc4c(c3)n(CCCCCCC)c3c4cc(cc3)/C=C(/C(=O)O)\C#N)ccc2c2c1ccc(c2)/C=C(/C(=O)O)\C#N	dichloromethane	-38	10.1016/j.dyepig.2017.08.022
CCCCCCOC1=CC=C(N2C3=C(SC(\C=C(/C#N)C(O)=O)=C3)C3=C2C=C(S3)C2=C3C(C)=CC(C)=[N+]3[B-](F)(F)N3C(C)=CC(C)=C23)C(OCCCCCC)=C1	dichloromethane	3	10.1021/acs.jpcc.6b07356
CCCCCCOC1=CC=C(N2C3=C(SC(\C=C(/C#N)C(O)=O)=C3)C3=C2C=C(S3)C2=CC3=C(S2)C2=C(C=C(S2)C2=C4C(C)=CC(C)=[N+]4[B-](F)(F)N4C(C)=CC(C)=C24)N3C2=CC(OCCCCCC)=CC(OCCCCCC)=C2)C(OCCCCCC)=C1	dichloromethane	7	10.1021/acs.jpcc.6b07356
CCCCCCOc1cc(OCCCCCC)ccc1n1c2cc(sc2c2c1cc(s2)/C=C(/C(=O)O)\C#N)c1ccc(cc1)N(c1ccccc1)c1ccccc1	dichloromethane	30	10.1021/acs.jpcc.6b07356
CCCCCCOc1cc(OCCCCCC)ccc1n1c2cc(sc2c2c1cc(s2)c1sc2c(c1)n(c1c2sc(c1)c1ccc(cc1)N(c1ccccc1)c1ccccc1)c1cc(OCCCCCC)cc(c1)OCCCCCC)/C=C(/C(=O)O)\C#N	dichloromethane	38	10.1021/acs.jpcc.6b07356
CCCCCCOC1=CC=C(N2C3=C(SC(\C=C(/C#N)C(O)=O)=C3)C3=C2C=C(S3)C2=CC=C(C=C2)C2=C3C(C)=CC(C)=[N+]3[B-](F)(F)N3C(C)=CC(C)=C23)C(OCCCCCC)=C1	dichloromethane	5	10.1021/acs.jpcc.6b07356
CCCCCCOc1cc(OCCCCCC)ccc1n1c2ccsc2c2c1cc(s2)/C=C(/C(=O)O)\C#N	dichloromethane	49	10.1021/acs.jpcc.6b07356
CC1=CC(C)=C2N1[B-](F)(F)[N+]1=C(C)C=C(C)C1=C2C1=CC=C(C=C1)C(O)=O	dichloromethane	-3	10.1021/acs.jpcc.6b07356
N#C/C(=C\c1ccc(s1)c1ccc(s1)c1ccc(s1)c1ccc(cc1)N(c1ccccc1)c1ccccc1)/C(=O)O	dichloromethane	40	10.1039/C6QM00271D
N#C/C(=C\	dichloromethane	6	10.1039/C6QM00271D

SMILES	SOLVENT	SHIFT (nm)	DOI
c1cc(c(s1)c1ccc(s1)c1ccc(s1)c1ccc(cc1)N(c1ccccc1)c1ccccc1)c1ccc(cc1)N(c1ccccc1)c1ccccc1)/C(=O)O			
N#C/C(=C\ c1cc(c(s1)c1sc(cc1c1ccc(cc1)N(c1ccccc1)c1ccccc1)c1ccc(s1)c1ccc(cc1)N(c1ccccc1)c1ccccc1)c1ccc(cc1)N(c1ccccc1)c1ccccc1)/C(=O)O	dichloromethane	4	10.1039/C6QM00271D
N#C/C(=C\ c1cc(c(s1)c1sc(cc1c1ccc(cc1)N(c1ccccc1)c1ccccc1)c1sc(c(c1)c1ccc(cc1)N(c1ccccc1)c1ccccc1)c1ccc(cc1)N(c1ccccc1)c1ccccc1)c1ccc(cc1)N(c1ccccc1)c1ccccc1)/C(=O)O	dichloromethane	4	10.1039/C6QM00271D
CCCC[C@H](Cn1c2cc3c(cc2c2c1ccc(c2)c1ccc(s1)c1ccc(s1)/C=C(/C(=O)O)\C#N)c1c(n3C[C@H](CCCC)CC)ccc(c1)c1ccc(s1)c1ccc(s1)/C=C(/C(=O)O)\C#N)CC	dichloromethane	-8	10.1021/ol500663b
CCCCCCOc1ccc(cc1)N(c1ccc(cc1)OCCCCCC)c1ccc(cc1)c1ccc(s1)c1ccc(s1)/C=C(/C(=O)O)\C#N	dichloromethane	73	10.1021/acsami.6b10162
CCCCCCOc1ccc(cc1)N(c1ccc(cc1)OCCCCCC)c1ccc(c(c1)c1cc(ccc1c1ccc(s1)c1ccc(s1)/C=C(/C(=O)O)\ C#N)N(c1ccc(cc1)OCCCCCC)c1ccc(cc1)OCCCCCC)c1ccc(s1)c1ccc(s1)/C=C(/C(=O)O)\ C#N	dichloromethane	53	10.1021/acsami.6b10162
CCCCCCOc1ccc(cc1)N(c1ccc(cc1)OCCCCCC)c1ccc(cc1)c1cc(c(s1)c1ccc(s1)/C=C(/C(=O)O)\C#N)c1cc(sc1c1ccc(s1)/C=C(/C(=O)O)\ C#N)c1ccc(cc1)N(c1ccc(cc1)OCCCCCC)c1ccc(cc1)OCCCCCC	dichloromethane	16	10.1021/acsami.6b10162
CCCCCCCCc1cc(sc1c1ccc(s1)c1ccc(cc1)N(c1ccccc1)c1ccccc1)c1ccc(c2c1nsn2)c1cc(c(s1)c1ccc(s1)c1ccc(cc1)/C=C(/C(=O)O)\ C#N)CCCCCCCC	dichloromethane	8	10.1039/c7ta00793k
CCCC[C@H](Cc1cc(sc1c1ccc(s1)c1ccc(cc1)N(c1ccccc1)c1ccccc1)c1ccc(c2c1nsn2)c1cc(c(s1)c1ccc(s1)c1ccc(cc1)/C=C(/C(=O)O)\C#N)C[C@H](CCCC)CC)CC	dichloromethane	18	10.1039/c7ta00793k
CCCCCCCCc1cc(sc1c1cc(c(s1)c1ccc(cc1)N(c1ccccc1)c1ccccc1)CCCCCCCC)c1ccc(c2c1nsn2)c1cc(c(s1)c1ccc(s1)c1ccc(cc1)/C=C(/	dichloromethane	2	10.1039/c7ta00793k

SMILES	SOLVENT	SHIFT (nm)	DOI
C(=O)O)\C#N)CCCCCCCC			
CCCCCCCCc1cc(sc1c1ccc(s1)c1ccc(cc1)N(c1ccc(cc1)CCCCCC)c1ccc(cc1)CCCCCC)c1ccc(c2c1nsn2)c1sc(c(c1)CCCCCCCC)c1ccc(s1)c1ccc(cc1)/C=C(/C(=O)O)\C#N	dichloromethane	9	10.1039/c7ta00793k
N#C/C(=C\c1ccc(s1)c1ccc(cc1)C(=C(c1ccc(cc1)N(CC)CC)c1ccc(cc1)N(CC)CC)c1ccc(cc1)Br)/C(=O)O	dichloromethane	23	10.1021/acs.joc.5b01140
CCN(c1ccc(cc1)C(=C(c1ccc(cc1)c1ccc(s1)/C=C(/C(=O)O)\C#N)c1ccc(cc1)c1ccc(s1)/C=C(/C(=O)O)\C#N)c1ccc(cc1)N(CC)CC)CC	dichloromethane	0	10.1021/acs.joc.5b01140
N#C/C(=C\c1ccc(s1)/C=C/1\C=C(OC(=C1)c1ccc(cc1)C(C)(C)C)c1ccc(cc1)C(C)(C)C)/C(=O)O	dichloromethane	93	10.1039/c5ra23339a
N#C/C(=C\c1sc(c2c1OCCO2)/C=C/1\C=C(OC(=C1)c1ccc(cc1)C(C)(C)C)c1ccc(cc1)C(C)(C)C)/C(=O)O	dichloromethane	86	10.1039/c5ra23339a
N#C/C(=C\c1cnc(s1)/C=C/1\C=C(OC(=C1)c1ccc(cc1)C(C)(C)C)c1ccc(cc1)C(C)(C)C)/C(=O)O	dichloromethane	69	10.1039/c5ra23339a
OC(=O)C(=CC1=CC=C(S1)C1=CC=C(C=C1)N(C1=CC=CC=C1)C1=CC=CC=C1)C#N	dichloromethane	21	10.1039/c5ra23339a
CCCCCCOc1ccc(cc1)N1C2C3SC(C(C3SC2C2C1CC(S2)/C=C(/C(=O)O)\C#N)CCCCCC)c1ccc(cc1)N(c1ccc(cc1)OCCCCCC)c1ccc(cc1)OCCCCCC	dichloromethane	33	10.1021/acs.orglett.7b01465
CCCCCCOc1ccc(cc1)N1C2C3SC(C(C3SC2C2C1CC(S2)c1sc(cc1CCCCCC)/C=C(/C(=O)O)\C#N)CCCCCC)c1ccc(cc1)N(c1ccc(cc1)OCCCCCC)c1ccc(cc1)OCCCCCC	dichloromethane	22	10.1021/acs.orglett.7b01465
CCCCCCOc1ccc(cc1)N1C2CC(SC2C2C1C1SC(C(C1S2)CCCCCC)/C=C(/C(=O)O)\C#N)c1ccc(cc1)N(c1ccc(cc1)OCCCCCC)c1ccc(cc1)OCCCCCC	dichloromethane	33	10.1021/acs.orglett.7b01465
CCCCCCOc1ccc(cc1)N1C2CC(SC2C2C1C1SC(C(C1S2)CCCCCC)/C=C(/C(=O)O)\C#N)c1sc(cc1CCCCCC)c1ccc(cc1)N(c1ccc(cc1)OCCCCCC)c1ccc(cc1)OCCCCCC	dichloromethane	27	10.1021/acs.orglett.7b01465
CCCCCCOc1cc(OCCCCCC)ccc1c1ccc(cc1)N(c1ccc(cc1)c1ccc(cc1OCCCCCC)OCCCCCC)c1ccc(cc1)c1sc2c(c1)C(c1c2sc(c1)/C=C(/C(=O)O)\C#N)(CCCCCC)CCCCCC	dichloromethane	101	10.1021/cm401593b
CCCCCCOC1=CC=C(C=C1)N(C1=CC=C(OCCCCCC)C=C1)C1=CC=C(C=C1)C1=CC2=C(S1)C1	dichloromethane	104	10.1021/cm401593b

SMILES	SOLVENT	SHIFT (nm)	DOI
=C(C=C(S1)C=C(C#N)C(O)=O)C2(CCCCCC)CCCCCC			
CCCCCCOc1cc(OCCCCCC)c2c(c1)C(C)(C)c1c2cc(cc1)N(c1ccc2c(c1)C(C)(C)c1c2c(OCCCCCC)cc(c1)OCCCCCC)c1ccc(cc1)c1sc2c(c1)C(c1c2sc(c1)/C=C(/C(=O)O)\C#N)(CCCCCC)CCCCCC	dichloromethane	86	10.1021/cm401593b
OC(=O)N1C(=S)S/C(=C\c2ccc(s2)c2ccc(cc2)N(c2ccccc2)c2ccccc2)/C1=O	dichloromethane	-21	10.1021/acsami.7b04233
N#C/C(=c\1/s/c(=C\c2ccc(s2)c2ccc(cc2)N(c2ccccc2)c2ccccc2)/c(=O)n1C)/C(=O)O	dichloromethane	-26	10.1021/acsami.7b04233
N#C/C(=C\c1ccc(cc1)c1ccc(c2c1nccn2)c1ccc2c(c1)[C@@H]1CCC[C@@H]1N2c1ccc(cc1)C)/C(=O)O	dichloromethane	16	10.1039/C5TC01195G
N#C/C(=C\c1ccc(cc1)c1ccc(c2c1nc(c1ccc(s1)C)c(n2)c1ccc(s1)C)c1ccc2c(c1)[C@H]1CCC[C@H]1N2c1ccc(cc1)C)/C(=O)O	dichloromethane	22	10.1039/C5TC01195G
N#C/C(=C\c1ccc(s1)c1ccc(c2c1nccn2)c1ccc2c(c1)[C@@H]1CCC[C@@H]1N2c1ccc(cc1)C)/C(=O)O	dichloromethane	66	10.1039/C5TC01195G
N#C/C(=C\c1ccc(s1)c1ccc(c2c1nc(c1ccc(s1)C)c(n2)c1ccc(s1)C)c1ccc2c(c1)[C@H]1CCC[C@H]1N2c1ccc(cc1)C)/C(=O)O	dichloromethane	51	10.1039/C5TC01195G
CCCCCCOc1ccc(cc1)N(c1ccc(cc1)OCCCCCC)c1ccc(cc1)/C=C/c1sc2c(c1)n(c1c2sc(c1)/C=C(/C(=O)O)\C#N)c1ccc(cc1)N1c2ccccc2Sc2c1cccc2	dichloromethane	28	10.1039/C7RA08965A
CCCCCCOc1ccc(cc1)N(c1ccc(cc1)OCCCCCC)c1ccc(cc1)/C=C/c1sc2c(c1)n(c1c2sc(c1)/C=C(/C(=O)O)\C#N)c1ccc2c(c1)C(CCC)(CCC)c1c2c2c(c3c1c1ccccc1C3(CCC)CCC)c1c(C2(CCC)CCC)cccc1	dichloromethane	25	10.1039/C7RA08965A
CCCCCCOc1ccc(cc1)N(c1ccc(cc1)OCCCCCC)c1ccc(cc1)/C=C/c1sc2c(c1)n(c1c2sc(c1)/C=C(/C(=O)O)\C#N)c1ccc(cc1)OCCCCCC	dichloromethane	44	10.1039/C7RA08965A
CCCCCCN1c2ccc(cc2Sc2c1ccc(c2)/C=C(/C(=O)O)\C#N)c1ccc(cc1)OC	dichloromethane	63.5	10.1016/j.dyepig.2018.01.011
CCCCCCN1c2ccc(cc2Sc2c1ccc(c2)c1ccccc1)c	dichloromethane	54	10.1016/

SMILES	SOLVENT	SHIFT (nm)	DOI
1ccc(s1)/C=C(/C(=O)O)\C#N			j.dyepig.2018.01.011
CCCCCCN1c2ccc(cc2Sc2c1ccc(c2)c1ccc(cc1)OC)c1ccc(s1)/C=C(/C(=O)O)\C#N	dichloromethane	60	10.1016/j.dyepig.2018.01.011
CCCCCCN1c2ccc(cc2Sc2c1ccc(c2)c1ccccc1)c1ccc(o1)/C=C(/C(=O)O)\C#N	dichloromethane	64.5	10.1016/j.dyepig.2018.01.011
CCCCCCN1c2ccc(cc2Sc2c1ccc(c2)c1ccc(cc1)OC)c1ccc(o1)/C=C(/C(=O)O)\C#N	dichloromethane	65	10.1016/j.dyepig.2018.01.011
N#C/C(=C/c1ccc(s1)c1ccc(c2c1nc(c1ccccc1)c(n2)c1ccccc1)c1ccc2c(c1)[C@H]1CCC[C@H]1N2c1ccc(cc1)C)/C(=O)O	dichloromethane	34	10.1016/j.solener.2018.02.041
CCCCCCCCC1(CCCCCCCC)c2cc(sc2c2c1cc(s2)/C=C(\C(=O)O)/C#N)c1ccc(c2c1nc(c1ccc(cc1)OC)c(n2)c1ccc(cc1)OC)c1ccc2c(c1)[C@H]1CCC[C@H]1N2c1ccc(cc1)C	dichloromethane	39	10.1016/j.solener.2018.02.041
N#C/C(=C/c1ccc2c(c1)N(c1ccc3c(c1)C(CC)(CC)c1c3cccc1)c1c(S2)cccc1)/C(=O)O	dichloromethane	28	10.1016/j.electacta.2018.02.134
CCCC1(CCC)c2cc(ccc2c2c1cccc2)N1c2ccccc2Sc2c1cc(cc2)/C=C(\C(=O)O)/C#N	dichloromethane	42	10.1016/j.electacta.2018.02.134
CCC1(CC)c2cc(ccc2c2c1cccc2)N1c2cc(ccc2Sc2c1cccc2)/C=C\1/SC(=S)N(C1=O)CC(=O)O	dichloromethane	0	10.1016/j.electacta.2018.02.134
CCCC1(CCC)c2cc(ccc2c2c1cccc2)N1c2cc(ccc2Sc2c1cccc2)/C=C\1/SC(=S)N(C1=O)CC(=O)O	dichloromethane	3	10.1016/j.electacta.2018.02.134
CCCCCCC1(CCCCCC)c2cc(ccc2c2c1cccc2)N1c2ccccc2Sc2c1cc(cc2)/C=C\1/SC(=S)N(C1=O)CC(=O)O	dichloromethane	9	10.1016/j.electacta.2018.02.134
CCCCCCN1c2ccc(cc2Sc2c1ccc(c2)/C=C(/C(=O)O)\C#N)c1ccc(cc1)N(c1ccc(cc1)C)c1ccc(cc1)C	dichloromethane	49	10.1016/j.jphotochem.2017.10.033
CCCCCCN1c2ccc(cc2Sc2c1ccc(c2)/C=C(/C(=O)O)\C#N)c1ccc(cc1)N(c1ccc(cc1)c1ccc2c(c1)Sc1c(N2CCCCCC)ccc(c1)/C=C(/C(=O)O)\C#N)c1ccc(cc1)C	dichloromethane	34	10.1016/j.jphotochem.2017.10.033
CCCCCCN1c2ccc(cc2Sc2c1ccc(c2)/C=C(/C(=O)O)\C#N)c1ccc(cc1)N(c1ccc(cc1)C)c1ccc(cc1)c1ccc(cc1)N(c1ccc(cc1)c1ccc2c(c1)Sc1c(N2CCCCCC)ccc(c1)/C=C(/C(=O)O)\C#N)c1ccc(cc1)C	dichloromethane	36	10.1016/j.jphotochem.2017.10.033
CCCCCCN1c2ccc(cc2Sc2c1ccc(c2)/C=C(/C(=O)O)\	dichloromethane	20	10.1016/j.jphotochem.2017.10.033

SMILES	SOLVENT	SHIFT (nm)	DOI
C#N)c1ccc(cc1)N(c1ccc(cc1)c1ccc2c(c1)Sc1c(N2CCCCCC)ccc(c1)/C=C(/C(=O)O)\C#N)c1ccc(cc1)c1ccc2c(c1)Sc1c(N2CCCCCC)ccc(c1)/C=C(/C(=O)O)\C#N			
N#C/C(=C\c1ccc(s1)c1ccc(c2c1nc(c1ccccc1)c(n2)c1ccccc1)c1ccc2c(c1)C1CCCC1N2c1ccc(cc1)C)/C(=O)O	dichloromethane	16	10.1021/acsami.8b02676
N#C/C(=C\c1ccc(s1)c1ccc(c2c1nc1c(n2)c2ccccc2c2c1cccc2)c1ccc2c(c1)[C@@H]1CCC[C@@H]1N2c1ccc(cc1)C)/C(=O)O	dichloromethane	48	10.1021/acsami.8b02676
CCCCCCCCOc1c(c2ccc3c(c2)[C@H]2CCC[C@H]2N3c2ccc(cc2)C)c2nc3c(nc2c(c1OCCCCCCCC)c1ccc(s1)/C=C(/C(=O)O)\C#N)c1ccccc1c1c3cccc1	dichloromethane	14	10.1021/acsami.8b02676
CCCCCCCCC1(CCCCCCCC)c2cc(sc2c2c1cc(s2)c1ccc(c2c1nc1c3ccccc3c3c(c1n2)cccc3)c1ccc2c(c1)[C@H]1CCC[C@H]1N2c1ccc(cc1)C)/C=C(/C(=O)O)\C#N	dichloromethane	14	10.1021/acsami.8b02676
CCCCCCCCCCCCN(c1ccc(cc1)n1c2ccc(cc2c2c1ccc(c2)C(C)(C)C)C(C)(C)C)c1ccc(cc1)c1ccc(s1)/C=C(/C(=O)O)\C#N	dichloromethane	28	10.1021/jp304489t
CCCCCCCCCCCCN(c1ccc(cc1)n1c2ccc(cc2c2c1ccc(c2)C(C)(C)C)C(C)(C)C)c1ccc(cc1)c1ccc(s1)c1ccc(s1)/C=C(/C(=O)O)\C#N	dichloromethane	28	10.1021/jp304489t
CCCCCCCCCCCCN(c1ccc(cc1)n1c2ccc(cc2c2c1ccc(c2)C(C)(C)C)C(C)(C)C)c1ccc(cc1)c1ccc(s1)c1ccc(s1)c1ccc(s1)/C=C(/C(=O)O)\C#N	dichloromethane	20	10.1021/jp304489t
CCCCCCN1c2ccc(cc2Sc2c1ccc(c2)c1ccc(cc1)OC[C@H](CCCC)CC)c1ccc(c2c1nn(n2)C[C@H](CCCC)CC)c1cc2c(s1)c1c([nH]2)cc(s1)[C@@H](C(=O)O)C#N	dichloromethane	1	10.1016/j.jpowsour.2018.03.059
CCCCCCN1c2ccc(cc2Sc2c1ccc(c2)c1ccc(cc1)OC[C@H](CCCC)CC)c1ccc(c2c1nsn2)c1cc2c(s1)c1c([nH]2)cc(s1)[C@@H](C(=O)O)C#N	dichloromethane	4	10.1016/j.jpowsour.2018.03.059
CCCCCCN1c2ccc(cc2Sc2c1ccc(c2)c1ccc(cc1)OC[C@H]	dichloromethane	1	10.1016/j.jpowsour.2018.03.059

SMILES	SOLVENT	SHIFT (nm)	DOI
(CCCC)CC)c1ccc(c2c1nc(c1ccc(cc1)OC[C@H](CCCC)CC)c(n2)c1ccc(cc1)OC[C@H](CCCC)CC)c1cc2c(s1)c1c([nH]2)cc(s1)[C@@H](C(=O)O)C#N			
CCCCCCN1c2ccc(cc2Sc2c1ccc(c2)c1cc2c(s1)c1c([nH]2)cc(s1)[C@@H](C(=O)O)C#N)c1ccc(cc1)OC[C@H](CCCC)CC	dichloromethane	17	10.1016/j.jpowsour.2018.03.059
CCCCCCCCOc1ccc(cc1)N(c1ccc(cc1)/C=c/1\sc(=C(C#N)C#N)[nH]c1=O)c1ccc(cc1)OCCCCCCCC	dichloromethane	11	10.1002/ange.201204948
CCCCCCCCOc1ccc(cc1)N(c1ccc(cc1)OCCCCCCCC)c1ccc(cc1)c1ccc(s1)/C=c/1\sc(=C(C#N)C#N)[nH]c1=O	dichloromethane	42	10.1002/ange.201204948
CCCCCCCCOc1ccc(cc1)N(c1ccc(cc1)/C=C(/C(=O)O)\C#N)c1ccc(cc1)OCCCCCCCC	dichloromethane	17	10.1002/ange.201204948
CCCCCCCCOc1ccc(cc1)N(c1ccc(cc1)c1ccc(s1)/C=C(/C(=O)O)\C#N)c1ccc(cc1)OCCCCCCCC	dichloromethane	19	10.1002/ange.201204948
CCCCCCCCCC[C@]1(CCCCCCCC)C(=[N+](c2c1cc1c3ccccc3C(c1c2)(CCCCCCCC)CCCCCCCC)C)/C=C/1\C(=C(C1=O)/C=C\1/N(C)c2c(C1(C)C)cc(cc2)C(=O)O)[O-]	dichloromethane	-5	10.1021/acsami.8b09866
CCCCCCCCCC[C@]1(CCCCCCCC)C(=[N+](c2c1cc1c3ccccc3C(c1c2)(CCCCCCCC)CCCCCCCC)CCCCCC)/C=C/1\C(=C(C1=O)/C=C\1/N(C)c2c(C1(C)C)cc(cc2)C(=O)O)[O-]	dichloromethane	-6	10.1021/acsami.8b09866
CCCCCCCCCC[C@]1(CCCCCCCC)C(=[N+](c2c1cc1c3ccccc3C(c1c2)(CCCCCCCC)CCCCCCCC)C)/C=C/1\C(=C(C1=O)/C=C\1/N(C)c2c(C1(C)C)c1ccc(cc1cc2)C(=O)O)[O-]	dichloromethane	-5	10.1021/acsami.8b09866
CCCCCCCCCC[C@]1(CCCCCCCC)C(=[N+](c2c1cc1c3ccccc3C(c1c2)(CCCCCCCC)CCCCCCCC)CCCCCC)/C=C/1\C(=C(C1=O)/C=C/1\N(C)c2c(C1(C)C)c1ccc(cc1cc2)C(=O)O)[O-]	dichloromethane	-5	10.1021/acsami.8b09866
N#C/C(=C\c1ccc(s1)c1ccc(c2c1nc(c1ccccc1)c(n2)c1ccccc1)c1ccc2c(c1)[C@H]1CCC[C@H]1N2c1ccc(cc1)C)/C(=O)O	dichloromethane	16	10.1021/acsami.8b02676
N#C/C(=C\c1ccc(s1)c1ccc(c2c1nc1c(n2)c2ccccc2c2c1cccc2)c1ccc2c(c1)	dichloromethane	48	10.1021/acsami.8b02676

SMILES	SOLVENT	SHIFT (nm)	DOI
[C@H]1CCC[C@H]1N2c1ccc(cc1)C)/C(=O)O			
CCCCCCCCOc1c(c2ccc3c(c2)C2CCCC2N3c2ccc(cc2)C)c2nc3c(nc2c(c1OCCCCCCCC)c1ccc(s1)/C=C(/C(=O)O)\C#N)c1ccccc1c1c3cccc1	dichloromethane	14	10.1021/acsami.8b02676
CCCCCCCCC1(CCCCCCCC)c2cc(sc2c2c1cc(s2)c1ccc(c2c1nc1c3ccccc3c3c(c1n2)cccc3)c1ccc2c(c1)C1CCCC1N2c1ccc(cc1)C)/C=C(/C(=O)O)\C#N	dichloromethane	14	10.1021/acsami.8b02676
N#Cc1ccc(cn1)c1ccc(c2c1nccn2)c1ccc(cc1)N(c1ccc(cc1)C)c1ccc(cc1)C	dichloromethane	43	10.1002/ajoc.201300178
N#Cc1ccc(cn1)c1ccc(c2c1nsn2)c1ccc(cc1)N(c1ccc(cc1)C)c1ccc(cc1)C	dichloromethane	19	10.1002/ajoc.201300178
N#Cc1ccc(cn1)c1cnc(c2c1nsn2)c1ccc(cc1)N(c1ccc(cc1)C)c1ccc(cc1)C	dichloromethane	5	10.1002/ajoc.201300178
Cc1ccc(cc1)N(c1ccc(cc1)C)c1ccc(cc1)c1ccc(c2c1nsn2)c1ccncc1	dichloromethane	4	10.1002/ajoc.201300178
c1ccc(cc1)N(c1ccccc1)c1ccc(cc1)c1ccncc1	dichloromethane	-1	10.1039/C3RA41182F
[O-][n+]1ccc(cc1)c1ccc(cc1)N(c1ccccc1)c1ccccc1	dichloromethane	-11	10.1039/C3RA41182F
[O-][n+]1ccc(cc1)c1ccc(s1)c1ccc(cc1)N(c1ccccc1)c1ccccc1	dichloromethane	-5	10.1039/C3RA41182F
CCCCCCc1cc(sc1c1ccc(cc1)N(c1ccccc1)c1ccccc1)c1cc[n+](cc1)[O-]	dichloromethane	-11	10.1039/C3RA41182F
N#C/C(=C\c1ccc(cc1)/C=C/c1ccc(cc1)N(c1ccc(cc1)OC)c1ccc(cc1)OC)/c1n[nH]nn1	dichloromethane	45	10.1021/jp502488g
N#C/C(=C\c1ccc(cc1)/C=C/c1ccc(cc1)N(c1ccc(cc1)OC)c1ccc(cc1)OC)/C(=O)O	dichloromethane	52	10.1021/jp502488g
N#C/C(=C\c1ccc(cc1)/C=C/c1ccc(cc1)N(c1ccc(cc1)OC)c1ccc(cc1)OC)/c1ccncc1	dichloromethane	8	10.1021/jp502488g
OC(=O)CNc1ccc2c3c1cccc3c1c3c2ccc2c3c(cc1)c(=O)n(c2=O)c1cc(ccc1C(C)(C)C)C(C)(C)C	dichloromethane	6	10.1039/B203260K
CC(c1cccc(c1n1c(=O)c2cc(Oc3ccccc3)c3c4c2c(c1=O)cc(c4c1c2c3c(Oc3ccccc3)cc3c2c(cc1Oc1ccccc1)C(=O)OC3=O)Oc1ccccc1)C(C)C)C	dichloromethane	49	10.1021/jp076447c
CN(c1ccc(cc1)C#Cc1ccc2c3c1cccc3c1c3c2c(Oc2ccc(cc2)C(CC(C)(C)C)(C)C)cc2c3c(cc1Oc1ccc(cc1)C(CC(C)(C)C)(C)C)C(=O)OC2=O)C	dichloromethane	110	10.1021/jp076447c

SMILES	SOLVENT	SHIFT (nm)	DOI
O=C1OC(=O)c2c3c1ccc1c3c(cc2)c2c3c1ccc(c3ccc2)N(c1ccc(cc1)C(CC(C)(C)C)(C)C)c1ccc(cc1)C(CC(C)(C)C)(C)C	dichloromethane	127	10.1021/jp076447c
O=C1OC(=O)c2c3c1cc(Oc1ccc(cc1)C(CC(C)(C)C)(C)C)c1c3c(c(c2)Oc2ccc(cc2)C(CC(C)(C)C)(C)C)c2c3c1cccc3c(cc2)N(c1ccc(cc1)C(CC(C)(C)C)(C)C)c1ccc(cc1)C(CC(C)(C)C)(C)C	dichloromethane	129	10.1021/jp076447c
O=C1OC(=O)c2c3c1cc(Oc1ccc(cc1)C(CC(C)(C)C)(C)C)c1c3c(c(c2)Oc2ccc(cc2)C(CC(C)(C)C)(C)C)c2c3c1cccc3c(cc2)N(c1ccccc1)c1ccccc1	dichloromethane	110	10.1021/jp076447c
N#Cc1cc(ccc1O)c1ccc(s1)c1ccc(cc1)N(c1ccccc1)c1ccccc1	dichloromethane	0	10.1016/j.tetlet.2012.04.049
N#Cc1cc(ccc1O)c1cc(c(s1)c1ccc(cc1)N(c1ccccc1)c1ccccc1)C	dichloromethane	-2	10.1016/j.tetlet.2012.04.049
CCCCCCc1cc(sc1c1ccc(cc1)N(c1ccccc1)c1ccccc1)c1ccc(c(c1)C#N)O	dichloromethane	-5	10.1016/j.tetlet.2012.04.049
CCCCCCc1cc(sc1c1cc(c(s1)c1ccc(cc1)N(c1ccccc1)c1ccccc1)C)c1ccc(c(c1)C#N)O	dichloromethane	-1	10.1016/j.tetlet.2012.04.049
N#C/C(=C\c1cc2c(s1)cc(s2)/C=C/1\C=C(OC(=C1)c1ccccc1)c1ccccc1)/C(=O)O	dichloromethane	87	10.1016/j.dyepig.2018.09.035
N#C/C(=C\c1cc2c(s1)cc(s2)/C=C/1\C=C(OC(=C1)C(C)(C)C)C(C)(C)C)/C(=O)O	dichloromethane	84	10.1016/j.dyepig.2018.09.035
N#C/C(=C\C=C\c1cc2c(s1)cc(s2)/C=C/1\C=C(OC(=C1)c1ccccc1)c1ccccc1)/C(=O)O	dichloromethane	102	10.1016/j.dyepig.2018.09.035
CCCCCCc1c(/C=C/2\C=C(OC(=C2)c2ccccc2)c2ccccc2)sc2c1sc(c2CCCCCC)/C=C(/C(=O)O)\C#N	dichloromethane	78	10.1016/j.dyepig.2018.09.035
CCCCCCc1c(/C=C/c2sc(c(c2CCCCCC)CCCCCC)/C=C(/C(=O)O)\C#N)sc(c1CCCCCC)/C=C/1\C=C(OC(=C1)c1ccccc1)c1ccccc1	dichloromethane	67	10.1016/j.dyepig.2018.09.035
CCCCCCn1c2cc(ccc2c2c1c1c(s2)c2c(n1CCCCCC)cc(s2)/C=C(/C(=O)O)\C#N)c1ccc(cc1)N(c1ccccc1)c1ccccc1	dichloromethane	51	10.1016/j.dyepig.2017.09.053
CCCCCCOc1ccc(cc1)N(c1ccc(cc1)OCCCCCC)c1ccc(cc1)c1ccc(cc1)c1cc2c(s1)c1c(n2CCCCCC)cc(s1)/C=C(/C(=O)O)\C#N	dichloromethane	57	10.1016/j.dyepig.2017.09.053
CCCCCCn1c2cc(ccc2c2c1c1n(CCCCCC)c3c(c1s2)sc(c3)/C=C(/C(=O)O)\C#N)c1ccc(cc1)N(c1ccc(cc1)OCCCCCC)c1ccc(cc1)OCCCCCC	dichloromethane	70	10.1016/j.dyepig.2017.09.053

SMILES	SOLVENT	SHIFT (nm)	DOI
CCCCCCc1cc(sc1c1ccc(cc1)N(c1ccc(cc1)OCCCCCC)c1ccc(cc1)OCCCCCC)c1ccc2c(c1)n(CCCCCC)c1c2sc2c1n(CCCCCC)c1c2sc(c1)/C=C(/C(=O)O)\C#N	dichloromethane	42	10.1016/j.dyepig.2017.09.053
CCCCCCn1c2c(c3c1cc(s3)/C=C(/C(=O)O)\C#N)sc1c2n(CCCCCC)c2c1cccc2	dichloromethane	77	10.1016/j.dyepig.2017.09.053
CCCCCCCCOc1nc(cc(c1C#N)c1ccc2c(c1)n(CC)c1c2cccc1)c1ccc(s1)c1ccc(s1)/C=C(/C(=O)O)\C#N	dichloromethane	-3	10.1016/j.solener.2018.09.073
CCCCCCCCOc1nc(cc(c1C#N)c1ccc2c(c1)n(CC)c1c2cccc1)c1ccc(cc1)c1ccc(s1)/C=C(/C(=O)O)\C#N	dichloromethane	2	10.1016/j.solener.2018.09.073
CCCCCCOc1ccc(cc1)N(c1ccc(cc1)OCCCCCC)c1ccc(cc1)c1cc(c(s1)c1sc2c(c1)n(c1c2sc(c1)c1cc(c(s1)/C=C(\C(=O)O)/C#N)CCCCCC)c1ccc(cc1)OCCCCCC)CCCCCC	dichloromethane	26	10.1016/j.dyepig.2018.10.018
CCCCCCOc1ccc(cc1)N1C2C3SC(C(C3SC2C2C1C1SC(C(C1S2)CCCCCC)/C=C(/C(=O)O)\C#N)CCCCCC)c1ccc(cc1)N(c1ccc(cc1)OCCCCCC)c1ccc(cc1)OCCCCCC	dichloromethane	29	10.1016/j.dyepig.2018.10.018
CCCCCCC1C2SC3C(C2SC1c1ccc(cc1)N(c1ccccc1)c1ccccc1)N(C1C3SC2C1SC(C2CCCCCC)/C=C(/C(=O)O)\C#N)c1ccc(cc1)OCCCCCC	dichloromethane	31	10.1016/j.dyepig.2018.10.018
CCCCCCC1C2SC3C(C2SC1c1ccc(cc1)N(c1ccccc1)c1ccccc1)N(C1C3SC2C1SC(C2CCCCCC)c1ccc(c2c1nsn2)/C=C(\C(=O)O)/C#N)c1ccc(cc1)OCCCCCC	dichloromethane	-27	10.1016/j.dyepig.2018.10.018
CCCC[C@@H](COc1ccc(cc1)n1c(cc2c1cc(n2c1ccc(cc1)OC[C@H](CCCC)CC)c1ccc(cc1)c1ccc(cc1)C=C(C(=O)O)C#N)c1ccc(cc1)c1ccc(cc1)C[C@H](CCCC)CC)CC	dichloromethane	-215	10.1002/chem.201803688
CCCC[C@@H](COc1ccc(cc1)n1c(cc2c1cc(n2c1ccc(cc1)OC[C@H](CCCC)CC)c1ccc(cc1)c1ccc(s1)C=C(C(=O)O)C#N)c1ccc(cc1)c1ccc(cc1)C[C@H](CCCC)C)CC	dichloromethane	-167	10.1002/chem.201803688
CCCC[C@@H](COc1ccc(cc1)n1c(cc2c1cc(n2c1ccc(cc1)OC[C@H](CCCC)CC)c1ccc(cc1)c1ccc(s1)C=C(C(=O)O)C#N)c1ccc(cc1)c1ccc(cc1)C(=C(c1ccccc1)c1ccccc1)c1ccccc1)CC	dichloromethane	-202	10.1002/chem.201803688

SMILES	SOLVENT	SHIFT (nm)	DOI
CCCC[C@@H](COc1ccc(cc1)n1c(cc2c1cc(n2c1ccc(cc1)OC[C@H](CCCC)CC)c1ccc(cc1)c1ccc(cc1)C=C(C(=O)O)C#N)c1ccc(cc1)c1ccc(cc1)C(=C(c1ccccc1)c1ccccc1)c1ccccc1)CC	dichloromethane	-182	10.1002/chem.201803688
CCCCCCCCOc1ccc(cc1)n1c(cc2c1cc(n2c1ccc(cc1)OCCCCCCCC)c1ccc(cc1)C(=C(c1ccccc1)c1ccccc1)c1ccccc1)c1ccc(cc1)c1ccc(s1)C=Cc1ccc(c2c1csc2)c1ccc(cc1)C=C(C(=O)O)C#N	dichloromethane	-219	10.1002/chem.201803688
CCCCCCCCOc1ccc(cc1)n1c(cc2c1cc(n2c1ccc(cc1)OCCCCCCCC)c1ccc(cc1)C(=C(c1ccccc1)c1ccccc1)c1ccccc1)c1ccc(cc1)c1ccc(s1)C=Cc1ccc(c2c1csc2)c1ccc(s1)C=C(C(=O)O)C#N	dichloromethane	-219	10.1002/chem.201803688
CCOC(=O)/C(=C/c1ccc(cc1)N(c1ccccc1)c1ccccc1)/c1nnn[nH]1	dichloromethane	14	10.1016/j.jechem.2015.10.015
CCOC(=O)/C(=C/c1ccc(cc1)N(c1ccc(cc1)N1c2ccccc2Sc2c1cccc2)c1ccc(cc1)N1c2ccccc2Sc2c1cccc2)/c1nnn[nH]1	dichloromethane	0	10.1016/j.jechem.2015.10.015
CCOC(=O)/C(=C/c1ccc(cc1)N(c1ccc(cc1)n1c2ccccc2c2c1cccc2)c1ccc(cc1)n1c2ccccc2c2c1cccc2)/c1nnn[nH]1	dichloromethane	17	10.1016/j.jechem.2015.10.015
CCOC(=O)/C(=C/c1ccc(cc1)N(c1ccc(cc1)N(c1ccccc1)c1ccccc1)c1ccc(cc1)N(c1ccccc1)c1ccccc1)/c1nnn[nH]1	dichloromethane	11	10.1016/j.jechem.2015.10.015
N#CC(=Cc1ccc(cc1)N(c1ccccc1)c1ccccc1)c1[nH]nnn1	dichloromethane	30	10.1016/j.jechem.2015.10.015
OC(=O)/C(=C/c1ccc(cc1)N(c1ccccc1)c1ccccc1)/c1[nH]nnn1	dichloromethane	-24	10.1016/j.jechem.2015.10.015
CCOC(=O)C(=Cc1ccc(cc1)N(c1ccc(cc1)C=C(c1nnn[nH]1)C(=O)OCC)c1ccccc1)c1nnn[nH]1	dichloromethane	5	10.1016/j.saa.2017.01.061
N#CC(=Cc1ccc(cc1)N(c1cccc(c1)C=C(c1[nH]nnn1)C#N)c1ccccc1)c1[nH]nnn1	dichloromethane	11	10.1016/j.saa.2017.01.061
OC(=O)/C(=C/c1ccc(cc1)N(c1ccc(cc1)/C=C(\c1nnn[nH]1)/C(=O)O)c1ccccc1)/c1nnn[nH]1	dichloromethane	-8	10.1016/j.saa.2017.01.061
CCCCCCOc1c2cc(sc2c(c2c1sc(c2)c1ccc(cc1)C=C(C(=O)O)C#N)OCCCCCC)c1ccc2c(c1)	dichloromethane	-20	10.1021/acsami.5b07690

SMILES	SOLVENT	SHIFT (nm)	DOI
[C@H]1CCC[C@H]1N2c1ccc(cc1)C			
CCCCCCOc1c2cc(sc2c(c2c1sc(c2)c1cnc(nc1)C(=O)O)OCCCCCC)c1ccc2c(c1)[C@H]1CCC[C@H]1N2c1ccc(cc1)C	dichloromethane	-16	10.1021/acsami.5b07690
CCCCCCCCCC[C@@]1(CCCCCCCC)C(=[N+](c2c1cc1c3ccccc3C(c1c2)(CCCCCCCC)CCCCCCCC)C)/C=C/1\C(=C(C1=O)/C=C\1/N(C)c2c(C1(C)C)cc(cc2)C(=O)O)[O-]	dichloromethane	-5	10.1021/acsaem.8b00834
CCCCCCCCCC[C@@]1(CCCCCCCC)C(=[N+](c2c1cc1c3ccccc3C(c1c2)(CCCCCCCC)CCCCCCCC)CCCCCC)/C=C/1\C(=C(C1=O)/C=C\1/N(C)c2c(C1(C)C)cc(cc2)C(=O)O)[O-]	dichloromethane	-6	10.1021/acsaem.8b00834
CCCCCCCCCC[C@@]1(CCCCCCCC)C(=[N+](c2c1cc1c3ccccc3C(c1c2)(CCCCCCCC)CCCCCCCC)C)/C=C/1\C(=C(C1=O)/C=C\1/N(C)c2c(C1(C)C)c1ccc(cc1cc2)C(=O)O)[O-]	dichloromethane	-5	10.1021/acsaem.8b00834
CCCCCCCCCC[C@@]1(CCCCCCCC)C(=[N+](c2c1cc1c3ccccc3C(c1c2)(CCCCCCCC)CCCCCCCC)CCCCCC)/C=C/1\C(=C(C1=O)/C=C\1/N(C)c2c(C1(C)C)c1ccc(cc1cc2)C(=O)O)[O-]	dichloromethane	-5	10.1021/acsaem.8b00834
CCCCn1c(=O)c2c(c1c1ccc(cc1)c1ccc(cc1)N(c1ccc(cc1)OC)c1ccc(cc1)OC)c(=O)n(c2c1ccc(cc1)c1ccc(s1)/C=C(/C(=O)O)\C#N)CCCC	dichloromethane	-81	10.1021/jp909786k
CCCCn1c(=O)c2c(c1c1ccc(s1)c1ccc(cc1)N(c1ccc(cc1)OC)c1ccc(cc1)OC)c(=O)n(c2c1ccc(cc1)c1ccc(s1)/C=C(/C(=O)O)\C#N)CCCC	dichloromethane	-75	10.1021/jp909786k
CCCCn1c(=O)c2c(c1c1ccc(cc1)c1ccc(cc1)N(c1ccc(cc1)OC)c1ccc(cc1)OC)c(=O)n(c2c1ccc(cc1)c1ccc(o1)/C=C(/C(=O)O)\C#N)CCCC	dichloromethane	-65	10.1016/j.dyepig.2011.09.009
CCCCn1c(c2ccc(cc2)c2ccc(cc2)N(c2ccc(cc2)OC)c2ccc(cc2)OC)c2c(c1=O)c(n(c2=O)CCCC)c1ccc(cc1)c1ccc(cc1)/C=C(/C(=O)O)\C#N	dichloromethane	-55	10.1016/j.dyepig.2011.09.009
CCCCC(Cn1c(c2ccc(cc2)c2ccc(s2)/C=C(/C(=O)O)\C#N)c2c(c1=O)c(n(c2=O)CC(CCCC)CC)c1ccc(cc1)Br)CC	dichloromethane	-7	10.1016/j.jphotochem.2011.09.023
CCCCC(Cn1c(=O)c2c(c1c1ccc(cc1)c1ccc(s1)/C=C\1/SC(=S)N(C1=O)CC(=O)O)c(=O)n(c2c1ccc(cc1)Br)CC(CCCC)CC)CC	dichloromethane	-7	10.1016/j.jphotochem.2011.09.023

SMILES	SOLVENT	SHIFT (nm)	DOI
C1(=CC=CC=C1)N(C1=CC=C(C=C1)C1=CC=C(C=C1)C=1N(C(C2=C(N(C(C21)=O)CCCCCCCC)C2=CC=C(C=C2)C2=CC=C(C=C2)N(C2=CC=CC=C2)C2=CC=CC=C2)=O)CC(=O)O)C2=CC=CC=C2	dichloromethane	-58	10.1016/j.synthmet.2010.06.017
COC1=CC=C(C=C1)N(C1=CC=C(C=C1)C1=CC=C(C=C1)C=1N(C(C2=C(N(C(C21)=O)CCCCCCCC)C2=CC=C(C=C2)C2=CC=C(C=C2)N(C2=CC=C(C=C2)OC)C2=CC=C(C=C2)OC)=O)CC(=O)O)C2=CC=C(C=C2)OC	dichloromethane	-61	10.1016/j.synthmet.2010.06.017
CCCCn1c(c2ccc(s2)c2ccc(cc2)N(c2ccc(cc2)OC)c2ccc(cc2)OC)c2c(c1=O)c(n(c2=O)CCCC)c1ccc(s1)c1ccc(s1)C=O	dichloromethane	35	10.1016/j.solener.2012.05.003
CCCCn1c(c2ccc(s2)c2ccc(cc2)N(c2ccc(cc2)OC)c2ccc(cc2)OC)c2c(c1=O)c(n(c2=O)CCCC)c1ccc(s1)c1ccc(s1)/C=C(/C(=O)O)\C#N	dichloromethane	-75	10.1016/j.solener.2012.05.003
CCCCCCCCOc1ccc(c(c1)OCCCCCCCC)c1cc(ccc1N(c1ccc2c(c1)C(C)(C)c1c2sc(c1)c1c(F)c(F)c(c2c1nsn2)c1ccc(s1)C=C(C(=O)O)C#N)c1ccc(cc1)c1ccc(cc1OCCCCCCCC)OCCCCCCCC)c1ccc(cc1OCCCCCCCC)OCCCCCCCC	dichloromethane	16	10.1021/acssuschemeng.6b00574
CCCCCCCCOc1ccc(c(c1)OCCCCCCCC)c1cc(ccc1N(c1ccc2c(c1)C(C)(C)c1c2sc(c1)c1ccc(c2c1nsn2)c1ccc(s1)C=C(C(=O)O)C#N)c1ccc(cc1)c1ccc(cc1OCCCCCCCC)OCCCCCCCC)c1ccc(cc1OCCCCCCCC)OCCCCCCCC	dichloromethane	16	10.1021/acssuschemeng.6b00574
CCCCCCCCOc1ccc(c(c1)OCCCCCCCC)c1cc(ccc1N(c1ccc2c(c1)C(C)(C)c1c2sc(c1)c1ncc(c2c1nc(c1ccccc1)c(n2)c1ccccc1)c1ccc(s1)C=C(C(=O)O)C#N)c1ccc(cc1)c1ccc(cc1OCCCCCCCC)OCCCCCCCC)c1ccc(cc1OCCCCCCCC)OCCCCCCCC	dichloromethane	18	10.1021/acssuschemeng.6b00574
CCCCCCC1(CCCCCC)c2c3c4ccc(cc4C(c3c3c(c2c2c1cccc2)C(CCCCCC)(CCCCCC)c1c3cccc1)(CCCCCC)CCCCCC)N(c1ccc(cc1)N(c1ccc2c(c1)c1ccccc1C2(CCC)CCC)c1ccc2c(c1)c1ccccc1C2(CCC)CCC)c1ccc(cc1)c1cc2c(s1)c1c(n2CCCCCC)cc(s1)/C=C(/C(=O)O)\C#N	dichloromethane	39	10.1021/acsami.5b06481
CCCCCCN(c1ccc(cc1)N(c1ccc2c(c1)C(CCCCCC)(CCCCCC)c1c2c2c(c3c1c1ccccc1C3(CCCCCC)CCCCCC)c1c(C2(CCCCCC)CCCCCC)cccc1)c1ccc(cc1)c1cc2c(s1)c1c(n2CCCCCC)cc(s1)/	dichloromethane	41	10.1021/acsami.5b06481

SMILES	SOLVENT	SHIFT (nm)	DOI
C=C(/C(=O)O)\C#N)CCCCCC			
CCCCCCn1c2ccsc2c2c1cc(s2)c1ccc(cc1)N(c1ccc2c(c1)C(CCCCCC)(CCCCCC)c1c2c2c(c3c1c1ccccc1C3(CCCCCC)CCCCCC)c1c(C2(CCCCCC)CCCCCC)cccc1)c1ccc(cc1)c1sc2c(c1)n(c1c2sc(c1)/C=C(/C(=O)O)\C#N)CCCCCC	dichloromethane	59	10.1021/acsami.5b06481
CCCCCCN1C2=C(SC(\C=C(/C#N)C(O)=O)=C2)C2=C1C=C(S2)C1=CC=C(C=C1)N(C1=CC=C(C=C1)N1C2=C(SC=C2)C2=C1C=CS2)C1=CC2=C(C=C1)C1=C3C(C4=CC=CC=C4C3(CCCCCC)CCCCCC)=C3C(C4=CC=CC=C4C3(CCCCCC)CCCCCC)=C1C2(CCCCCC)CCCCCC	dichloromethane	23	10.1021/acsami.5b06481
N#C/C(=C/c1ccc(s1)c1ccc(c2c1nc(c1ccccc1)c(n2)c1ccccc1)c1ccc2c(c1)N(c1ccc(cc1)C)C1C2CCC1)/C(=O)O	dichloromethane	19	10.1021/jp412259t
CCCCCCc1cc(sc1c1ccc2c(c1)N(c1ccc(cc1)C)C1C2CCC1)c1ccc(c2c1nc(c1ccccc1)c(n2)c1ccccc1)c1sc(c(c1)CCCCCC)/C=C(\C(=O)O)/C#N	dichloromethane	-7	10.1021/jp412259t
CCCCCCc1cc(sc1c1ccc2c(c1)N(c1ccc(cc1)C)C1C2CCC1)c1ccc(c2c1nc(c1ccccc1)c(n2)c1ccccc1)c1sc(c(c1)CCCCCC)c1ccc(s1)/C=C(\C(=O)O)/C#N	dichloromethane	-57	10.1021/jp412259t
N#C/C(=C/c1ccc(s1)c1ccc(s1)c1ccc(c2c1nc(c1ccccc1)c(n2)c1ccccc1)c1ccc2c(c1)N(c1ccc(cc1)C)C1C2CCC1)/C(=O)O	dichloromethane	37	10.1021/jp412259t
CCCCCCCCOc1c(OCCCCCCCC)c(c2ccc(s2)N(c2ccccc2)c2ccccc2)c2c(c1c1ccc(cc1)/C=C(/C(=O)O)\C#N)nsn2	dichloromethane	16	10.1039/C4TC00169A
CCCCCCCCOc1c(OCCCCCCCC)c(c2ccc(s2)N(c2ccccc2)c2ccccc2)c2c(c1c1ccc(o1)/C=C(\C(=O)O)/C#N)nsn2	dichloromethane	17	10.1039/C4TC00169A
CCCCCCCCOc1c(OCCCCCCCC)c(c2ccc(s2)N(c2ccccc2)c2ccccc2)c2c(c1c1ccc(s1)/C=C(\C(=O)O)/C#N)nsn2	dichloromethane	25	10.1039/C4TC00169A
CCCCCCc1cc(sc1c1cnc(c2c1nsn2)c1ccc(cc1)N(c1ccc(cc1)OCCCCCC)c1ccc(cc1)OCCCCCC)c1ccc(s1)/C=C(/C(=O)O)\C#N	dichloromethane	11	10.1039/C4TA05350H
CCCCCCOc1ccc(cc1)N(c1ccc(cc1)OCCCCCC)c1ccc(cc1)c1ncc(c2c1nsn2)c1sc(cc1CCCCCC)c1sc(c(c1)CCCCCC)/C=C(/C(=O)O)\C#N	dichloromethane	9	10.1039/C4TA05350H

SMILES	SOLVENT	SHIFT (nm)	DOI
CCCCCCc1cc(sc1c1cnc(c2c1nsn2)c1ccc(cc1)N(c1ccc(cc1)OCCCCCC)c1ccc(cc1)OCCCCCC)c1ccc(cc1)/C=C(/C(=O)O)\C#N	dichloromethane	3	10.1039/C4TA05350H
CCCCCCc1cc(sc1c1ncc(c2c1nsn2)c1sc(cc1CCCCCC)c1ccc(cc1)/C=C(/C(=O)O)\C#N)c1ccc(cc1)N(c1ccc(cc1)OCCCCCC)c1ccc(cc1)OCCCCCC	dichloromethane	2	10.1039/C4TA05350H
CCCCCCOc1ccc(cc1)N(c1ccc(cc1)OCCCCCC)c1ccc(cc1)c1ccc(s1)c1ncc(c2c1nsn2)c1ccc(s1)c1ccc(cc1)/C=C(/C(=O)O)\C#N	dichloromethane	33	10.1039/C4TA05350H
CCCCCCN1c2ccc(cc2CCc2c1ccc(c2)c1ccc(s1)/C=C(/C(=O)O)\C#N)c1ccc(cc1)N(c1ccccc1)c1ccccc1	dichloromethane	26	10.1016/j.dyepig.2013.08.023
OCCCCCCN1c2ccc(cc2CCc2c1ccc(c2)c1ccc(s1)/C=C(/C(=O)O)\C#N)c1ccc(cc1)N(c1ccccc1)c1ccccc1	dichloromethane	49	10.1016/j.dyepig.2013.08.023
N#C/C(=C\c1ccc(s1)c1ccc2c(c1)CCc1c(N2CCCCCCOc2ccc(cc2)C(=O)O)ccc(c1)c1ccc(cc1)N(c1ccccc1)c1ccccc1)/C(=O)O	dichloromethane	6	10.1016/j.dyepig.2013.08.023
N#CC(=Cc1ccc(s1)c1cnc(c2c1nsn2)c1ccc(cc1)N(c1ccccc1)c1ccccc1)C(=O)O	dichloromethane	32	10.1016/j.tet.2014.04.039
N#CC(=Cc1ccc(cc1)c1cnc(c2c1nsn2)c1ccc(cc1)N(c1ccccc1)c1ccccc1)C(=O)O	dichloromethane	24	10.1016/j.tet.2014.04.039
N#CC(=Cc1ccc(s1)c1cnc(c2c1nsn2)c1ccc(s1)N(c1ccccc1)c1ccccc1)C(=O)O	dichloromethane	66	10.1016/j.tet.2014.04.039
N#CC(=Cc1ccc(cc1)c1cnc(c2c1nsn2)c1ccc(s1)N(c1ccccc1)c1ccccc1)C(=O)O	dichloromethane	51	10.1016/j.tet.2014.04.039
CCCCCCCCN1c2cc(ccc2/C(=C\2/c3ccc(cc3N(C2=O)CCCCCCCC)c2ccc(s2)/C=C(\C(=O)O)/C#N)/C1=O)c1ccc(cc1)N(c1ccccc1)c1ccccc1	dichloromethane	21	10.1021/am300925e
CCCCCCCCN1c2cc(ccc2/C(=C\2/c3ccc(cc3N(C2=O)CCCCCCCC)c2ccc(o2)/C=C(\C(=O)O)/C#N)/C1=O)c1ccc(cc1)N(c1ccccc1)c1ccccc1	dichloromethane	23	10.1021/am300925e
CCCCCCCCN1c2cc(ccc2/C(=C\2/c3ccc(cc3N(C2=O)CCCCCCCC)c2ccc(cc2)/C=C(/C(=O)O)\C#N)/C1=O)c1ccc(cc1)N(c1ccccc1)c1ccccc1	dichloromethane	20	10.1021/am300925e
CCCCCCCCN1c2cc(ccc2/C(=C/2\C(=O)N(c3c2ccc(c3)c2ccc(s2)/C=C(\C(=O)O)/C#N)CCCCCCCC)/C1=O)c1ccc(cc1)N(c1ccc(cc1)OC)c1ccc(cc1)	dichloromethane	29	10.1021/am300925e

SMILES	SOLVENT	SHIFT (nm)	DOI
OC			
CCCCCCCCN1c2cc(ccc2/C(=C/2\C(=O)N(c3c2ccc(c3)c2ccc(o2)/C=C(\C(=O)O)/C#N)CCCCCCCC)/C1=O)c1ccc(cc1)N(c1ccc(cc1)OC)c1ccc(cc1)OC	dichloromethane	29	10.1021/am300925e
CCCCCCCCN1c2cc(ccc2/C(=C/2\C(=O)N(c3c2ccc(c3)c2ccc(cc2)/C=C(/C(=O)O)\C#N)CCCCCCCC)/C1=O)c1ccc(cc1)N(c1ccc(cc1)OC)c1ccc(cc1)OC	dichloromethane	28	10.1021/am300925e
CCCCCCOc1ccc(cc1)N(c1ccc(cc1)OCCCCCC)c1ccc(cc1)c1ccc(s1)c1ccc(s1)c1ccc(c2c1nsn2)/C=C(/C(=O)O)\C#N	dichloromethane	30	10.1002/adfm.201102519
CCCCCCOc1ccc(cc1)N(c1ccc(cc1)OCCCCCC)c1ccc(cc1)c1ccc(s1)c1ccc(s1)c1ccc(c2c1nsn2)c1ccc(cc1)/C=C(/C(=O)O)\C#N	dichloromethane	9	10.1002/adfm.201102519
CCCCOc1ccc(c(c1)OCCCC)c1ccc(cc1)N(c1ccc(cc1)c1ccc(cc1OCCCC)OCCCC)c1ccc(cc1)c1ccc(s1)/C=C(/C(=O)O)\C#N	dichloromethane	46	10.1002/adfm.201002319
CCCCOc1cc(OCCCC)ccc1c1ccc(cc1)N(c1ccc(cc1)c1ccc(s1)/C=C(/C(=O)O)\C#N)c1ccc(cc1)c1ccc(s1)/C=C(/C(=O)O)\C#N	dichloromethane	53	10.1002/adfm.201002319
N#C/C(=C\c1ccc(cc1)/C=C/c1ccc(cc1)N(c1ccccc1)c1ccccc1)/C(=O)O	dichloromethane	15	10.1021/jp800953s
N#C/C(=C\c1cc(Br)c(cc1Br)/C=C/c1ccc(cc1)N(c1ccccc1)c1ccccc1)/C(=O)O	dichloromethane	16	10.1021/jp800953s
N#C/C(=C\c1cc(Cl)c(cc1Cl)/C=C/c1ccc(cc1)N(c1ccccc1)c1ccccc1)/C(=O)O	dichloromethane	16	10.1021/jp800953s
N#C/C(=C\c1cc(c2cccs2)c(cc1c1cccs1)/C=C/c1ccc(cc1)N(c1ccccc1)c1ccccc1)/C(=O)O	dichloromethane	13	10.1021/jp800953s
N#C/C(=C\c1cc(C#N)c(cc1C#N)/C=C/c1ccc(cc1)N(c1ccccc1)c1ccccc1)/C(=O)O	dichloromethane	20	10.1021/jp800953s
OC(=O)CN1C(=S)S/C(=C/c2ccc(cc2)/C=C/c2ccc(cc2)N(c2ccccc2)c2ccccc2)/C1=O	dichloromethane	10	10.1021/jp800953s
OC(=O)CN1C(=S)S/C(=C/c2cc(Br)c(cc2Br)/C=C/c2ccc(cc2)N(c2ccccc2)c2ccccc2)/C1=O	dichloromethane	9	10.1021/jp800953s
OC(=O)CN1C(=S)S/C(=C/c2cc(Cl)c(cc2Cl)/C=C/c2ccc(cc2)N(c2ccccc2)c2ccccc2)/C1=O	dichloromethane	12	10.1021/jp800953s
OC(=O)CN1C(=S)S/C(=C/c2cc(c3cccs3)c(cc2c2cccs2)/C=C/c2ccc(cc2)N(c2ccccc2)c2ccccc2)/C1=O	dichloromethane	9	10.1021/jp800953s
N#C/C(=C\c1ccc(s1)c1ccc(s1)c1ccc(s1)/	dichloromethane	2	10.1021/jp800953s

SMILES	SOLVENT	SHIFT (nm)	DOI
C=C/c1ccc(cc1)N(c1ccccc1)c1ccccc1)/C(=O)O			
N#C/C(=C\c1cc2c(s1)c1c(s2)cc(s1)/C=C/c1ccc(cc1)N(c1ccccc1)c1ccccc1)/C(=O)O	dichloromethane	42	10.1021/jp800953s
OC(=O)C[C@@H]1C(=S)S/C(=C/c2ccc(cc2)N(c2ccccc2)c2ccccc2)/C1=O	dichloromethane	6	10.1002/adfm.200800516
OC(=O)CN1C(=S)S/C(=C/c2ccc(cc2)N(c2ccccc2)c2ccc(cc2)/C=C/c2ccc(cc2)N(c2ccccc2)c2ccccc2)/C1=O	dichloromethane	1	10.1002/adfm.200800516
CCCCCCCCn1c2ccc(cc2c2c1ccc(c2)c1ccc(s1)/C=C(/C(=O)O)\C#N)c1ccc(cc1)N(c1ccccc1)c1ccccc1	dichloromethane	-21	10.1016/j.dyepig.2013.12.025
CCCCCCCCn1c2ccc(cc2c2c1ccc(c2)c1ccc(o1)/C=C(/C(=O)O)\C#N)c1ccc(cc1)N(c1ccccc1)c1ccccc1	dichloromethane	11	10.1016/j.dyepig.2013.12.025
CCCCCCn1c2ccc(cc2c2c1ccc(c2)c1ccc(o1)/C=C(/C(=O)O)\C#N)c1ccc(cc1)N(c1ccccc1)c1ccccc1	dichloromethane	-20	10.1016/j.dyepig.2013.12.025
N#C/C(=C\c1ccc(o1)c1ccc2c(c1)c1cc(ccc1n2CCCC(C)C)c1ccc(cc1)N(c1ccccc1)c1ccccc1)/C(=O)O	dichloromethane	23	10.1016/j.dyepig.2013.12.025
CCCCCCc1nc(sc1c1ccc(cc1)N(c1ccccc1)c1ccccc1)c1sc(c(n1)CCCCCC)c1ccc(s1)/C=C(/C(=O)O)\C#N	dichloromethane	-14	10.1039/c0jm03811c
CCCCCCc1nc(sc1C#Cc1ccc(cc1)N(c1ccccc1)c1ccccc1)c1nc(c(s1)c1ccc(s1)/C=C(/C(=O)O)\C#N)CCCCCC	dichloromethane	-9	10.1039/c0jm03811c
CCCCCCc1nc(sc1c1ccc(cc1)N(c1ccc(cc1)OC)c1ccc(cc1)OC)c1nc(c(s1)c1ccc(s1)/C=C(/C(=O)O)\C#N)CCCCCC	dichloromethane	-7	10.1039/c0jm03811c
CCCCCCc1nc(sc1c1ccc(cc1)N(c1ccc(cc1)OC)c1ccc(cc1)OC)c1nc(c(s1)c1ccc(cc1)/C=C(/C(=O)O)\C#N)CCCCCC	dichloromethane	-31	10.1039/c0jm03811c
CCCCCCc1nc(sc1c1ccc(cc1)N(c1ccc(cc1)OC)c1ccc(cc1)OC)c1sc(c(n1)CCCCCC)C#Cc1ccc(cc1)/C=C(/C(=O)O)\C#N	dichloromethane	-22	10.1039/c0jm03811c
CCCCCCOc1c(OCCCCCC)c(c2ccc(s2)/C=C(/C(=O)O)\C#N)c2c(c1c1ccc(s1)c1ccc(cc1)N(c1ccccc1)c1ccccc1)nc(c(n2)C)C	dichloromethane	-18	10.1016/j.dyepig.2013.03.015
CCCCCCOc1c(OCCCCCC)c(c2ccc(o2)/C=C(/C(=O)O)\C#N)c2c(c1c1ccc(o1)c1ccc(cc1)N(c1ccccc1)c1ccccc1)nc(c(n2)C)C	dichloromethane	-46	10.1016/j.dyepig.2013.03.015

SMILES	SOLVENT	SHIFT (nm)	DOI
N#C/C(=C\c1ccc(s1)c1ccc(s1)c1ccc(cc1)N(c1ccccc1)c1ccccc1)/C(=O)O	dichloromethane	-10	10.1016/j.dyepig.2013.03.015
CCCCCCOc1c(OCCCCCC)c(c2sc(c(c2)CCCCCC)/C=C(/C(=O)O)\C#N)c2c(c1c1sc(c(c1)CCCCCC)c1ccc(cc1)N(c1ccccc1)c1ccccc1)nc1c(n2)c2ccccc2c2c1cccc2	dichloromethane	-16	10.1039/c2jm33833e
CCCCCCOc1c(OCCCCCC)c(c2sc(c(c2)CCCCCC)/C=C(/C(=O)O)\C#N)c2c(c1c1sc(c(c1)CCCCCC)c1ccc(cc1)N(c1ccc(cc1)C)c1ccc(cc1)C)nc1c(n2)c2ccccc2c2c1cccc2	dichloromethane	-12	10.1039/c2jm33833e
CCCCCCc1cc(sc1c1ccc(cc1)N(c1ccc(cc1)OC)c1ccc(cc1)OC)c1c(OCCCCCC)c(OCCCCCC)c(c2c1nc1c3ccccc3c3c(c1n2)cccc3)c1sc(c(c1)CCCCCC)/C=C(/C(=O)O)\C#N	dichloromethane	-10	10.1039/c2jm33833e
CCCCCCCCn1c2ccc(cc2c2c1c1c3ccccc3n(c1c1c(c3c2n(CCCCCCCC)c2c3cccc2)n(c2c1cccc2)CCCCCCCC)CCCCCCCC)c1ccc(s1)/C=C(/C(=O)O)\C#N	dichloromethane	20	10.1016/j.dyepig.2015.05.015
CCCCCCCCn1c2ccc(cc2c2c1c1c3ccccc3n(c1c1c(c3c2n(CCCCCCCC)c2c3cccc2)n(c2c1cccc2)CCCCCCCC)CCCCCCCC)c1ccc(s1)c1ccc(s1)/C=C(/C(=O)O)\C#N	dichloromethane	14	10.1016/j.dyepig.2015.05.015
CCCCCCCCc1cc(sc1c1ccc(s1)c1sc(cc1CCCCCCCC)/C=C(\C(=O)O)/C#N)c1ccc2c(c1)c1nc3ccccc3nc1n2CC(CCCC)CC	dichloromethane	5	10.1016/j.jpowsour.2015.01.148
CCCCC(Cn1c2ccc(cc2c2c1ccc(c2)c1ccc(s1)/C=C(\C(=O)O)/C#N)c1ccc2c(c1)c1nc3ccccc3nc1n2CC(CCCC)CC)CC	dichloromethane	25	10.1016/j.jpowsour.2015.01.148
CCCCC(Cn1c2ccc(cc2c2c1ccc(c2)c1ccc(o1)/C=C(\C(=O)O)/C#N)c1ccc2c(c1)c1nc3ccccc3nc1n2CC(CCCC)CC)CC	dichloromethane	15	10.1016/j.jpowsour.2015.01.148
CCCCCCn1c(/C=C/c2ccc(cc2)N(c2ccccc2)c2ccccc2)ccc1/C=C(/C(=O)O)\C#N	dichloromethane	-19	10.1021/am403668d
N#C/C(=C\c1ccc(n1CCCCCCOc1ccc(cc1)C(=O)OC)/C=C/c1ccc(cc1)N(c1ccccc1)c1ccccc1)/C(=O)O	dichloromethane	-18	10.1021/am403668d
N#C/C(=C\	dichloromethane	-15	10.1021/am403668d

SMILES	SOLVENT	SHIFT (nm)	DOI
c1ccc(n1CCCCCCOc1ccc(cc1)C(=O)OC(C)(C)C)/C=C/c1ccc(cc1)N(c1ccccc1)c1ccccc1)/C(=O)O			
COC(=O)c1cc(OCCCCCCn2c(/C=C/c3ccc(cc3)N(c3ccccc3)c3ccccc3)ccc2/C=C(/C(=O)O)\C#N)cc(c1)OCCCCCCn1c(/C=C/c2ccc(cc2)N(c2ccccc2)c2ccccc2)ccc1/C=C(/C(=O)O)\C#N	dichloromethane	3	10.1021/am403668d
C1CCN(CC1)c1ccc(c2c1nsn2)/C=C/c1ccncc1	dichloromethane	14	10.1016/j.tet.2015.04.089
O1CCN(CC1)c1ccc(c2c1nsn2)/C=C/c1ccncc1	dichloromethane	3	10.1016/j.tet.2015.04.089
n1ccc(cc1)/C=C/c1ccc(c2c1nsn2)/C=C/c1ccncc1	dichloromethane	-16	10.1016/j.tet.2015.04.089
CCCCn1c2ccc(cc2c2c1cccc2)/C=C/c1ccc(c2c1nsn2)/C=C/c1ccncc1	dichloromethane	1	10.1016/j.tet.2015.04.089
CCCCN1c2ccc(cc2Sc2c1cccc2)/C=C/c1ccc(c2c1nsn2)/C=C/c1ccncc1	dichloromethane	1	10.1016/j.tet.2015.04.089
CCCCOc1cc(OCCCC)ccc1c1ccc(cc1)N(c1ccc(cc1)c1ccc(cc1OCCCC)OCCCC)c1ccc(cc1)c1ccc2c(c1)Oc1c(N2CC(CCCC)CC)ccc(c1)/C=C(/C(=O)O)\C#N	dichloromethane	7	10.1016/j.dyepig.2018.10.066
CCCCC(CN1c2ccc(cc2Oc2c1ccc(c2)c1ccc(s1)/C=C(/C(=O)O)\C#N)c1ccc(cc1)N(c1ccc(cc1)c1ccc(cc1OCCCC)OCCCC)c1ccc(cc1)c1ccc(cc1OCCCC)OCCCC)CC	dichloromethane	13	10.1016/j.dyepig.2018.10.066
CCCCCCOc1ccc(cc1)N(c1ccc(cc1)OCCCCCC)c1ccc(cc1)c1ccc(s1)c1ccc(c2c1nsn2)c1ccc(n1C)C=C(C(=O)O)C#N	dichloromethane	-14	10.1039/C8TA06258G
CCCCCCOc1ccc(cc1)N(c1ccc(cc1)OCCCCCC)c1ccc(cc1)c1ccc(s1)c1ccc(c2c1nsn2)c1ccc(n1CCCCCC)C=C(C(=O)O)C#N	dichloromethane	-14	10.1039/C8TA06258G
CCCCCCCCn1c(ccc1c1ccc(c2c1nsn2)c1ccc(s1)c1ccc(cc1)N(c1ccc(cc1)OCCCCCC)c1ccc(cc1)OCCCCCC)C=C(C(=O)O)C#N	dichloromethane	-3	10.1039/C8TA06258G
CCCCCCCCCCn1c(ccc1c1ccc(c2c1nsn2)c1ccc(s1)c1ccc(cc1)N(c1ccc(cc1)OCCCCCC)c1ccc(cc1)OCCCCCC)C=C(C(=O)O)C#N	dichloromethane	-3	10.1039/C8TA06258G
CCCCCCCCCCCCn1c(ccc1c1ccc(c2c1nsn2)c1ccc(s1)c1ccc(cc1)N(c1ccc(cc1)OCCCCCC)c1ccc(cc1)OCCCCCC)C=C(C(=O)O)C#N	dichloromethane	-3	10.1039/C8TA06258G
CCCC[C@@H](CN1c2ccc(cc2Sc2c1cccc2)/C=C(/C(=O)O)\C#N)CC	dichloromethane	29	10.1021/ef900207y
CCCC[C@@H](CN1c2ccc(cc2Sc2c1ccc(c2)/	dichloromethane	10	10.1021/ef900207y

SMILES	SOLVENT	SHIFT (nm)	DOI
C=C(/C(=O)O)\C#N)/C=C(/C(=O)O)\C#N)CC			
CCCC[C@@H](CN1c2ccc(cc2Sc2c1cccc2)/C=C\1/SC(=S)N(C1=O)CC(=O)O)CC	dichloromethane	14	10.1021/ef900207y
CCCC[C@@H](CN1c2ccc(cc2Sc2c1ccc(c2)/C=C\1/SC(=S)N(C1=O)CC(=O)O)/C=C/1\SC(=S)N(C1=O)CC(=O)O)CC	dichloromethane	9	10.1021/ef900207y
CCCCCCN1c2ccc(cc2Sc2c1ccc(c2)C=C(C(=O)O)C#N)c1ccc2c(c1)c1ccccc1n2CCCCCC	dichloromethane	15	10.1016/j.dyepig.2014.05.015
CCCCCCN1c2ccc(cc2Sc2c1ccc(c2)C=C(C(=O)O)C#N)N(c1ccc(cc1)C(c1ccccc1)(C)C)c1ccc(cc1)C(c1ccccc1)(C)C	dichloromethane	18	10.1016/j.dyepig.2014.05.015
CCCCCCN1c2ccc(cc2Sc2c1ccc(c2)C=C(C(=O)O)C#N)c1ccc(cc1)N(c1ccc2c(c1)C(CCC)(CCC)c1c2cccc1)c1ccc2c(c1)C(CCC)(CCC)c1c2cccc1	dichloromethane	9	10.1016/j.dyepig.2014.05.015
CCCCCCCCCCCCN1c2ccc(cc2Oc2c1cccc2)/C=C/c1ccc(s1)/C=c/1\s/c(=C\2/SC(=S)N(C2=O)CCCCCCCC)/n(c1=O)CC(=O)O	dichloromethane	13	10.1039/b901238a
CCCCCCOc1ccc(cc1)N(c1ccc2c(c1)c1ccccc1n2CCCCCC)c1ccc2c(c1)Sc1c(N2CCCCCC)ccc(c1)/C=C(/C(=O)O)\C#N	dichloromethane	40	10.1016/j.synthmet.2016.01.025
CCCCCCN1c2ccc(cc2Sc2c1ccc(c2)/C=C(/C(=O)O)\C#N)N(c1ccc2c(c1)c1ccccc1n2CCCCCC)c1ccc2c(c1)c1ccccc1n2CCCCCC	dichloromethane	40	10.1016/j.synthmet.2016.01.025
CCCCCCN1c2ccc(cc2Sc2c1ccc(c2)/C=C(/C(=O)O)\C#N)N(c1ccc2c(c1)c1ccccc1n2CCCCCC)c1ccc2c(c1)scc2	dichloromethane	40	10.1016/j.synthmet.2016.01.025
CCCCCCN1c2ccc(cc2Sc2c1ccc(c2)/C=C(/C(=O)O)\C#N)c1cc2c(s1)c1c(n2CCCCCC)ccs1	dichloromethane	18	10.1016/j.solener.2017.08.012
CCCCCCOc1ccc(cc1)n1c2cc(sc2c2c1ccs2)c1ccc2c(c1)Sc1c(N2CCCCCC)ccc(c1)/C=C(/C(=O)O)\C#N	dichloromethane	23	10.1016/j.solener.2017.08.012
CCCCCCOc1cc(OCCCCCC)ccc1n1c2cc(sc2c2c1ccs2)c1ccc2c(c1)Sc1c(N2CCCCCC)ccc(c1)/C=C(/C(=O)O)\C#N	dichloromethane	28	10.1016/j.solener.2017.08.012
CCCCCCOc1cc(OCCCCCC)ccc1n1c2cc(sc2c2c1cc(s2)/C=C(/C(=O)O)\C#N)c1ccc2c(c1)Sc1c(N2CCCCCC)cccc1	dichloromethane	38	10.1016/j.solener.2017.08.012
COc1ccc(cc1)c1nc2c(ncc(c2nc1c1ccc(cc1)OC)c1ccc(s1)C=C(C(=O)O)C#N)c1ccc(cc1)N(c	dichloromethane	21	10.1039/C3SC51844B

SMILES	SOLVENT	SHIFT (nm)	DOI
1ccccc1)c1ccccc1			
COc1ccc(cc1)c1nc2c(ncc(c2nc1c1ccc(cc1)OC)c1ccc(o1)C=C(C(=O)O)C#N)c1ccc(cc1)N(c1ccccc1)c1ccccc1	dichloromethane	15	10.1039/C3SC51844B
COc1ccc(cc1)c1nc2c(ncc(c2nc1c1ccc(cc1)OC)c1ccc(cc1)C=C(C(=O)O)C#N)c1ccc(cc1)N(c1ccccc1)c1ccccc1	dichloromethane	7	10.1039/C3SC51844B
CCCCCCCCOc1ccc(cc1)N(c1ccc(cc1)OCCCCCCCC)c1ccc(cc1)c1ncc(c2c1nc(c1ccc(cc1)OC)c(n2)c1ccc(cc1)OC)c1ccc(s1)C=C(C(=O)O)C#N	dichloromethane	15	10.1039/C3SC51844B
N#CC(=Cc1ccc(s1)c1cnc(c2c1nccn2)c1ccc(cc1)N(c1ccccc1)c1ccccc1)C(=O)O	dichloromethane	8	10.1039/C3SC51844B
CCCCCCOc1ccc(cc1)n1c2cc(sc2c2c1cc(s2)C=C(C(=O)O)C#N)c1ccc2c(c1)C1CCCC1N2c1ccc(cc1)C	dichloromethane	88	10.1039/C5SC02778K
CCCCCCOc1ccc(cc1)n1c2cc(sc2c2c1cc(s2)C=C(C(=O)O)C#N)c1sc(c2c1OCCO2)c1ccc2c(c1)C1CCCC1N2c1ccc(cc1)C	dichloromethane	78	10.1039/C5SC02778K
CCCCCCOc1ccc(cc1)n1c2cc(sc2c2c1cc(s2)C=C(C(=O)O)C#N)c1ccc(c2c1nsn2)c1ccc2c(c1)C1CCCC1N2c1ccc(cc1)C	dichloromethane	33	10.1039/C5SC02778K
CCCCCCOc1ccc(cc1)n1c2cc(sc2c2c1cc(s2)C=C(C(=O)O)C#N)c1ccc(c2c1non2)c1ccc2c(c1)C1CCCC1N2c1ccc(cc1)C	dichloromethane	13	10.1039/C5SC02778K
CCCCCCOc1ccc(cc1)N(c1ccc(cc1)OCCCCCC)c1ccc(cc1)c1cc2c(s1)c1c(n2CCCCCC)c2c(s1)c1c(n2CCCCCC)cc(s1)C=C(C(=O)O)C#N	dichloromethane	87	10.1039/C4TA06705C
CCCCCCOc1ccc(cc1)n1c2cc(sc2c2c1c1c(s2)c2c(n1c1ccc(cc1)OCCCCCC)cc(s2)C=C(C(=O)O)C#N)c1ccc(cc1)N(c1ccc(cc1)OCCCCCC)c1ccc(cc1)OCCCCCC	dichloromethane	70	10.1039/C4TA06705C
CCCCCCOc1ccc(cc1)N(c1ccc(cc1)OCCCCCC)c1ccc(cc1)c1cc2c(s1)c1c(n2CCCCCC)cc(s1)C=C(C(=O)O)C#N	dichloromethane	70	10.1039/C4TA06705C
CCCCCCCCOc1ccc(c(c1)OCCCCCCCC)c1cc(ccc1N(c1ccc2c(c1)C(C)(C)c1c2sc(c1)c1cnc(c2c1nc(c1ccccc1)c(n2)c1ccccc1)c1sc2c(c1)C(c1c2sc(c1)/C=C(/C(=O)O)\C#N)(CC(CCCC)CC)CC(CCCC)CC)c1ccc(cc1)c1ccc(cc1OCCCCCCCC)OCCCCCCCC)c1ccc(cc1OCCCCCCCC)OCCCCCCCC	dichloromethane	24	10.1039/C6TA09723E
CCCCCCCCOc1ccc(c(c1)OCCCCCCCC)c1cc(cc	dichloromethane	26	10.1039/C6TA09723E

SMILES	SOLVENT	SHIFT (nm)	DOI
c1N(c1ccc2c(c1)C(C)(C)c1c2sc(c1)c1ccc(c2c1nc(c1ccccc1)c(n2)c1ccccc1)c1sc2c(c1)C(c1c2sc(c1)/C=C(/C(=O)O)\C#N)(CC(CCCC)CC)CC(CCCC)CC)c1ccc(cc1)c1ccc(cc1OCCCCCCCC)OCCCCCCCC)c1ccc(cc1OCCCCCCCC)OCCCCCCCC			
CCCCCCC1(CCCCCC)c2cc(sc2c2c1cc(s2)c1ccc(cc1)N(c1ccc(cc1)c1ccc(cc1OCCCC)OCCCC)c1ccc(cc1)c1ccc(cc1OCCCC)OCCCC)/C=C(/C(=O)O)\C#N	dichloromethane	70	10.1039/C4TA05774K
CCCCC(COc1cc(OCC(CCCC)CC)ccc1c1ccc(cc1)N(c1ccc(cc1)c1cc(OCCC(CCC)CC)cc(c1)OCC(CCCC)CC)c1ccc(cc1)c1ccc(s1)c1ccc(c2c1nc(c1ccccc1)c(n2)c1ccccc1)c1ccc(cc1)/C=C(/C(=O)O)\C#N)CC	dichloromethane	-3	10.1039/C5TA07254A
CCCCC(COc1cc(OCC(CCCC)CC)ccc1c1ccc(cc1)N(c1ccc(cc1)c1cc(OCCC(CCC)CC)cc(c1)OCC(CCCC)CC)c1ccc(cc1)c1sc(c2c1OCCO2)c1ccc(c2c1nc(c1ccccc1)c(n2)c1ccccc1)c1ccc(cc1)/C=C(/C(=O)O)\C#N)CC	dichloromethane	2	10.1039/C5TA07254A
CCCCC(COc1cc(OCC(CCCC)CC)ccc1c1ccc(cc1)N(c1ccc(cc1)c1cc(OCCC(CCC)CC)cc(c1)OCC(CCCC)CC)c1ccc(cc1)c1sc2c(c1)C(c1c2sc(c1)c1ccc(c2c1nc(c1ccccc1)c(n2)c1ccccc1)c1ccc(cc1)/C=C(/C(=O)O)\C#N)(CC(CCCC)CC)CC(CCCC)CC)CC	dichloromethane	17	10.1039/C5TA07254A
N#CC(=C1c2ccc(cc2c2c1ccc(c2)N(c1ccccc1)c1ccccc1)N(c1ccccc1)c1ccccc1)C(=O)O	dichloromethane	22	10.1039/C4TA02161D
CCCCC(C[Si]1(CC(CCCC)CC)c2cc(sc2c2c1cc(s2)/C=C(/C(=O)O)\C#N)c1ccc(c2c1nsn2)c1ccc2c(c1)C1CCCC1N2c1ccc(cc1)C)CC	dichloromethane	19	10.1039/C6TA05588E
CCCCCCCCCCCCOc1ccc(cc1)c1nc2c(ncc(c2nc1c1ccc(cc1)OCCCCCCCCCCCC)c1sc2c(c1)[Si](c1c2sc(c1)/C=C(/C(=O)O)\C#N)(CC(CCCC)CC)CC(CCCC)CC)c1ccc2c(c1)C1CCCC1N2c1ccc(cc1)C	dichloromethane	19	10.1039/C6TA05588E
CCCCCCCCCCCCc1ccc2c(c1)c1cc(CCCCCCCCCCCC)ccc1c1c2nc2c(n1)c(cnc2c1ccc2c(c1)C1CCCC1N2c1ccc(cc1)C)c1sc2c(c1)[Si](c1c2sc(c1)/C=C(/C(=O)O)\C#N)(CC(CCCC)CC)CC(CCCC)CC	dichloromethane	7	10.1039/C6TA05588E
N#CC(=Cc1ccc(s1)c1ccc2c(=c3sc(cc3c3c4OCCCCCCOc5c2c(OCCCCCCOc3ccc4)ccc5)c2cc	dichloromethane	55	10.1039/C3TA12368E

SMILES	SOLVENT	SHIFT (nm)	DOI
c(cc2)N(c2ccccc2)c2ccccc2)s1)C(=O)O			
N#CC(=Cc1ccc(s1)c1cc2c(=c3sc(cc3c3c4OCCCCCCOc5c2c(OCCCCCCOc3ccc4)ccc5)c2ccc3c(c2)n(CC)c2c3cccc2)s1)C(=O)O	dichloromethane	54	10.1039/C3TA12368E
N#CC(=Cc1ccc(s1)c1sc2=c3sc(cc3c3c4OCCCCCCOc5c(c2c1)c(OCCCCCCOc3ccc4)ccc5)c1ccc2c(c1)n(c1ccccc1)c1c2cccc1)C(=O)O	dichloromethane	60	10.1039/C3TA12368E
COc1ccc(cc1)N(c1ccc(cc1)OC)c1ccc(cc1)c1sc2=c3sc(cc3c3c4OCCCCCCOc5c(c2c1)c(OCCCCCCOc3ccc4)ccc5)c1ccc(s1)C=C(C(=O)O)C#N	dichloromethane	50	10.1039/C3TA12368E
CCCCCCOc1ccc(cc1)N(c1ccc(cc1)OCCCCCC)c1ccc(cc1)c1ccc(c2c1nsn2)c1ccc(s1)/C=C(/C(=O)O)\C#N	dichloromethane	21	10.1039/C5TA02120K
CCCCCCOc1ccc(cc1)N(c1ccc(cc1)OCCCCCC)c1ccc(cc1)c1ccc(c2c1nsn2)c1sc(c2c1OCCO2)c1ccc(s1)/C=C(/C(=O)O)\C#N	dichloromethane	-4	10.1039/C5TA02120K
CCCCCCc1cc(sc1c1ccc(s1)/C=C(/C(=O)O)\C#N)c1ccc(c2c1nsn2)c1ccc(cc1)N(c1ccc(cc1)OCCCCCC)c1ccc(cc1)OCCCCCC	dichloromethane	43	10.1039/C5TA02120K
CCCCCCOc1ccc(cc1)c1nc2c(c3ccc(cc3)N(c3ccc(cc3)C(C)(C)C)c3ccc(cc3)C(C)(C)C)c3nc(c4ccc(cc4)OCCCCCC)c(nc3c(c2nc1c1ccc(cc1)OCCCCCC)c1ccc(cc1)/C=C(/C(=O)O)\C#N)c1ccc(cc1)OCCCCCC	dichloromethane	10	10.1039/C4TA02499K
CCCCCCCCOc1ccc(cc1)c1nc2c(nc1c1ccc(cc1)OCCCCCCCC)c(c1ccc(cc1)N(c1ccc(cc1)C(C)(C)C)c1ccc(cc1)C(C)(C)C)c1c(c2c2ccc(s2)/C=C(/C(=O)O)\C#N)nc(c(n1)c1ccc(cc1)OCCCCCCCC)c1ccc(cc1)OCCCCCCCC	dichloromethane	-26	10.1039/C4TA02499K
CCCCCC(n1c(c2ccc(cc2)c2ccc3c(c2)n(C(CCCCC)CC)c2c3c(c3ccccc3)c3c(c2c2ccccc2)c2c(n3C(CCCCC)CC)cccc2)c2c(c1=O)c(n(c2=O)C(CCCCC)CC)c1ccc(cc1)c1ccc(s1)/C=C(/C(=O)O)\C#N)CC	dichloromethane	7	10.1039/C4TA05162A
CCCCCC(n1c(c2ccc(cc2)c2ccc3c(c2)n(C(CCCCC)CC)c2c3c(c3ccccc3)c3c(c2c2ccccc2)c2c(n3C(CCCCC)CC)cccc2)c2c(c1=O)c(n(c2=O)C(CCCCC)CC)c1ccc(cc1)c1ccc(cc1)/C=C(/C(=O)O)\C#N)CC	dichloromethane	-3	10.1039/C4TA05162A
CCCCCCCCN1c2ccc(cc2Sc2c1ccc(c2)c1ccc2c(c1)C1(c3c2cccc3)c2ccccc2c2c1cccc2)c1ccc(c2c1nsn2)c1ccc(cc1)C(=O)O	dichloromethane	42	10.1039/C8NJ04164D

SMILES	SOLVENT	SHIFT (nm)	DOI
CCCCCCCCN1c2ccc(cc2Sc2c1ccc(c2)c1ccc2c(c1)C1(c3c2cccc3)c2ccccc2c2c1cccc2)c1ccc(c2c1nsn2)c1ccc(s1)C=C(C(=O)O)C#N	dichloromethane	10	10.1039/C8NJ04164D
CCCCCCCCN1c2ccc(cc2Sc2c1ccc(c2)c1ccc(c2c1nsn2)c1ccncc1)c1ccc2c(c1)C1(c3c2cccc3)c2ccccc2c2c1cccc2	dichloromethane	4	10.1039/C8NJ04164D
N#C/C(=C\c1ccc(s1)/C=C/c1ccc(cc1)/C=C/c1ccc(cc1)N(c1cccc2c1cccc2)c1ccccc1)/C(=O)O	dichloromethane	-24	10.1039/C7NJ00413C
CCCCCCOc1ccc(cc1)N(c1ccc(cc1)OCCCCCC)c1ccc(cc1)/C=C/c1ccc(cc1)/C=C/c1ccc(s1)/C=C(/C(=O)O)\C#N	dichloromethane	-10	10.1039/C7NJ00413C
N#C/C(=C\c1ccc(cc1)/C=C/c1ccc(cc1)/C=C/c1ccc(cc1)N(c1cccc2c1cccc2)c1ccccc1)/C(=O)O	dichloromethane	-36	10.1039/C7NJ00413C
CCCCCCOc1ccc(cc1)N(c1ccc(cc1)OCCCCCC)c1ccc(cc1)/C=C/c1ccc(cc1)/C=C/c1ccc(cc1)/C=C(/C(=O)O)\C#N	dichloromethane	-25	10.1039/C7NJ00413C
N#C/C(=C\c1ccc(s1)C#Cc1ccc(cc1)C#Cc1ccc(cc1)N(c1cccc2c1cccc2)c1ccccc1)/C(=O)O	dichloromethane	-24	10.1039/C7NJ00413C
CCCCCCOc1ccc(cc1)N(c1ccc(cc1)OCCCCCC)c1ccc(cc1)C#Cc1ccc(cc1)C#Cc1ccc(s1)/C=C(/C(=O)O)\C#N	dichloromethane	-27	10.1039/C7NJ00413C
N#C/C(=C\c1ccc(cc1)C#Cc1ccc(cc1)C#Cc1ccc(cc1)N(c1cccc2c1cccc2)c1ccccc1)/C(=O)O	dichloromethane	-25	10.1039/C7NJ00413C
CCCCCCOc1ccc(cc1)N(c1ccc(cc1)OCCCCCC)c1ccc(cc1)C#Cc1ccc(cc1)C#Cc1ccc(cc1)/C=C(/C(=O)O)\C#N	dichloromethane	-30	10.1039/C7NJ00413C
N#CC(=c1[nH]c(=O)c(=Cc2ccc(cc2)N(c2ccc(cc2)c2cccs2)c2ccc(cc2)c2cccs2)s1)C#N	dichloromethane	60	10.1039/C4RA13782E
N#CC(=c1sc(=Cc2ccc(cc2)N(c2ccc(cc2)c2cc(cc3c2[nH]c2c3cc(cc2)C(C)(C)C)C(C)(C)C)c2ccc(cc2)c2cc(cc3c2[nH]c2c3cc(cc2)C(C)(C)C)C(C)(C)C)c(=O)[nH]1)C#N	dichloromethane	29	10.1039/C4RA13782E
CCCCCCc1ccc(s1)c1ccc(cc1)N(c1ccc(cc1)c1ccc(s1)CCCCCC)c1ccc(cc1)c1ccc(s1)C=c1sc(=C(C#N)C#N)[nH]c1=O	dichloromethane	66	10.1039/C4RA13782E
CCCCCCOc1ccc(cc1)N1c2ccc(cc2Sc2c1cccc2)/C=C/C1C/C(=C(/C(=O)O)\C#N)/CC(C1)(C)C	dichloromethane	55	10.1039/C5RA17898C
N#C/C(=C/1\CC(/C=C/c2ccc(cc2)N(c2ccccc2)c2ccccc2)CC(C1)(C)C)/C(=O)O	dichloromethane	62	10.1039/C5RA17898C

SMILES	SOLVENT	SHIFT (nm)	DOI
N#C/C(=C/1\CC(/C=C/c2ccc(cc2)N(c2cccc3c2cccc3)c2ccccc2)CC(C1)(C)C)/C(=O)O	dichloromethane	71	10.1039/C5RA17898C
CCCCCCOc1ccc(cc1)N(c1ccc(cc1)OCCCCCC)c1ccc(cc1)/C=C/C1C/C(=C(/C(=O)O)\C#N)/CC(C1)(C)C	dichloromethane	70	10.1039/C5RA17898C
CCCCCCc1cc(sc1c1ccc(cc1)N(c1ccc(cc1)OCCCC)c1ccc(cc1)OCCCC)c1sc(cc1CCCCCC)/C=C\1/SC(=S)N(C1=O)CC(=O)O	dichloromethane	31	10.1039/C3RA45034A
CCCCCCc1cc(sc1c1ccc(cc1)N(c1ccc(cc1)OCCCC)c1ccc(cc1)OCCCC)c1sc(cc1CCCCCC)/C=C\1/SC(=S)N(C1=O)CCC(=O)O	dichloromethane	7	10.1039/C3RA45034A
N#C/C(=C\c1ccc(s1)c1ccc2c(c1)C(CC)(CC)c1c2c2c(c3c1c1ccc(cc1C3(CC)CC)N(c1ccccc1)c1ccccc1)c1c(C2(CC)CC)cc(cc1)N(c1ccccc1)c1ccccc1)/C(=O)O	dichloromethane	-16	10.1021/jp902408z
CCCCCCC1(CCCCCC)c2cc(ccc2c2c1c1c3ccc(cc3C(c1c1c2C(CCCCCC)(CCCCCC)c2c1ccc(c2)N(c1ccccc1)c1ccccc1)(CCCCCC)CCCCCC)N(c1ccccc1)c1ccccc1)c1ccc(s1)/C=C(/C(=O)O)\C#N	dichloromethane	-5	10.1021/jp902408z
CCCCCCC1(CCCCCC)c2cc(ccc2c2c1c1c3ccc(cc3C(c1c1c2C(CCCCCC)(CCCCCC)c2c1ccc(c2)N(c1ccc(cc1)OC)c1ccc(cc1)OC)(CCCCCC)CCCCCC)c1ccc(s1)/C=C(/C(=O)O)\C#N)N(c1ccc(cc1)OC)c1ccc(cc1)OC	dichloromethane	4	10.1021/jp902408z
CCCC1(CCC)c2cc(ccc2c2c1c1c3ccccc3Cc1c1c2C(CCC)(CCC)c2c1cccc2)N(c1ccc2c(c1)C(CCC)(CCC)c1c2c2Cc3c(c2c2c1c1ccccc1C2(CCC)CCC)cccc3)c1ccc(cc1)c1ccc(s1)/C=C(/C(=O)O)\C#N	dichloromethane	32	10.1021/jp107439d
CCCC1(CCC)c2cc(ccc2c2c1c1c3ccccc3Cc1c1c2C(CCC)(CCC)c2c1cccc2)N(c1ccc2c(c1)C(CCC)(CCC)c1c2c2Cc3c(c2c2c1c1ccccc1C2(CCC)CCC)cccc3)c1ccc(cc1)/C=C/c1ccc(s1)/C=C(/C(=O)O)\C#N	dichloromethane	52	10.1021/jp107439d
CCCC1(CCC)c2cc(ccc2c2c1c1c3ccccc3Cc1c1c2C(CCC)(CCC)c2c1cccc2)N(c1ccc2c(c1)C(CCC)(CCC)c1c2c2Cc3c(c2c2c1c1ccccc1C2(CCC)CCC)cccc3)c1ccc(cc1)c1sc(c2c1OCCO2)/C=C(/C(=O)O)\C#N	dichloromethane	42	10.1021/jp107439d
CCCCCCOc1cc(OCCCCCC)ccc1c1ccc(cc1)N(c	dichloromethane	53	10.1021/cm401144j

SMILES	SOLVENT	SHIFT (nm)	DOI
1ccc(cc1)c1ccc(cc1OCCCCCC)OCCCCCC)c1ccc(cc1)c1cc2c(s1)c1c(n2C(CCCCCC)CCCCCC)cc(s1)C=C(C(=O)O)C#N			
CCCCCCOc1cc(OCCCCCC)cc2c1c1ccc(cc1C2(C)C)N(c1ccc2c(c1)C(C)(C)c1c2c(OCCCCCC)cc(c1)OCCCCCC)c1ccc(cc1)c1cc2c(s1)c1c(n2C(CCCCCC)CCCCCC)cc(s1)C=C(C(=O)O)C#N	dichloromethane	56	10.1021/cm401144j
C(#N)C(C(=O)O)=CC=1SC(=CC1)C1=C(N=C(S1)C=1SC(=C(N1)CCCCCC)C=1SC(=CC1)C1=CC=C(C=C1)N(C1=CC=CC=C1)C1=CC=CC=C1)CCCCCC	dichloromethane	-13	10.1002/chem.201103702
C(#N)C(C(=O)O)=CC=1OC(=CC1)C1=C(N=C(S1)C=1SC(=C(N1)CCCCCC)C=1SC(=CC1)C1=CC=C(C=C1)N(C1=CC=CC=C1)C1=CC=CC=C1)CCCCCC	dichloromethane	-37	10.1002/chem.201103702
C(#N)C(C(=O)O)=CC1=CC=C(C=C1)C1=C(N=C(S1)C=1SC(=C(N1)CCCCCC)C=1SC(=CC1)C1=CC=C(C=C1)N(C1=CC=CC=C1)C1=CC=CC=C1)CCCCCC	dichloromethane	-1	10.1002/chem.201103702
C(#N)C(C(=O)O)=CC=1SC(=CC1)C1=CC=C(C=C1)C(=CC1=C(N=C(S1)C=1SC(=C(N1)CCCCCC)C1=CC=C(C=C1)N(C1=CC=CC=C1)C1=CC=CC=C1)CCCCCC)C#N	dichloromethane	-69	10.1002/chem.201103702
C(#N)C(C(=O)O)=CC=1OC(=CC1)C1=CC=C(C=C1)C(=CC1=C(N=C(S1)C=1SC(=C(N1)CCCCCC)C1=CC=C(C=C1)N(C1=CC=CC=C1)C1=CC=CC=C1)CCCCCC)C#N	dichloromethane	-54	10.1002/chem.201103702
C(#N)C(C(=O)O)=CC1=CC=C(C=C1)C1=CC=C(C=C1)C(=CC1=C(N=C(S1)C=1SC(=C(N1)CCCCCC)C1=CC=C(C=C1)N(C1=CC=CC=C1)C1=CC=CC=C1)CCCCCC)C#N	dichloromethane	-58	10.1002/chem.201103702
C(=O)(O)C[N+]1=CC=C(C=C1)\C=C\C=1C=C2C(CC(N(C2=CC1)C)(C)C)C	dichloromethane	-8	10.1002/chem.201200826
C(=O)(O)C[N+]1=CC=C(C=C1)\C=C\C=1C=C2C(CC(N(C2=CC1OC)C)(C)C)C	dichloromethane	-11	10.1002/chem.201200826
C(=O)(O)C[N+]1=CC=C(C=C1)\C=C\C=1C=C2C(CC(N(C2=CC1)C)(C)CCCCCC)CCCCCC	dichloromethane	-17	10.1002/chem.201200826
C(=O)(O)C[N+]1=CC=C(C=C1)\C=C\C=1C=C2C(CC(N(C2=CC1OC)C)(C)CCCCCC)CCCCCC	dichloromethane	-11	10.1002/chem.201200826
CCCCCCC1CC(C)(CCCCCC)N(c2c1cc(/C=C(\C(=O)O)/C#N)c(c2)OC)C	dichloromethane	-7	10.1002/chem.201200826

SMILES	SOLVENT	SHIFT (nm)	DOI
CC1=CC=C(C=C1)N1C2CCCC2C2=CC(=CC=C12)C1=C2N=C(C3=CC=CC=C3)C(=NC2=C(C=N1)C1=CC=C(C1)\C=C(\C#N)C(O)=O)C1=CC=CC=C1	dichloromethane	38	10.1002/chem.201503514
CCCCCCCCOC1=CC=C(C=C1)C1=NC2=C(C=NC(C3=CC=C4N(C5CCCC5C4=C3)C3=CC=C(C)C=C3)=C2N=C1C1=CC=C(OCCCCCCCC)C=C1)C1=CC=C(C1)\C=C(\C#N)C(O)=O	dichloromethane	38	10.1002/chem.201503514
COC1=CC=C(C=C1)C1=NC2=C(C=CC(C3=CC=C4N(C5CCCC5C4=C3)C3=CC=C(C)C=C3)=C2N=C1C1=CC=C(OC)C=C1)C1=CC=C(S1)\C=C(/C#N)C(O)=O	dichloromethane	13	10.1002/chem.201103542
CCCCCCCCOC1=CC=C(C=C1)C1=NC2=C(C=CC(C3=CC=C4N(C5CCCC5C4=C3)C3=CC=C(C)C=C3)=C2N=C1C1=CC=C(OCCCCCCCC)C=C1)C1=CC=C(S1)\C=C(/C#N)C(O)=O	dichloromethane	1	10.1002/chem.201103542
COC1=CC=C(C=C1)C1=NC2=C(C=CC(C3=CC=C(C=C3)N(C3=CC=CC=C3)C3=CC=CC=C3)=C2N=C1C1=CC=C(OC)C=C1)C1=CC=C(S1)\C=C(/C#N)C(O)=O	dichloromethane	3	10.1002/chem.201103542
CCCCCCCCOC1=CC=C(C=C1)C1=NC2=C(C=CC(C3=CC=C(C=C3)N(C3=CC=CC=C3)C3=CC=CC=C3)=C2N=C1C1=CC=C(OCCCCCCCC)C=C1)C1=CC=C(S1)\C=C(/C#N)C(O)=O	dichloromethane	3	10.1002/chem.201103542
C1(=CC=CC=C1)C(=C(C1=CC=CC=C1)C1=CC=CC=C1)C1=CC=C(C=C1)C1=CC=C(C=C1)N(C1=CC=C(C=CC2=CC=C(O2)/C=C(/C(=O)O)\C#N)C=C1)C1=CC=C(C=C1)C1=CC=C(C=C1)C(=C(C1=CC=CC=C1)C1=CC=CC=C1)C1=CC=CC=C1	dichloromethane	-5	10.1002/ejoc.201200530
C1(=CC=CC=C1)C(=C(C1=CC=CC=C1)C1=CC=CC=C1)C1=CC=C(C=C1)C1=CC=C(C=C1)N(C1=CC=C(C=CC2=CC=C(S2)/C=C(/C(=O)O)\C#N)C=C1)C1=CC=C(C=C1)C1=CC=C(C=C1)C(=C(C1=CC=CC=C1)C1=CC=CC=C1)C1=CC=CC=C1	dichloromethane	-9	10.1002/ejoc.201200530
CCOC1=CC(\C=C(\C#N)C(O)=O)=C(OCC)C=C1\C=C\C1=CC=C(C=C1)N(C1=CC=CC=C1)C1=CC=CC=C1	dichloromethane	24	10.1002/cjoc.201200091
CCCCCN1C2=CC=CC=C2OC2=CC(\C=C\C3=CC(OCC)=C(\C=C(\C#N)C(O)=O)C=C3OCC)=CC=C12	dichloromethane	54	10.1002/cjoc.201200091
CCCCOC1=CC=C(C=C1)N(C1=CC=C(OCCCC)C=C1)C1=CC=C(\C=C\C2=CC(OCC)=C(\C=C(\	dichloromethane	58	10.1002/cjoc.201200091

SMILES	SOLVENT	SHIFT (nm)	DOI
C#N)C(O)=O)C=C2OCC)C=C1			
OC(=O)C(=C/C1=CC=C(\C=C\C2=CC=C(C=C2)N(C2=CC=CC=C2)C2=CC=CC=C2)N1C1=CC=C(C=C1)C1=CC=C(C=C1)N(C1=CC=CC=C1)C1=CC=CC=C1)\C#N	dichloromethane	23	10.1002/chem.200901150
CCCCCCN1C2=CC=CC=C2C2=CC(=CC=C12)C1=CC=C(C=C1)N1C(\C=C\C2=CC=C(C=C2)N(C2=CC=CC=C2)C2=CC=CC=C2)=CC=C1\C=C(\C#N)C(O)=O	dichloromethane	13	10.1002/chem.200901150
N#CC(=C1C=C(/C=C/c2cc3C(C)CC(N(c3cc2CCC(=O)O)C)(C)C)CC(C1)(C)C)C#N	dichloromethane	-7	10.1039/C2RA20436C
N#C/C(=C/1\C=C(/C=C/c2ccc3c(c2)C(C)CC(N3C)(C)C)CC(C1)(C)C)/C(=O)O	dichloromethane	-22	10.1039/C2RA20436C
N#C/C(=C\1/C=C(/C=C/c2cc3C(C)CC(N(c3cc2CCC(=O)O)C)(C)C)CC(C1)(C)C)/C(=O)O	dichloromethane	-17	10.1039/C2RA20436C
COc1ccc(cc1)N1c2ccc(cc2Oc2c1cccc2)C(C(=O)O)C#N	dichloromethane	25	10.1016/j.dyepig.2018.11.010
CCCCCCOc1ccc2c(c1)Oc1c(N2c2ccc(cc2)OC)ccc(c1)/C=C(/C(=O)O)\C#N	dichloromethane	37	10.1016/j.dyepig.2018.11.010
CCCCCCCCC1(CCCCCCCC)c2cc(sc2c2c1ccs2)c1ccc(c2c1nc(c1ccccc1)c(n2)c1ccccc1)c1cc2c(s1)c1c(C2(C)C)cc(s1)/C=C(/C(=O)O)\C#N	dichloromethane	35	10.1016/j.dyepig.2017.09.055
CCCCCCCC(C1c2cc(sc2c2c1cc(s2)/C=C(/C(=O)O)\C#N)c1ccc(c2c1nc(c1ccccc1)c(n2)c1ccccc1)c1cc2c(s1)c1c(C2(C)C)ccs1)CCCCCCC	dichloromethane	33	10.1016/j.dyepig.2017.09.055
CCCCCCCCC1(CCCCCCCC)c2cc(sc2c2c1ccs2)c1ccc(c2c1nc(c1ccccc1)c(n2)c1ccccc1)c1sc2c(c1)C(c1c2sc(c1)/C=C(/C(=O)O)\C#N)C(CCCCCCC)CCCCCCC	dichloromethane	29	10.1016/j.dyepig.2017.09.055
N#CC(=Cc1ccc(s1)c1ccc(cc1)N(CCO[Si](C(C)(C)C)(C)C)C)C(=O)O	dichloromethane	51	10.1016/j.dyepig.2017.07.063
CN(c1ccc(cc1)/C=C/c1ccc(s1)C=C(C(=O)O)C#N)CCO[Si](C(C)(C)C)(C)C	dichloromethane	61	10.1016/j.dyepig.2017.07.063
N#CC(=Cc1ccc(c2c1nsn2)c1ccc(cc1)N(CCO[Si](C(C)(C)C)(C)C)C)C(=O)O	dichloromethane	54	10.1016/j.dyepig.2017.07.063
N#CC(=Cc1ccc(cc1)c1ccc(c2c1nsn2)c1ccc(cc1)N(CCO[Si](C(C)(C)C)(C)C)C)C(=O)O	dichloromethane	136	10.1016/j.dyepig.2017.07.063
CCCCCCN1c2ccc(cc2Sc2c1ccc(c2)/C=C\1/SC(=S)C(C1=O)CC(=O)O)c1cccc(c1)n1c2cccc	dichloromethane	-74	10.1016/j.dyepig.2018.05.037

SMILES	SOLVENT	SHIFT (nm)	DOI
c2c2c1cccc2			
CCCCCCN1c2ccc(cc2Sc2c1ccc(c2)/C=C\1/SC(=S)C(C1=O)CC(=O)O)c1ccc(cc1)n1c2ccccc2c2c1cccc2	dichloromethane	-67	10.1016/j.dyepig.2018.05.037
CCCCCCOc1ccc(cc1)N(c1ccc(cc1)OCCCCCC)c1ccc(cc1)c1ccc(c2c1nc(c1ccc(cc1)C(=O)O)c(n2)c1ccc(cc1)C(=O)O)c1ccc(cc1)N(c1ccc(cc1)OCCCCCC)c1ccc(cc1)OCCCCCC	dichloromethane	79	10.3390/app8091421
CCCCCCOc1ccc(cc1)N(c1ccc(cc1)OCCCCCC)c1ccc(cc1)c1ccc(c2c1nc(C(=O)O)c(n2)C(=O)O)c1ccc(cc1)N(c1ccc(cc1)OCCCCCC)c1ccc(cc1)OCCCCCC	dichloromethane	131	10.3390/app8091421
CCCCCCOc1ccc(cc1)N(c1ccc(cc1)OCCCCCC)c1ccc(cc1)c1ccc(s1)c1ccc(c2c1nc(C(=O)O)c(n2)C(=O)O)c1ccc(s1)c1ccc(cc1)N(c1ccc(cc1)OCCCCCC)c1ccc(cc1)OCCCCCC	dichloromethane	190	10.3390/app8091421
CCCCCCOc1ccc(cc1)N(c1ccc(cc1)OCCCCCC)c1ccc(cc1)c1ccc(c2c1nc1c(n2)c2ccc(nc2c2c1ccc(n2)C(=O)O)C(=O)O)c1ccc(cc1)N(c1ccc(cc1)OCCCCCC)c1ccc(cc1)OCCCCCC	dichloromethane	28	10.3390/app8091421
CCCCCCCCn1c2ccc(cc2c2c1c1c(c3c2n(CCCCCCCC)c2c3cccc2)n(c2c1cccc2)CCCCCCCC)C#Cc1ccc(c2c1nsn2)c1ccc(cc1)C(=O)O	dichloromethane	-2	10.1039/C7NJ04629D
CCCCCCCCn1c2ccc(cc2c2c1c1c(c3c2n(CCCCCCCC)c2c3cccc2)n(c2c1cccc2)CCCCCCCC)C#Cc1ccc(c2c1nsn2)c1ccc(cc1)C=C(C(=O)O)C#N	dichloromethane	-9	10.1039/C7NJ04629D
CCCCCCCCn1c2ccc(cc2c2c1c1c(c3c2n(CCCCCCCC)c2c3cccc2)n(c2c1cccc2)CCCCCCCC)c1ccc(c2c1nsn2)c1ccc(cc1)C(=O)O	dichloromethane	-13	10.1039/C7NJ04629D
CCCCCCCCn1c2ccc(cc2c2c1c1c(c3c2n(CCCCCCCC)c2c3cccc2)n(c2c1cccc2)CCCCCCCC)c1ccc(c2c1nsn2)c1ccc(cc1)C=C(C(=O)O)C#N	dichloromethane	-1	10.1039/C7NJ04629D
C(#N)C(C(=O)O)=CC1=CC=C(C=C1)C1=CC2=C(N=CC=3C=C4C(=NC23)C=C(C(=C4OC)OC)OC)C=C1	dichloromethane	-23	10.1016/j.dyepig.2019.01.027
C(#N)C(C(=O)O)=CC1=CC=C(C=C1)C1=CC2=C(N=CC=3C=C4C(=NC23)C=CC(=C4)OC)C=C1	dichloromethane	-32	10.1016/j.dyepig.2019.01.027
C(#N)C(C(=O)O)=CC1=CC=C(C=C1)C1=CC2=C(N=CC=3C=C4C(=NC23)C=CC(=C4)N(C)C)C=C1	dichloromethane	-31	10.1016/j.dyepig.2019.01.027
C(#N)/C(/C(=O)O)=C\C1=CC=C(C=C1)C1=CC(=C(C2=NSN=C21)C2	dichloromethane	-52	10.1016/j.electacta.2019.02.077

SMILES	SOLVENT	SHIFT (nm)	DOI
=CC=C(C=C2)N(C2=CC=CC=C2)C2=CC=CC=C2)C			
C(#N)/C(/C(=O)O)=C/C=1SC(=CC1)C1=CC(=C(C2=NSN=C21)C2=CC=C(C=C2)N(C2=CC=CC=C2)C2=CC=CC=C2)C	dichloromethane	-87	10.1016/j.electacta.2019.02.077
C(#N)/C(/C(=O)O)=C\C1=CC=C(C=C1)C1=C(C=C(C2=NSN=C21)C2=CC=C(C=C2)N(C2=CC=CC=C2)C2=CC=CC=C2)C	dichloromethane	-2	10.1016/j.electacta.2019.02.077
C(#N)/C(/C(=O)O)=C/C=1SC(=CC1)C1=C(C=C(C2=NSN=C21)C2=CC=C(C=C2)N(C2=CC=CC=C2)C2=CC=CC=C2)C	dichloromethane	-38	10.1016/j.electacta.2019.02.077
CCCCCCOC1=CC=C(C=C1)N(C1=CC=C(OCCCCCC)C=C1)C1=CC=C(\C=C\C2=CC=C(S2)\C=C(/CC#N)C(O)=O)C=C1	dichloromethane	88	10.1002/ejoc.201801497
CCCCCCOC1=CC=C(C=C1)N(C1=CC=C(OCCCCCC)C=C1)C1=CC=C(\C=C\C2=CC=C(S2)\C=C(/CC#N)C(S)=O)C=C1	dichloromethane	72	10.1002/ejoc.201801497
CCCCCCOC1=CC=C(C=C1)N(C1=CC=C(OCCCCCC)C=C1)C1=CC=C(\C=C\C2=CC=C(S2)\C=C(/CC#N)C(S)=S)C=C1	dichloromethane	48	10.1002/ejoc.201801497
CCCCC(Cn1c2cc3c4ccc(cc4n(c3cc2c2c1cc(cc2)c1ccc(cc1)N(c1ccccc1)c1ccccc1)CC(CCCC)CC)c1ccc(s1)/C=C(/C(=O)O)\C#N)CC	dichloromethane	47	10.1016/j.solener.2018.08.029
CCCCC(Cn1c2cc3c4cc(ccc4n(c3cc2c2c1ccc(c2)c1ccc(cc1)N(c1ccccc1)c1ccccc1)CC(CCCC)CC)c1ccc(s1)/C=C(/C(=O)O)\C#N)CC	dichloromethane	80	10.1016/j.solener.2018.08.029
C(#N)/C(/C(=O)O)=C\C1=CC=C(C=C1)C=1C=CC=2C(N(C(C3=CC=CC1C23)=O)C=2C=CC=3N(C1=CC=CC=C1C3C2)CC)=O	dichloromethane	-9	10.1007/s10854-018-9750-4
C(#N)/C(/C(=O)O)=C\C=1SC(=CC1)C=1C=CC=2C(N(C(C3=CC=CC1C23)=O)C=2C=CC=3N(C1=CC=CC=C1C3C2)CC)=O	dichloromethane	12	10.1007/s10854-018-9750-4
C(#N)/C(/C(=O)O)=C\C1=CC=C(S1)C=1SC(=CC1)C=1C=CC=2C(N(C(C3=CC=CC1C23)=O)C=2C=CC=3N(C1=CC=CC=C1C3C2)CC)=O	dichloromethane	18	10.1007/s10854-018-9750-4
C(#N)/C(/C(=O)O)=C\C1=CC2=C(C3=C(N2C2=CC=4C(C5=CC=CC=C5C4C=C2)	dichloromethane	21	10.1007/s10854-018-9750-4

SMILES	SOLVENT	SHIFT (nm)	DOI
(CCCC)CCCC)C=C(S3)C=3C=CC=2C(N(C(C4=CC=CC3C24)=O)C=2C=CC=4N(C3=CC=CC=C3C4C2)CC)=O)S1			
N#C/C(=C\c1ccc(cc1)c1ccc(cc1)N(c1ccc(cc1)/C=C/c1ccccc1)c1ccc(cc1)/C=C/c1ccccc1)/C(=O)O	dichloromethane	-47	10.1002/solr.201900066
N#C/C(=C\c1ccc(o1)c1ccc(cc1)N(c1ccc(cc1)/C=C/c1ccccc1)c1ccc(cc1)/C=C/c1ccccc1)/C(=O)O	dichloromethane	-1	10.1002/solr.201900066
N#C/C(=C\c1ccc(s1)c1ccc(cc1)N(c1ccc(cc1)/C=C/c1ccccc1)c1ccc(cc1)/C=C/c1ccccc1)/C(=O)O	dichloromethane	6	10.1002/solr.201900066
CCCCCCc1cc(sc1c1ccc(cc1)n1c2cc(sc2c2c1cc(s2)c1ccc(cc1)OCC(CCCC)CC)c1ccc(cc1)OCC(CCCC)CC)c1ccc(c2c1nsn2)c1sc(c(c1)CCCCCC)c1ccc(cc1)C(=O)O	dichloromethane	-11	10.1016/j.solener.2019.04.059
CCCCCCc1cc(sc1c1cc2c(s1)c1c(n2c2ccc(cc2)OCC(CCCC)CC)ccs1)c1ccc(c2c1nsn2)c1sc(c(c1)CCCCCC)c1ccc(cc1)C(=O)O	dichloromethane	-10	10.1016/j.solener.2019.04.059
CCCCCCOc1ccc(cc1)N(c1ccc(cc1)OCCCCCC)c1ccc(cc1)c1sc2c(c1CCCCCC)sc1c2n(c2ccc3c(c2)C(CCCCCC)(CCCCCC)c2c3c3c(c4c2c2ccccc2C4(CCCCCC)CCCCCC)c2c(C3(CCCCCC)CCCCCC)cccc2)c2c1sc1c2sc(c1CCCCCC)/C=C(/C(=O)O)\C#N	dichloromethane	63	10.1016/j.dyepig.2019.04.048
CCCCCCOc1ccc(cc1)N(c1ccc2c(c1)C(CCCCCC)(CCCCCC)c1c2c2c(c3c1c1ccccc1C3(CCCCCC)CCCCCC)c1c(C2(CCCCCC)CCCCCC)cccc1)c1ccc(cc1)c1sc2c(c1CCCCCC)sc1c2n(c2ccc(cc2)OCCCCCC)c2c1sc1c2sc(c1CCCCCC)/C=C(/C(=O)O)\C#N	dichloromethane	43	10.1016/j.dyepig.2019.04.048
CCCCOc1cc(OCCCC)ccc1c1ccc(cc1)N(c1ccc(cc1)c1ccc(cc1OCCCC)OCCCC)c1ccc(cc1)c1ccc(s1)c1ccc(s1)c1cc(nc(c1)C(=O)O)C(=O)O	dichloromethane	-94	10.1039/C4CC06432A
CCCCCCCCCCCCn1c2ccc(cc2c2c1ccc(c2)c1ccc(c2c1nsn2)c1ccc2c(c1)n(CCCCCCCCCCCC)c1c2cc(cc1)/C=C(/C(=O)O)\C#N)/C=C(/C(=O)O)\C#N	DMF	-16	10.1016/j.dyepig.2016.07.017
CCCCCCCCCCCCN1c2ccc(cc2Sc2c1ccc(c2)/C=C(/C(=O)O)\C#N)c1ccc(c2c1nsn2)c1ccc2c(c1)Sc1c(N2CCCCCCCCCCCC)ccc(c1)/C=C(/C(=O)O)\C#N	DMF	-11	10.1016/j.dyepig.2016.07.017
CCN1c2ccc(cc2Sc2c1cccc2)/C=C/c1sc(c2c1OCCO2)/C=C(/C(=O)O)\C#N	DMF	-8	10.1016/j.matlet.2010.10.072

SMILES	SOLVENT	SHIFT (nm)	DOI
CCCCCCc1ccc(cc1)N1c2ccc(cc2Sc2c1ccc(c2)c1ccc(s1)/C=C(\C(=O)O)/C#N)c1ccc(s1)/C=C(\C(=O)O)/C#N	DMF	-5	10.1016/j.dyepig.2013.10.032
CCCCCCc1ccc(cc1)N1c2ccc(cc2Sc2c1ccc(c2)c1ccc(s1)c1ccc(s1)/C=C(/C(=O)O)\C#N)c1ccc(s1)c1ccc(s1)/C=C(\C(=O)O)/C#N	DMF	3	10.1016/j.dyepig.2013.10.032
C(#N)C(C(=O)O)=CC=1C=CC=2N(C3=CC=CC=C3SC2C1)CCCCCC	DMF	-2	10.1016/j.dyepig.2013.08.005
N#C/C(=C/c1ccc2c(c1)Sc1c(N2CCCCCCN2c3ccccc3Sc3c2ccc(c3)/C=C(/C(=O)O)\C#N)cccc1)/C(=O)O	DMF	-6	10.1016/j.dyepig.2013.08.005
N#C/C(=C\c1ccc2c(c1)Sc1c(N2CCCCCOc2ccc(cc2)[C@@](c2ccc(cc2)OCCCCCCN2c3ccccc3Sc3c2ccc(c3)/C=C(\C(=O)O)/C#N)(c2ccc(cc2)OCCCCCCN2c3ccccc3Sc3c2ccc(c3)/C=C(/C(=O)O)\C#N)C)cccc1)/C(=O)O	DMF	-14	10.1016/j.dyepig.2013.08.005
CCCCCCN1c2ccc(cc2Sc2c1cccc2)c1ccc(s1)/C=C(/C(=O)O)\C#N	DMF	-16	10.1016/j.synthmet.2011.02.012
CCCCCCN1c2ccc(cc2Sc2c1ccc(c2)c1ccc(s1)/C=C(/C(=O)O)\C#N)c1ccc(s1)/C=C(/C(=O)O)\C#N	DMF	22	10.1016/j.synthmet.2011.02.012
N#C/C(=C\c1ccc(cc1)N(c1ccc(cc1)/C=C(/C(=O)O)\C#N)c1ccc(cc1)N1c2ccccc2Sc2c1cccc2)/C(=O)O	DMF	-23	10.1166/jnn.2017.14076
OC(=O)CN1C(=S)S/C(=C\c2ccc(cc2)N(c2ccc(cc2)N2c3ccccc3Sc3c2cccc3)c2ccc(cc2)/C=C/2\SC(=S)N(C2=O)CC(=O)O)/C1=O	DMF	-2	10.1166/jnn.2017.14076
CCCCCCn1c2ccc(cc2c2c1ccc(c2)/C=C/1\SC(=S)N(C1=O)CC(=O)O)/C=C/1\SC(=S)N(C1=O)CC(=O)O	DMF	8	10.1590/S0103-50532011000400023
CCN1c2ccc(cc2CCc2c1ccc(c2)/C=C/1\SC(=S)N(C1=O)CC(=O)O)/C=C/1\SC(=S)N(C1=O)CC(=O)O	DMF	-2	10.1590/S0103-50532011000400023
CCN1c2ccc(cc2Sc2c1ccc(c2)/C=C/1\SC(=S)N(C1=O)CC(=O)O)/C=C/1\SC(=S)N(C1=O)CC(=O)O	DMF	1	10.1590/S0103-50532011000400023
CCCCCCCC[N+]1=C(/C=C/2\C(=C(C2=O)/C=C\2/N(CC)c3c(C2(C)C)cccc3)[O-])C(c2c1ccc(c2)C(=O)O)(C)C	DMF	6	10.1002/adfm.201203384

SMILES	SOLVENT	SHIFT (nm)	DOI
CCCCCCCC[N+]1=C(/C=C\2/C(=C(C2=C(C#N)C#N)/C=C\2/N(CC)c3c(C2(C)C)cccc3)[O-])C(c2c1ccc(c2)C(=O)O)(C)C	DMF	-8	10.1002/adfm.201203384
CCCCCCCCN1c2ccccc2C(/C/1=C\C1=C([O-])/C(=C/C2=[N+](CCCCCCCC)c3c(C2(C)C)cc(cc3)C(=O)O)/C1=C(C#N)C#N)(C)C	DMF	-2	10.1002/adfm.201303769
CCCCCCCC[N+]1=C(/C=C\2/C(=C(/C2=C(\C(=O)OCC)/C#N)/C=C/2\N(CCCCCCCO)c3c(C2(C)C)cccc3)[O-])C(c2c1ccc(c2)C(=O)O)(C)C	DMF	-8	10.1002/adfm.201303769
CCCCCCCC[N+]1=C(/C=C\2/C(=C(/C2=C(\C(=O)OCC)/C#N)/C=C/2\N(CCCCCCCO)c3c(C2(C)C)cc(cc3)C(=O)O)[O-])C(c2c1ccc(c2)C(=O)O)(C)C	DMF	-8	10.1002/adfm.201303769
N#C/C(=C\c1ccc(s1)c1ccc(cc1)N(c1ccc(cc1)c1cccs1)c1ccc(cc1)c1cccs1)/C(=O)O	DMF	-18	10.1016/j.synthmet.2016.04.009
N#C/C(=C\c1ccc(s1)c1ccc(cc1)N(c1ccc(cc1)c1ccc(s1)/C=C(/C(=O)O)\C#N)c1ccc(cc1)c1cccs1)/C(=O)O	DMF	-14	10.1016/j.synthmet.2016.04.009
N#C/C(=C\c1ccc(s1)c1ccc(cc1)N(c1ccc(cc1)c1ccc(s1)/C=C(/C(=O)O)\C#N)c1ccc(cc1)c1ccc(s1)/C=C(/C(=O)O)\C#N)/C(=O)O	DMF	-6	10.1016/j.synthmet.2016.04.009
C(#N)C(C(=O)O)=CC=1C=CC=2N(C3=CC=CC=C3SC2C1)CCCCCC	DMF	-36	10.1016/j.dyepig.2016.05.035
N#C/C(=C\c1ccc2c(c1)Sc1c(N2CCCCN2c3ccccc3Sc3c2ccc(c3)/C=C(/C(=O)O)\C#N)cccc1)/C(=O)O	DMF	-21	10.1016/j.dyepig.2016.05.035
N#C/C(=C\c1ccc2c(c1)Sc1c(N2CCCCCCN2c3ccccc3Sc3c2ccc(c3)/C=C(/C(=O)O)\C#N)cccc1)/C(=O)O	DMF	-24	10.1016/j.dyepig.2016.05.035
N#C/C(=C\c1ccc2c(c1)Sc1c(N2CCCCCCCCN2c3ccccc3Sc3c2ccc(c3)/C=C(/C(=O)O)\C#N)cccc1)/C(=O)O	DMF	-23	10.1016/j.dyepig.2016.05.035
N#C/C(=C\c1ccc2c(c1)Sc1c(N2CCCCCCCCCCN2c3ccccc3Sc3c2ccc(c3)/C=C(/C(=O)O)\C#N)cccc1)/C(=O)O	DMF	-32	10.1016/j.dyepig.2016.05.035

SMILES	SOLVENT	SHIFT (nm)	DOI
N#C/C(=C\c1ccc2c(c1)Sc1c(N2CCCCCCCCCCCCN2c3ccccc3Sc3c2ccc(c3)/C=C(/C(=O)O)\C#N)cccc1)/C(=O)O	DMF	-27	10.1016/j.dyepig.2016.05.035
N#C/C(=C\c1ccc(s1)c1ccc2c(c1)Sc1c(N2C2CCCCC2)cccc1)/C(=O)O	DMF	-53	10.1016/j.dyepig.2017.11.042
N#C/C(=C\c1ccc(s1)c1ccc2c(c1)c1ccccc1n2CC)/C(=O)O	DMF	-68	10.1016/j.dyepig.2017.11.042
N#C/C(=C\c1ccc(s1)c1ccc2c(c1)Sc1c(N2CCCCCCn2c3ccc(cc3c3c2cccc3)c2ccc(s2)/C=C(/C(=O)O)\C#N)cccc1)/C(=O)O	DMF	-77	10.1016/j.dyepig.2017.11.042
CCCCCCOc1ccc(cc1)N(c1ccc(cc1)OCCCCCC)c1ccc(cc1)c1sc(c2c1OCCO2)c1sc2c(c1)sc(c2)c1ccc(c(c1)C#N)C(=O)O	DMF	10	10.1021/la203104v
OC(=O)c1ccc(cc1)C#Cc1ccc(s1)c1ccc2c(c1)Sc1c(N2c2ccc3c(c2)C(C)(C)c2c3cccc2)ccc(c1)c1ccc2c(c1)C(C)(C)c1c2cccc1	DMF	-22	10.1039/c5nj02967h
OC(=O)c1cccc(c1)c1cc(C#Cc2ccc(s2)c2ccc3c(c2)Sc2c(N3c3ccc4c(c3)C(C)(C)c3c4cccc3)ccc(c2)c2ccc3c(c2)C(C)(C)c2c3cccc2)cc(c1)c1cccc(c1)C(=O)O	DMF	-25	10.1039/c5nj02967h
CC1(C)c2cc(ccc2c2c1cccc2)N1c2ccc(cc2Sc2c1ccc(c2)c1ccc2c(c1)C(C)(C)c1c2cccc1)c1ccc(s1)C#Cc1cc(cc(c1)c1cccnc1)c1cccnc1	DMF	-13	10.1039/c5nj02967h
C(CCCCC)N1C2=CC=CC=C2SC=2C=C(C=CC12)\C=C\1/C(C2=CC=C(C=C2C1=O)C(=O)O)=O	DMF	-42	10.1016/j.cplett.2018.11.026
OC(=O)c1ccc2c(c1)C(=O)/C(=C/c1ccc3c(c1)Sc1c(N3CCCCCCCCCCN3c4ccccc4Sc4c3ccc(c4)/C=C/3\C(=O)c4c(C3=O)cc(cc4)C(=O)O)cccc1)/C2=O	DMF	-64	10.1016/j.cplett.2018.11.026
CCCCCCCN1c2ccc(cc2Sc2c1cccc2)/C=C(/C(=O)O)\C#N	DMF	-33	10.1016/j.cplett.2018.11.026
N#C/C(=C\c1ccc2c(c1)Sc1c(N2CCCCCCCN2c3ccccc3Sc3c2ccc(c3)/C=C(/C(=O)O)\C#N)cccc1)/C(=O)O	DMF	-25	10.1016/j.cplett.2018.11.026
C(#N)/C(/C(=O)O)=C\	DMF	26	10.1002/chem.201402342

SMILES	SOLVENT	SHIFT (nm)	DOI
C1=CC=C(S1)C=1SC(=CC1)C1=C2N=C(C(=NC2=C(C=C1)C1=CC=C(C=C1)N(C1=CC=CC=C1)C1=CC=CC=C1)C1=CC=CC=C1)C1=CC=CC=C1			
C(C)C1=CC=C(C=C1)N(C1=CC=C(C=C1)C=1C=CC(=C2N=C(C(=NC12)C1=CC=CC=C1)C1=CC=CC=C1)C1=CC=C(S1)/C=C(/C(=O)O)\C#N)C1=CC=C(C=C1)CC	DMF	31	10.1002/chem.201402342
C(#N)/C(/C(=O)O)=C\C1=CC2=C(S1)C=C(S2)C2=C1N=C(C(=NC1=C(C=C2)C2=CC=C(C=C2)N(C2=CC=CC=C2)C2=CC=CC=C2)C2=CC=CC=C2)C2=CC=CC=C2	DMF	57	10.1002/chem.201402342
C(#N)/C(/C(=O)O)=C\C=1SC(=CC1)C1=C2N=C(C(=NC2=C(C=C1)C1=CC=C(S1)C=1SC(=CC1)C1=CC=C(C=C1)N(C1=CC=CC=C1)C1=CC=CC=C1)C1=CC=CC=C1)C1=CC=CC=C1	DMF	105	10.1002/chem.201402342
C(#N)/C(/C(=O)O)=C\C1=CC2=C(C3=C(S2)C=C(S3)C3=C2N=C(C(=NC2=C(C=C3)C3=CC=C(C=C3)N(C3=CC=CC=C3)C3=CC=CC=C3)C3=CC=CC=C3)C3=CC=CC=C3)S1	DMF	9	10.1002/chem.201402342
C(C)C1=CC=C(C=C1)N(C1=CC=C(C=C1)C=1C=CC(=C2N=C(C(=NC12)C1=CC=CC=C1)C1=CC=CC=C1)C1=CC=C(S1)C=1SC(=CC1)/C=C(/C(=O)O)\C#N)C1=CC=C(C=C1)CC	DMF	11	10.1002/chem.201402342
C(C)C1(C2=CC=CC=C2C=2C=CC(=CC12)N(C1=CC=C(C=C1)C=1C=CC(=C2N=C(C(=NC12)C1=CC=CC=C1)C1=CC=CC=C1)C1=CC=C(S1)/C=C(/C(=O)O)\C#N)C1=CC=2C(C3=CC=CC=C3C2C=C1)(CC)CC)CC	DMF	2	10.1002/chem.201402342
C(#N)/C(/C(=O)O)=C\C1=C2N=C(C(=NC2=C(C=C1)C1=CC=C(C=C1)N(C1=CC=CC=C1)C1=CC=CC=C1)C1=CC=CC=C1)C1=CC=CC=C1	DMF	-22	10.1002/chem.201201000
C(#N)/C(/C(=O)O)=C\C1=C2N=C(C(=NC2=C(C=C1)\C=C\C1=CC=C(C=C1)N(C1=CC=CC=C1)C1=CC=CC=C1)C1=CC=CC=C1)C1=CC=CC=C1	DMF	-32	10.1002/chem.201201000
C(#N)/C(/C(=O)O)=C\C1=C2N=C(C(=NC2=C(C=C1)C=1SC(=CC1)C1=CC=C(C=C1)N(C1=CC=CC=C1)C1=CC=CC=C1)C1=CC=CC=C1)C1=CC=CC=C1	DMF	-57	10.1002/chem.201201000
C(#N)/C(/C(=O)O)=C\	DMF	-64	10.1002/chem.201201000

SMILES	SOLVENT	SHIFT (nm)	DOI
C1=C2N=C(C(=NC2=C(C=C1)C=1SC(=C(C1)CCCCCC)C1=CC=C(C=C1)N(C1=CC=CC=C1)C1=CC=CC=C1)C1=CC=CC=C1)C1=CC=CC=C1			
C(#N)/C(/C(=O)O)=C\C1=C2N=C(C(=NC2=C(C=C1)C1=CC(=C(S1)C=1SC(=C(C1)CCCCCC)C1=CC=C(C=C1)N(C1=CC=CC=C1)C1=CC=CC=C1)CCCCCC)C1=CC=CC=C1)C1=CC=CC=C1	DMF	-92	10.1002/chem.201201000
C(#N)/C(/C(=O)O)=C\C=1SC(=CC1)C1=C2N=C(C(=NC2=C(C=C1)C1=CC=C(C=C1)N(C1=CC=CC=C1)C1=CC=CC=C1)C1=CC=CC=C1)C1=CC=CC=C1	DMF	-40	10.1002/chem.201201000
OC(=O)C(=C/C1=CC=C(\C=C\C2=CC=C(C=C2)N(C2=CC=CC=C2)C2=CC=CC=C2)N1C1=CC=C(C=C1)C1=CC=C(C=C1)N(C1=CC=CC=C1)C1=CC=CC=C1)\C#N	DMF	-35	10.1002/chem.200901150
CCCCCCN1C2=CC=CC=C2C2=CC(=CC=C12)C1=CC=C(C=C1)N1C(\C=C\C2=CC=C(C=C2)N(C2=CC=CC=C2)C2=CC=CC=C2)=CC=C1\C=C(\C#N)C(O)=O	DMF	-29	10.1002/chem.200901150
CCCCCCN1c2ccc(cc2Oc2c1ccc(c2)/C=C(/c1ccc(s1)/C=C(/C(=O)O)\C#N)\C#N)/C=C(\c1ccc(s1)/C=C(/C(=O)O)\C#N)/C#N	DMF	-2	10.1039/C3RA42852D
N#C/C(=C/c1ccc(cc1)N(c1ccc(cc1)/C=C(/c1ccc(s1)/C=C(/C(=O)O)\C#N)\C#N)c1ccccc1)/c1ccc(s1)/C=C(/C(=O)O)\C#N	DMF	34	10.1039/C3RA42852D
N#CC(=Cc1ccc(s1)c1ccc(cc1)N(c1ccc(cc1)c1ccc(s1)C=C(C(=O)O)C#N)c1ccc(cc1)c1ccc(s1)C=C(C(=O)O)C#N)C(=O)O	DMF	-4	10.1039/C6RA00636A
N#C/C(=C/c1ccc(s1)c1ccc(s1)c1ccc(s1)/C=C/c1c(OC)cc(cc1OC)OC)/C(=O)O	DMF	-55	10.1016/j.dyepig.2018.05.011
COc1cc(OC)cc(c1/C=C/c1ccc(s1)c1ccc(s1)c1ccc(s1)/C=C/1\SC(=S)N(C1=O)CC(=O)O)OC	DMF	-27	10.1016/j.dyepig.2018.05.011
N#C/C(=C\c1ccc(s1)c1ccc(cc1)n1c2cc3c(cc2c2c1cccc2)c1c(C3(C)C)cccc1)/C(=O)O	DMF	-34	10.1039/c7pp00350a
N#C/C(=C\c1ccc(s1)c1ccc(s1)c1ccc(cc1)n1c2cc3c(cc2c2c1cccc2)c1c(C3(C)C)cccc1)/C(=O)O	DMF	-17	10.1039/c7pp00350a
N#C/C(=C\c1ccc(s1)c1ccc2c(c1)c1cc3c4ccccc4C(c3cc1n2c1ccccc1)(C)C)/C(=O)O	DMF	-35	10.1039/c7pp00350a

SMILES	SOLVENT	SHIFT (nm)	DOI
N#C/C(=C\c1ccc(s1)c1ccc(s1)c1ccc2c(c1)c1cc3c4ccccc4C(c3cc1n2c1ccccc1)(C)C)/C(=O)O	DMF	-26	10.1039/c7pp00350a
CCCCCC[Si]1(CCCCCC)c2sc(cc2c2c1cc(s2)/C=C(/C(=O)O)\C#N)c1sc(c2c1OCCO2)c1ccc(cc1)N(c1ccc(cc1)OCC(CCCC)CC)c1ccc(cc1)OCC(CCCC)CC	DMF	0	10.1021/cm9036988
N#C/C(=C\c1ccc(cc1)N(c1ccccn1)c1ccccn1)/c1ccc(s1)/C=C(/C(=O)O)\C#N	DMF	-82	10.1039/C7NJ04620K
N#C/C(=C\c1ccc(s1)N(c1ccccn1)c1ccccn1)/c1ccc(s1)/C=C(/C(=O)O)\C#N	DMF	-81	10.1039/C7NJ04620K
N#C/C(=C\c1ccc(cc1)N(c1ccccn1)c1ccccn1)/c1ccc(s1)/C=C/1\SC(=S)N(C1=O)CC(=O)O	DMF	-70	10.1039/C7NJ04620K
N#C/C(=C\c1ccc(s1)N(c1ccccn1)c1ccccn1)/c1ccc(s1)/C=C/1\SC(=S)N(C1=O)CC(=O)O	DMF	-65	10.1039/C7NJ04620K
OC(=O)c1ccc(cc1)C#Cc1ccc2c3c1cccc3c1c3c2c(Oc2ccc(cc2)C(C)(C)C)cc2c3c(cc1Oc1cccc(c1)C(C)(C)C)c(=O)n(c2=O)c1cc(ccc1C(C)(C)C)C(C)(C)C	DMF	46	10.1039/C8SC05693E
C/C(=C(\c1ccc(cc1)C#Cc1ccc2c3c1cccc3c1c3c2c(Oc2ccc(cc2)C(C)(C)C)cc2c3c(cc1Oc1cccc(c1)C(C)(C)C)c(=O)n(c2=O)c1cc(ccc1C(C)(C)C)C(C)(C)C)/C(=O)C)/O	DMF	28	10.1039/C8SC05693E
O=c1c2cc(Oc3cccc(c3)C(C)(C)C)c3c4c2c(c(=O)n1c1cc(ccc1C(C)(C)C)C(C)(C)C)cc(c4c1c2c3cccc2c(cc1)C#Cc1ccc(cc1)P(=O)(O)O)Oc1ccc(cc1)C(C)(C)C	DMF	36	10.1039/C8SC05693E
Oc1ccc(c2c1nccc2)C#Cc1ccc2c3c1cccc3c1c3c2c(Oc2ccc(cc2)C(C)(C)C)cc2c3c(cc1Oc1cccc(c1)C(C)(C)C)c(=O)n(c2=O)c1cc(ccc1C(C)(C)C)C(C)(C)C	DMF	20	10.1039/C8SC05693E
OC(=O)c1cc(C#Cc2ccc3c4c2cccc4c2c4c3c(Oc3ccc(cc3)C(C)(C)C)cc3c4c(cc2Oc2cccc(c2)C(C)(C)C)c(=O)n(c3=O)c2cc(ccc2C(C)(C)C)C(C)(C)C)cc(n1)C(=O)O	DMF	46	10.1039/C8SC05693E
CCCCCCn1c2ccc(cc2c2c1cccc2)C(=CC=Nc1ccc(c(c1)O)C(=O)O)Cl	DMF	-12	10.1002/slct.201803940

SMILES	SOLVENT	SHIFT (nm)	DOI
CCCCCCn1c2ccc(cc2c2c1ccc(c2)C(=CC=Nc1ccc(c(c1)O)C(=O)O)Cl)C(=CC=Nc1ccc(c(c1)O)C(=O)O)Cl	DMF	-30	10.1002/slct.201803940
ClC(=CC=Nc1ccc(c(c1)O)C(=O)O)c1ccc2c(c1)c1ccccc1n2c1ccccc1	DMF	-20	10.1002/slct.201803940
ClC(=CC=Nc1ccc(c(c1)O)C(=O)O)c1ccc2c(c1)c1cc(ccc1n2c1ccccc1)C(=CC=Nc1ccc(c(c1)O)C(=O)O)Cl	DMF	-6	10.1002/slct.201803940
OC(=O)c1ccc(cc1)c1ccc2c(c1)[nH]c1c2ccc(c1)N(c1ccccc1)c1ccccc1	dioxane	-10	10.1002/ange.201102552
CCCCn1c2cc(ccc2c2c1cc(cc2)N(c1ccccc1)c1ccccc1)c1ccc(cc1)C(=O)O	dioxane	-8	10.1002/ange.201102552
c1ccc(cc1)N(c1ccc2c(c1)[nH]c1c2ccc(c1)c1ccncc1)c1ccccc1	dioxane	-25	10.1002/ange.201102552
CCCCn1c2cc(ccc2c2c1cc(cc2)c1ccncc1)N(c1ccccc1)c1ccccc1	dioxane	-25	10.1002/ange.201102552
c1ccc(cc1)N(c1ccc2c(c1)[nH]c1c2ccc(c1)c1ccc(s1)c1ccncc1)c1ccccc1	dioxane	-30	10.1002/ange.201102552
CCCCn1c2cc(ccc2c2c1cc(cc2)N(c1ccccc1)c1ccccc1)c1ccc(s1)c1ccncc1	dioxane	-30	10.1002/ange.201102552
CCCCCCOc1cc(C=C(C(=O)O)C#N)c(cc1n1nnc(c1)c1ccc(cc1)N(c1ccccc1)c1ccccc1)OCCCCCC	dioxane	-32	10.1016/ j.dyepig.2011.11.008
CCCCCCOc1cc(/C=C(\C(=O)O)/C#N)c(cc1n1nnc(c1)c1ccc(cc1)N(c1ccc(cc1)c1nnn(c1)c1cc(OCCCCCC)c(cc1OCCCCCC)/C=C(\C(=O)O)/C#N)c1ccccc1)OCCCCCC	dioxane	0	10.1016/ j.dyepig.2011.11.008
CCCCCCOc1cc(/C=C(\C(=O)O)/C#N)c(cc1n1nnc(c1)c1ccc(cc1)N(c1ccc(cc1)c1nnn(c1)c1cc(OCCCCCC)c(cc1OCCCCCC)/C=C(\C(=O)O)/C#N)c1ccc(cc1)c1nnn(c1)c1cc(OCCCCCC)c(cc1OCCCCCC)/C=C(\C(=O)O)/C#N)OCCCCCC	dioxane	-71	10.1016/ j.dyepig.2011.11.008
C1(=CC=CC=C1)N(C1=CC=2N(C3=CC(=CC=C3C2C=C1)C1=CC=NC=C1)CCCCCCC(=O)O)C1=CC=CC=C1	dioxane	-25	10.1002/chem.201101923
C1(=CC=CC=C1)N(C1=CC=2N(C3=CC(=CC=C3C2C=C1)C=1SC(=CC1)C1=CC=NC=C1)CCCCCCC(=O)O)C1=CC=CC=C1	dioxane	-40	10.1002/chem.201101923
O=Cc1ccc(cc1)c1ccc2c(c1)n(CCCCCCC(=O)O)c1c2ccc(c1)N(c1ccccc1)c1ccccc1	dioxane	-10	10.1039/C3NJ00430A
CCCCn1c2cc(ccc2c2c1cc(cc2)N(c1ccccc1)c1ccccc1)c1ccc(cc1)C=O	dioxane	-26	10.1039/C3NJ00430A

SMILES	SOLVENT	SHIFT (nm)	DOI
COc1ccc(cc1)N(c1ccc(cc1)OC)c1ccc(cc1)c1sc(c2c1OC[C@]1(CO2)COc2c(OC1)c(sc2c1ccc(cc1)N(c1ccc(cc1)OC)c1ccc(cc1)OC)/C=C(/C(=O)O)\C#N)/C=C(\C(=O)O)/C#N	DMSO	-27	10.1021/acsami.7b09010
COc1ccc(cc1)N(c1ccc(c2c1nsn2)c1sc(c2c1OC[C@@]1(CO2)COc2c(OC1)c(sc2/C=C(/C(=O)O)\C#N)c1ccc(c2c1nsn2)N(c1ccc(cc1)OC)c1ccc(cc1)OC)/C=C(\C(=O)O)/C#N)c1ccc(cc1)OC	DMSO	31	10.1021/acsami.7b09010
COc1ccc(cc1)N(c1ccc(cc1)OC)c1ccc(cc1)c1sc(c2c1OC[C@@]1(CO2)COc2c(OC1)c(sc2c1ccc(c2c1nsn2)N(c1ccc(cc1)OC)c1ccc(cc1)OC)/C=C(\C(=O)O)/C#N)/C=C(/C(=O)O)\C#N	DMSO	5	10.1021/acsami.7b09010
CCCC[C@@H](COc1ccc(cc1)N(c1ccc(cc1)OC[C@H](CCCC)CC)c1ccc(cc1)c1sc(c2c1OC[C@]1(CO2)COc2c(OC1)c(sc2/C=C(/C(=O)O)\C#N)c1ccc(c2c1nsn2)N(c1ccc(cc1)OC)c1ccc(cc1)OC)/C=C(/C(=O)O)\C#N)CC	DMSO	-25	10.1021/acsami.7b09010
COc1ccc(cc1)N(c1ccc(cc1)OC)c1ccc(cc1)c1sc(c(c1OC)OC)/C=C(\C(=O)O)/C#N	DMSO	62	10.1021/acsami.7b09010
COc1ccc(cc1)N(c1ccc(cc1)OC)c1ccc(cc1)c1ccc(s1)c1sc(c2c1OCC1(CO2)COc2c(OC1)c(sc2c1ccc(s1)c1ccc(cc1)N(c1ccc(cc1)OC)c1ccc(cc1)OC)C(C(=O)O)C#N)C(C(=O)O)C#N	DMSO	-24	10.1016/j.dyepig.2018.09.056
COc1ccc(cc1)N(c1ccc(cc1)OC)c1ccc(cc1)c1sc(c2c1OCC1(CO2)COc2c(OC1)c(sc2c1ccc(cc1)N(c1ccc(cc1)OC)c1ccc(cc1)OC)c1ccc(s1)C(C(=O)O)C#N)c1ccc(s1)C(C(=O)O)C#N	DMSO	-38	10.1016/j.dyepig.2018.09.056
COc1ccc(cc1)N(c1ccc(cc1)OC)c1ccc(cc1)c1ccc(s1)c1sc(c2c1OCC1(CO2)COc2c(OC1)c(sc2c1ccc(s1)c1ccc(cc1)N(c1ccc(cc1)OC)c1ccc(cc1)OC)c1ccc(s1)C(C(=O)O)C#N)c1ccc(s1)C(C(=O)O)C#N	DMSO	37	10.1016/j.dyepig.2018.09.056
N#CC(=Cc1ccc(s1)c1sc(c2c1nsn2)c1ccc(s1)C=C(C(=O)O)C#N)C(=O)O	DMSO	-15	10.1039/C2RA21718J
OC(=O)CN1C(=S)S/C(=C\c2ccc(s2)c2cc3CCCN4c3c(c2)CCC4)/C1=O	ethanol	102	10.1016/j.synthmet.2013.07.028
N#C/C(=C\c1ccc(s1)c1cc2CCCN3c2c(c1)CCC3)/C(=O)O	ethanol	41	10.1016/j.synthmet.2013.07.028
OC(=O)CN1C(=S)S/C(=C\c2cc3CCCN4c3c(c2)CCC4)/C1=O	ethanol	86	10.1016/j.synthmet.2013.07.028
N#C/C(=C/c1ccc(s1)c1ccc2c(c1)c1ccccc1n2c1ccc2c(c1	ethanol	3	10.1016/j.tet.2006.12.082

SMILES	SOLVENT	SHIFT (nm)	DOI
)C(C)(C)c1c2cccc1)/C(=O)O			
N#C/C(=C\ c1ccc(s1)c1ccc(s1)c1ccc2c(c1)c1ccccc1n2c1ccc2c(c1)C(C)(C)c1c2cccc1)/C(=O)O	ethanol	0	10.1016/j.tet.2006.12.082
OC(=O)CN1C(=S)S/C(=C\ c2ccc(s2)c2ccc3c(c2)c2ccccc2n3c2ccc3c(c2)C(C)(C)c2c3cccc2)/C1=O	ethanol	-17	10.1016/j.tet.2006.12.082
OC(=O)CN1C(=S)S/C(=C\ c2ccc(s2)c2ccc(s2)c2ccc3c(c2)c2ccccc2n3c2ccc3c(c2)C(C)(C)c2c3cccc2)/C1=O	ethanol	17	10.1016/j.tet.2006.12.082
N#C/C(=C\ c1ccc(s1)c1ccc2c(c1)c1ccccc1n2c1ccc(cc1)C=C(c1ccccc1)c1ccccc1)/C(=O)O	ethanol	-2	10.1016/j.tet.2006.12.082
N#C/C(=C\c1ccc(s1)/C=C/ c1ccc(s1)c1ccc2c(c1)c1ccccc1n2c1ccc2c(c1)C(C)(C)c1c2cccc1)/C(=O)O	ethanol	11	10.1016/j.tet.2006.12.082
N#C/C(=C\ c1cn(c2c1cccc2)c1ccc(cc1)N(c1ccccc1)c1ccccc1)/C(=O)O	ethanol	-10	10.1016/ j.synthmet.2015.10.024
CCCCCCN1c2ccc(cc2Sc2c1cccc2)n1cc(c2c1cccc2)/C=C(/C(=O)O)\C#N	ethanol	-4	10.1016/ j.synthmet.2015.10.024
CCCCCCn1c2ccc(cc2c2c1cccc2)n1cc(c2c1cccc2)/C=C(/C(=O)O)\C#N	ethanol	-1	10.1016/ j.synthmet.2015.10.024
N#C/C(=C\ c1ccc(s1)c1ccc(s1)c1cc2CCC[C@H]3c2c(c1)[C@H](c1ccc2c(c1)C(C)(C)c1c2cccc1)N3c1ccccc1)/C(=O)O	ethanol	2	10.1016/ j.jphotochem.2008.10.011
N#C/C(=C\ c1ccc(s1)c1ccc(s1)c1ccc2c3c1CCC[C@H]3N([C@@H]2c1ccc2c(c1)C(C)(C)c1c2cccc1)c1ccccc1)/C(=O)O	ethanol	11	10.1016/ j.jphotochem.2008.10.011
CCCCn1c(/C=C/ c2ccc(n2c2ccc(cc2)c2ccc(cc2)N(c2ccccc2)c2ccccc2)/C=C(/C(=O)O)\ C#N)cc2c1ccc(c2)c1ccc(cc1)N(c1ccccc1)c1ccccc1	ethanol	4	10.1021/jp906334w
CCCCCCn1c2ccc(cc2c2c1cccc2)c1ccc(cc1)n1c(ccc1/C=C(/C(=O)O)\C#N)/C=C/ c1cc2c(n1CCCC)ccc(c2)c1ccc2c(c1)c1ccccc1n2CCCCCC	ethanol	0	10.1021/jp906334w
C(#N)C(C(=O)O)=CC=1SC(=CC1)C=CC=1C=C2C(CC(N(C2=CC1)C)(C)C)C	ethanol	8	10.1021/cm070617g
N#C/C(=C\c1ccc(s1)c1ccc(s1)/C=C/ c1ccc2c(c1)[C@@H](C)CC(N2C)(C)C)/	ethanol	14	10.1021/cm070617g

SMILES	SOLVENT	SHIFT (nm)	DOI
C(=O)O			
N#C/C(=C\c1ccc(s1)c1ccc(s1)c1ccc(s1)/C=C/c1ccc2c(c1)[C@@H](C)CC(N2C)(C)C)/C(=O)O	ethanol	62	10.1021/cm070617g
N#C/C(=C\c1cc2c(s1)c1c(s2)cc(s1)/C=C/c1ccc2c(c1)[C@@H](C)CC(N2C)(C)C)/C(=O)O	ethanol	10	10.1021/cm070617g
N#C/C(=C\c1ccc(s1)/C=C/c1ccc(s1)/C=C/c1ccc2c(c1)[C@@H](C)CC(N2C)(C)C)/C(=O)O	ethanol	70	10.1021/cm070617g
N#C/C(=C\c1ccc(s1)c1ccc2c(c1)[C@@H](C)CC(N2C)(C)C)/C(=O)O	ethanol	4	10.1021/cm070617g
N#C/C(=C\c1ccc(s1)c1ccc(s1)c1ccc2c(c1)[C@@H](C)CC(N2C)(C)C)/C(=O)O	ethanol	9	10.1021/cm070617g
N#C/C(=C\c1ccc(s1)c1ccc(s1)c1ccc(s1)c1ccc2c(c1)[C@@H](C)CC(N2C)(C)C)/C(=O)O	ethanol	44	10.1021/cm070617g
N#C/C(=C\c1cc2c(s1)c1c(s2)cc(s1)c1ccc2c(c1)[C@@H](C)CC(N2C)(C)C)/C(=O)O	ethanol	7	10.1021/cm070617g
N#C/C(=C\c1ccc(s1)/C=C/c1ccc2c(c1)[C@H](C)CC(N2Cc1ccccc1)(C)C)/C(=O)O	ethanol	1	10.1021/cm070617g
N#C/C(=C\c1ccc(s1)/C=C/c1ccc2c(c1)[C@@H](C)CC(N2C)(C)C)/P(=O)(O)O	ethanol	8	10.1021/cm070617g
C(#N)C(C(=O)O)=CC=1SC(=CC1)C=CC=1SC(=CC1)C=CC=1C=C2C(CC(N(C2=CC1)C)(C)C)C	ethanol	70	10.1016/j.jphotochem.2007.02.018
C(#N)C(C(=O)O)=CC=1SC(=CC1)C=1C=C2C(CC(N(C2=CC1)C)(C)C)C	ethanol	4	10.1016/j.jphotochem.2007.02.018
CCCCC1(CCCC)c2cc(ccc2c2c1cc(cc2)/C=C(/C(=O)O)\C#N)N(c1ccccc1)c1ccccc1	ethanol	40	10.1021/jp305884u
CCCCC1(CCCC)c2cc(ccc2c2c1cc(cc2)/C=C(/C(=O)O)\C#N)N(c1ccc(cc1)OC)c1ccc(cc1)OC	ethanol	36	10.1021/jp305884u
CCCCC1(CCCC)c2cc(ccc2c2c1cc(cc2)/C=C(/C(=O)O)\C#N)N(c1ccc(cc1)OCCOC(C(F)(F)F)(C(F)(F)F)C(F)(F)F)c1ccc(cc1)OCCOC(C(F)(F)F)(C(F)(F)F)C(F)(F)F	ethanol	-8	10.1021/jp305884u
N#C/C(=C\c1ccc2c(c1)C(CCCOC(C(F)(F)F)(C(F)(F)F)C(F)(F)F)(CCCOC(C(F)(F)F)(C(F)(F)F)C(F)(F)F)c1c2ccc(c1)N(c1ccccc1)c1ccccc1)/C(=O)O	ethanol	-15	10.1021/jp305884u
N#C/C(=C\c1ccc2c(c1)C(CCCOC(C(F)(F)F)(C(F)(F)F)C(F)(F)F)(CCCOC(C(F)(F)F)(C(F)(F)F)C(F)	ethanol	4	10.1021/jp305884u

SMILES	SOLVENT	SHIFT (nm)	DOI
(F)F)c1c2ccc(c1)N(c1ccc(cc1)OCCOC(C(F)(F)F)(C(F)(F)F)C(F)(F)F)c1ccc(cc1)OCCOC(C(F)(F)F)(C(F)(F)F)C(F)(F)F)/C(=O)O			
CCCCC1(CCCC)c2cc(ccc2c2c1cc(cc2)C(=O)O)N(c1ccccc1)c1ccccc1	ethanol	-73	10.1021/jp305884u
N#C/C(=C/c1ccc(s1)c1ccc(c2c1nsn2)C#Cc1ccc(s1)c1cc2cc3c4c(c2oc1=O)C(C)(C)CCN4CCC3(C)C)/C(=O)O	ethanol	46	10.1016/j.jpowsour.2017.01.115
N#C/C(=C/c1ccc(cc1)c1ccc(c2c1nsn2)C#Cc1ccc(s1)c1cc2cc3c4c(c2oc1=O)C(C)(C)CCN4CCC3(C)C)/C(=O)O	ethanol	12	10.1016/j.jpowsour.2017.01.115
N#C/C(=C\c1ccc(cc1)N(c1ccc(cc1)c1ccccc1)c1ccc(cc1)c1ccccc1)/C(=O)O	ethanol	-4	10.1016/j.electacta.2017.08.089
N#C/C(=C\c1ccc(cc1)N(c1ccc(cc1)/C=C/c1ccccc1)c1ccc(cc1)/C=C/c1ccccc1)/C(=O)O	ethanol	0	10.1016/j.electacta.2017.08.089
N#C/C(=C\c1ccc(cc1)N(c1ccc(cc1)C#Cc1ccccc1)c1ccc(cc1)C#Cc1ccccc1)/C(=O)O	ethanol	-3	10.1016/j.electacta.2017.08.089
CCCCCCCC[N+]1=C(/C=C\2/C(=C(C2=O)/C=c/2\n(CCCCCC)c3c4c2cccc4ccc3)[O-])C(c2c1ccc(c2)C(=O)O)(C)C	ethanol	-14	10.1021/acs.joc.8b00070
CCCCCCCC[N+]1=C(/C=C\2/C(=C(C2=O)/C=c/2\n(CCCCCC)c3c4c2cccc4ccc3)[O-])C(c2c1ccc1c2ccc(c1)C(=O)O)(C)C	ethanol	-16	10.1021/acs.joc.8b00070
CCCCCCn1/c(=C\C2=C([O-])/C(=C/C3=[N+](CCCCCCCC)c4c(C3(C)C)c3ccc(cc3cc4)C(=O)O)/C2=O)/c2c3c1ccc(c3ccc2)C[C@@H](CCCC)CC	ethanol	-18	10.1021/acs.joc.8b00070
CCCCCCn1/c(=C\C2=C([O-])/C(=C/C3=[N+](CCCCCCCC)c4c(C3(C)C)c3ccc(cc3cc4)C(=O)O)/C2=O)/c2c3c1ccc(c3ccc2)OCCCC	ethanol	-15	10.1021/acs.joc.8b00070
CCCCCCn1/c(=C\C2=C([O-])/C(=C/C3=[N+](CCCCCCCC)c4c(C3(C)C)c3ccc(cc3cc4)C(=O)O)/C2=O)/c2c3c1ccc(c3ccc2)N(CCCC)CCCC	ethanol	-9	10.1021/acs.joc.8b00070
CCCCCCCCN(c1ccc2c3c1cccc3/c(=C/C1=C([O-])/C(=C/C3=[N+](CCCCCCCC)c4c(C3(C)C)c3ccc(cc3cc4)C(=O)O)/C1=O)/n2CCCCCC)CCCCCCCC	ethanol	-10	10.1021/acs.joc.8b00070
CCCCCCn1/c(=C\C2=C([O-])/C(=C/C3=[N+](CCCCCCCC)c4c(C3(C)C)c3ccc(cc3cc4)C(=O)	ethanol	-8	10.1021/acs.joc.8b00070

SMILES	SOLVENT	SHIFT (nm)	DOI
O)/C2=O)/c2c3c1ccc(c3ccc2)N(C[C@H](CCCC)CC)C[C@H](CCCC)CC			
COc1ccc(cc1)[C@H](C1=C(O)C(=O)c2c(C1=O)cccc2)Nc1ccc(cc1)C	ethanol	-12	10.1016/j.molstruc.2018.05.014
Cc1ccc(cc1)[C@H](C1=C(O)C(=O)c2c(C1=O)cccc2)Nc1ccc(cc1)C	ethanol	-18	10.1016/j.molstruc.2018.05.014
Brc1ccc(cc1)[C@H](C1=C(O)C(=O)c2c(C1=O)cccc2)Nc1ccc(cc1)C	ethanol	-11	10.1016/j.molstruc.2018.05.014
Clc1ccc(cc1)[C@H](C1=C(O)C(=O)c2c(C1=O)cccc2)Nc1ccc(cc1)C	ethanol	-19	10.1016/j.molstruc.2018.05.014
Clc1ccc(c(c1)Cl)[C@H](C1=C(O)C(=O)c2c(C1=O)cccc2)Nc1ccc(cc1)C	ethanol	-9	10.1016/j.molstruc.2018.05.014
N#C/C(=C\c1ccc(c(c1)O)O)/c1cccs1	ethanol	-199	10.1021/jp105687z
N#C/C(=C\c1ccc(c(c1)O)O)/c1ccc(s1)c1cccs1	ethanol	-185	10.1021/jp105687z
N#C/C(=C\c1ccc(c(c1)O)O)/c1ccc(s1)c1ccc(s1)c1cccs1	ethanol	-207	10.1021/jp105687z
CCn1c2ccc(cc2c2c1cccc2)c1ccc(s1)C=C1C(=O)NC(=O)NC1=O	ethanol	-38	10.3998/ark.5550190.p008.686
CCn1c2ccc(cc2c2c1cccc2)c1ccc(cc1)C=C1C(=O)NC(=O)NC1=O	ethanol	-6	10.3998/ark.5550190.p008.686
C(#N)C(C(=O)O)=CC=1SC(=CC1)C=1C=CC=2N(C3=CC=CC=C3C2C1)CC	ethanol	-15	10.3998/ark.5550190.p008.686
N#C/C(=C\c1ccc(cc1)c1ccc2c(c1)c1ccccc1n2CC)/C(=O)O	ethanol	-56	10.3998/ark.5550190.p008.686
c1ccc(cc1)N(c1ccccc1)c1ccc(cc1)C=C(c1[nH]nnn1)c1[nH]nnn1	ethanol	-56	10.1007/s13738-017-1096-y
c1ccc(cc1)N(c1ccc(cc1)C=C(c1nnn[nH]1)c1nnn[nH]1)c1ccc(cc1)C=C(c1nnn[nH]1)c1nnn[nH]1	ethanol	-45	10.1007/s13738-017-1096-y
CN1C2=CC=CC=C2C(=O)\C1=C1/N(C)C2=C(C=C(NC3=CC=C4N(C5=CC=CC=C5)C5=CC=C(\C=C(/C#N)C(O)=O)C=C5SC4=C3)C=C2)C1=O	ethanol	-15	10.1179/1753555715Y.0000000063
OC(=O)C(=C\C1=CC=C2N(C3=CC=CC=C3)C3=CC=C(NC4=CC5=C(S\C(C5=O)=C5\SC6=CC=CC=C6C5=O)C=C4)C=C3SC2=C1)\	ethanol	-25	10.1179/1753555715Y.0000000063

SMILES	SOLVENT	SHIFT (nm)	DOI
C#N			
OC(=O)CN1C(=S)S/C(=C\c2ccc(cc2)N(c2ccc(cc2)c2cccc3c2[nH]c2c3cccc2)c2ccccc2)/C1=O	ethanol	-34	10.1021/jp106275v
OC(=O)CN1C(=S)S/C(=C\c2ccc(cc2)N(c2ccc(cc2)c2cccc3c2[nH]c2c3cccc2)c2ccc(cc2)/C=C\2/SC(=S)N(C2=O)CC(=O)O)/C1=O	ethanol	-48	10.1021/jp106275v
OC(=O)CN1C(=S)S/C(=C\c2ccc(cc2)N(c2ccc(cc2)c2cccc3c2[nH]c2c3cccc2)c2ccc(cc2)c2cccc3c2[nH]c2c3cccc2)/C1=O	ethanol	-33	10.1021/jp106275v
COc1ccc(cc1)C1N=C(NC1c1ccc(cc1)OC)c1ccc(cc1)N(c1ccc(cc1)C=C(C(=O)O)C#N)c1ccccc1	ethanol	-25	10.1016/j.dyepig.2013.12.024
N#CC(=Cc1ccc(cc1)N(c1ccccc1)c1ccc(cc1)C1=NC(C(N1)c1ccc(cc1)c1cccs1)c1ccc(cc1)c1cccs1)C(=O)O	ethanol	-30	10.1016/j.dyepig.2013.12.024
N#C/C(=C/c1cc2cc3c4c(c2oc1=O)C(C)(C)CCN4CCC3(C)C)/C(=O)O	ethanol	32	10.1002/adma.200601020
N#C/C(=C\c1ccc(s1)/C=C/c1cc2cc3c4c(c2oc1=O)C(C)(C)CCN4CCC3(C)C)/C(=O)O	ethanol	30	10.1021/jp806927b
N#C/C(=C\c1ccc(s1)/C(=C/c1cc2cc3c4c(c2oc1=O)C(C)(C)CCN4CCC3(C)C)/C#N)/C(=O)O	ethanol	46	10.1021/jp806927b
N#C/C(=C\1/C=C(/C=C/c2cc3cc4c5c(c3oc2=O)C(C)(C)CCN5CCC4(C)C)CC(C1)(C)C)/C(=O)O	ethanol	5	10.1021/jp044851v
CCCCCCC1(CCCCCC)c2cc(sc2c2c1cc(s2)c1ccc(cc1)N(c1ccc(cc1)c1ccc(cc1OCCCC)OCCCC)c1ccc(cc1)c1ccc(cc1OCCCC)OCCCC)/C=C(/C(=O)O)\C#N	ethanol	62	10.1039/C4TA05774K
CCCCCCC1(CCCCCC)c2cc(sc2c2c1cc(s2)c1ccc(cc1)N(c1ccc2c(c1)C(C)(C)c1c2c(OCCCC)cc(c1)OCCCC)c1ccc2c(c1)Cc1c2c(OCCCC)cc(c1)OCCCC)/C=C(/C(=O)O)\C#N	ethanol	43	10.1039/C4TA05774K
CCCCCCn1c2ccccc2c2c1ccc(c2)C=Cc1ccc(cc1)C=C1SC(=S)N(C1=O)CC(=O)O	ethanol	-60	10.1039/C5TA06548H
CCCCCCn1c2ccc(cc2c2c1cccc2)/C=C/c1ccc(cc1)C=C(C(=O)O)C#N	ethanol	-10	10.1039/C5TA06548H
N#CC(=Cc1ccc(s1)c1ccc(cc1)c1n(CC)c(=C)c2c1c(=C)n(c2c1ccc(cc1)N(c1ccccc1)c1ccccc1)	ethanol	-32	10.1039/C8NJ00847G

SMILES	SOLVENT	SHIFT (nm)	DOI
CC)C(=O)O			
N#CC(=Cc1ccc(s1)c1ccc(cc1)c1n(CC)c(=C)c2c1c(=C)n(c2c1ccc(cc1)n1c2CCCc2c2c1cccc2)CC)C(=O)O	ethanol	-22	10.1039/C8NJ00847G
N#CC(=Cc1ccc(s1)c1ccc(cc1)c1n(CC)c(=C)c2c1c(=C)n(c2c1ccc(cc1)n1c2ccccc2c2c1cccc2)CC)C(=O)O	ethanol	-25	10.1039/C8NJ00847G
[O-]C(=O)CC[N+]1=C(/C=C/C2=C(Cl)/C(=C/C=C/3\N(CCC(=O)O)c4c(C3(C)C)c3ccccc3cc4)/CCC2)C(c2c1ccc1c2cccc1)(C)C	ethanol	65	10.1016/j.dyepig.2015.07.003
CCCCCCCC[N+]1=C(/C=C\2/C(=C(C2=O)C=C2N(CC)c3c(C2(C)C)cccc3)[O-])C(c2c1ccc(c2)C(=O)O)(C)C	ethanol	-9	10.1039/c2cc17187b
CCCCCCCCN1c2ccc(cc2C(/C/1=C\C1=C([O-])/C(=C\C2=[N+](CCCCCCCC)c3c(C2(C)C)cc(cc3)C(=O)O)/C1=O)(C)C)C(=O)O	ethanol	-11	10.1039/c2cc17187b
CCCCCCCC[N+]1=C(/C=C/2\C(=C(C2=O)c2ccc(n2C)c2ccc(s2)c2ccc(cc2)N(c2ccc3c(c2)C(C)(C)c2c3cccc2)c2ccc3c(c2)C(C)(C)c2c3cccc2)[O-])C(c2c1ccc(c2)C(=O)O)(C)C	ethanol	19	10.1039/c0cc05378c
CCCCCCCC[N+]1=C(/C=C/2\C(=C(C2=O)c2ccc(n2C)c2ccc(s2)c2ccc(cc2)N(c2ccc3c(c2)C(C)(C)c2c3ccc(c2)OCCCCCC)c2ccc3c(c2)C(C)(C)c2c3ccc(c2)OCCCCCC)[O-])C(c2c1ccc(c2)C(=O)O)(C)C	ethanol	32	10.1039/c0cc05378c
CCCCCC[N+]1=C(C=C2C(=C(C2=O)C=c2sc3c(n2CCCCCC)cccc3)[O-])C(c2c1ccc(c2)C(=O)O)(C)C	ethanol	-8	10.1021/la101257b
CCCCCC[N+]1=C(C=C2C(=C(C2=O)C=c2sc3c(n2CCCCCC)cc(cc3)OC)[O-])C(c2c1ccc(c2)C(=O)O)(C)C	ethanol	-13	10.1021/la101257b
CCCC[N+]1=C(C=C2C(=C(C2=O)c2sc(c3c2OCCO3)c2ccc(cc2)N(c2ccc(cc2)OCCCCCC)c2ccc(cc2)OCCCCCC)[O-])C(c2c1ccc(c2)C(=O)O)(C)C	ethanol	30	10.1021/ol102127x
CCCC[N+]1=C(C=C2C(=C(C2=O)c2ccc(s2)c2ccc(s2)c2ccc(cc2)N(c2ccc(cc2)OCCCCCC)c2ccc(cc2)OCCCCCC)[O-])C(c2c1ccc(c2)C(=O)O)(C)C	ethanol	-20	10.1021/ol102127x
CCCCCCCCCCCC[NH+]1c2ccc(cc2C(/C/1=C\	ethanol	23	10.1002/anie.201101362

SMILES	SOLVENT	SHIFT (nm)	DOI
C1=C([O-])/C(=C\C2=[N+](CC)c3c(C2(C)C)cccc3)/C1=O)(C)C)c1ccc(s1)C=C(C(=O)O)C#N			
C1(=CC=CC=C1)N(C1=CC=C(C=C1)C1=CC=C(S1)C1=CC=C(S1)/C=C(/C(=O)O)\C#N)C1=CC=CC=C1	ethanol	8	10.1021/jp9033722
C(CCCCC)OC1=CC=C(C=C1)N(C1=CC=C(C=C1)C1=CC=C(S1)C1=CC=C(S1)/C=C(/C(=O)O)\C#N)C1=CC=C(C=C1)OCCCCCC	ethanol	8	10.1021/jp9033722
C(CCCCCCCCCCC)OC1=CC=C(C=C1)N(C1=CC=C(C=C1)C1=CC=C(S1)C1=CC=C(S1)/C=C(/C(=O)O)\C#N)C1=CC=C(C=C1)OCCCCCCCCCCCC	ethanol	5	10.1021/jp9033722
CCCCCCc1ccc(s1)c1sc(cc1CCCCCC)c1ccc(c2c1nsn2)c1cc(c(s1)c1ccc(s1)P(=O)(O)O)CCCCCC	ethanol	-9	10.1021/acsami.5b11134
CCCCCCCCN1c2ccccc2C(/C/1=C\C1=C([O-])/C(=C\C2=[N+](CCCCCCCC)c3c(C2)cc(cc3)C(=O)O)/C1=C(C(=O)OCC)C#N)(C)C	ethanol	-5	10.1021/acsami.5b11134
CCCCCCCCN1c2ccc(cc2C(/C/1=C\C1=C([O-])/C(=C\C2=[N+](CCCCCCCC)c3c(C2)cc(cc3)C(=O)O)/C1=C(C(=O)OCC)C#N)(C)C)C(=O)O	ethanol	-7	10.1021/acsami.5b11134
CCCCCCOC1=CC=C2C3=C(C=C(C=C3)N(C3=CC=C(C=C3)C3=CC=C(S3)C3=CC=C(S3)\C=C(/C#N)C(O)=O)C3=CC4=C(C=C3)C3=CC=C(OCCCCCC)C=C3C4(C)C)C(C)(C)C2=C1	ethanol	42	10.1002/chem.201300736
C(CCCCC)OC1=CC=C2C=3C=CC(=CC3C(C2=C1)(C)C)N(C=1C=C2C(C3=C(SC(=C3)C3=CC=C(S3)/C=C(/C(=O)O)\C#N)C2=CC1)(C)C)C1=CC=2C(C3=CC(=CC=C3C2C=C1)OCCCCCC)(C)C	ethanol	71	10.1002/chem.201300736
C(CCCCC)OC1=CC=C2C=3C=CC(=CC3C(C2=C1)(C)C)N(C1=CC=2C(C3=C(SC4=C3SC(=C4)C4=CC=C(S4)/C=C(/C(=O)O)\C#N)C2C=C1)(C)C)C1=CC=2C(C4=CC(=CC=C4C2C=C1)OCCCCCC)(C)C	ethanol	73	10.1002/chem.201300736
OC(=O)C(=C/C1=CC=C(\C=C\C2=CC=C(C=C2)N(C2=CC=CC=C2)C2=CC=CC=C2)N1C1=CC=C(C=C1)C1=CC=C(C=C1)N(C1=CC=CC=C1)C1=CC=CC=C1)\C#N	ethanol	-1	10.1002/chem.200901150

SMILES	SOLVENT	SHIFT (nm)	DOI
CCCCCCN1C2=CC=CC=C2C2=CC(=CC=C12)C1=CC=C(C=C1)N1C(\C=C\C2=CC=C(C=C2)N(C2=CC=CC=C2)C2=CC=CC=C2)=CC=C1\C=C(\C#N)C(O)=O	ethanol	-16	10.1002/chem.200901150
N#C/C(=C\c1ccc(s1)c1ccc(cc1)N(c1ccc(cc1)c1ccc(cc1)N(C)C)c1ccc(cc1)c1ccc(cc1)N(C)C)/C(=O)O	ethanol	26	10.1039/B911397P
CCCCOc1ccc(c(c1)OCCCC)c1ccc(cc1)N(c1ccc(cc1)c1ccc(cc1OCCCC)OCCCC)c1ccc(cc1)c1ccc(s1)/C=C(/C(=O)O)\C#N	ethanol	1	10.1039/B911397P
CCCCOc1ccc(c(c1)OCCCC)c1ccc(cc1)N(c1ccc(cc1)c1ccc(s1)/C=C(/C(=O)O)\C#N)c1ccc(cc1)c1ccc(cc1)N(C)C	ethanol	13	10.1039/B911397P
CCN(c1ccc(cc1)N=Nc1ccccc1C(=O)O)CC	ethanol	4	10.3390/ma9100813
OCC1OC(C(C(C1O)O)O)c1c(O)c2c(c(c1O)O)C(=O)c1c(C2=O)c(C)c(c(c1)O)C(=O)O	ethanol	-31	10.3390/ma9100813
N#C/C(=C\c1ccc(s1)C#Cc1c2ccccc2c(c2c1cccc2)c1ccc(s1)N(c1ccccc1)c1ccccc1)/C(=O)O	ethanol	10	10.5012/bkcs.2013.34.4.1081
N#C/C(=C\c1ccc(s1)c1ccc(s1)C#Cc1c2ccccc2c(c2c1cccc2)c1ccc(s1)N(c1ccccc1)c1ccccc1)/C(=O)O	ethanol	37	10.5012/bkcs.2013.34.4.1081
OB(O)C1=C2C=CC=CC2=CC2=CC(=CC=C12)C1=CC=C(C2=CC=C(O2)\C=C2\SC(=S)N(CC(O)=O)C2=O)C2=NSN=C12	ethanol	-102	10.1016/j.jphotochem.2018.12.004
OB(O)C1=CC=C(S1)C1=CC=C(S1)C1=CC=C(S1)C1=CC=C(C2=CC=C(O2)\C=C2\SC(=S)N(CC(O)=O)C2=O)C2=NSN=C12	ethanol	-86	10.1016/j.jphotochem.2018.12.004
OB(O)C1=C2C=CC3=CC=CC4=C3C2=C(C=C1)C=C4C1=CC=C(C2=CC=C(O2)\C=C2\SC(=S)N(CC(O)=O)C2=O)C2=NSN=C12	ethanol	-95	10.1016/j.jphotochem.2018.12.004
CCCCCCOC1=CC=C(C=C1)N(C1=CC=C(OCCCCC)C=C1)C1=CC=C(\C=C\C2=CC=C(S2)\C=C(/CC#N)C(O)=O)C=C1	ethanol	67	10.1002/ejoc.201801497
CCCCCCOC1=CC=C(C=C1)N(C1=CC=C(OCCCCC)C=C1)C1=CC=C(\C=C\C2=CC=C(S2)\C=C(/CC#N)C(S)=O)C=C1	ethanol	44	10.1002/ejoc.201801497
CCCCCCOC1=CC=C(C=C1)N(C1=CC=C(OCCCCC)C=C1)C1=CC=C(\C=C\C2=CC=C(S2)\C=C(/CC#N)C(S)=S)C=C1	ethanol	22	10.1002/ejoc.201801497
OC(=O)C(=C/C1=CC=C(C=C1)N(C1=CC=CC=C1)C1=CC=CC=C1)\C#N	methanol	-11	10.1039/C4RA04782F
N#C/C(=C\	methanol	1	10.1039/C4RA04782F

SMILES	SOLVENT	SHIFT (nm)	DOI
c1ccc(cc1)N(c1ccccc1)c1ccc(cc1)/C=C/1\SC(=C(S1)SC)SC)/C(=O)O			
CSC1=C(SC)S/C(=C\c2ccc(cc2)N(c2ccc(cc2)/C=C(/C(=O)O)\C#N)c2ccc(cc2)/C=C(/C(=O)O)\C#N)/S1	methanol	-1	10.1039/C4RA04782F
CCCCCn1c2ccc(cc2c2c1cccc2)N1c2ccc(cc2[C@@H]2[C@H]1CCC2)c1ccc(s1)/C=C\1/SC(=S)N(C1=O)CC(=O)O	methanol	4	10.1016/j.dyepig.2018.03.072
CCCCCCCCn1c(=O)/c(=C/c2ccc(s2)c2ccc3c(c2)[C@H]2CCC[C@H]2N3c2ccc3c(c2)c2ccccc2n3CCCCC)/s/c/1=C(\C(=O)O)/C#N	methanol	2	10.1016/j.dyepig.2018.03.072
CCCCCn1c2ccc(cc2c2c1cccc2)N1c2ccc(cc2[C@@H]2[C@H]1CCC2)c1ccc(cc1)/C=C/1\SC(=S)N(C1=O)CC(=O)O	methanol	2	10.1016/j.dyepig.2018.03.072
CCCCCCCCn1c(=O)/c(=C\c2ccc(cc2)c2ccc3c(c2)[C@H]2CCC[C@H]2N3c2ccc3c(c2)c2ccccc2n3CCCCC)/s/c/1=C(\C(=O)O)/C#N	methanol	1	10.1016/j.dyepig.2018.03.072
N#C/C(=C/c1ccc(cc1)N(c1ccccc1)c1ccc(cc1)C=C1SC(=C(S1)SC)SC)/C(=O)O	methanol	-21	10.1039/C5RA06774J
CCCCCCSC1=C(SCCCCCC)SC(=Cc2ccc(cc2)N(c2ccc(cc2)/C=C(\C(=O)O)/C#N)c2ccccc2)S1	methanol	-8	10.1039/C5RA06774J
CCCCCCCCCCSC1=C(SCCCCCCCCCC)SC(=Cc2ccc(cc2)N(c2ccc(cc2)/C=C(\C(=O)O)/C#N)c2ccccc2)S1	methanol	-2	10.1039/C5RA06774J
N#C/C(=C\c1ccc(s1)c1ccc(s1)c1ccc2c(c1)C(=O)c1c2cccc1)/C(=O)O	THF	-29	10.1016/j.dyepig.2013.03.007
CCCCC1(CCCC)c2cc(ccc2c2c1cccc2)c1ccc2c(c1)C(=O)c1c2ccc(c1)c1ccc(s1)/C=C(/C(=O)O)\C#N	THF	-13	10.1016/j.dyepig.2013.03.007
CCCCC1(CCCC)c2cc(ccc2c2c1cccc2)c1ccc2c(c1)C(=O)c1c2ccc(c1)c1ccc(s1)c1ccc(s1)/C=C(/C(=O)O)\C#N	THF	-23	10.1016/j.dyepig.2013.03.007
N#C/C(=C\c1ccc(s1)c1ccc(s1)c1ccc2c(c1)C(=O)c1c2ccc(c1)c1ccc(cc1)N(c1ccc(cc1)C)c1ccc(cc1)C)/C(=O)O	THF	-11	10.1016/j.dyepig.2013.03.007
CCCCCCCCCCc1csc(n1)c1nc(c(s1)c1ccc(s1)/C=C(/C(=O)O)\C#N)CCCCCCCCC	THF	14	10.1016/j.dyepig.2012.10.002
CCCCCCCCCCc1nc(sc1c1cccs1)c1nc(c(s1)c1ccc(s1)/C=C(/C(=O)O)\C#N)CCCCCCCCC	THF	19	10.1016/j.dyepig.2012.10.002

SMILES	SOLVENT	SHIFT (nm)	DOI
CCCCCCCCCCc1nc(sc1c1ccc(cc1)N(c1ccc(cc1)C)c1ccccc1)c1nc(c(s1)c1ccc(s1)/C=C(/C(=O)O)\C#N)CCCCCCCCC	THF	16	10.1016/j.dyepig.2012.10.002
CCCCCCCCCCc1nc(sc1c1ccc2c(c1)n(CCCC)c1c2cccc1)c1nc(c(s1)c1ccc(s1)/C=C(/C(=O)O)\C#N)CCCCCCCCC	THF	8	10.1016/j.dyepig.2012.10.002
CCCCCCCCCCc1nc(sc1c1ccc2c(c1)C(CCCC)(CCCC)c1c2cccc1)c1nc(c(s1)c1ccc(s1)/C=C(/C(=O)O)\C#N)CCCCCCCCC	THF	12	10.1016/j.dyepig.2012.10.002
N#C/C(=C\c1ccc(cc1)/C=C/c1ccc(cc1)N(c1ccccc1)c1ccccc1)/C(=O)O	THF	33	10.1016/j.jphotochem.2011.12.013
N#C/C(=C\c1ccc(cc1F)/C=C/c1ccc(cc1)N(c1ccccc1)c1ccccc1)/C(=O)O	THF	37	10.1016/j.jphotochem.2011.12.013
N#C/C(=C\c1c(F)cc(cc1F)/C=C/c1ccc(cc1)N(c1ccccc1)c1ccccc1)/C(=O)O	THF	42	10.1016/j.jphotochem.2011.12.013
N#C/C(=C\c1ccc(cc1)/C=C/c1ccc(cc1)N(c1cccc2c1cccc2)c1ccccc1)/C(=O)O	THF	28	10.1016/j.jphotochem.2011.12.013
N#C/C(=C\c1ccc(cc1F)/C=C/c1ccc(cc1)N(c1cccc2c1cccc2)c1ccccc1)/C(=O)O	THF	20	10.1016/j.jphotochem.2011.12.013
N#C/C(=C\c1c(F)cc(cc1F)/C=C/c1ccc(cc1)N(c1cccc2c1cccc2)c1ccccc1)/C(=O)O	THF	23	10.1016/j.jphotochem.2011.12.013
CCCCCCC1(CCCCCC)c2cc(ccc2c2c1cc(cc2)c1sc2c(c1)n(c1c2sc(c1)/C=C(/C(=O)O)\C#N)CCCCCC)c1sc2c(c1)n(c1c2sc(c1)/C=C(/C(=O)O)\C#N)CCCCCC	THF	6	10.1016/j.orgel.2013.04.046
CCCCCCC1(CCCCCC)c2cc(ccc2c2c1cc(cc2)c1sc2c(c1)n(c1c2sc(c1)/C=C(/C(=O)O)\C#N)c1ccc(cc1)OC)c1sc2c(c1)n(c1c2sc(c1)/C=C(/C(=O)O)\C#N)c1ccc(cc1)OC	THF	22	10.1016/j.orgel.2013.04.046
CCCCCCC1(CCCCCC)c2cc(ccc2c2c1cc(cc2)c1sc2c(c1)n(c1c2sc(c1)/C=C(/C(=O)O)\C#N)c1ccc(cc1)N(c1ccc(cc1)C)c1ccc(cc1)C)c1sc2c(c1)n(c1c2sc(c1)/C=C(/C(=O)O)\C#N)c1ccc(cc1)N(c1ccc(cc1)C)c1ccc(cc1)C	THF	6	10.1016/j.orgel.2013.04.046
N#C/C(=C\c1ccc(s1)/C=N/N(c1ccccc1)c1ccccc1)/C(=O)O	THF	-4	10.1016/j.dyepig.2011.08.014
N#C/C(=C\c1sc(c(c1OC)OC)/C=N/N(c1ccccc1)c1ccccc1)/C(=O)O	THF	3	10.1016/j.dyepig.2011.08.014
N#C/C(=C\c1sc(c2c1OCCO2)/C=N/N(c1ccccc1)c1ccccc1)/C(=O)O	THF	15	10.1016/j.dyepig.2011.08.014
CCCCCCCCOc1c(/C=C(/C(=O)O)\	THF	-13	10.1016/

SMILES	SOLVENT	SHIFT (nm)	DOI
C#N)sc(c1OCCCCCCCC)/C=N/N(c1ccccc1)c1ccccc1			j.dyepig.2011.08.014
CN(c1ccc(cc1)C#Cc1c2ccccc2c(c2c1cccc2)C#Cc1ccc(cc1)C(=O)O)C	THF	50	10.1021/acs.jpcc.5b08101
CCCCCCCCN(c1ccc(cc1)C#Cc1c2ccccc2c(c2c1cccc2)C#Cc1ccc(cc1)C(=O)O)CCCCCCCC	THF	30	10.1021/acs.jpcc.5b08101
CCCCCCCCN(c1ccc(cc1)C#Cc1c2ccccc2c(c2c1cccc2)C#Cc1ccc(c(c1)F)C(=O)O)CCCCCCCC	THF	39	10.1021/acs.jpcc.5b08101
CCCCCCCCN(c1ccc(cc1)C#Cc1c2ccccc2c(c2c1cccc2)C#Cc1ccc(c(c1)[N+](=O)[O-])C(=O)O)CCCCCCCC	THF	24	10.1021/acs.jpcc.5b08101
CCCCCCCCN(c1ccc(cc1)C#Cc1c2ccccc2c(c2c1cccc2)C#Cc1ccc(cc1)/C=C(/C(=O)O)\C#N)CCCCCCCC	THF	27	10.1021/acs.jpcc.5b08101
CCCCn1c(nc2c1c1cccc3c1c1c2cccc1cc3)c1ccc(cc1)c1ccc(s1)/C=C(/C(=O)O)\C#N	THF	-80	10.1021/ol2006874
CCCCn1c(nc2c1c1cccc3c1c1c2cccc1cc3)c1ccc(s1)c1ccc(s1)/C=C(/C(=O)O)\C#N	THF	-92	10.1021/ol2006874
CCCCn1c(nc2c1c1cccc3c1c1c2cccc1cc3)c1ccc(s1)c1ccc(s1)c1ccc(s1)/C=C(/C(=O)O)\C#N	THF	-116	10.1021/ol2006874
N#C/C(=C\c1ccc(s1)c1ccc(s1)c1ccc2c(c1)n1c3ccc(cc3c3c1c2cc(c3)C(C)(C)C)C(C)(C)C)/C(=O)O	THF	-4	10.1039/c5ra02720a
CCCCCCc1cc(sc1c1sc(c(c1)CCCCCC)/C=C(/C(=O)O)\C#N)c1ccc2c(c1)n1c3ccc(cc3c3c1c2cc(c3)C(C)(C)C)C(C)(C)C	THF	-6	10.1039/c5ra02720a
CCCCn1c2ccc(cc2c2c1ccc(c2)c1sc(c2c1OCCO2)/C=C(/C(=O)O)\C#N)c1ccc2c(c1)c1ccccc1n2CCCC	THF	0	10.1039/c2ra01002j
CCCCn1c2ccc(cc2c2c1ccc(c2)c1ccc2c(c1)c1ccccc1n2CCCC)c1ccc2c(c1)c1cc(ccc1n2CCCC)c1sc(c2c1OCCO2)/C=C(/C(=O)O)\C#N	THF	-4	10.1039/c2ra01002j
CCCCn1c2ccc(cc2c2c1ccc(c2)c1ccc2c(c1)c1cc(ccc1n2CCCC)c1sc(c2c1OCCO2)/C=C(/C(=O)O)\C#N)c1ccc2c(c1)c1cc(ccc1n2CCCC)c1ccc2c(c1)c1ccccc1n2CCCC	THF	-4	10.1039/c2ra01002j
N#C/C(=C/c1ccc(s1)c1ccc(cc1)N(c1ccc(cc1)n1c2ccccc2c2c1cccc2)c1ccc(cc1)n1c2ccccc2c2c1cccc2)/C(=O)O	THF	-72	10.1039/c0ee00008f
N#C/C(=C\	THF	-62	10.1039/c0ee00008f

SMILES	SOLVENT	SHIFT (nm)	DOI
c1ccc(s1)c1ccc(cc1)N(c1ccc(cc1)N(c1ccccc1)c1ccccc1)c1ccc(cc1)N(c1ccccc1)c1ccccc1)/C(=O)O			
N#C/C(=C\c1ccc(s1)c1ccc(cc1)N(c1ccccc1)c1ccccc1)/C(=O)O	THF	-75	10.1039/c0ee00008f
N#C/C(=C\c1sc2c(c1)C(c1c2sc(c1)c1ccc(s1)n1c2ccc(cc2c2c1ccc(c2)C(C)(C)C)C(C)(C)C)(C)C)/C(=O)O	THF	40	10.1016/j.dyepig.2013.08.016
N#C/C(=C\c1sc2c(c1)C(c1c2sc(c1)c1ccc(s1)c1ccc(s1)n1c2ccc(cc2c2c1ccc(c2)C(C)(C)C)C(C)(C)C)(C)C)/C(=O)O	THF	35	10.1016/j.dyepig.2013.08.016
CCCCCCCCc1cc(sc1c1sc2c(c1)C(c1c2sc(c1)/C=C(/C(=O)O)\C#N)(C)C)n1c2ccc(cc2c2c1ccc(c2)C(C)(C)C)C(C)(C)C	THF	30	10.1016/j.dyepig.2013.08.016
N#C/C(=C\c1cc2c(s1)c1c(C2(C)C)cc(s1)c1ccc(s1)c1ccc(cc1)N(c1ccccc1)c1ccccc1)/C(=O)O	THF	47	10.1016/j.dyepig.2013.08.016
CCCCCCn1c2ccc(cc2c2c1ccc(c2)c1ccc(s1)c1ccc(s1)/C=C(/C(=O)O)\C#N)c1ccc(s1)c1ccc(s1)/C=C(/C(=O)O)\C#N	THF	36	10.1016/j.dyepig.2011.09.012
CCCCCCn1c2ccc(cc2c2c1ccc(c2)c1ccc(s1)c1ccc(s1)c1ccc(s1)/C=C(/C(=O)O)\C#N)c1ccc(s1)c1ccc(s1)c1ccc(s1)/C=C(/C(=O)O)\C#N	THF	41	10.1016/j.dyepig.2011.09.012
CCCCCCn1c2ccc(cc2c2c1ccc(c2)c1sc(cc1CCCCCC)c1ccc(s1)/C=C(/C(=O)O)\C#N)c1sc(cc1CCCCCC)c1ccc(s1)/C=C(/C(=O)O)\C#N	THF	34	10.1016/j.dyepig.2011.09.012
CCCCCCCCC(n1c2ccc(cc2c2c1ccc(c2)c1ccc(s1)c1ccc(s1)/C=C(/C(=O)O)\C#N)c1ccc(s1)c1ccc(s1)/C=C(/C(=O)O)\C#N)CCCCCCCC	THF	23	10.1016/j.dyepig.2011.09.012
CCCCCCCCC(n1c2ccc(cc2c2c1ccc(c2)c1ccc(s1)c1ccc(s1)c1ccc(s1)/C=C(/C(=O)O)\C#N)c1ccc(s1)c1ccc(s1)c1ccc(s1)/C=C(/C(=O)O)\C#N)CCCCCCCC	THF	26	10.1016/j.dyepig.2011.09.012
CCCCCCCCC(n1c2ccc(cc2c2c1ccc(c2)c1sc(cc1CCCCCC)c1ccc(s1)/C=C(/C(=O)O)\C#N)c1sc(cc1CCCCCC)c1ccc(s1)/C=C(/C(=O)O)\C#N)CCCCCCCC	THF	21	10.1016/j.dyepig.2011.09.012

SMILES	SOLVENT	SHIFT (nm)	DOI
N#C/C(=C\c1ccc2c(c1)[C@@H]1CCC[C@H]1N2c1ccc(cc1)C)/C(=O)O	THF	84	10.1039/c1cp20484j
N#C/C(=C\c1ccc(s1)c1ccc2c(c1)[C@@H]1CCC[C@H]1N2c1ccc(cc1)C)/C(=O)O	THF	45	10.1039/c1cp20484j
N#C/C(=C\c1ccc(s1)/C=C/c1ccc2c(c1)[C@@H]1CCC[C@H]1N2c1ccc(cc1)C)/C(=O)O	THF	33	10.1039/c1cp20484j
N#C/C(=C\c1ccc(s1)/C=C/c1ccc(s1)c1ccc2c(c1)[C@@H]1CCC[C@H]1N2c1ccc(cc1)C)/C(=O)O	THF	38	10.1039/c1cp20484j
CC1=CC=C(C=C1)N1C2CCCC2C2=C1C=CC(=C2)C1=CC=C(S1)\C=C(/C#N)C(O)=O	THF	42	10.1016/j.dyepig.2013.04.031
CCCC[C@@H](Cn1nc2c(n1)c1cc(sc1c1c2cc(s1)c1ccc(cc1)N(c1ccccc1)c1ccccc1)/C=C(/C(=O)O)\C#N)CC	THF	-6	10.1039/C6TA02275H
CCCC[C@H](Cn1nc2c(n1)c1cc(sc1c1c2cc(s1)c1ccc(cc1)N(c1ccccc1)c1ccccc1)c1ccc(s1)/C=C(\C(=O)O)/C#N)CC	THF	-16	10.1039/C6TA02275H
CCCCCCc1cc(sc1/C=C(/C(=O)O)\C#N)c1sc2c(c1)c1nn(nc1c1c2sc(c1)c1ccc(cc1)N(c1ccccc1)c1ccccc1)C[C@@H](CCCC)CC	THF	-2	10.1039/C6TA02275H
CCCC[C@@H](Cn1nc2c(n1)c1cc(sc1c1c2cc(s1)c1ccc2c(c1)n(C[C@H](CCCC)CC)c1c2cccc1)c1ccc(s1)/C=C(/C(=O)O)\C#N)CC	THF	-17	10.1039/C6TA02275H
CCCCCCc1cc(sc1/C=C(/C(=O)O)\C#N)c1sc2c(c1)c1nn(nc1c1c2sc(c1)c1ccc2c(c1)n(C[C@H](CCCC)CC)c1c2cccc1)C[C@H](CCCC)CC	THF	-19	10.1039/C6TA02275H
CCCCCCc1cc(sc1/C=C(/C(=O)O)\C#N)c1sc2c(c1)c1nn(nc1c1c2sc(c1)c1cc2c(s1)c1c(n2C[C@@H](CCCC)CC)cccc1)C[C@H](CCCC)CC	THF	-15	10.1039/C6TA02275H
CCCC[C@@H](CN1c2ccc(cc2Sc2c1cccc2)c1ccc(s1)/C=C(/C(=O)O)\C#N)CC	THF	18	10.1002/asia.201301228
CCCC[C@@H](CN1c2ccc(cc2Sc2c1ccc(c2)c1ccc(s1)/C=C(/C(=O)O)\C#N)c1ccc(s1)/C=C(\C(=O)O)/	THF	32	10.1002/asia.201301228

SMILES	SOLVENT	SHIFT (nm)	DOI
C#N)CC			
CCCC[C@@H](CN1c2ccc(cc2Sc2c1ccc(c2)c1cc(c(s1)/C=C(/C(=O)O)\C#N)CCCCCC)c1cc(c(s1)/C=C(\C(=O)O)/C#N)CCCCCC)CC	THF	15	10.1002/asia.201301228
CCCC[C@@H](CN1c2ccc(cc2Sc2c1ccc(c2)c1ccc(s1)c1cc(c(s1)/C=C(/C(=O)O)\C#N)CCCCCC)c1ccc(s1)c1cc(c(s1)/C=C(\C(=O)O)/C#N)CCCCCC)CC	THF	62	10.1002/asia.201301228
N#C/C(=C\c1ccc2c(c1)Sc1c(N2C)ccc(c1)c1ccc(cc1)N(c1cccc2c1cccc2)c1ccccc1)/C(=O)O	THF	31	10.1039/c2jm13961h
CCCCCCN1c2ccc(cc2Sc2c1ccc(c2)/C=C(/C(=O)O)\C#N)c1ccc(cc1)N(c1cccc2c1cccc2)c1ccccc1	THF	3	10.1039/c2jm13961h
CCCCCCN1c2ccc(cc2Sc2c1ccc(c2)c1ccc(s1)/C=C(/C(=O)O)\C#N)c1ccc(cc1)N(c1cccc2c1cccc2)c1ccccc1	THF	12	10.1039/c2jm13961h
CCCCCCN1c2ccc(cc2Sc2c1ccc(c2)/C=C(/C(=O)O)\C#N)c1ccc(s1)c1ccc(cc1)N(c1cccc2c1cccc2)c1ccccc1	THF	3	10.1039/c2jm13961h
N#C/C(=C\c1ccc2c(c1)Sc1c(N2c2ccc(cc2)C(c2ccc(cc2)C)c2ccc(cc2)C)ccc(c1)c1ccc(cc1)N(c1cccc2c1cccc2)c1ccccc1)/C(=O)O	THF	12	10.1039/c2jm13961h
N#C/C(=C\c1ccc(s1)c1ccc2c(c1)Sc1c(N2c2ccc(cc2)N(c2ccc(cc2)C)c2ccc(cc2)C)ccc(c1)c1ccc(cc1)N(c1cccc2c1cccc2)c1ccccc1)/C(=O)O	THF	15	10.1039/c2jm13961h
N#C/C(=C\c1ccc2c(c1)Sc1c(N2c2ccc(cc2)N(c2ccc(cc2)C)c2ccc(cc2)C)ccc(c1)c1ccc(s1)c1ccc(cc1)N(c1cccc2c1cccc2)c1ccccc1)/C(=O)O	THF	12	10.1039/c2jm13961h
CCCCCCN1C2=CC=C(\C=C(/C#N)C(O)=O)C=C2SC2=C1C=CC=C2	THF	46	10.1039/c2jm13961h
C(#N)C(C(=O)O)=CC=1C=C2SC=3C=C(C=CC3N(C2=CC1)CCCCCC)C=1SC(=CC1)CCCCCC	THF	2	10.1016/j.synthmet.2014.06.009
CCCCCCc1ccc(s1)c1ccc2c(c1)Oc1c(N2CCCCCC)ccc(c1)/C=C(/C(=O)O)\C#N	THF	6	10.1016/j.synthmet.2014.06.009
C(#N)C(C(=O)O)=CC=1C=C2SC=3C=C(C=CC3N(C2=CC1)CCCCCC)C=1SC(=CC1)C=1SC(=CC1)CCCCCC	THF	4	10.1016/j.synthmet.2014.06.009

SMILES	SOLVENT	SHIFT (nm)	DOI
CCCCCCc1ccc(s1)c1ccc(s1)c1ccc2c(c1)Oc1c(N2CCCCCC)ccc(c1)/C=C(/C(=O)O)\C#N	THF	7	10.1016/j.synthmet.2014.06.009
CCCCN1c2ccc(cc2Sc2c1ccc(c2)c1nc2c(n1c1ccccc1)cccc2)c1ccc(s1)/C=C(/C(=O)O)\C#N	THF	-28	10.1039/C4RA09300C
CCCCN1c2ccc(cc2Sc2c1ccc(c2)c1nc2c(n1c1ccccc1)cccc2)c1ccc(s1)c1ccc(s1)/C=C(/C(=O)O)\C#N	THF	-27	10.1039/C4RA09300C
CCCCN1c2ccc(cc2Sc2c1ccc(c2)c1nc2c(n1c1ccccc1)cccc2)c1ccc(cc1)/C=C(/C(=O)O)\C#N	THF	-18	10.1039/C4RA09300C
CCCCN1c2ccc(cc2Sc2c1ccc(c2)c1nc2c(n1c1ccccc1)cccc2)c1ccc(s1)c1ccc(cc1)/C=C(/C(=O)O)\C#N	THF	-53	10.1039/C4RA09300C
CCCCN1c2ccc(cc2Sc2c1cccc2)c1ccc(s1)c1ccc(s1)/C=C(/C(=O)O)\C#N	THF	-54	10.1039/C4RA09300C
C(C)(C)(C)C=1C=CC=2N(C3=CC=C(C=C3C2C1)C(C)(C)C)CCCCCCN1C2=CC=C(C=C2SC=2C=CC=CC12)C=C(C(=O)O)C(=O)O	THF	-37	10.1016/j.dyepig.2014.09.032
C(C)(C)(C)C=1C=CC=2N(C3=CC=C(C=C3C2C1)C(C)(C)C)CCCCCCN1C2=CC=C(C=C2SC=2C=CC=CC12)/C=C/C(=O)O	THF	-14	10.1016/j.dyepig.2014.09.032
CCCCN1c2ccc(cc2Oc2c1cccc2)/C=C(/C(=O)O)\C#N	THF	20	10.1016/j.dyepig.2013.10.005
CCCCN1c2ccc(cc2Oc2c1ccc(c2)/C=C(/C(=O)O)\C#N)/C=C(/C(=O)O)\C#N	THF	16	10.1016/j.dyepig.2013.10.005
COc1ccc(cc1)N1c2ccc(cc2Oc2c1cccc2)/C=C(/C(=O)O)\C#N	THF	32	10.1016/j.dyepig.2013.10.005
COc1ccc(cc1)N1c2ccc(cc2Oc2c1ccc(c2)/C=C(/C(=O)O)\C#N)/C=C(/C(=O)O)\C#N	THF	4	10.1016/j.dyepig.2013.10.005
CCCCCC[C@H](Cc1cc(sc1c1cc2c(s1)c1sc(cc1c1c2non1)c1sc(cc1C[C@H](CCCCCC)CCCC)/C=C(/C(=O)O)\C#N)c1ccc(cc1)N(c1ccccc1)c1ccccc1)CCCC	THF	-22	10.1021/am5067145
CCCCCC[C@H](Cc1cc(sc1c1sc2c(c1)c1nonc1c1c2sc(c1)c1sc(cc1C[C@H](CCCCCC)CCCC)/C=C(/C(=O)O)\C#N)c1ccc2c(c1)Sc1c(N2CC)cccc1)CCCC	THF	-40	10.1021/am5067145
CCCCCC[C@@H](Cc1cc(sc1c1sc2c(c1)c1nonc1c1c2sc(c1)c1sc(cc1C[C@H](CCCCCC)CCCC)/C=C(/C(=O)O)\C#N)c1ccc2c(c1)C(CC)	THF	-24	10.1021/am5067145

SMILES	SOLVENT	SHIFT (nm)	DOI
(CC)c1c2ccc(c1)N(c1ccccc1)c1ccccc1)CCCC			
CCCCCC[C@@H](Cc1cc(sc1c1sc2c(c1)c1nonc1c1c2sc(c1)c1sc(cc1C[C@@H](CCCCCC)CCCC)c1ccc2c(c1)c1ccccc1n2CC)/C=C(/C(=O)O)\C#N)CCCC	THF	-14	10.1021/am5067145
CCCCN1c2cc(ccc2Sc2c1cccc2)/C=C(/C(=O)O)\C#N	THF	-28	10.1016/j.jpowsour.2016.05.120
CCCCN1c2cc(ccc2Sc2c1cc(cc2)/C=C(/C(=O)O)\C#N)/C=C/1\SC(=C(S1)SCCCCCC)SCCCCCC	THF	-18	10.1016/j.jpowsour.2016.05.120
CCCCN1c2cc(ccc2Sc2c1cc(cc2)/C=C(/C(=O)O)\C#N)c1ccc(cc1)/C=C/1\SC(=C(S1)SCCCCCC)SCCCCCC	THF	-31	10.1016/j.jpowsour.2016.05.120
CCCCN1c2cc(ccc2Sc2c1cc(cc2)/C=C(/C(=O)O)\C#N)c1ccc(cc1F)/C=C/1\SC(=C(S1)SCCCCCC)SCCCCCC	THF	-60	10.1016/j.jpowsour.2016.05.120
CCCCCCOc1cc(ccc1/C=C/1\SC(=C(S1)SCCCCCC)SCCCCCC)c1ccc2c(c1)N(CCCC)c1c(S2)ccc(c1)/C=C(/C(=O)O)\C#N	THF	-44	10.1016/j.jpowsour.2016.05.120
CCCCCCSC1=C(SCCCCCC)S/C(=C\c2ccc(cc2)c2ccc(cc2)c2ccc3c(c2)N(CCCC)c2c(S3)ccc(c2)/C=C(/C(=O)O)\C#N)/S1	THF	-33	10.1016/j.jpowsour.2016.05.120
CCCCN1C2=CC=CC=C2SC2=CC(\C=C(/C#N)C(O)=O)=CC=C12	THF	-28	10.1039/c6ra20769c
CCCCN1c2ccc(cc2Sc2c1cccc2)c1ccc(c2c1nsn2)c1ccc(cc1)/C=C(/C(=O)O)\C#N	THF	-11	10.1039/c6ra20769c
CCCCN1c2ccc(cc2Sc2c1ccc(c2)N(c1ccccc1)c1ccccc1)c1ccc(c2c1nsn2)c1ccc(cc1)/C=C(/C(=O)O)\C#N	THF	-3	10.1039/c6ra20769c
CCCCN1c2ccc(cc2Sc2c1ccc(c2)c1ccc(cc1)N(c1ccccc1)c1ccccc1)c1ccc(c2c1nsn2)c1ccc(cc1)/C=C(/C(=O)O)\C#N	THF	-23	10.1039/c6ra20769c
CCCCN1c2ccc(cc2Sc2c1ccc(c2)OCCCCCC)c1ccc(c2c1nsn2)c1ccc(cc1)/C=C(/C(=O)O)\C#N	THF	-19	10.1039/c6ra20769c
CCCCCCOc1cccc(c1N1c2ccc(cc2Sc2c1ccc(c2)c1sc(c(c1)CCCCCC)/C=C(/C(=O)O)\C#N)c1sc(c(c1)CCCCCC)/C=C(/C(=O)O)\C#N)OCCCCCC	THF	72	10.1039/c4cc09294e
CCCCCCOc1cccc(c1N1c2ccc(cc2Sc2c1ccc(c2)c1sc(c(n1)CCCCCC)/C=C(/C(=O)O)\C#N)c1sc(c(n1)CCCCCC)/C=C(/C(=O)O)\C#N)OCCCCCC	THF	12	10.1039/c4cc09294e

SMILES	SOLVENT	SHIFT (nm)	DOI
CCCC[C@@H](COc1cccc(c1N1c2ccc(cc2Sc2c1ccc(c2)c1sc(c(c1)CCCCCC)/C=C(/C(=O)O)\C#N)c1sc(c(c1)CCCCCC)/C=C(\C(=O)O)/C#N)OC[C@H](CCCC)CC)CC	THF	68	10.1039/c4cc09294e
CCC(CCCCN1c2ccc(cc2Sc2c1ccc(c2)c1ccc(c2c1nc(c1ccccc1)c(n2)c1ccccc1)C1=CC=C(C1)/C=C(/C(=O)O)\C#N)c1ccc(cc1)OCCCCC(CC)CC)CC	THF	-20	10.1016/j.dyepig.2017.06.068
CCC(CCCCN1c2ccc(cc2Sc2c1ccc(c2)c1ccc(c2c1nc(c1ccccc1)c(n2)c1ccccc1)C1=CC=C(C1)/C=C(/C(=O)O)\C#N)c1ccc(cc1)c1ccc2c(c1)ccc(c2)OCCCCC(CC)CC)CC	THF	-31	10.1016/j.dyepig.2017.06.068
CCC(CCCCN1c2ccc(cc2Sc2c1ccc(c2)c1ccc(c2c1nc(c1ccccc1)c(n2)c1ccccc1)C1=CC=C(C1)/C=C(/C(=O)O)\C#N)c1ccc(cc1)c1ccc2c(c1)ccc(c2c1c(OCCCCC(CC)CC)ccc2c1cccc2)OCCCCC(CC)CC)CC	THF	-27	10.1016/j.dyepig.2017.06.068
CCCCC1(CCCC)c2cc(ccc2c2c1cccc2)n1c2cc(sc2c2c1ccs2)/C=C(/C(=O)O)\C#N	THF	-3	10.1016/j.orgel.2015.07.019
CCCCC1(CCCC)c2cc(ccc2c2c1cccc2)n1c2cc(sc2c2c1cc(s2)c1ccc(cc1)N(c1ccccc1)c1ccccc1)/C=C(/C(=O)O)\C#N	THF	-10	10.1016/j.orgel.2015.07.019
CCCCC1(CCCC)c2cc(ccc2c2c1cc(cc2)N(c1ccccc1)c1ccccc1)c1sc2c(c1)n(c1c2sc(c1)/C=C(/C(=O)O)\C#N)c1ccc2c(c1)C(CCCC)(CCCC)c1c2cccc1	THF	-32	10.1016/j.orgel.2015.07.019
CCCCN1c2ccc(cc2Sc2c1cccc2)c1ccc(s1)c1cc2c(s1)c1c(n2c2ccc3c(c2)C(CCCC)(CCCC)c2c3cccc2)cc(s1)/C=C(/C(=O)O)\C#N	THF	-31	10.1016/j.orgel.2015.07.019
CCCCn1c2ccc(cc2c2c1cccc2)c1ccc(s1)c1cc2c(s1)c1c(n2c2ccc3c(c2)C(CCCC)(CCCC)c2c3cccc2)cc(s1)/C=C(/C(=O)O)\C#N	THF	-33	10.1016/j.orgel.2015.07.019
N#C/C(=C\c1ccc(s1)c1ccc2c(c1)C(CC)(CC)c1c2ccc(c1)c1nc(c(n1CC)c1ccccc1)c1ccccc1)/C(=O)O	THF	-46	10.1021/jo500330r
N#C/C(=C\c1ccc(s1)c1ccc(s1)c1ccc2c(c1)C(CC)(CC)c1c2ccc(c1)c1nc(c(n1CC)c1ccccc1)c1ccccc1)/C(=O)O	THF	-75	10.1021/jo500330r
N#C/C(=C\c1ccc(s1)c1ccc2c(c1)C(CC)(CC)c1c2c(cc(c1)c1nc(c(n1CC)c1ccccc1)c1ccccc1)c1nc(c(n1CC)c1ccccc1)c1ccccc1)/C(=O)O	THF	-42	10.1021/jo500330r

SMILES	SOLVENT	SHIFT (nm)	DOI
N#C/C(=C\c1ccc(s1)c1ccc(s1)c1ccc2c(c1)C(CC)(CC)c1c2c(cc(c1)c1nc(c(n1CC)c1ccccc1)c1ccccc1)c1nc(c(n1CC)c1ccccc1)c1ccccc1)/C(=O)O	THF	-52	10.1021/jo500330r
N#C/C(=C\c1ccc(s1)c1ccc2c(c1)C(CC)(CC)c1c2c(cc(c1)c1nc(c(n1CC)c1ccc2c(c1)C(CC)(CC)c1c2cccc1)c1ccc2c(c1)C(CC)(CC)c1c2cccc1)c1nc(c(n1CC)c1ccc2c(c1)C(CC)(CC)c1c2cccc1)c1ccc2c(c1)C(CC)(CC)c1c2cccc1)/C(=O)O	THF	-36	10.1021/jo500330r
N#C/C(=C\c1ccc(s1)c1ccc(s1)c1ccc2c(c1)C(CC)(CC)c1c2c(cc(c1)c1nc(c(n1CC)c1ccc2c(c1)C(CC)(CC)c1c2cccc1)c1ccc2c(c1)C(CC)(CC)c1c2cccc1)c1nc(c(n1CC)c1ccc2c(c1)C(CC)(CC)c1c2cccc1)c1ccc2c(c1)C(CC)(CC)c1c2cccc1)/C(=O)O	THF	-53	10.1021/jo500330r
CCCCN1c2ccc(cc2Sc2c1cccc2)c1nc2c(n1CCCC)c(ccc2c1ccc(cc1)/C=C(/C(=O)O)\C#N)c1ccc(cc1)/C=C(/C(=O)O)\C#N	THF	-42	10.1002/asia.201600766
CCCCn1c(nc2c1c(ccc2c1ccc(cc1)/C=C(/C(=O)O)\C#N)c1ccc(cc1)/C=C(/C(=O)O)\C#N)c1ccc(cc1)N(c1ccccc1)c1ccccc1	THF	-55	10.1002/asia.201600766
CCCCN1c2ccc(cc2Sc2c1cccc2)c1nc2c(n1CCCC)c(ccc2c1ccc(s1)/C=C(/C(=O)O)\C#N)c1ccc(s1)/C=C(/C(=O)O)\C#N	THF	-4	10.1002/asia.201600766
CCCCn1c(nc2c1c(ccc2c1ccc(s1)/C=C(/C(=O)O)\C#N)c1ccc(s1)/C=C(/C(=O)O)\C#N)c1ccc(cc1)N(c1ccccc1)c1ccccc1	THF	-44	10.1002/asia.201600766
CCCCN1c2ccc(cc2Sc2c1cccc2)c1nc2c(n1CCCC)c(ccc2c1ccc(s1)c1ccc(s1)/C=C(/C(=O)O)\C#N)c1ccc(s1)c1ccc(s1)/C=C(/C(=O)O)\C#N	THF	-71	10.1002/asia.201600766
CCCCn1c(nc2c1c(ccc2c1ccc(s1)c1ccc(s1)/C=C(/C(=O)O)\C#N)c1ccc(s1)c1ccc(s1)/C=C(/C(=O)O)\C#N)c1ccc(cc1)N(c1ccccc1)c1ccccc1	THF	-30	10.1002/asia.201600766
N#C/C(=C\c1ccc(cc1)c1ccc(cc1)c1[nH]c2c(n1)c(sc2c1ccc(cc1)C(C)(C)C)c1ccc(cc1)C(C)(C)C)/C(=O)O	THF	12	10.1016/j.dyepig.2016.02.009
N#C/C(=C\c1ccc(s1)c1ccc(s1)c1[nH]c2c(n1)c(sc2c1ccc(cc1)C(C)(C)C)c1ccc(cc1)C(C)(C)C)/C(=O)O	THF	-47	10.1016/j.dyepig.2016.02.009
CCCCn1c(nc2c1c(sc2c1cccs1)c1cccs1)c1ccc	THF	-16	10.1016/

SMILES	SOLVENT	SHIFT (nm)	DOI
(cc1)c1ccc(cc1)/C=C(/C(=O)O)\C#N			j.dyepig.2016.02.009
CCCCn1c(nc2c1c(sc2c1cccs1)c1cccs1)c1ccc(cc1)c1ccc(s1)/C=C(/C(=O)O)\C#N	THF	-61	10.1016/j.dyepig.2016.02.009
CCCCn1c(nc2c1c(sc2c1cccs1)c1cccs1)c1ccc(s1)c1ccc(s1)/C=C(/C(=O)O)\C#N	THF	-69	10.1016/j.dyepig.2016.02.009
CCCCCCOc1ccc(cc1)N(c1ccc(cc1)OCCCCCC)c1ccc(cc1)c1ccc(s1)c1nc2c(n1CCCCCC)c(ccc2c1ccc(s1)/C=C(/C(=O)O)\C#N)c1ccc(s1)/C=C(/C(=O)O)\C#N	THF	-26	10.1016/j.dyepig.2017.01.068
CCCCCCOc1ccc(cc1)N(c1ccc(cc1)OCCCCCC)c1ccc(cc1)c1nc2c(n1CCCCCC)c(ccc2c1cc(c(s1)/C=C(/C(=O)O)\C#N)CCCCCC)c1cc(c(s1)/C=C(/C(=O)O)\C#N)CCCCCC	THF	13	10.1016/j.dyepig.2017.01.068
CCCCCCOc1ccc(cc1)N(c1ccc(cc1)OCCCCCC)c1ccc(cc1)c1nc2c(n1CCCCCC)c(ccc2c1ccc(s1)c1sc(c(c1)CCCCCC)/C=C(/C(=O)O)\C#N)c1ccc(s1)c1sc(c(c1)CCCCCC)/C=C(/C(=O)O)\C#N	THF	40	10.1016/j.dyepig.2017.01.068
CCCC[C@@H](Cn1c2cc(c3ccc(s3)c3ccc(c4c3nc(c3ccccc3)c(n4)c3ccccc3)c3ccc(s3)/C=C(/C(=O)O)\C#N)c3c4c2c2c1cc(c1ccc(cc1)OC[C@H](CCCC)CC)c1c2c(c4ccc3)ccc1)CC	THF	-12	10.1016/j.dyepig.2016.12.013
CCCC[C@@H](Cn1c2cc(c3ccc(s3)c3ccc(c4c3nc(c3ccccc3)c(n4)c3ccccc3)c3ccc(s3)/C=c/3\sc(=C(C#N)C#N)[nH]c3=O)c3c4c2c2c1cc(c1ccc(cc1)OC[C@H](CCCC)CC)c1c2c(c4ccc3)ccc1)CC	THF	-17	10.1016/j.dyepig.2016.12.013
N#C/C(=C\c1ccc(s1)c1ccc(c2c1n[se]n2)c1ccc(cc1)N(c1ccccc1)c1ccccc1)/C(=O)O	THF	-14	10.1016/j.dyepig.2017.02.013
CCCCCCCCOc1ccc(cc1)N(c1ccc(cc1)OCCCCCCCC)c1ccc(cc1)c1ccc(c2c1n[se]n2)c1ccc(s1)/C=C(/C(=O)O)\C#N	THF	-30	10.1016/j.dyepig.2017.02.013
CCCCCCCCOc1ccc(cc1)N(c1ccc(cc1)OCCCCCCCC)c1ccc(cc1)c1ccc(c2c1n[se]n2)c1ccc(cc1)/C=C(/C(=O)O)\C#N	THF	-7	10.1016/j.dyepig.2017.02.013
CCCCCCn1c2ccc(cc2c2c1cccc2)C=C1C(=O)NC(=O)NC1=O	THF	-32	10.1080/15421406.2015.1106895
CCCCn1c2ccc(cc2c2c1cccc2)C=C1C(=O)NC(=O)NC1=O	THF	-12.5	10.1080/15421406.2015.1106895
CCCCn1c2ccc(cc2c2c1ccc(c2)C=C1C(=O)NC(=O)NC1=O)C=C1C(=O)NC(=O)NC1=O	THF	24	10.1080/15421406.2015.1106895

SMILES	SOLVENT	SHIFT (nm)	DOI
CCCCn1c2ccc(cc2c2c1ccc(c2)/C=C/1\SC(=O)NC1=O)/C=C/1\SC(=O)NC1=O	THF	7	10.1080/15421406.2015.1106895
O=C1N/C(=C\c2ccc(cc2)N(c2ccccc2)c2ccccc2)/C(=O)N1	THF	29	10.1039/C6NJ01314G
O=C1S/C(=C\c2ccc(cc2)N(c2ccccc2)c2ccccc2)/C(=O)N1	THF	38	10.1039/C6NJ01314G
O=C1NC(=O)/C(=C/c2ccc(cc2)N(c2ccc(cc2)/C=C/2\NC(=O)NC2=O)c2ccccc2)/N1	THF	47	10.1039/C6NJ01314G
O=C1NC(=O)/C(=C/c2ccc(cc2)N(c2ccc(cc2)/C=C/2\SC(=O)NC2=O)c2ccccc2)/S1	THF	38	10.1039/C6NJ01314G
CCCCCCCCN(c1ccccc1)c1ccc(cc1)C=C1C(=O)NC(=O)NC1=O	THF	-23	10.1016/j.synthmet.2015.06.018
CCCCCCCCN(c1ccccc1)c1ccc(cc1)C=C1C(=O)NC(=S)NC1=O	THF	-28	10.1016/j.synthmet.2015.06.018
CCCCCCCC[C@@]1(CC=C(C=C1)NC(=C1C(=O)NC(=O)NC1=O)c1ccccc1)C=C1C(=O)NC(=O)NC1=O	THF	-13	10.1016/j.synthmet.2015.06.018
CCCCCCCCN(c1ccc(cc1)C=C1C(=O)NC(=S)NC1=O)c1ccc(cc1)C=C1C(=O)NC(=S)NC1=O	THF	-4	10.1016/j.synthmet.2015.06.018
OC(=O)c1cc2C(=O)C(=Cc3ccc(cc3)N(c3ccccc3)c3ccccc3)C(=O)c2cc1C(=O)O	THF	4	10.1002/jccs.201500141
CCCCCCOc1ccc(cc1)N(c1ccc(cc1)OCCCCCC)c1ccc(cc1)C=C1C(=O)c2c(C1=O)cc(c(c2)C(=O)O)C(=O)O	THF	2	10.1002/jccs.201500141
CCCCCCn1c(nc(c1c1ccc(cc1)c1ccc(s1)/C=C(/C(=O)O)\C#N)c1ccc(cc1)c1ccc(s1)/C=C(/C(=O)O)\C#N)c1ccc2c(c1)c1ccccc1n2CCCCCC	THF	-34	10.1002/ejoc.201501131
CCCCCCOc1ccc(cc1)N(c1ccc(cc1)OCCCCCC)c1ccc(cc1)c1ccc(s1)c1nc(c(n1CCCCCC)c1ccc(cc1)c1ccc(s1)/C=C(/C(=O)O)\C#N)c1ccc(cc1)c1ccc(s1)/C=C(/C(=O)O)\C#N	THF	-44	10.1002/ejoc.201501131
CCCCCCOc1ccc(cc1)N(c1ccc(cc1)OCCCCCC)c1ccc(cc1)c1ccc(s1)c1nc(c(n1CCCCCC)c1ccc(s1)c1ccc(s1)/C=C(/C(=O)O)\C#N)c1ccc(s1)c1ccc(s1)/C=C(/C(=O)O)\C#N	THF	22	10.1002/ejoc.201501131
CCCCCCOc1ccc(cc1)N(c1ccc(cc1)OCCCCCC)c1ccc(cc1)c1ccc(s1)c1nc2c(n1CCCCCC)c1cc(sc1c1c2cc(s1)c1ccc(s1)/C=C(/C(=O)O)\C#N)c1ccc(s1)/C=C(/C(=O)O)\C#N	THF	-68	10.1002/ejoc.201501131
CCCCCCn1c2ccc(cc2c2c1cccc2)c1[nH]c(c(n1)c1ccc(cc1)c1ccc(s1)/C=C(/C(=O)O)\C#N)c1ccc(cc1)c1ccc(s1)/C=C(/C(=O)O)\	THF	11	10.1002/ejoc.201501131

SMILES	SOLVENT	SHIFT (nm)	DOI
C#N			
N#C/C(=C\c1cc(sc1c1sc2c(c1)C1(c3c2sc(c3)c2sc(cc2/C=C(/C(=O)O)\C#N)[Si](C(C)C)(C(C)C)C(C)C)c2ccc(cc2c2c1ccc(c2)c1ccc(cc1)C(C)(C)C)c1ccc(cc1)C(C)(C)C)[Si](C(C)C)(C(C)C)C(C)C)/C(=O)O	THF	-16	10.1021/ol303121z
CCCCCCC1(CCCCCC)c2cc(ccc2c2c1cc(cc2)c1ccc(s1)c1ccc(s1)/C=C(/C(=O)O)\C#N)c1ccc(s1)c1ccc(s1)/C=C(/C(=O)O)\C#N	THF	32	10.1016/j.tet.2010.11.044
CCCCCCC1(CCCCCC)c2cc(ccc2c2c1cc(cc2)c1ccc(s1)c1ccc(s1)c1ccc(s1)/C=C(/C(=O)O)\C#N)c1ccc(s1)c1ccc(s1)c1ccc(s1)/C=C(/C(=O)O)\C#N	THF	27	10.1016/j.tet.2010.11.044
CCCCCCCCC(n1c2cc(sc2c2c1cc(s2)c1ccc(s1)/C=C(/C(=O)O)\C#N)c1ccc(s1)/C=C(/C(=O)O)\C#N)CCCCCCCC	THF	49	10.1016/j.tet.2010.11.044
CCCCCCCCC(n1c2cc(sc2c2c1cc(s2)c1ccc(s1)c1ccc(s1)/C=C(/C(=O)O)\C#N)c1ccc(s1)c1ccc(s1)/C=C(/C(=O)O)\C#N)CCCCCCCC	THF	54	10.1016/j.tet.2010.11.044
N#C/C(=C\c1ccc(s1)c1ccc(cc1)N(CCO[Si](C(C)(C)C)(C)C)C)/C(=O)O	THF	22	10.1016/j.dyepig.2017.07.063
CN(c1ccc(cc1)/C=C\c1ccc(s1)/C=C(/C(=O)O)\C#N)CCO[Si](C(C)(C)C)(C)C	THF	28	10.1016/j.dyepig.2017.07.063
N#C/C(=C\c1ccc(c2c1nsn2)c1ccc(cc1)N(CCO[Si](C(C)(C)C)(C)C)C)/C(=O)O	THF	35	10.1016/j.dyepig.2017.07.063
N#C/C(=C\c1ccc(cc1)c1ccc(c2c1nsn2)c1ccc(cc1)N(CCO[Si](C(C)(C)C)(C)C)C)/C(=O)O	THF	140	10.1016/j.dyepig.2017.07.063
N#C/C(=C\c1ccc(s1)c1ccc(s1)c1ccc(cc1)N(c1ccc(cc1)C#Cc1cc2CCCc3ccc(CCCc1cc2)cc3)c1ccc(cc1)C#Cc1cc2CCCc3ccc(CCCc1cc2)cc3)/C(=O)O	THF	5	10.1016/j.dyepig.2016.09.043
N#C/C(=C\c1ccc(s1)c1ccc(s1)c1ccc(cc1)N(c1ccc(cc1)C#Cc1ccccc1)c1ccc(cc1)C#Cc1ccccc1)/C(=O)O	THF	6	10.1016/j.dyepig.2016.09.043
N#C/C(=C\c1ccc(s1)c1ccc(s1)c1ccc(cc1)N(c1ccc(cc1)/C=C/Cc1cc2CCCc3ccc(CCCc1cc2)cc3)c1ccc(cc1)/C=C/Cc1cc2CCCc3ccc(CCCc1cc2)cc3)/	THF	10	10.1016/j.dyepig.2016.09.043

SMILES	SOLVENT	SHIFT (nm)	DOI
C(=O)O			
N#C/C(=C\c1ccc(s1)c1ccc(s1)c1ccc(cc1)N(c1ccc(cc1)/C=C/c1ccccc1)c1ccc(cc1)/C=C/c1ccccc1)/C(=O)O	THF	9	10.1016/j.dyepig.2016.09.043
CCCCn1nc2c(n1)c(ccc2c1ccc(cc1)/C=C(/C(=O)O)\C#N)c1ccc(cc1)N(c1ccccc1)c1ccccc1	THF	-10	10.1016/j.dyepig.2018.05.044
CCCC[NH+]1[N-]C2C(N1)C(c1ccc(cc1)/C=C(/C(=O)O)\C#N)C1C(C2c2ccc(cc2)N(c2ccccc2)c2ccccc2)NN(N1)CCCC	THF	-2	10.1016/j.dyepig.2018.05.044
CCCCn1nc2c(n1)c(c1ccc(cc1)/C=C(/C(=O)O)\C#N)c1c(c2c2ccc(cc2)N(c2ccccc2)c2ccccc2)nc(c(n1)c1ccccc1)c1ccccc1	THF	0	10.1016/j.dyepig.2018.05.044
CCCCN1NC2C(N1)C(c1ccc(cc1)/C=C(/C(=O)O)\C#N)C1C(C2c2ccc(cc2)N(c2ccccc2)c2ccccc2)NSN1	THF	6	10.1016/j.dyepig.2018.05.044
CCCCCCOc1c2cc(sc2c2c(c1OCCCCCC)cc(s2)/C=C(/C(=O)O)\C#N)c1ccc(cc1)N(c1ccccc1)c1ccccc1	THF	10	10.1021/acsami.7b15181
CCCCCCOc1c2cc(sc2c2c(c1OCCCCCC)cc(s2)c1ccc(s1)/C=C(/C(=O)O)\C#N)c1ccc(cc1)N(c1ccccc1)c1ccccc1	THF	-1	10.1021/acsami.7b15181
CCCCCCOc1c2cc(sc2c2c(c1OCCCCCC)cc(s2)c1ccc(cc1)N(c1ccccc1)c1ccccc1)c1sc2c(c1)C(c1c2sc(c1)/C=C(/C(=O)O)\C#N)(CCCCCC)CCCCCC	THF	20	10.1021/acsami.7b15181
CCCCCCOc1c2cc(sc2c2c(c1OCCCCCC)cc(s2)c1ccc(cc1)N(c1ccccc1)c1ccccc1)c1cc2c(s1)c1sc(cc1c1c2nn(n1)[C@H](CCCCC)CC)/C=C(\C(=O)O)/C#N	THF	11	10.1021/acsami.7b15181
CCCCCCOc1ccc(cc1)N(c1ccc(cc1)OCCCCCC)c1ccc(cc1)c1ccc2c3c1cccc3c1n(c2=O)c2c(n1)cc(cc2)C(=O)O	THF	1	10.1016/j.dyepig.2015.05.028
OC(=O)c1ccc2c(c1)nc1n2c(=O)c2c3c1cccc3c(cc2)c1ccc(cc1)N(c1ccccc1)c1ccccc1	THF	-12	10.1016/j.dyepig.2015.05.028
CCn1c2ccc(cc2c2c1ccc(c2)c1ccncc1)c1ccncc1	THF	-23	10.1039/C3CC40498F
CCCCn1c2ccc(cc2c2c1ccc(c2)c1ccc(s1)c1ccncc1)c1ccc(s1)c1ccncc1	THF	-27	10.1039/C3CC40498F
CCCCC1(CCCC)c2cc(ccc2c2c1cccc2)n1c2ccs	THF	-47	10.1016/

SMILES	SOLVENT	SHIFT (nm)	DOI
c2c2c1cc(s2)c1ccc(s1)/C=C(/C(=O)O)\C#N			j.dyepig.2018.08.035
CCCCC1(CCCC)c2cc(ccc2c2c1cccc2)n1c2ccsc2c2c1cc(s2)c1ccc(s1)c1ccc(s1)/C=C(/C(=O)O)\C#N	THF	-49	10.1016/j.dyepig.2018.08.035
CCCCC1(CCCC)c2cc(ccc2c2c1cccc2)n1c2ccsc2c2c1cc(s2)c1sc2c(c1)n(c1c2sc(c1)/C=C(/C(=O)O)\C#N)c1ccc2c(c1)C(CCCC)(CCCC)c1c2cccc1	THF	-46	10.1016/j.dyepig.2018.08.035
CCCCC1(CCCC)c2cc(ccc2c2c1cccc2)n1c2ccsc2c2c1cc(s2)c1sc2c(c1)n(c1c2sc(c1)c1sc2c(c1)n(c1c2sc(c1)/C=C(/C(=O)O)\C#N)c1ccc2c(c1)C(CCCC)(CCCC)c1c2cccc1)c1ccc2c(c1)C(CCCC)(CCCC)c1c2cccc1	THF	-7	10.1016/j.dyepig.2018.08.035
C(#N)C(C(=O)O)=CC=1SC(=CC1)C=1C=NC(=CC1)C=1N(C(C=2C1C(N(C2C2=NC=C(C=C2)C2=CC=C(C=C2)N(C2=CC=CC=C2)C2=CC=CC=C2)CCCCCCCC)=O)=O)CCCCCCCC	THF	12	10.1039/C5RA27288B
C(#N)C(C(=O)O)=CC=1SC(=CC1)C=1C=NC(=CC1)C=1N(C(C=2C1C(N(C2C2=NC=C(C=C2)C2=CC=C(C=C2)N(C2=CC=CC=C2)C2=CC=CC=C2)CC(CCCC)CC)=O)=O)CC(CCCC)CC	THF	6	10.1039/C5RA27288B
COC1=CC=C(C=C1)N(C1=CC=C(C=C1)C=1C=CC(=NC1)C1=C2C(C(N1CCCCCCCC)=O)=C(N(C2=O)CCCCCCCC)C2=CC=C(C=N2)C2=CC=C(S2)C=C(C(=O)O)C#N)C2=CC=C(C=C2)OC	THF	17	10.1039/C5RA27288B
COC1=CC=C(C=C1)N(C1=CC=C(C=C1)C=1C=CC(=NC1)C1=C2C(C(N1CC(CCCC)CC)=O)=C(N(C2=O)CC(CCCC)CC)C2=CC=C(C=N2)C2=CC=C(S2)C=C(C(=O)O)C#N)C2=CC=C(C=C2)OC	THF	15	10.1039/C5RA27288B
CCCCCCCCn1c2cc3c(cc2c(=O)c2c1ccc(c2)c1ccc(cc1)N(c1ccccc1)c1ccccc1)n(CCCCCCCC)c1c(c3=O)cc(cc1)c1ccc(o1)C=C(C(=O)O)C#N	THF	118	10.1039/c2jm31929b
CCCCCCCCn1c2cc3c(=O)c4cc(ccc4n(c3cc2c(=O)c2c1ccc(c2)C=Cc1ccc(cc1)N(c1ccccc1)c1ccccc1)CCCCCCCC)c1ccc(o1)C=C(C(=O)O)C#N	THF	93	10.1039/c2jm31929b
CCCCCCCCn1c2cc3c(=O)c4cc(ccc4n(c3cc2c(=O)c2c1ccc(c2)c1ccc(cc1)N(c1ccc(cc1)OC)c1ccc(cc1)OC)CCCCCCCC)c1ccc(o1)C=C(C(=O)O)C#N	THF	107	10.1039/c2jm31929b
OC(=O)\C=C\C1=C(C2=CC=CC3=C2C(=O)\C(S3)=C2/SC3=CC=CC=C3C2=O)C2=CC=CC=C2C=C1	THF	-20	10.1016/j.dyepig.2015.07.016

SMILES	SOLVENT	SHIFT (nm)	DOI
OC(=O)C(=C\C1=C(C2=CC=CC3=C2C(=O)\C(S3)=C2/SC3=CC=CC=C3C2=O)C2=CC=CC=C2C=C1)\C#N	THF	-25	10.1016/j.dyepig.2015.07.016
OC(=O)\C=C\C1=C(C2=CC=CC3=C2C(=O)\C(N3)=C2\NC3=CC=CC=C3C2=O)C2=CC=CC=C2C=C1	THF	-19	10.1080/00397911.2013.831102
OC(=O)C(=C\C1=C(C2=CC=CC3=C2C(=O)\C(N3)=C2\NC3=CC=CC=C3C2=O)C2=CC=CC=C2C=C1)\C#N	THF	-23	10.1080/00397911.2013.831102
N#C/C(=C\c1ccc(s1)c1ccc(c2c1nsn2)c1ccc(s1)c1ccc(cc1)N(c1ccccc1)c1ccccc1)/C(=O)O	THF	15	10.1016/j.dyepig.2011.03.015
N#C/C(=C\c1ccc(s1)c1ccc(c2c1nsn2)c1ccc(s1)/C=C/c1ccc(cc1)N(c1ccccc1)c1ccccc1)/C(=O)O	THF	18	10.1016/j.dyepig.2011.03.015
N#C/C(=C\c1ccc(s1)c1ccc(c2c1nsn2)c1ccc(s1)C#Cc1ccc(cc1)N(c1ccccc1)c1ccccc1)/C(=O)O	THF	14	10.1016/j.dyepig.2011.03.015
CCCC[C@@H](COc1ccc(cc1)N(c1ccc(cc1)OC[C@H](CCCC)CC)c1ccc(cc1)c1ccc(s1)c1ccc(c2c1nsn2)c1ccc(s1)/C=C(/C(=O)O)\C#N)CC	THF	17	10.1016/j.dyepig.2011.03.015
OC1Cc2cc(ccc2N(C1)c1ccccc1)/C=C/1\SC(=S)N(C1=O)CC(=O)O	THF	1	10.1016/j.dyepig.2014.01.010
CCCOC1Cc2cc(ccc2N(C1)c1ccccc1)/C=C/1\SC(=S)N(C1=O)CC(=O)O	THF	22	10.1016/j.dyepig.2014.01.010
CCCOC1Cc2cc(/C=N/N(c3ccc(cc3)C=C3SC(=S)N(C3=O)CC(=O)O)c3ccccc3)ccc2N(C1)c1ccccc1	THF	-11	10.1016/j.dyepig.2014.01.010
CCCCCCCCCOC1Cc2cc(/C=N/N(c3ccc(cc3)C=C3SC(=S)N(C3=O)CC(=O)O)c3ccccc3)ccc2N(C1)c1ccccc1	THF	-12	10.1016/j.dyepig.2014.01.010
CCCOC1Cc2cc(/C=N/N(c3ccc(cc3)C=C(C(=O)O)C#N)c3ccccc3)ccc2N(C1)c1ccccc1	THF	13	10.1016/j.dyepig.2014.01.010
CCn1c2ccc(cc2c2c1c1c(c3c2n(CC)c2c3cccc2)n(c2c1cccc2)CC)c1ccc(s1)/C=C/1\SC(=S)N(C1=O)CC(=O)O	THF	17	10.1016/j.dyepig.2014.10.007
CCn1c2ccc(cc2c2c1c1c(c3c2n(CC)c2c3cccc2)n(c2c1cccc2)CC)c1ccc(cc1)/C=C\1/SC(=S)N(C1=O)CC(=O)O	THF	26	10.1016/j.dyepig.2014.10.007
CCCCCCOc1c(OCCCCCC)c(c2ccc(s2)/C=C(/	THF	11	10.1039/c2jm30427a

SMILES	SOLVENT	SHIFT (nm)	DOI
C(=O)O)\C#N)c2c(c1c1ccc(cc1)N(c1ccccc1)c1ccccc1)nsn2			
CCCCCCOc1c(OCCCCCC)c(c2ccc(s2)/C=C(/C(=O)O)\C#N)c2c(c1c1ccc3c(c1)C(CC)(CC)c1c3ccc(c1)N(c1ccccc1)c1ccccc1)nsn2	THF	13	10.1039/c2jm30427a
CCCCCCOc1c(OCCCCCC)c(c2ccc(s2)/C=C(/C(=O)O)\C#N)c2c(c1C#Cc1ccc(cc1)N(c1ccccc1)c1ccccc1)nsn2	THF	18	10.1039/c2jm30427a
CCCCCCOc1c(OCCCCCC)c(c2ccc(s2)/C=C(/C(=O)O)\C#N)c2c(c1c1ccc(s1)c1ccc(cc1)N(c1ccccc1)c1ccccc1)nsn2	THF	12	10.1039/c2jm30427a
CCCCCCOc1c(c2ccc(s2)c2ccc(cc2)N(c2ccc(cc2)OC)c2ccc(cc2)OC)c2nsnc2c(c1OCCCCCC)c1ccc(s1)/C=C(/C(=O)O)\C#N	THF	18	10.1039/c2jm30427a
N#C/C(=C\c1ccc(s1)c1ccc(c2c1nsn2)c1ccc(cc1)N(c1ccccc1)c1ccccc1)/C(=O)O	THF	11	10.1039/c2jm30427a
N#C/C(=C\c1ccc(s1)C1=Nc2c(C1(C)C)cc(cc2)N(c1ccccc1)c1ccccc1)/C(=O)O	THF	-12	10.1039/c2jm33228k
N#C/C(=C\c1ccc(o1)C1=Nc2c(C1(C)C)cc(cc2)N(c1ccccc1)c1ccccc1)/C(=O)O	THF	-21	10.1039/c2jm33228k
N#C/C(=C\c1cnc(s1)C1=Nc2c(C1(C)C)cc(cc2)N(c1ccccc1)c1ccccc1)/C(=O)O	THF	-19	10.1039/c2jm33228k
N#C/C(=C\c1ccc(cc1)C1=Nc2c(C1(C)C)cc(cc2)N(c1ccccc1)c1ccccc1)/C(=O)O	THF	-1	10.1039/c2jm33228k
CCCCCCc1cc(sc1c1sc(c(c1)CCCCCC)c1sc(c2c1OCCO2)/C=C(/C(=O)O)\C#N)c1sc2c(c1)c(c1ccc(s1)CC(CCCC)CC)c1c(c2c2ccc(s2)CC(CCCC)CC)ccs1	THF	11	10.1039/c5ta00332f
CCCCC(Cc1ccc(s1)c1c2sc(cc2c(c2c1cc(s2)c1cc(c(s1)c1cc(c(s1)c1sc(c2c1OCCO2)/C=C(/C(=O)O)\C#N)CCCCCC)CCCCCC)c1ccc(s1)CC(CCCC)CC)c1cc(c(s1)c1cc(c(s1)c1sc(c2c1OCCO2)/C=C(/C(=O)O)\C#N)CCCCCC)CCCCCC)CC	THF	-26	10.1039/c5ta00332f
CCCCCCOc1ccc(cc1)C1(c2ccc(cc2)OCCCCCC)c2cc3c4sc(cc4C(c3cc2c2c1cc(s2)/C=C(/	THF	20	10.1021/jo201730a

SMILES	SOLVENT	SHIFT (nm)	DOI
C(=O)O)\C#N)(c1ccc(cc1)OCCCCCC)c1ccc(cc1)OCCCCCC)N(c1ccccc1)c1ccccc1			
CCCCCCOc1ccc(cc1)C1(c2ccc(cc2)OCCCCCC)c2cc3c4sc(cc4C(c3cc2c2c1cc(s2)/C=C(/C(=O)O)\C#N)(c1ccc(cc1)OCCCCCC)c1ccc(cc1)OCCCCCC)N(c1cccc(c1)C)c1ccc(cc1)C	THF	20	10.1021/jo201730a
CCCCCCOc1ccc(cc1)C1(c2ccc(cc2)OCCCCCC)c2cc3c4sc(cc4C(c3cc2c2c1cc(s2)/C=C(/C(=O)O)\C#N)(c1ccc(cc1)OCCCCCC)c1ccc(cc1)OCCCCCC)N(c1cccc(c1)OC)c1ccc(cc1)OC	THF	20	10.1021/jo201730a
CCCCCCOc1cc(ccc1OCCCCCC)c1nc2c(nc1c1ccc(c(c1)OCCCCCC)OCCCCCC)c(sc2c1ccc(s1)c1ccc(cc1)N(c1ccccc1)c1ccccc1)c1ccc(s1)C=C(C(=O)O)C#N	THF	20	10.1039/C8TA04774J
CCCCCCOc1cc(ccc1OCCCCCC)c1nc2c(nc1c1ccc(c(c1)OCCCCCC)OCCCCCC)c(ccc2c1ccc(s1)c1ccc(cc1)N(c1ccccc1)c1ccccc1)c1ccc(s1)C=C(C(=O)O)C#N	THF	15	10.1039/C8TA04774J
CCCCCCOc1ccc(cc1)N(c1ccc(cc1)OCCCCCC)c1ccc(cc1)c1ccc(s1)c1sc(c2c1nc(c1ccc(c(c1)OCCCCCC)OCCCCCC)c(n2)c1ccc(c(c1)OCCCCCC)OCCCCCC)c1ccc(s1)C=C(C(=O)O)C#N	THF	13	10.1039/C8TA04774J
CCCCCCOc1ccc(cc1)N(c1ccc(cc1)OCCCCCC)c1ccc(cc1)c1ccc(s1)c1ccc(c2c1nc(c1ccc(c(c1)OCCCCCC)OCCCCCC)c(n2)c1ccc(c(c1)OCCCCCC)OCCCCCC)c1ccc(s1)C=C(C(=O)O)C#N	THF	8	10.1039/C8TA04774J
CCCCCCN1c2ccc(cc2Sc2c1ccc(c2)OC[C@H](CCCC)CC)c1ccc(s1)[C@@H]1[C@H]([O-])/C(=C/[C@@H]2[NH+](CCCCCCCC)c3c(C2(C)C)cc(cc3)C(=O)O)/C1=O	THF	13	10.1016/j.dyepig.2014.07.031
CCCCCCN1c2ccc(cc2Sc2c1ccc(c2)OC[C@H](CCCC)CC)c1ccc(s1)c1ccc(s1)[C@@H]1[C@H]([O-])/C(=C/[C@@H]2[NH+](CCCCCCCC)c3c(C2(C)C)cc(cc3)C(=O)O)/C1=O	THF	17	10.1016/j.dyepig.2014.07.031
CCCCCCN1c2ccc(cc2Sc2c1ccc(c2)OC[C@H](CCCC)CC)c1sc2c(c1)sc(c2)[C@@H]1[C@H]([O-])/C(=C/[C@@H]2[NH+](CCCCCCCC)c3c(C2(C)C)cc(cc3)C(=O)O)/C1=O	THF	12	10.1016/j.dyepig.2014.07.031

SMILES	SOLVENT	SHIFT (nm)	DOI
CCCCCCN1c2ccc(cc2Sc2c1ccc(c2)OC[C@H](CCCC)CC)c1ccc(s1)[C@@H]1[C@H]([O-])/C(=C/[C@@H]2[NH+](CCCCCCCC)c3c(C2(C)C)cc(cc3)c2ccc(s2)/C=C(/C(=O)O)\C#N)/C1=O	THF	16	10.1016/j.dyepig.2014.07.031
CCCCCCOc1c2cc(ccc2c(c2c1ccc(c2)/C=C(/C(=O)O)\C#N)OCCCCCC)N(c1ccc2c(c1)Sc1c(N2CCCCCC)cccc1)c1ccccc1	THF	-6	10.1002/cssc.201403016
CCCCCCOc1c2cc(ccc2c(c2c1ccc(c2)c1ccc(s1)/C=C(/C(=O)O)\C#N)OCCCCCC)N(c1ccc2c(c1)Sc1c(N2CCCCCC)cccc1)c1ccccc1	THF	-16	10.1002/cssc.201403016
CCCCCCOc1c2cc(ccc2c(c2c1cccc2)OCCCCCC)N(c1ccc2c(c1)Sc1c(N2CCCCCC)ccc(c1)/C=C(/C(=O)O)\C#N)c1ccccc1	THF	-20	10.1002/cssc.201403016
CCCCCCN1c2ccc(cc2Sc2c1ccc(c2)c1ccc(s1)/C=C(\C(=O)O)/C#N)N(c1ccc2c(c1)c(OCCCCCC)c1c(c2OCCCCCC)cccc1)c1ccccc1	THF	-42	10.1002/cssc.201403016
CCCCCCOc1c2cc(ccc2c(c2c1cccc2)OCCCCCC)N(c1ccc2c(c1)Sc1c(N2CCCCCC)ccc(c1)/C=C(\C(=O)O)/C#N)c1ccccc1	THF	-53	10.1002/cssc.201403016
N#CC(=Cc1ccc(cc1)N(c1ccccc1)c1ccccc1)C(=O)O	THF	15	10.1016/j.tet.2012.02.077
N#C/C(=C\c1ccc(cc1)N(c1ccc(cc1)C=Cc1ccc(cc1)N(c1ccccc1)c1ccccc1)c1ccc(cc1)C=Cc1ccc(cc1)N(c1ccccc1)c1ccccc1)/C(=O)O	THF	2	10.1016/j.tet.2012.02.077
CCCCCCCCN1c2ccc(cc2Sc2c1cccc2)/C=C/c1ccc(cc1)N(c1ccc(cc1)/C=C(/C(=O)O)\C#N)c1ccc(cc1)/C=C/c1ccc2c(c1)Sc1c(N2CCCCCCCC)cccc1	THF	-46	10.1016/j.tet.2012.02.077
CCCCCCCCn1c2ccccc2c2c1cc(/C=C/c1ccc(cc1)N(c1ccc(cc1)/C=C(/C(=O)O)\C#N)c1ccc(cc1)/C=C/c1ccc3c(c1)n(CCCCCCCC)c1c3cccc1)cc2	THF	-35	10.1016/j.tet.2012.02.077
CCCCCCOc1ccc(cc1)c1ccc2c(c1)Sc1c(N2c2ccc(cc2)OCCOCCOCCOC)ccc(c1)/C=C(/C(=O)O)\C#N	THF	-6	10.1002/cssc.201500589
CCCCCCOc1ccc(cc1)N1c2ccc(cc2Sc2c1ccc(c2)/C=C(/C(=O)O)\C#N)c1ccc(cc1)CCOCOCOCCOC	THF	-8	10.1002/cssc.201500589
COCCOCOCOCCc1ccc(cc1)c1ccc2c(c1)Sc1c(	THF	-1	10.1002/cssc.201500589

SMILES	SOLVENT	SHIFT (nm)	DOI
N2c2ccc(cc2)OOCCOCCOC)ccc(c1)/C=C(/C(=O)O)\C#N			
COCCOCCOCCOc1cc(OCCOCCOCCOC)ccc1c1ccc2c(c1)Sc1c(N2c2ccc(cc2)OOCCOCCOC)ccc(c1)/C=C(/C(=O)O)\C#N	THF	-8	10.1002/cssc.201500589
CCCCCCOc1ccc(cc1)c1ccc2c(c1)Sc1c(N2c2ccc(cc2)OCCCCCC)ccc(c1)/C=C(/C(=O)O)\C#N	THF	-10	10.1002/cssc.201500589
CCCCCCn1c2ccc(cc2c2c1c(c1ccccc1)c1c(c2c2ccccc2)n(c2c1cccc2)CCCCCC)c1ccc(s1)c1ccc(s1)/C=C(\C(=O)O)/C#N	THF	-14	10.1039/C3TA11748K
CCCCCCn1c2ccc(cc2c2c1c(c1ccccc1)c1c(c2c2ccccc2)n(c2c1cccc2)CCCCCC)c1ccc(c2c1nsn2)c1ccc(s1)/C=C(\C(=O)O)/C#N	THF	5	10.1039/C3TA11748K
CCCCCCn1c2ccc(cc2c2c1c(c1ccccc1)c1c(c2c2ccccc2)n(c2c1cccc2)CCCCCC)c1ccc(s1)c1cc2c(s1)C=C(C2)/C=C(\C(=O)O)/C#N	THF	6	10.1039/C3TA11748K
CCCCCCn1c2ccc(cc2c2c1c(c1ccccc1)c1c(c2c2ccccc2)n(c2c1cccc2)CCCCCC)c1ccc(c2c1nsn2)c1sc2c(c1)sc(c2)/C=C(/C(=O)O)\C#N	THF	8	10.1039/C3TA11748K
CCCCCCn1c2ccc(cc2c2c1cccc2)c1ccc(s1)c1ccc(s1)/C=C(\C(=O)O)/C#N	THF	35	10.1039/C3TA11748K
CCCCCCc1cc(sc1c1ccc2c(c1)C(=[N+]1C2=Cc2n([B-]1(F)F)c(c1c2ccc(c1)c1sc(cc1CCCCCC)/C=C(\C(=O)O)/C#N)c1ccccc1OC)c1ccccc1OC)C=C(C(=O)O)C#N	THF	-3	10.1039/C3TA15340A
CCCCCCc1cc(sc1c1ccc2c(c1)C1=Cc3c4cc(ccc4c(n3[B-]([N+]1=C2c1ccccc1OC)(F)F)c1ccccc1OC)c1sc(cc1CCCCCC)/C=C(\C(=O)O)/C#N)/C=C(\C(=O)O)/C#N	THF	-40	10.1039/C3TA15340A
CCCCCCc1cc(sc1c1ccc2c(c1)C(=[N+]1C2=Cc2n([B-]1(F)F)c(c1c2ccc(c1)c1sc(cc1CCCCCC)/C=C(\C(=O)O)/C#N)c1ccccc1OC)c1ccccc1OC)CO	THF	-66	10.1039/C3TA15340A
CCCCCCCCCCOc1cc2c(cc1OCCCCCCCCCC)c1sc(cc1c1c2sc(c1)c1ccc2c(c1)C(CCCC)(CCCC)c1c2ccc(c1)N(c1ccccc1)c1ccccc1)C=C(C(=O)O)C#N	THF	-27	10.1039/C3TA12901B
CCCCCCCCCCOc1cc2c(cc1OCCCCCCCCCC)c1sc(cc1c1c2sc(c1)c1ccc2c(c1)C(CCCC)(CCCC)c1c2ccc(c1)N(c1ccccc1)c1ccccc1)c1cc2c(s1)c1ccccc1c1c2cc(s1)C=C(C(=O)O)C#N	THF	-61	10.1039/C3TA12901B
CCCCC(COc1cc2c3sc(cc3c3c(c2cc1OCC(CCC	THF	-59	10.1039/C3TA12901B

SMILES	SOLVENT	SHIFT (nm)	DOI
C)CC)sc(c3)C=C(C(=O)O)C#N)c1ccc2c(c1)c1ccccc1n2CCCC)CC			
CCCCCCCCCCOc1cc2c3sc(cc3c3c(c2cc1OCCCCCCCCCC)sc(c3)C=C(C(=O)O)C#N)c1ccc2c(c1)c1ccccc1n2CCCCCCCCCC	THF	-57	10.1039/C3TA12901B
Oc1cc(ccc1O)c1ccc2c(c1)C(C)(C)c1c2ccc(c1)N(c1ccccc1)c1ccccc1	THF	-38	10.1039/C4TA01286K
CCCCn1c2cc(ccc2c2c1cc(cc2)c1ccc(c(c1)O)O)N(c1ccccc1)c1ccccc1	THF	-36	10.1039/C4TA01286K
CCCCn1c2cc(ccc2c2c1cc(cc2)N(c1ccccc1)c1ccccc1)c1ccc(s1)c1ccc(s1)c1ccc(s1)c1ccc(c(c1)O)O	THF	-71	10.1039/C4TA01286K
N#C/C(=C\c1c2c(Sc3c(N2C)cccc3)cc2c1cccc2)/C(=O)O	THF	27	10.1039/C4TA02720E
N#C/C(=C\c1ccc(s1)/C=C/c1c2c(Sc3c(N2C)cccc3)cc2c1cccc2)/C(=O)O	THF	30	10.1039/C4TA02720E
N#C/C(=C\c1ccc(s1)c1ccc(s1)/C=C/c1c2c(Sc3c(N2C)cccc3)cc2c1cccc2)/C(=O)O	THF	18	10.1039/C4TA02720E
CCCCCCN1c2ccc(cc2Sc2c1ccc(c2)C=C(C(=O)O)C#N)c1ccc2[n+](c1)[B-](F)(F)n1c(=C2C#N)cccc1	THF	0	10.1039/C5TA03807C
CCCCCCN1c2ccc(cc2Sc2c1ccc(c2)c1ccc(s1)C=C(C(=O)O)C#N)c1ccc2[n+](c1)[B-](F)(F)n1c(=C2C#N)cccc1	THF	1	10.1039/C5TA03807C
CCCCCCN1c2ccc(cc2Sc2c1ccc(c2)/C=C(\C(=O)O)/C#N)c1ccc(s1)c1ccc2[n+](c1)[B-](F)(F)n1c(=C2C#N)cccc1	THF	3	10.1039/C5TA03807C
CCCCCCc1cc(sc1c1ccc2[n+](c1)[B-](F)(F)n1c(=C2C#N)cccc1)c1ccc2c(c1)Sc1c(N2CCCCCC)ccc(c1)/C=C(\C(=O)O)/C#N	THF	-1	10.1039/C5TA03807C
CCCCCCc1cc(sc1c1ccc2c(c1)Sc1c(N2CCCCCC)ccc(c1)/C=C(\C(=O)O)/C#N)c1ccc2[n+](c1)[B-](F)(F)n1c(=C2C#N)cccc1	THF	3	10.1039/C5TA03807C
CCCCCCc1cc(sc1c1ccc2c(c1)Sc1c(N2c2ccc(cc2)c2ccc3[n+](c2)[B-](F)(F)n2c(=C3C#N)cccc2)ccc(c1)/C=C(\C(=O)O)/C#N)c1ccc2[n+](c1)[B-](F)(F)n1c(=C2C#N)cccc1	THF	-3	10.1039/C5TA03807C
CCCCCCc1cc(sc1c1ccc2[n+](c1)[B-](F)(F)n1c(=C2C#N)cccc1)c1ccc2c(c1)Sc1c(N2c2ccc(cc2)c2ccc3[n+](c2)[B-](F)(F)n2c(=C3C#N)cccc2)ccc(c1)/C=C(\C(=O)O)/C#N	THF	1	10.1039/C5TA03807C
N#C/C(=C/	THF	0	10.1039/C5TA03807C

SMILES	SOLVENT	SHIFT (nm)	DOI
c1ccc2c(c1)Sc1c(N2c2ccc(cc2)c2ccc3[n+](c2)[B-](F)(F)n2c(=C3C#N)cccc2)cccc1)/C(=O)O			
CCCCCCN1c2ccc(cc2Sc2c1ccc(c2)/C=C(\C(=O)O)/C#N)c1ccc(cc1)N(c1ccccc1)c1ccccc1	THF	17	10.1039/C5TA03807C
CCCCCCN1c2ccc(cc2Sc2c1cccc2)/C=C(\C(=O)O)/C#N	THF	44	10.1039/C5TA03807C
N#C/C(=C\c1ccc(cc1)c1ccc2c(c1)c1cccc3c1n2c1ccccc1C3(CC)CC)/C(=O)O	THF	-18	10.1039/C3TA01657A
N#C/C(=C\c1ccc(s1)c1ccc2c(c1)c1cccc3c1n2c1ccccc1C3(CC)CC)/C(=O)O	THF	22	10.1039/C3TA01657A
N#CC(=Cc1ccc(s1)c1ccc(s1)c1ccc2c(c1)c1cccc3c1n2c1ccccc1C3(CC)CC)C(=O)O	THF	16	10.1039/C3TA01657A
N#CC(=Cc1ccc(s1)c1ccc(s1)c1ccc(s1)c1ccc2c(c1)c1cccc3c1n2c1ccccc1C3(CC)CC)C(=O)O	THF	27	10.1039/C3TA01657A
N#CC(=Cc1ccc(s1)c1ccc(c2c1nsn2)c1ccc2c(c1)c1cccc3c1n2c1ccccc1C3(CC)CC)C(=O)O	THF	6	10.1039/C3TA01657A
N#CC(=Cc1ccc(s1)c1ccc(cc1)N(c1ccccc1)c1ccccc1)C(=O)O	THF	21	10.1039/C3TA01657A
CCCCCC1(CCCCC)COc2c(OC1)c(sc2c1sc2c(n1)sc(n2)c1sc(c2c1OCC(CO2)(CCCCC)CCCCC)c1ccc(s1)C=C(C(=O)O)C#N)c1ccc(cc1)N(c1ccccc1)c1ccccc1	THF	103	10.1039/C5RA03530A
CCCCCCOc1ccc(cc1)N(c1ccc(cc1)OCCCCCC)c1ccc(cc1)c1sc(c2c1OCC(CO2)(CCCCC)CCCCC)c1sc2c(n1)sc(n2)c1sc(c2c1OCC(CO2)(CCCCC)CCCCC)c1ccc(s1)C=C(C(=O)O)C#N	THF	110	10.1039/C5RA03530A
CCCCCCSc1ccc(cc1)N(c1ccc(cc1)SCCCCCC)c1ccc(cc1)c1sc(c2c1OCC(CO2)(CCCCC)CCCCC)c1sc2c(n1)sc(n2)c1sc(c2c1OCC(CO2)(CCCCC)CCCCC)c1ccc(s1)C=C(C(=O)O)C#N	THF	86	10.1039/C5RA03530A
CCCCCC1(CCCCC)COc2c(OC1)c(sc2c1sc2c(n1)sc(n2)c1sc(c2c1OCC(CO2)(CCCCC)CCCCC)c1sc(c2c1OCCO2)C=C(C(=O)O)C#N)c1ccc(cc1)N(c1ccccc1)c1ccccc1	THF	138	10.1039/C5RA03530A
CCCCCC1(CCCCC)COc2c(OC1)c(sc2c1nc2c(s1)nc(s2)c1sc(c2c1OCC(CO2)(CCCCC)CCCCC)c1ccc(s1)C=C(C(=O)O)C#N)c	THF	104	10.1039/C5RA03530A

SMILES	SOLVENT	SHIFT (nm)	DOI
1ccc2c(c1)Sc1c(N2c2ccc(cc2)OC)cccc1			
CCCCCCCCOc1ccc(cc1)c1n(CCCCCCCC)c2c(c1c1c(c3ccc(cc3)OCCCCCCCC)n(c3c1cc(cc3)c1ccc(o1)C=C(C(=O)O)C#N)CCCCCCCC)ccc(c2)c1ccc(o1)C=C(C(=O)O)C#N	THF	28	10.1039/C4RA12888E
CCCCCCCCOc1ccc(cc1)c1n(CCCCCCCC)c2c(c1c1c(c3ccc(cc3)OCCCCCCCC)n(c3c1cc(cc3)c1ccc(s1)C=C(C(=O)O)C#N)CCCCCCCC)ccc(c2)c1ccc(s1)C=C(C(=O)O)C#N	THF	29	10.1039/C4RA12888E
CCCCCCCCOc1ccc(cc1)c1n(CCCCCCCC)c2c(c1c1c(c3ccc(cc3)OCCCCCCCC)n(c3c1cc(cc3)c1ccc(s1)c1ccc(s1)C=C(C(=O)O)C#N)CCCCCCCC)ccc(c2)c1ccc(s1)c1ccc(s1)C=C(C(=O)O)C#N	THF	19	10.1039/C4RA12888E
N#CC(=Cc1ccc(s1)c1ccc(s1)C1=NN(C(C1)c1ccc(cc1)N(C)C)c1ccc(cc1)c1sc2c(n1)ccc(c2)C)C(=O)O	THF	45	10.1039/C6RA00738D
CCCCCCOc1ccc(cc1)C1CC(=NN1c1ccc(cc1)c1sc2c(n1)ccc(c2)C)c1ccc(s1)c1ccc(s1)C=C(C(=O)O)C#N	THF	40	10.1039/C6RA00738D
CCOC(=O)c1ccc(cc1)C1CC(=NN1c1ccc(cc1)c1sc2c(n1)ccc(c2)C)c1ccc(s1)c1ccc(s1)C=C(C(=O)O)C#N	THF	40	10.1039/C6RA00738D
N#CC(=Cc1ccc(s1)c1ccc(s1)C1=NN(C(C1)c1ccccc1)c1ccc(cc1)c1sc2c(n1)ccc(c2)C)C(=O)O	THF	44	10.1039/C6RA00738D
CN(C)C1=CC=C(C=C1)C1=CC=C(CC1)C(O)=O	THF	35	10.1002/chem.201001294
OC(=O)C1=CC=C(CC1)C1=CC=C(C=C1)N(C1=CC=CC=C1)C1=CC=CC=C1	THF	34	10.1002/chem.201001294
OC(=O)C1=CC=C(CC1)C1=CC2=C(C=C1)N(CC2)C1=CC=CC=C1	THF	25	10.1002/chem.201001294
CN(C)C#CC1=CC=C(C=C1)C1=C2C(=O)N(CC3=CC=CC=C3)C(=C2C(=O)N1CC1=CC=CC=C1)C1=CC=C(C=C1)C(O)=O	THF	-7	10.1002/cplu.201200059
CCCCC(CC)CN1C(=O)C2=C(N(CC(CC)CCCC)C(=O)C2=C1C1=CC=C(S1)C#CCN(C)C)C1=CC=C(S1)C#CC1=CC=C(C=C1)C(O)=O	THF	32	10.1002/cplu.201200059
CCCCCCC1=C(SC(=C1)C1=CC=C(C2=CC(CCCCCC)=C(S2)\C=C(/C#N)C(O)=O)C2=NSN=C12)\C=C\C1=CC=C(C=C1)N(C1=CC=CC=C1)C1=CC=CC=C1	THF	-30	10.1002/asia.201000158
CCCCCCC1=C(SC(=C1)C1=CC=C(C2=CC(CCCCCC)=C(S2)\C=C(/	THF	-34	10.1002/asia.201000158

SMILES	SOLVENT	SHIFT (nm)	DOI
C#N)C(O)=O)C2=NSN=C12)\C=C\ C1=CC=C(C=C1)N(C1=CC=C(OC)C=C1)C1=C C=C(OC)C=C1			
OC(=O)C(=C/C1=CC=C(\C=C\ C2=CC=C(C=C2)N(C2=CC=CC=C2)C2=CC=CC =C2)N1C1=CC=C(C=C1)C1=CC=C(C=C1)N(C 1=CC=CC=C1)C1=CC=CC=C1)\C#N	THF	0	10.1002/chem.200901150
CCCCCCN1C2=CC=CC=C2C2=CC(=CC=C12)C 1=CC=C(C=C1)N1C(\C=C\ C2=CC=C(C=C2)N(C2=CC=CC=C2)C2=CC=CC =C2)=CC=C1\C=C(\C#N)C(O)=O	THF	-7	10.1002/chem.200901150
CCCCCc1c(sc2c1sc1c2c2nccnc2c2c1sc1c2sc (c1CCCCC)/C=C(/C(=O)O)\ C#N)c1ccc2c(c1)c1ccccc1n2CC(CCCC)CC	THF	-73	10.1016/ j.dyepig.2018.06.010
CCCCCc1c(sc2c1sc1c2c2nccnc2c2c1sc1c2sc (c1CCCCC)/C=C(/C(=O)O)\ C#N)c1ccc(c2c1nsn2)c1ccc2c(c1)c1ccccc1n 2CC(CCCC)CC	THF	-63	10.1016/ j.dyepig.2018.06.010
CCCCCc1c(sc2c1sc1c2c2nc3ccccc3nc2c2c1s c1c2sc(c1CCCCC)/C=C(/C(=O)O)\ C#N)c1ccc2c(c1)c1ccccc1n2CC(CCCC)CC	THF	-84	10.1016/ j.dyepig.2018.06.010
CCCCCc1c(sc2c1sc1c2c2nc3ccccc3nc2c2c1s c1c2sc(c1CCCCC)/C=C(/C(=O)O)\ C#N)c1ccc(c2c1nsn2)c1ccc2c(c1)c1ccccc1n 2CC(CCCC)CC	THF	-73	10.1016/ j.dyepig.2018.06.010
CCCCC(Cn1c2ccccc2c2c1ccc(c2)c1sc2c(c1C(CCCC)CC)sc1c2c2nccnc2c2c1sc1c2sc(c1C(C CCC)CC)/C=C(/C(=O)O)\C#N)CC	THF	-50	10.1016/ j.dyepig.2018.06.010
N#CC(=Cc1ccc(s1)c1ccc(cc1)N(CCO[Si](C(C) (C)C)(C)C)C)C(=O)O	THF	22	10.1016/ j.dyepig.2017.07.063
CN(c1ccc(cc1)/C=C/ c1ccc(s1)C=C(C(=O)O)C#N)CCO[Si](C(C) (C)C)(C)C	THF	28	10.1016/ j.dyepig.2017.07.063
N#CC(=Cc1ccc(c2c1nsn2)c1ccc(cc1)N(CCO[Si](C(C)(C)C)(C)C)C)C(=O)O	THF	35	10.1016/ j.dyepig.2017.07.063
N#CC(=Cc1ccc(cc1)c1ccc(c2c1nsn2)c1ccc(c c1)N(CCO[Si](C(C)(C)C)(C)C)C)C(=O)O	THF	140	10.1016/ j.dyepig.2017.07.063
CC(CCOc1ccc(cc1)N1c2ccc(cc2Sc2c1cccc2)/ C=C/c1nc2cc(ccc2nc1/C=C/ c1ccc2c(c1)Sc1c(N2c2ccc(cc2)OCCC(C)C)ccc c1)C(=O)O)C	THF	6	10.1016/ j.tetlet.2018.07.049
N#CC(=Cc1ccc(cc1)c1c2cc3ccccc3cc2c(c2c1 cc1ccccc1c2)C#C[Si](C(C)C) (C(C)C)C(C)C)C(=O)O	THF	-18	10.1039/C8NR01502C

SMILES	SOLVENT	SHIFT (nm)	DOI
N#CC(=Cc1ccc(cc1)C#Cc1c2cc3ccccc3cc2c(c2c1cc1ccccc1c2)C#C[Si](C(C)C)(C(C)C)C(C)C)C(=O)O	THF	-30	10.1039/C8NR01502C
N#CC(=Cc1ccc(s1)c1c2cc3ccccc3cc2c(c2c1cc1ccccc1c2)C#C[Si](C(C)C)(C(C)C)C(C)C)C(=O)O	THF	-20	10.1039/C8NR01502C
N#CC(=Cc1ccc(s1)C#Cc1c2cc3ccccc3cc2c(c2c1cc1ccccc1c2)C#C[Si](C(C)C)(C(C)C)C(C)C)C(=O)O	THF	-38	10.1039/C8NR01502C
C(#N)/C(/C(=O)O)=C\C=1SC(=CC1)C1=CC2=C(C=3N(C4=C(C3S2)SC2=C4C=CC(=C2)C2=CC=C(C=C2)N(C2=CC=CC=C2)C2=CC=CC=C2)CC(CCCC)CC)C=C1	THF	31	10.1016/j.dyepig.2018.10.004
C(#N)/C(/C(=O)O)=C\C=1SC(=CC1)C1=CC2=C(C=3N(C4=C(C3S2)SC2=C4C=CC(=C2)C2=CC=4C(C3=CC=CC=C3C4C=C2)(C)C)CC(CCCC)CC)C=C1	THF	26	10.1016/j.dyepig.2018.10.004
C(#N)/C(/C(=O)O)=C\C=1SC(=CC1)C1=CC2=C(C=3N(C4=C(C3S2)SC2=C4C=CC(=C2)C2=CC=4N(C3=CC=CC=C3C4C=C2)CC)CC(CCCC)CC)C=C1	THF	33	10.1016/j.dyepig.2018.10.004
C(#N)/C(/C(=O)O)=C\C=1SC(=CC1)C1=CC2=C(C=3N(C4=C(C3S2)SC2=C4C=CC=C2)CC(CCCCCCCC)CCCCCC)C=C1	THF	39	10.1016/j.dyepig.2019.02.003
C(#N)/C(/C(=O)O)=C\C=1SC(=C(C1)CCCCCC)C1=CC2=C(C=3N(C4=C(C3S2)SC2=C4C=CC=C2)CC(CCCCCCCC)CCCCCC)C=C1	THF	27	10.1016/j.dyepig.2019.02.003
C(CCCCC)C(CN1C2=C(C3=C1C1=C(S3)C=C(C=C1)C1=CC=C(S1)/C=C(/C(=O)O)\C#N)SC1=C2C=CC(=C1)C1=CC=C(S1)/C=C(/C(=O)O)\C#N)CCCCCCCC	THF	8	10.1016/j.dyepig.2019.02.003
C(CCCCC)C(CN1C2=C(C3=C1C1=C(S3)C=C(C=C1)C1=C(C=C(S1)/C=C(/C(=O)O)\C#N)CCCCCC)SC1=C2C=CC(=C1)C1=C(C=C(S1)/C=C(/C(=O)O)\C#N)CCCCCC)CCCCCCCC	THF	3	10.1016/j.dyepig.2019.02.003
N#C/C(=C/c1ccc(s1)c1ccc(cc1)N(c1ccccc1)c1ccccc1)/C(=O)O	THF	-77	10.1016/j.dyepig.2019.02.002
C(#N)/C(/C(=O)O)=C\C=1SC(=CC1)C1=CC=2C(C3=CC=CC=C3N(C2C=C1)C1=CC=CC=C1)(C)C	THF	-69	10.1016/j.dyepig.2019.02.002
C(#N)C(C(=O)O)=CC=1SC(=CC1)C1=CC=2C(C	THF	-59	10.1016/

SMILES	SOLVENT	SHIFT (nm)	DOI
3=CC=CC=C3N(C2C=C1)C1=CC=CC=C1)(C1=CC=CC=C1)C1=CC=CC=C1			j.dyepig.2019.02.002
C(#N)C(C(=O)O)=CC1=CC=C(S1)C=1SC(=CC1)C1=CC=2C(C3=CC=CC=C3N(C2C=C1)C1=CC=CC=C1)(C)C	THF	-95	10.1016/j.dyepig.2019.02.002
C(C)(C)(C)C1=CC=C2N(C=3C=CC(=CC3C(C2=C1)(C)C)C1=CC=C(S1)C=1SC(=CC1)/C=C(/C(=O)O)\C#N)C1=CC=C(C=C1)C(C)(C)C	THF	-85	10.1016/j.dyepig.2019.02.002
CCCCCCCCN1C2=CC=C(C=C2SC2=CC(=CC=C12)C1=CC=CC=C1)C1=CC=C(S1)\C=C(/C#N)C(O)=O	THF	9	10.1039/C9TA00472F
CCCCCCCCN1C2=CC=C(C=C2SC2=CC(=CC=C12)C1=CC=C(OC)C=C1)C1=CC=C(S1)\C=C(/C#N)C(O)=O	THF	-2	10.1039/C9TA00472F
C(#N)/C(/C(=O)O)=C\C=1SC(=CC1)C=1C=CC=2N(C3=CC=C(C=C3SC2C1)C1=C(C=CC=C1)OC)CCCCCC	THF	15	10.1039/C9TA00472F
C(#N)/C(/C(=O)O)=C\C=1SC(=CC1)C=1C=CC=2N(C3=CC=C(C=C3SC2C1)C1=CC=C(C=C1)SC)CCCCCC	THF	-1	10.1039/C9TA00472F
C(#N)/C(/C(=O)O)=C\C=1SC(=CC1)C=1C=CC=2N(C3=CC=C(C=C3SC2C1)C1=C(C=C(C=C1)OC)OC)CCCCCC	THF	13	10.1039/C9TA00472F
C(#N)/C(/C(=O)O)=C\C=1SC(=CC1)C=1C=CC=2N(C3=CC=C(C=C3SC2C1)C1=C(C(=CC=C1)OC)OC)CCCCCC	THF	12	10.1039/C9TA00472F
C(#N)/C(/C(=O)O)=C\C=1SC(=CC1)C=1C=CC=2N(C3=CC=C(C=C3SC2C1)C1=CC2=CC=CC=C2C=C1)CCCCCC	THF	10	10.1039/C9TA00472F
C(#N)/C(/C(=O)O)=C\C=1SC(=CC1)C=1C=CC=2N(C3=CC=C(C=C3SC2C1)C1=CC2=CC=C(C=C2C=C1)OC)CCCCCC	THF	10	10.1039/C9TA00472F
C(#N)/C(/C(=O)O)=C\C=1SC(=CC1)C=1C=CC=2N(C3=CC=C(C=C3SC2C1)C1=CC2=CC=C(C=C2C=C1)OCCOCCOCCOC)CCCCCC	THF	7	10.1039/C9TA00472F
C(#N)/C(/C(=O)O)=C\C=1SC(=CC1)C=1C=CC=2N(C3=CC=C(C=C3SC2C1)C1=CC=C2C=CC3=CC=CC4=CC=C1C2=C34)CCCCCC	THF	0	10.1039/C9TA00472F
C(#N)/C(/C(=O)O)=C\C=1SC(=CC1)C=1C=CC=2N(C3=CC=CC=C3SC2C1)CCCCCC	THF	10	10.1039/C9TA00472F

SMILES	SOLVENT	SHIFT (nm)	DOI
C(CCCCCCC)SC=1SC(SC1SCCCCCCCC)=CC1=CC=C(C=C1)C1=CC=C(S1)C1=CC=C(C=C1)/C=C(/C(=O)O)\C#N	THF	-31	10.1039/C8TC06109B
C(CCCCCCC)SC=1SC(SC1SCCCCCCCC)=CC1=CC=C(C=C1)C=1C=C2OC=3C=C(C=CC3N(C2=CC1)CCCC)C1=CC=C(C=C1)/C=C(/C(=O)O)\C#N	THF	-44	10.1039/C8TC06109B
C(CC(C)C)OC1=CC=C(C=C1)N1C2=CC=CC=C2SC=2C=C(C=CC12)/C=C/C=1C=C2N=C(C(=NC2=CC1\C=C\C=1C=CC=2N(C3=CC=CC=C3SC2C1)C1=CC=C(C=C1)OCCC(C)C)C(=O)O)C(=O)O	THF	-37	10.1016/j.electacta.2018.12.133
C(CC(C)C)OC1=CC=C(C=C1)N1C2=CC=CC=C2SC=2C=C(C=CC12)/C=C/C1=C2N=C(C(=NC2=C(C=C1)\C=C\C=1C=CC=2N(C3=CC=CC=C3SC2C1)C1=CC=C(C=C1)OCCC(C)C)C(=O)O)C(=O)O	THF	-39	10.1016/j.electacta.2018.12.133
OB(O)C1=C2C=CC=CC2=CC2=CC(=CC=C12)C1=CC=C(C2=CC=C(O2)\C=C2\SC(=S)N(CC(O)=O)C2=O)C2=NSN=C12	THF	-95	10.1016/j.jphotochem.2018.12.004
OB(O)C1=CC=C(S1)C1=CC=C(S1)C1=CC=C(S1)C1=CC=C(C2=CC=C(O2)\C=C2\SC(=S)N(CC(O)=O)C2=O)C2=NSN=C12	THF	-73	10.1016/j.jphotochem.2018.12.004
OB(O)C1=C2C=CC3=CC=CC4=C3C2=C(C=C1)C=C4C1=CC=C(C2=CC=C(O2)\C=C2\SC(=S)N(CC(O)=O)C2=O)C2=NSN=C12	THF	-90	10.1016/j.jphotochem.2018.12.004
C(CCCCCCC)OC1=CC=C(C=C1)C1=NC2=C(C=CC(=C2N=C1C1=CC=C(C=C1)OCCCCCCCC)C1=CC=2C(=C3C(=C4N=C(C(=NC24)CCCCCCCC)CCCCCCCC)C=C(S3)C3=CC=C(S3)/C=C(/C(=O)O)\C#N)S1)C1=CC=3C2C(N(C3C=C1)C1=CC=C(C=C1)C)CCC2	THF	18	10.1007/s10854-018-0333-1
C(#N)/C(/C(=O)O)=C\C=1SC(=CC1)C1=CC2=C3N=C(C(=NC3=C3C(=C2S1)SC(=C3)C3=C1N=C(C(=NC1=C(C=C3)C3=CC=1C2C(N(C1C=C3)C3=CC=C(C=C3)C)CCC2)C2=CC=CC=C2)C2=CC=CC=C2)CCCCCCC)CCCCCCCC	THF	14	10.1007/s10854-018-0333-1
C(#N)/C(/C(=O)O)=C\C=1SC(=CC1)C1=CC2=C3N=C(C(=NC3=C3C(=C2S1)SC(=C3)C3=C1N=C2C4=C(C5=C(C2=NC1=C(C=C3)C3=CC=1C2C(N(C1C=C3)C3=CC=C(C=C3)C)CCC2)C=CC=C5)C=CC=C4)CCCCCCCC)CCCCCCCC	THF	-23	10.1007/s10854-018-0333-1

SMILES	SOLVENT	SHIFT (nm)	DOI
CCCCCCN1c2ccc(cc2Sc2c1ccc(c2)c1ccc(cc1)OC)c1ccc(cc1)/C=C(/C(=O)O)\C#N	THF	-3	10.1016/j.dyepig.2019.05.007
CCCCCCN1c2ccc(cc2Sc2c1ccc(c2)c1ccc(cc1)OC)c1ccc(s1)/C=C(/C(=O)O)\C#N	THF	0	10.1016/j.dyepig.2019.05.007
CCCCCCN1c2ccc(cc2Sc2c1cccc2)c1ccc(s1)/C=C(/C(=O)O)\C#N	THF	5	10.1016/j.dyepig.2019.05.007
CCCCCCN1c2ccc(cc2Sc2c1cccc2)c1ccc(cc1)/C=C(/C(=O)O)\C#N	THF	2	10.1016/j.dyepig.2019.05.007
CCCCCCN1c2ccc(cc2Sc2c1cc(cc2)c1ccc(cc1)OC)c1ccc(s1)/C=C(/C(=O)O)\C#N	THF	12	10.1016/j.dyepig.2019.05.007
CCCCCCc1cc(sc1c1cc(c(s1)/C=C(/C(=O)O)\C#N)CCCCCC)c1sc(cc1CCCCCC)c1ccc2c(c1)n(CC)c1c2cccc1	toluene	64	10.1021/jp808536v
CCCCCCCCn1c2ccc(cc2c2c1cc1c3ccccc3n(c1c2)CCCCCCCC)c1sc(c(c1)CCCCCC)c1sc(c(c1)CCCCCC)/C=C(/C(=O)O)\C#N	toluene	63	10.1021/jp808536v
CCCCCCCCn1c2ccc(cc2c2c1cc1c3ccccc3n(c1c2)CCCCCCCC)c1sc(c(c1)CCCCCC)c1sc(c(c1)CCCCCC)c1sc(c(c1)CCCCCC)/C=C(/C(=O)O)\C#N	toluene	64	10.1021/jp808536v
CCCCCCc1cc(sc1c1sc(c(c1)CCCCCC)c1sc(cc1CCCCCC)/C=C(/C(=O)O)\C#N)c1sc(cc1CCCCCC)c1sc2c(c1)n(c1c2cccc1)CC	toluene	39	10.1021/jp105548u
CCCCCCc1cc(sc1c1sc(c(c1)CCCCCC)/C=C(/C(=O)O)\C#N)c1cc2c(s1)c1c(n2CC)cccc1	toluene	44	10.1021/jp105548u
CCCCCCc1cc(sc1c1sc(c(c1)CCCCCC)/C=C(/C(=O)O)\C#N)c1sc(cc1CCCCCC)c1cc2c(s1)c1c(n2CC)cccc1	toluene	30	10.1021/jp105548u
CCCCCCC1(CCCCCC)c2cc(ccc2c2c1cc(cc2)N(c1ccccc1)c1ccccc1)c1sc(c(c1)C(C(C(C(C(C(F)(F)F)(F)F)(F)F)(F)F)(F)F)(F)F)/C=C(/C(=O)O)\C#N	toluene	-19	10.1002/ajoc.201800071
CCCCCCC1(CCCCCC)c2cc(ccc2c2c1cc(cc2)c1sc(c(c1)CCCCCC)/C=C(/C(=O)O)\C#N)N(c1ccccc1)c1ccccc1	toluene	2	10.1002/ajoc.201800071
CCCCCCC1(CCCCCC)c2cc(ccc2c2c1cc(cc2)N(c1ccccc1)c1ccccc1)c1ccc(s1)c1sc(c(c1)C(C(C(C(C(C(F)(F)F)(F)F)(F)F)(F)F)(F)F)(F)F)/C=C(/C(=O)O)\C#N	toluene	-22	10.1002/ajoc.201800071
CCCCCCC1(CCCCCC)c2cc(ccc2c2c1cc(cc2)N(c1ccccc1)c1ccccc1)c1cc(c(s1)c1ccc(s1)/C=C(/C(=O)O)\C#N)C(C(C(C(C(C(F)(F)F)(F)F)	toluene	0	10.1002/ajoc.201800071

SMILES	SOLVENT	SHIFT (nm)	DOI
(F)F)(F)F)(F)F)(F)F			
CCCCCCC1(CCCCCC)c2cc(ccc2c2c1cc(cc2)c1ccc(s1)c1sc(c(c1)CCCCCC)/C=C(/C(=O)O)\C#N)N(c1ccccc1)c1ccccc1	toluene	-7	10.1002/ajoc.201800071
CCCCCCC1(CCCCCC)c2cc(ccc2c2c1cc(cc2)c1sc(c(c1)CCCCCC)c1ccc(s1)/C=C(/C(=O)O)\C#N)N(c1ccccc1)c1ccccc1	toluene	13	10.1002/ajoc.201800071
CCCCCCOc1ccc(cc1)N(c1ccc(cc1)OCCCCCC)c1ccc(cc1)c1ccc(s1)c1ccc(c2c1nc(c1ccc(s1)/C=C(/C(=O)O)\C#N)c(n2)c1cccs1)c1ccc(s1)c1ccc(cc1)N(c1ccc(cc1)OCCCCCC)c1ccc(cc1)OCCCCCC	toluene	6	10.1021/am505153q
CCCCCCOc1ccc(cc1)N(c1ccc(cc1)OCCCCCC)c1ccc(cc1)c1ccc(s1)c1ccc(c2c1nc1c(n2)c2scc2c2c1sc(c2)/C=C(/C(=O)O)\C#N)c1ccc(s1)c1ccc(cc1)N(c1ccc(cc1)OCCCCCC)c1ccc(cc1)OCCCCCC	toluene	8	10.1021/am505153q
CCCCCCCCOc1ccc(cc1)N(c1ccc(cc1)OCCCCCCCC)c1ccc(cc1)c1ccc(s1)c1sc(c2c1nccn2)c1ccc(s1)C=C(C(=O)O)C#N	toluene	26	10.1021/am505153q
CCCCCCOc1ccc(cc1)N(c1ccc(cc1)OCCCCCC)c1ccc(cc1)c1ccc(s1)c1ccc(c2c1nc(c1ccc(s1)/C=C(\C(=O)O)/C#N)c(n2)c1ccc(s1)/C=C(/C(=O)O)\C#N)c1ccc(s1)c1ccc(cc1)N(c1ccc(cc1)OCCCCCC)c1ccc(cc1)OCCCCCC	toluene	3	10.1039/C3TA11398A
N#C/C(=C\c1ccc(cc1)c1ccc(c2c1nsn2)c1ccc2c(c1)[C@@H]1CCC[C@@H]1N2c1ccc(cc1)C)/C(=O)O	dichloromethane	5	10.1002/adfm.201001801
CCCCCCCCN1c2ccc(cc2C(/C/1=C\C1=C([O-])/C(=C\C2=[N+](CCCCCCCC)c3c(C2(C)C)cc(cc3)C(=O)O)/C1=O)(C)C)C(=O)O	dichloromethane	-2	10.1039/c2cc17187b
CCCCCCCC[N+]1=C(/C=C\2/C(=C(/C2=C(\C(=O)OCC)/C#N)/C=C/2\N(CCCCCCCO)c3c(C2(C)C)cc(cc3)C(=O)O)[O-])C(c2c1ccc(c2)C(=O)O)(C)C	dichloromethane	-7	10.1002/adfm.201303769
CCCCn1c(nc2c1c(ccc2c1ccc(cc1)N(c1ccccc1)c1ccccc1)c1ccc(cc1)N(c1ccccc1)c1ccccc1)c1ccc(s1)c1ccc(s1)/C=C(\C(=O)O)/C#N	THF	-52	10.1021/acs.joc.5b02590
CCCCn1c(nc2c1c(ccc2c1ccc(s1)c1ccc(s1)/C=C(/C(=O)O)\C#N)c1ccc(cc1)N(c1ccccc1)c1ccccc1)c1ccc(cc1)N(c1ccccc1)c1ccccc1	THF	-1	10.1021/acs.joc.5b02590

SMILES	SOLVENT	SHIFT (nm)	DOI
CCCCn1c(nc2c1c(ccc2c1ccc(cc1)N(c1ccccc1)c1ccccc1)c1ccc(s1)c1ccc(s1)C=C(C(=O)O)C#N)c1ccc(cc1)N(c1ccccc1)c1ccccc1	THF	-22	10.1021/acs.joc.5b02590
CCCCCCn1c2ccc(cc2c2c1c1ccccc1C2(CC)CC)c1ccc(s1)/C=C(/C(=O)O)\C#N	dichloromethane	20	10.1016/j.jpowsour.2016.09.138
CCCCCCn1c2ccc(cc2c2c1c1ccccc1C2(CC)CC)c1ccc(o1)/C=C(/C(=O)O)\C#N	dichloromethane	15	10.1016/j.jpowsour.2016.09.138
CCCCCCn1c2ccc(cc2c2c1c1ccccc1C2(CCCCCC)CCCCCC)c1ccc(s1)/C=C(/C(=O)O)\C#N	dichloromethane	16	10.1016/j.jpowsour.2016.09.138
CCCCCCn1c2ccc(cc2c2c1c1ccccc1C2(CCCCCC)CCCCCC)c1ccc(o1)/C=C(/C(=O)O)\C#N	dichloromethane	13	10.1016/j.jpowsour.2016.09.138
CCCCCCn1nc2c(n1)c1c(c3c2c2sc(cc2n3CCCCCC)c2ccc(cc2)N(c2ccccc2)c2ccccc2)n(c2c1sc(c2)/C=C(/C(=O)O)\C#N)CCCCCC	dichloromethane	71	10.1016/j.dyepig.2015.10.022
CCCCCCOc1ccc(cc1)N(c1ccc(cc1)OCCCCCC)c1ccc(cc1)c1sc2c(c1)n(c1c2c2nn(nc2c2c1n(CCCCCC)c1c2sc(c1)/C=C(/C(=O)O)\C#N)CCCCCC)CCCCCC	dichloromethane	84	10.1016/j.dyepig.2015.10.022
CCCCCCOc1ccc(cc1)c1ccc(cc1)N(c1ccc(cc1)c1ccc(cc1)OCCCCCC)c1ccc(cc1)c1sc2c(c1)n(c1c2c2nn(nc2c2c1n(CCCCCC)c1c2sc(c1)/C=C(/C(=O)O)\C#N)CCCCCC)CCCCCC	dichloromethane	85	10.1016/j.dyepig.2015.10.022
CCCCCCn1nc2c(n1)c1c3sc(cc3n(c1c1c2c2sc(cc2n1CC(CCCC)CC)c1ccc(cc1)N(c1ccccc1)c1ccccc1)CC(CCCC)CC)/C=C(/C(=O)O)\C#N	dichloromethane	57	10.1016/j.dyepig.2015.10.022
CCCCC(COc1cc(OCC(CCCC)CC)ccc1c1ccc(cc1)C(=Cc1ccc(cc1)N1C2CCCC2c2c1ccc(c2)c1cnc(c2c1nc(c1ccccc1)c(n2)c1ccccc1)c1sc2c(c1)C(c1c2sc(c1)/C=C(/C(=O)O)\C#N)(CC(CCCC)CC)CC(CCCC)CC)c1ccc(cc1)c1ccc(cc1OCC(CCCC)CC)OCC(CCCC)CC)CC	dichloromethane	21	10.1039/C6QM00119J
CCCCC(COc1cc(OCC(CCCC)CC)ccc1c1ccc(cc1)C(=Cc1ccc(cc1)N1c2ccc(cc2C2C1CCC2)c1cnc(c2c1nc(c1ccccc1)c(n2)c1ccccc1)c1sc(c2c1OCCO2)/C=C(/C(=O)O)\C#N)c1ccc(cc1)c1ccc(cc1OCC(CCCC)CC)OCC(CCCC)CC)CC	dichloromethane	31	10.1039/C6QM00119J
CCCCCCn1c2ccc(cc2c2c1ccc(c2)/C=C/1\SC(=S)NC1=O)c1ccc2c(c1)c1cc(ccc1n2CCCCCC)/C=C\1/SC(=S)NC1=O	THF	71	10.1007/s10854-017-8262-y
CCCCCCn1c2ccc(cc2c2c1ccc(c2)/C=C(\C(=O)O)/C#N)c1ccc2c(c1)c1cc(ccc1n2CCCCCC)/C=C(\C(=O)O)/C#N	THF	34	10.1007/s10854-017-8262-y

SMILES	SOLVENT	SHIFT (nm)	DOI
CCCCCCn1c2ccc(cc2c2c1ccc(c2)/C=C(\C(=O)OCC)/C#N)c1ccc2c(c1)c1cc(ccc1n2CCCCCC)/C=C(\C(=O)OCC)/C#N	THF	4	10.1007/s10854-017-8262-y
N#CC(=Cc1sc(c(c1)CO[Si](C(C)(C)C)(C)C)C=C1C=C(OC(=C1)c1ccccc1)c1ccccc1)C(=O)O	dichloromethane	48	10.1016/j.dyepig.2015.07.026
N#CC(=Cc1cc(c(s1)C=C1SC(=C(S1)C)C)CO[Si](C(C)(C)C)(C)C)C(=O)O	dichloromethane	50	10.1016/j.dyepig.2015.07.026
N#CC(=Cc1cc(c(s1)c1ccc(cc1)N(c1ccccc1)c1ccccc1)CO[Si](C(C)(C)C)(C)C)C(=O)O	dichloromethane	28	10.1016/j.dyepig.2015.07.026
N#C/C(=C\c1ccc(s1)c1cc2c3c(c1)C(c1ccc(cc1)C)(c1ccc(cc1)C)c1c4N3c3c(C2(c2ccc(cc2)C)c2ccc(cc2)C)cccc3C(c4ccc1)(c1ccc(cc1)C)c1ccc(cc1)C)/C(=O)O	dichloromethane	33	10.1016/j.synthmet.2015.03.033
CCCCCCc1ccc(s1)c1cc2c3c(c1)C(c1ccc(cc1)C)(c1ccc(cc1)C)c1c4N3c3c(C2(c2ccc(cc2)C)c2ccc(cc2)C)cc(cc3C(c4cc(c1)c1ccc(s1)/C=C(/C(=O)O)\C#N)(c1ccc(cc1)C)c1ccc(cc1)C)c1ccc(s1)CCCCCC	dichloromethane	27	10.1016/j.synthmet.2015.03.033
CCCCCCc1ccsc1c1cc2c3c(c1)C(c1ccc(cc1)C)(c1ccc(cc1)C)c1c4N3c3c(C2(c2ccc(cc2)C)c2ccc(cc2)C)cc(cc3C(c4cc(c1)c1ccc(s1)/C=C(/C(=O)O)\C#N)(c1ccc(cc1)C)c1ccc(cc1)C)c1sccc1CCCCCC	dichloromethane	33	10.1016/j.synthmet.2015.03.033
N#C/C(=C\c1ccc(cc1)c1cc2c3c(c1)C(c1ccc(cc1)C)(c1ccc(cc1)C)c1c4N3c3c(C2(c2ccc(cc2)C)c2ccc(cc2)C)cccc3C(c4ccc1)(c1ccc(cc1)C)c1ccc(cc1)C)/C(=O)O	dichloromethane	17	10.1016/j.tetlet.2015.01.156
N#C/C(=C\c1ccc(o1)c1cc2c3c(c1)C(c1ccc(cc1)C)(c1ccc(cc1)C)c1c4N3c3c(C2(c2ccc(cc2)C)c2ccc(cc2)C)cccc3C(c4ccc1)(c1ccc(cc1)C)c1ccc(cc1)C)/C(=O)O	dichloromethane	21	10.1016/j.tetlet.2015.01.156
COc1ccc(cc1)N(c1ccc(cc1)OC)c1ccc(cc1)c1ccc(o1)/C=C(/C(=O)O)\C#N	dichloromethane	43	10.1016/j.dyepig.2014.09.014
COc1ccc(c(c1)OC)N(c1ccc(cc1OC)OC)c1ccc(cc1)c1ccc(o1)C=C(C(=O)O)C#N	dichloromethane	48	10.1016/j.dyepig.2014.09.014
N#CC(=Cc1ccc(o1)c1ccc(cc1)N(c1ccc(c(c1)OC)OC)c1ccc(c(c1)OC)OC)C(=O)O	dichloromethane	48	10.1016/j.dyepig.2014.09.014
N#CC(=Cc1ccc(o1)c1ccc(cc1)N(c1ccc2c(c1)	dichloromethane	42	10.1016/

SMILES	SOLVENT	SHIFT (nm)	DOI
OCCO2)c1ccc2c(c1)OCCO2)C(=O)O			j.dyepig.2014.09.014
N#C/C(=C\c1ccc(s1)c1cc2c(s1)c(C#C[Si](C(C)C)(C(C)C)C(C)C)c1c(c2C#C[Si](C(C)C)(C(C)C)C(C)C)sc(c1)c1ccc(cc1)N(c1cccc2c1cccc2)c1ccccc1)/C(=O)O	THF	31	10.1016/j.dyepig.2014.04.043
CCCCCCOc1c2cc(sc2c(c2c1sc(c2)c1ccc(s1)/C=C(/C(=O)O)\C#N)OCCCCCC)c1ccc(cc1)N(c1cccc2c1cccc2)c1ccccc1	THF	13	10.1016/j.dyepig.2014.04.043
N#C/C(=C\c1ccc(s1)c1cc2c(o1)c(C#C[Si](C(C)C)(C(C)C)C(C)C)c1c(c2C#C[Si](C(C)C)(C(C)C)C(C)C)oc(c1)c1ccc(cc1)N(c1cccc2c1cccc2)c1ccccc1)/C(=O)O	THF	34	10.1016/j.dyepig.2014.04.043
CCCCCCOc1c2cc(oc2c(c2c1oc(c2)c1ccc(s1)/C=C(/C(=O)O)\C#N)OCCCCCC)c1ccc(cc1)N(c1cccc2c1cccc2)c1ccccc1	THF	13	10.1016/j.dyepig.2014.04.043
N#C/C(=C\c1ccc(s1)c1ccc(s1)c1ccc(s1)N(c1ccc(cc1)C)c1ccc(cc1)C)/C(=O)O	THF	44	10.1016/j.tsf.2014.03.010
COc1ccc(cc1)N(c1ccc(cc1)OC)c1ccc(s1)c1ccc(s1)c1ccc(s1)/C=C(/C(=O)O)\C#N	THF	43	10.1016/j.tsf.2014.03.010
N#C/C(=C\c1ccc(s1)c1ccc(s1)c1ccc(cc1)N(c1ccc(cc1)C)c1ccc(cc1)C)/C(=O)O	THF	44	10.1016/j.tsf.2014.03.010
N#C/C(=C\c1ccc(s1)c1ccc(s1)c1ccc(cc1)N(c1ccc(cc1)OC)c1ccc(cc1)OC)/C(=O)O	THF	48	10.1016/j.tsf.2014.03.010
N#C/C(=C\c1ccc(s1)c1ccc(cc1)c1ccc(cc1)N(c1ccc(cc1)C)c1ccc(cc1)C)/C(=O)O	THF	24	10.1016/j.tsf.2014.03.010
COc1ccc(cc1)N(c1ccc(cc1)OC)c1ccc(cc1)c1ccc(cc1)c1ccc(s1)/C=C(/C(=O)O)\C#N	THF	16	10.1016/j.tsf.2014.03.010
N#C/C(=C\c1ccc(cc1)c1ccc(cc1)c1ccc(cc1)N(c1ccc(cc1)C)c1ccc(cc1)C)/C(=O)O	THF	44	10.1016/j.tsf.2014.03.010
N#C/C(=C\c1ccc(cc1)c1ccc(cc1)c1ccc(cc1)N(c1ccc(cc1)OC)c1ccc(cc1)OC)/C(=O)O	THF	50	10.1016/j.tsf.2014.03.010
N#C/C(=C\c1ccc(s1)c1ccc2c(c1)c(C#C[Si](C(C)C)(C(C)C)C(C)C)c1c(c2C#C[Si](C(C)C)(C(C)C)C(C)C)cc(cc1)c1ccc(cc1)N(c1ccccc1)c1ccccc1)/C(=O)O	THF	8	10.1016/j.tet.2013.11.072
CCCCc1c2cc(ccc2c(c2c1ccc(c2)c1ccc(s1)/	THF	6	10.1016/j.tet.2013.11.072

SMILES	SOLVENT	SHIFT (nm)	DOI
C=C(/C(=O)O)\C#N)CCCC)c1ccc(cc1)N(c1ccccc1)c1ccccc1			
N#C/C(=C\c1ccc(cc1)c1ccc(s1)c1ccc(n1c1c(cccc1C(C)C)C(C)C)c1ccc(s1)c1ccc(cc1)N(c1ccccc1)c1ccccc1)/C(=O)O	THF	-20	10.1016/j.tet.2013.11.050
N#C/C(=C\c1ccc(cc1F)c1ccc(s1)c1ccc(n1c1c(cccc1C(C)C)C(C)C)c1ccc(s1)c1ccc(cc1)N(c1ccccc1)c1ccccc1)/C(=O)O	THF	-77	10.1016/j.tet.2013.11.050
N#C/C(=C\c1ccc(s1)c1ccc(c2c1nsn2)c1ccc(s1)n1c2ccc(cc2c2c1ccc(c2)c1ccc(cc1)C=Cc1ccc(cc1)N(c1ccccc1)c1ccccc1)c1ccc(cc1)C=Cc1ccc(cc1)N(c1ccccc1)c1ccccc1)/C(=O)O	DMF	-32	10.1016/j.tet.2013.08.075
N#CC(=Cc1ccc(s1)c1ccc(c2c1nsn2)c1ccc(s1)c1ccc(cc1)n1c2ccc(cc2c2c1ccc(c2)c1ccc(cc1)C=Cc1ccc(cc1)N(c1ccccc1)c1ccccc1)c1ccc(cc1)C=Cc1ccc(cc1)N(c1ccccc1)c1ccccc1)C(=O)O	DMF	-12	10.1016/j.tet.2013.08.075
CCCCCCc1cc(sc1c1ccc(c2c1nsn2)c1sc(cc1CCCCCC)C=C(C(=O)O)C#N)c1ccc(cc1)n1c2ccc(cc2c2c1ccc(c2)c1ccc(cc1)C=Cc1ccc(cc1)N(c1ccccc1)c1ccccc1)c1ccc(cc1)C=Cc1ccc(cc1)N(c1ccccc1)c1ccccc1	DMF	-44	10.1016/j.tet.2013.08.075
N#CC(=Cc1ccc(cc1)c1ccc2c(c1)sc(n2)c1ccc(cc1)N(c1ccc(cc1)OC)c1ccc(cc1)OC)C(=O)O	dichloromethane	8	10.1007/s11426-012-4758-8
N#CC(=Cc1ccc(s1)c1ccc2c(c1)sc(n2)c1ccc(cc1)N(c1ccc(cc1)OC)c1ccc(cc1)OC)C(=O)O	dichloromethane	41	10.1007/s11426-012-4758-8
N#CC(=Cc1ccc(o1)c1ccc2c(c1)sc(n2)c1ccc(cc1)N(c1ccc(cc1)OC)c1ccc(cc1)OC)C(=O)O	dichloromethane	44	10.1007/s11426-012-4758-8
N#C/C(=C\c1ccc(s1)c1ccc(cc1)N(c1ccc(cc1)n1c2ccc(cc2c2c1ccc(c2)C(C)(C)C)C(C)(C)C)c1ccc(cc1)n1c2ccc(cc2c2c1ccc(c2)C(C)(C)C)C(C)(C)C)/C(=O)O	dichloromethane	22	10.1002/ejoc.201201479
N#C/C(=C\c1ccc(s1)c1ccc(s1)c1ccc(cc1)N(c1ccc(cc1)n1c2ccc(cc2c2c1ccc(c2)C(C)(C)C)C(C)(C)C)c1ccc(cc1)n1c2ccc(cc2c2c1ccc(c2)C(C)(C)C)C(C)(C)C)/C(=O)O	dichloromethane	13	10.1002/ejoc.201201479
CCCCC(CC1(CC(CCCC)CC)c2cc(ccc2c2c1cc1c3ccc(cc3C(c1c2)(CC(CCCC)CC)CC(CCCC)CC)C=C(C(=O)O)C#N	THF	-2	10.1016/j.tet.2012.07.045

SMILES	SOLVENT	SHIFT (nm)	DOI
)N(c1ccccc1)c1ccccc1)CC			
CCCCC(CC1(CC(CCCC)CC)c2cc(ccc2c2c1cc1c3ccc(cc3C(c1c2)(CC(CCCC)CC)CC(CCCC)CC)N(c1ccccc1)c1ccccc1)c1ccc(s1)C=C(C(=O)O)C#N)CC	THF	1	10.1016/j.tet.2012.07.045
CCCCC(CC1(CC(CCCC)CC)c2cc(ccc2c2c1cc1c3ccc(cc3C(c1c2)(CC(CCCC)CC)CC(CCCC)CC)N(c1ccccc1)c1ccccc1)c1ccc(o1)C=C(C(=O)O)C#N)CC	THF	-28	10.1016/j.tet.2012.07.045
N#CC(=Cc1ccc(c2c1nsn2)c1ccc(s1)N(c1ccccc1)c1ccccc1)C(=O)O	dichloromethane	100	10.1016/j.tet.2012.05.052
N#CC(=Cc1ccc(c2c1nsn2)c1ccc(s1)N(c1ccc(cc1)OC)c1ccc(cc1)OC)C(=O)O	dichloromethane	102	10.1016/j.tet.2012.05.052
CCCCCCOc1ccc(cc1)N(c1ccc(s1)c1ccc(c2c1nsn2)C=C(C(=O)O)C#N)c1ccc(cc1)OCCCCCC	dichloromethane	107	10.1016/j.tet.2012.05.052
N#C/C(=C\ c1ccc(cc1)c1ccc(s1)c1ccc(cc1)N(c1ccc(cc1)C(C)(C)C)c1ccc(cc1)C(C)(C)C)/C(=O)O	THF	34	10.1002/asia.201100777
N#C/C(=C\ c1ccc(cc1)c1ccc(s1)c1ccc(cc1)N(c1ccc(cc1)C(C)(C)C)c1ccccc1)/C(=O)O	THF	32	10.1002/asia.201100777
N#C/C(=C\ c1ccc(cc1)c1ccc(s1)c1ccc2c(c1)CC(N2c1ccc(cc1)C(C)(C)C)C)/C(=O)O	THF	45	10.1002/asia.201100777
N#C/C(=C\ c1ccc(cc1)c1ccc(s1)c1ccc2c(c1)c1ccccc1n2c1ccc(cc1)C(C)(C)C)/C(=O)O	THF	10	10.1002/asia.201100777
N#C/C(=C\ c1ccc(cc1)c1ccc(s1)c1ccc2c(c1)CCCN2c1ccc(cc1)C(C)(C)C)/C(=O)O	THF	48	10.1002/asia.201100777
N#C/C(=C\ c1ccc(cc1)c1ccc(s1)c1ccc2c(c1)Sc1c(N2c2ccc(cc2)C(C)(C)C)cccc1)/C(=O)O	THF	38	10.1002/asia.201100777
N#CC(=Cc1ccc(s1)c1ccc(s1)n1c2ccc(cc2c2c1ccc(c2)c1ccc(cc1)n1c2ccccc2c2c1cccc2)c1ccc(cc1)n1c2ccccc2c2c1cccc2)C(=O)O	DMF	115	10.1002/asia.201100661
N#CC(=Cc1ccc(s1)c1ccc(s1)n1c2ccc(cc2c2c1ccc(c2)c1ccc(cc1)N(c1ccccc1)c1ccccc1)c1ccc(cc1)N(c1ccccc1)c1ccccc1)C(=O)O	DMF	96	10.1002/asia.201100661
N#CC(=Cc1ccc(s1)c1ccc(s1)n1c2ccc(cc2c2c1ccc(c2)c1ccc(cc1)C=Cc1ccc(cc1)N(c1ccccc1)c1ccccc1)c1ccc(cc1)C=Cc1ccc(cc1)N(c1ccccc1)c1ccccc1)C(=O)O	DMF	54	10.1002/asia.201100661
N#CC(=Cc1ccc(s1)c1ccc(s1)c1ccc(cc1)n1c2c	DMF	69	10.1002/asia.201100661

SMILES	SOLVENT	SHIFT (nm)	DOI
cc(cc2c2c1ccc(c2)c1ccc(cc1)C=Cc1ccc(cc1)N(c1ccccc1)c1ccccc1)c1ccc(cc1)C=Cc1ccc(cc1)N(c1ccccc1)c1ccccc1)C(=O)O			
CCCOc1cc(C=C(C(=O)O)C#N)c(cc1c1ccc(s1)c1ccc(cc1)N(c1ccccc1)c1ccccc1)OCCC	dichloromethane	32	10.1007/s10853-011-5971-0
N#CC(=Cc1c(F)cc(cc1F)c1ccc(s1)c1ccc(cc1)N(c1ccccc1)c1ccccc1)C(=O)O	dichloromethane	35	10.1007/s10853-011-5971-0
N#C/C(=C\c1ccc(cc1)/C=C/c1c(ccc2c1cccc2)N1CCCC1)/C(=O)O	ethanol	-3	10.1039/C2JM34363K
N#C/C(=C\c1ccc(cc1)/C=C/c1ccc2c(c1)ccc(c2)N(c1ccccc1)c1ccccc1)/C(=O)O	ethanol	-9	10.1039/C2JM34363K
N#C/C(=C\c1ccc(cc1)/C=C/c1c(ccc2c1cccc2)N(c1ccccc1)c1ccccc1)/C(=O)O	ethanol	4	10.1039/C2JM34363K
N#C/C(=C\c1ccc(cc1)/C=C/c1ccc2c(c1)ccc(c2)N1CCCC1)/C(=O)O	ethanol	2	10.1039/C2JM34363K
CCCCn1c2ccc(cc2c(=O)c2c1cc1c(c2)n(CCCC)c2c(c1=O)cccc2)c1ccc(s1)C=C(C(=O)O)C#N	methanol	112	10.5012/bkcs.2011.32.8.2553
CCCCn1c2ccc(cc2c(=O)c2c1cc1c(c2)n(CCCC)c2c(c1=O)cccc2)c1ccc(cc1)C=C(C(=O)O)C#N	methanol	122	10.5012/bkcs.2011.32.8.2553
CCOc1ccc(cc1)N(c1ccc(cc1)OCC)c1ccc(cc1)c1ccc(s1)c1ccc2c(c1)sc1c2ccnc1	DMSO	12	10.1016/j.dyepig.2015.08.012
CCOc1ccc(cc1)N(c1ccc(cc1)OCC)c1ccc(cc1)c1ccc(s1)c1ccc2c(c1)sc1c2cc[n+](c1)CCCS(=O)(=O)[O-]	DMSO	15	10.1016/j.dyepig.2015.08.012
CCCCCCCCn1c2ccc(cc2c2c1cccc2)c1ccc(cc1F)/C=C(/C(=O)O)\C#N	dichloromethane	-133	10.1016/j.orgel.2016.10.024
N#C/C(=C\c1ccc(c(c1)F)c1ccc(cc1)N(c1ccccc1)c1ccccc1)/C(=O)O	dichloromethane	-124	10.1016/j.orgel.2016.10.024
CCCCCCCCN1c2ccc(cc2Sc2c1cccc2)c1ccc(cc1F)/C=C(/C(=O)O)\C#N	dichloromethane	-138	10.1016/j.orgel.2016.10.024
CCCCCCCCn1c2cc(ccc2c2c1cc(cc2)c1cccs1)c1ccc(cc1F)/C=C(/C(=O)O)\C#N	dichloromethane	-165	10.1016/j.orgel.2016.10.024
CCCCCCCCOc1ccc(cc1)N(c1ccc(cc1)OCCCCCCCC)c1ccc(cc1)c1ccc(c2c1nccn2)c1sc(c2c1OCCO2)/C=C(\C(=O)O)/C#N	toluene	17	10.1039/C5TA01025J
CCCCCCCCOc1ccc(cc1)N(c1ccc(cc1)OCCCCCCCC)c1ccc(cc1)c1ccc(c2c1nccn2)c1sc(c2c1OCCO2)/C=C/1\SC(=S)N(C1=O)C(=O)O	toluene	26	10.1039/C5TA01025J
CCCCCCCCOc1ccc(cc1)N(c1ccc(cc1)OCCCCC	toluene	17	10.1039/C5TA01025J

SMILES	SOLVENT	SHIFT (nm)	DOI
CCC)c1ccc(cc1)c1sc(c2c1OCCO2)c1ccc(c2c1nccn2)c1sc(c2c1OCCO2)/C=C(\C(=O)O)/C#N			
CCCCCCCCOc1ccc(cc1)N(c1ccc(cc1)OCCCCCCCC)c1ccc(cc1)c1sc(c2c1OCCO2)c1ccc(c2c1nccn2)c1sc(c2c1OCCO2)/C=C/1\SC(=S)N(C1=O)C(=O)O	toluene	22	10.1039/C5TA01025J
CCCCC[Si]1(CCCCC)c2cc(ccc2c2c1cc(cc2)c1ccc(cc1)/C=C(\C(=O)O)/C#N)c1ccc2c(c1)C1CCCC1N2c1ccc(cc1)C	DMF	4	10.1039/C2JM30978E
CCCCC[Si]1(CCCCC)c2cc(ccc2c2c1cc(cc2)c1ccc(cc1)/C=C(\C(=O)O)/C#N)c1ccc2c(c1)C1CCCC1N2c1ccc(cc1)OC	DMF	11	10.1039/C2JM30978E
CCCCC[Si]1(CCCCC)c2cc(ccc2c2c1cc(cc2)c1ccc(cc1)/C=C(\C(=O)O)/C#N)c1ccc2c(c1)C1CCCC1N2c1cc(c(c(c1)C(C)(C)C)OC)C(C)(C)C	DMF	-1	10.1039/C2JM30978E
CCCCCC1(CCCCC)c2cc(ccc2c2c1cc(cc2)c1ccc(cc1)/C=C(\C(=O)O)/C#N)c1ccc2c(c1)C1CCCC1N2c1ccc(cc1)C	DMF	-11	10.1039/C2JM30978E
COC1=CC=C(C=C1)N1C2=CC=CC=C2C2=CC(=CC=C12)C1=CC2=C(S1)C1=C(S2)C=C(S1)C=C(C#N)C(O)=O	ethanol	-22	10.1016/j.tet.2013.02.058
CC1=CC=C(C=C1)N1C2CCCC2C2=CC(=CC=C12)C1=CC2=C(S1)C1=C(S2)C=C(S1)C=C(C#N)C(O)=O	ethanol	21	10.1016/j.tet.2013.02.058
COC1=CC=C(C=C1)N1C2CCCC2C2=CC(=CC=C12)C1=CC2=C(S1)C1=C(S2)C=C(S1)C=C(C#N)C(O)=O	ethanol	15	10.1016/j.tet.2013.02.058
COC1=CC=C(C=C1)N1C2=CC=CC=C2C2=CC(=CC=C12)C1=CC2=C(S1)C1=C(S2)C=C(S1)C=C(C#N)C(O)=O	dioxane	-11	10.1016/j.tet.2013.02.058
CC1=CC=C(C=C1)N1C2CCCC2C2=CC(=CC=C12)C1=CC2=C(S1)C1=C(S2)C=C(S1)C=C(C#N)C(O)=O	dioxane	33	10.1016/j.tet.2013.02.058
COC1=CC=C(C=C1)N1C2CCCC2C2=CC(=CC=C12)C1=CC2=C(S1)C1=C(S2)C=C(S1)C=C(C#N)C(O)=O	dioxane	27	10.1016/j.tet.2013.02.058
CCCCC(COc1ccc(cc1)c1cc2ccc(c3c2n2c1ccc2c(c3)c1ccc(cc1)OCC(CCCC)CC)C=C(C(=O)O)C#N)CC	chloroform	10	10.1039/C6RA11918B
CCCCC(COc1ccc(cc1)c1cc2ccc(c3c2n2c1ccc2c(c3)c1ccc(cc1)OCC(CCCC)CC)C=Cc1ccc(cc1)C(=O)O)CC	chloroform	10	10.1039/C6RA11918B

SMILES	SOLVENT	SHIFT (nm)	DOI
CCCCC(COc1ccc(cc1)c1cc2ccc(c3c2n2c1ccc2c(c3)c1ccc(cc1)OCC(CCCC)CC)C=Cc1ccc(s1)C(=O)O)CC	chloroform	29	10.1039/C6RA11918B
CCCCn1c2cc(ccc2c2c1cc(cc2)c1ccc(s1)c1ccc2n1[B-](F)(F)[N+]1=C(C=CC1C2c1ccc(s1)/C=C(/c1ccncc1)\C#N)c1ccc(s1)c1ccc2c(c1)n(CCCC)c1c2ccc(c1)N(c1ccccc1)c1ccccc1)N(c1ccccc1)c1ccccc1	THF	-7	10.1039/C7RA00799J
CCCCn1c2cc(ccc2c2c1cc(cc2)c1ccc(s1)c1ccc2n1[B-](F)(F)[N+]1=C(C=CC1C2c1ccc(s1)/C=C(/C(=O)O)\C#N)c1ccc(s1)c1ccc2c(c1)n(CCCC)c1c2ccc(c1)N(c1ccccc1)c1ccccc1)N(c1ccccc1)c1ccccc1	THF	0	10.1039/C7RA00799J
N#CC(=Cc1ccc(cc1)n1c2ccccc2c2c1cccc2)C(=O)O	chloroform	-42	10.1016/j.dyepig.2016.08.013
N#CC(=Cc1ccc(cc1)c1ccc(c2c1nsn2)C#Cc1ccc(cc1)n1c2ccccc2c2c1cccc2)C(=O)O	chloroform	-52	10.1016/j.dyepig.2016.08.013
CCCCCCOc1ccc(cc1)N(c1ccc(cc1)OCCCCCC)c1ccc(cc1)c1cc2c(s1)c1c(n2c2ccc(cc2)CCCCCC)c2c(s1)c1c(n2c2ccc(cc2)CCCCCC)cc(s1)C=C(C(=O)O)C#N	dichloromethane	75	10.1039/C4TA06705C
CCCCC(C[Ge]1(CC(CCCC)CC)c2cc(sc2c2c1cc(s2)c1ccc(cc1)N(c1ccccc1)c1ccccc1)c1cnc(c2c1nsn2)c1ccc(s1)c1ccncc1)CC	THF	-12	10.1039/C6DT02469F
CCCCC(C[Ge]1(CC(CCCC)CC)c2cc(sc2c2c1cc(s2)c1cnc(c2c1nsn2)c1ccc(s1)C(=O)O)c1ccc(cc1)N(c1ccccc1)c1ccccc1)CC	THF	6	10.1039/C6DT02469F
CCCCC(C[Ge]1(CC(CCCC)CC)c2cc(sc2c2c1cc(s2)c1ccc(cc1)N(c1ccccc1)c1ccccc1)c1cnc(c2c1nsn2)c1ccc(s1)/C=C(\C(=O)O)/C#N)CC	THF	-40	10.1039/C6DT02469F
CCCCC(CN1C(=O)/C(=C\2/C(=O)N(C3C2C=CC(=C3)c2ccc(s2)/C=C(\C(=O)O)/C#N)CC(CCCC)CC)/c2c1cc(cc2)c1ccc(s1)c1ccc(cc1)N1c2ccccc2C=C(c2c1cccc2)OC)CC	chloroform	23	10.1016/j.dyepig.2016.01.004
CCCCC(CN1C(=O)/C(=C\2/C(=O)N(C3C2C=CC(=C3)c2ccc(s2)/C=C(\C(=O)O)/C#N)CC(CCCC)CC)/c2c1cc(cc2)c1ccc(s1)c1ccc(cc1)N(c1ccc(cc1)N1c2ccccc2C=C(c2c1cccc2)OC)c1ccc(cc1)N1c2ccccc2C=C(c2c1cccc2)OC)CC	chloroform	40	10.1016/j.dyepig.2016.01.004
CCCCC(C[Ge]1(CC(CCCC)CC)c2cc(sc2c2c1cc(s2)c1cnc(c2c1nsn2)c1ccc(s1)c1ccncc1)c1c	THF	-28	10.1246/cl.161034

SMILES	SOLVENT	SHIFT (nm)	DOI
cc(cc1)N(c1ccc(cc1)OCCCCCC)c1ccc(cc1)OCCCCCC)CC			
CCCCC(C[Ge]1(CC(CCCC)CC)c2cc(sc2c2c1cc(s2)c1cnc(c2c1nsn2)c1ccc(cc1)c1ccncc1)c1ccc(cc1)N(c1ccc(cc1)OCCCCCC)c1ccc(cc1)OCCCCCC)CC	THF	-14	10.1246/cl.161034
CCCCCCN1c2ccc(cc2Sc2c1ccc(c2)/C=C(/C(=O)O)\C#N)C#Cc1ccc2c3c1ccc1c3c(cc2)ccc1	acetonitrile	-87	10.1039/C7TA01744H
CCCCCCCCN1c2ccc(cc2Sc2c1ccc(c2)/C=C(/C(=O)O)\C#N)C#Cc1ccc2c3c1ccc1c3c(cc2)ccc1	acetonitrile	-72	10.1039/C7TA01744H
CCCCCCN1c2ccc(cc2Sc2c1ccc(c2)/C=C\1/SC(=S)N(C1=O)CC(=O)O)C#Cc1ccc2c3c1ccc1c3c(cc2)ccc1	acetonitrile	-100	10.1039/C7TA01744H
CCCCCCCCN1c2ccc(cc2Sc2c1ccc(c2)/C=C\1/SC(=S)N(C1=O)CC(=O)O)C#Cc1ccc2c3c1ccc1c3c(cc2)ccc1	acetonitrile	-109	10.1039/C7TA01744H
CCCCCCN1c2ccc(cc2Sc2c1ccc(c2)C=C(C#N)C#N)C#Cc1ccc2c3c1ccc1c3c(cc2)ccc1	acetonitrile	46	10.1039/C7TA01744H
CCCCCCCCN1c2ccc(cc2Sc2c1ccc(c2)C=C(C#N)C#N)C#Cc1ccc2c3c1ccc1c3c(cc2)ccc1	acetonitrile	56	10.1039/C7TA01744H
CCCCCC1=C2C(=C(C(=[N+]2[B-](n2c1c(C)c(c2C)C=C(C(=O)O)C#N)(F)F)C)c1ccc2c(c1)c1ccccc1n2CCCCCCCC)C	chloroform	35	10.1039/C7RA04402J
CCCCCC1=C2C(=C(C(=[N+]2[B-](n2c1c(C)c(c2C)C=C(C(=O)O)C#N)(F)F)C)c1ccc2c(c1)c1ccccc1n2c1ccc(cc1)OC)C	chloroform	27	10.1039/C7RA04402J
CCCCCC1=C2C(=C(C(=[N+]2[B-](n2c1c(C)c(c2C)C=C(C(=O)O)C#N)(F)F)C)c1ccc2c(c1)n(CCCCCCCC)c1c2cccc1)C	chloroform	30	10.1039/C7RA04402J
CCCCCC1=C2C(=C(C(=[N+]2[B-](n2c1c(C)c(c2C)C=C(C(=O)O)C#N)(F)F)C)c1ccc2c(c1)n(c1ccc(cc1)OC)c1c2cccc1)C	chloroform	19	10.1039/C7RA04402J
CCCCC(Cn1c2nc3ccccc3nc2c2c1cc(cc2)c1ccc(s1)C=C(C(=O)O)C#N)CC	dichloromethane	59	10.1016/j.jpowsour.2016.06.127
CCCCC(CN1c2cc(ccc2Sc2c1cccc2)c1ccc(s1)C=C(C(=O)O)C#N)CC	dichloromethane	42	10.1016/j.jpowsour.2016.06.127
CCCCC(CN1c2ccc(cc2Sc2c1cc(cc2)c1ccc(s1)C=C(C(=O)O)C#N)c1ccc2c(c1)n(CC(CCCC)CC	dichloromethane	39	10.1016/j.jpowsour.2016.06.127

SMILES	SOLVENT	SHIFT (nm)	DOI
)c1c2nc2c(n1)cccc2)CC			
CCCCC(CN1c2ccc(cc2Sc2c1cc(cc2)c1ccc(o1)C=C(C(=O)O)C#N)c1ccc2c(c1)n(CC(CCCC)CC)c1c2nc2c(n1)cccc2)CC	dichloromethane	26	10.1016/j.jpowsour.2016.06.127
N#C/C(=C\c1ccc(cc1)c1ccc(c2c1nccn2)c1ccc2c(c1)C1CCCC1N2c1ccc(cc1)C)/C(=O)O	dichloromethane	16	10.1039/C5TC01195G
N#C/C(=C\c1ccc(s1)c1ccc(c2c1nccn2)c1ccc2c(c1)C1CCCC1N2c1ccc(cc1)C)/C(=O)O	dichloromethane	-42	10.1039/C5TC01195G
N#C/C(=C\c1ccc(cc1)c1ccc(c2c1nc(c1ccc(s1)C)c(n2)c1ccc(s1)C)c1ccc2c(c1)C1CCCC1N2c1ccc(cc1)C)/C(=O)O	dichloromethane	-5	10.1039/C5TC01195G
N#C/C(=C\c1ccc(s1)c1ccc(c2c1nc(c1ccc(s1)C)c(n2)c1ccc(s1)C)c1ccc2c(c1)C1CCCC1N2c1ccc(cc1)C)/C(=O)O	dichloromethane	-61	10.1039/C5TC01195G
CCCCCCn1c2cc(sc2c2c1cc(s2)C=C(C(=O)O)C#N)c1ccc(cc1)N(c1ccc(cc1)OC)c1ccc(cc1)OC	dichloromethane	53	10.1021/acs.jpcc.6b06604
CCCCCC/C=C/c1cc(ccc1c1cc2c(s1)c1c(n2CCCCCC)cc(s1)C=C(C(=O)O)C#N)N(c1ccc(cc1)OC)c1ccc(cc1)OC	dichloromethane	34	10.1021/acs.jpcc.6b06604
CCCCCCn1c2cc(ccc2c2c1cccc2)N(c1ccc2c(c1)C(CCCCCC)(CCCCCC)c1c2c2c(c3c1c1ccccc1C3(CCCCCC)CCCCCC)c1c(C2(CCCCCC)CCCCCC)cccc1)c1ccc(cc1)c1cc2c(s1)c1c(n2CCCCCC)cc(s1)C=C(C(=O)O)C#N	dichloromethane	40	10.1021/acs.jpcc.6b06604
CCCCCC/C=C/c1cc(ccc1c1cc2c(s1)c1c(n2CCCCCC)cc(s1)C=C(C(=O)O)C#N)N(c1ccc2c(c1)C(CCCCCC)(CCCCCC)c1c2c2c(c3c1c1ccccc1C3(CCCCCC)CCCCCC)c1c(C2(CCCCCC)CCCCCC)cccc1)c1ccc2c(c1)n(CCCCCC)c1c2cccc1	dichloromethane	35	10.1021/acs.jpcc.6b06604
OC(=O)CN1C(=S)S/C(=C/c2ccc(s2)c2cc3c4c(c2)C(c2ccc(cc2)C)(c2ccc(cc2)C)c2c5N4c4c(C3(c3ccc(cc3)C)c3ccc(cc3)C)cccc4C(c5ccc2)(c2ccc(cc2)C)c2ccc(cc2)C)/C1=O	dichloromethane	71	10.1002/cjoc.201500137
N#CC(=Cc1ccc(s1)C=Cc1cc2c3c(c1)C(c1ccc(cc1)C)(c1ccc(cc1)C)c1c4N3c3c(C2(c2ccc(cc2)C)c2ccc(cc2)C)cccc3C(c4ccc1)	dichloromethane	42	10.1002/cjoc.201500137

SMILES	SOLVENT	SHIFT (nm)	DOI
(c1ccc(cc1)C)c1ccc(cc1)C)C(=O)O			
N#CC(=Cc1ccc(s1)c1cc2c3c(c1)C(c1ccc(cc1)C)(c1ccc(cc1)C)c1c4N3c3c(C2(c2ccc(cc2)C)c2ccc(cc2)C)cc(cc3C(c4cc(c1)c1ccc(s1)C=C(C(=O)O)C#N)(c1ccc(cc1)C)c1ccc(cc1)C)c1ccc(s1)C=C(C(=O)O)C#N)C(=O)O	dichloromethane	42	10.1002/cjoc.201500137
N#CC(=Cc1ccc(s1)c1cc2c3c(c1)C(c1ccc(cc1)C)(c1ccc(cc1)C)c1c4N3c3c(C2(c2ccc(cc2)C)c2ccc(cc2)C)cccc3C(c4ccc1)(c1ccc(cc1)C)c1ccc(cc1)C)C(=O)O	dichloromethane	35	10.1002/cjoc.201500137
CCCC(CN1c2ccc(cc2Sc2c1cccc2)c1ccc(s1)c1ccc(s1)c1ccc(s1)/C=C(/C(=O)O)\C#N)CC	dichloromethane	35	10.1016/j.jpowsour.2016.01.055
CCCCCCCCc1cc(sc1c1ccc(s1)c1sc(cc1CCCCCCCC)/C=C(/C(=O)O)\C#N)c1ccc2c(c1)Sc1c(N2CC(CCC)CC)cccc1	dichloromethane	33	10.1016/j.jpowsour.2016.01.055
CCCCCCCCc1cc(sc1c1ccc(s1)c1sc(cc1CCCCCCCC)/C=C(/C(=O)O)\C#N)c1ccc2c(c1)Sc1c(N2CC(CCC)CC)ccc(c1)c1ccc(cc1)OCCCC	dichloromethane	11	10.1016/j.jpowsour.2016.01.055
CCCCC(Cn1c2cc(c3ccc(cc3)N(c3ccccc3)c3ccccc3)c3c4c2c2c1cc(c1ccc(s1)C=C(C(=O)O)C#N)c1c2c(c4ccc3)ccc1)CC	dichloromethane	-6	10.1039/C5CC00650C
CCCCC(Cn1c2cc(c3ccc(cc3)N(c3ccc(cc3)OC)c3ccc(cc3)OC)c3c4c2c2c1cc(c1ccc(s1)C=C(C(=O)O)C#N)c1c2c(c4ccc3)ccc1)CC	dichloromethane	-10	10.1039/C5CC00650C
CCCCC(Cn1c2cc(c3ccc(cc3)N(c3ccc(cc3)OC)c3ccc(cc3)OC)c3c4c2c2c1cc(c1ccc(s1)c1ccc(s1)/C=C(/C(=O)O)\C#N)c1c2c(c4ccc3)ccc1)CC	dichloromethane	0	10.1039/C5CC00650C
CCCCC(Cn1c2cc(c3ccc(cc3)N(c3ccc(cc3)OC)c3ccc(cc3)OC)c3c4c2c2c1cc(C=C(C(=O)O)C#N)c1c2c(c4ccc3)ccc1)CC	dichloromethane	28	10.1039/C5CC00650C
CCCCCCOc1ccc(cc1)N(c1ccc(c2c1nsn2)c1cc2c(s1)c1c(n2CCCCCC)cc(s1)C=C(C(=O)O)C#N)c1ccc(cc1)OCCCCCC	dichloromethane	26	10.1016/j.jpowsour.2016.09.141
CCCCCCOc1ccc(cc1)N(c1ccc(c2c1nsn2)c1sc2c(c1)n(c1c2sc(c1)C=C(C(=O)O)C#N)c1ccc(cc1)OCCCCCC)c1ccc(cc1)OCCCCCC	dichloromethane	12	10.1016/j.jpowsour.2016.09.141
CCCCCCOc1ccc(cc1)N(c1ccc(c2c1nsn2)c1sc(c2c1OCCO2)C=C(C(=O)O)C#N)c1ccc(cc1)OCCCCCC	dichloromethane	21	10.1016/j.jpowsour.2016.09.141

SMILES	SOLVENT	SHIFT (nm)	DOI
N#CC(=Cc1cnc(nc1)Nc1ccc(cc1)/C=C/c1ccc(cc1)N(c1ccc(cc1)OC)c1ccc(cc1)OC)C(=O)O	toluene	-20	10.1016/j.jphotochem.2018.09.033
CCCCCCCCOc1ccc(cc1)N(c1ccc(cc1)OCCCCCCCC)c1ccc(cc1)/C=C/c1ccc(cc1)Nc1ncc(cn1)C=C(C(=O)O)C#N	toluene	-17	10.1016/j.jphotochem.2018.09.033
CCCCCCc1ccc(cc1)C1(c2ccc(cc2)CCCCCC)c2cc(sc2c2c1cc1c3sc(cc3C(c1c2)(c1ccc(cc1)CCCCCC)c1ccc(cc1)CCCCCC)c1ccc(cc1)c1ccc(cc1)C(=O)O)c1ccc(cc1)N(c1ccc(cc1)c1ccc(cc1OCCCCCCCC)OCCCCCCCC)c1ccc(cc1)c1ccc(cc1OCCCCCCCC)OCCCCCCCC	toluene	6	10.1002/anie.201808609
CCCCCCc1ccc(cc1)C1(c2ccc(cc2)CCCCCC)c2cc(sc2c2c1cc1c3sc(cc3C(c1c2)(c1ccc(cc1)CCCCCC)c1ccc(cc1)CCCCCC)c1c(F)c(F)c(c2c1nsn2)c1ccc(cc1)C(=O)O)c1ccc(cc1)N(c1ccc(cc1)c1ccc(cc1OCCCCCCCC)OCCCCCCCC)c1ccc(cc1)c1ccc(cc1OCCCCCCCC)OCCCCCCCC	toluene	28	10.1002/anie.201808609
CCCCCCc1ccc(cc1)C1(c2ccc(cc2)CCCCCC)c2cc(sc2c2c1cc1c3sc(cc3C(c1c2)(c1ccc(cc1)CCCCCC)c1ccc(cc1)CCCCCC)c1ccc(c2c1nsn2)c1ccc(cc1)C(=O)O)c1ccc(cc1)N(c1ccc(cc1)c1ccc(cc1OCCCCCCCC)OCCCCCCCC)c1ccc(cc1)c1ccc(cc1OCCCCCCCC)OCCCCCCCC	toluene	44	10.1002/anie.201808609
CCCCCCc1ccc(cc1)C1(c2ccc(cc2)CCCCCC)c2cc(sc2c2c1cc1c3sc(cc3C(c1c2)(c1ccc(cc1)CCCCCC)c1ccc(cc1)CCCCCC)c1ccc(c2c1non2)c1ccc(cc1)C(=O)O)c1ccc(cc1)N(c1ccc(cc1)c1ccc(cc1OCCCCCCCC)OCCCCCCCC)c1ccc(cc1)c1ccc(cc1OCCCCCCCC)OCCCCCCCC	toluene	36	10.1002/anie.201808609
CCCCCCOc1ccc(cc1)N(c1ccc(cc1)OCCCCCC)c1ccc(cc1)c1ccc(c2c1nsn2)C#Cc1ccc(cc1)C(=O)O	dichloromethane	6	10.1002/cssc.201701949
CCCCn1c2cc(ccc2c2c1cc(cc2)N(c1ccccc1)c1ccccc1)c1ccc(s1)c1cccnc1	dioxane	-4	10.1002/ejoc.201500341
CCCCn1c2cc(ccc2c2c1cc(cc2)N(c1ccccc1)c1ccccc1)c1ccc(s1)c1cncc(c1)c1ccc(s1)c1ccc2c(c1)n(CCCC)c1c2ccc(c1)N(c1ccccc1)c1ccccc1	dioxane	-12	10.1002/ejoc.201500341
CCCCCCCC[N+]1=C(C=C2C(=C(C2=O)/C=C\2/N(CC)c3c(C2(C)C)cc(cc3)N(c2ccc(cc2)C)c2cc	ethanol	2	10.1016/j.dyepig.2014.03.008

SMILES	SOLVENT	SHIFT (nm)	DOI
c(cc2)C)[O-])C(c2c1ccc(c2)C(=O)O)(C)C			
CCCCCCCC[N+]1=C(C=C2C(=C(C2=O)/C=C\2/N(CC)c3c(C2(C)C)cc(cc3)N(c2ccc(cc2)OC)c2ccc(cc2)OC)[O-])C(c2c1ccc(c2)C(=O)O)(C)C	ethanol	43	10.1016/j.dyepig.2014.03.008
CCCCCCCC[N+]1=C(/C=C/2\C(=C(C2=O)/C=C\2/N(CC)c3c(C2(C)C)cc(cc3)N(c2ccc(cc2)OCCCCCC)c2ccc(cc2)OCCCCCC)[O-])C(c2c1ccc(c2)C(=O)O)(C)C	ethanol	35	10.1016/j.dyepig.2014.03.008
CCCCCCCC[NH+]1c2cc(ccc2C(C1C=C1C(=C(C1=O)C=C1N(CC)c2c(C1(C)C)cccc2)O)(C)C)C(=O)O	ethanol	0	10.1016/j.dyepig.2014.03.008
CCCCN(c1ccc2c(c1)OC1=NC(n3c(C1=C2)nc1c3cccc1)c1ccc(c(c1)C(=O)O)O)CCCC	DMSO	12	10.1016/j.solener.2008.10.002
CCN(c1ccc2c(c1)OC1=NC(n3c(C1=C2)nc1c3cccc1)c1ccc(c(c1)C(=O)O)O)CC	DMSO	13	10.1016/j.solener.2008.10.002
OC(=O)c1cc(ccc1O)C1N=C2Oc3c(C=C2c2n1c1ccccc1n2)cc1c2c3CCCN2CCC1	DMSO	47	10.1016/j.solener.2008.10.002
CCCCN(c1ccc2c(c1)OC1=NC(n3c(C1=C2)nc1c3cccc1)c1ccc(cc1)C(=O)O)CCCC	DMSO	15	10.1016/j.solener.2008.10.002
CCN(c1ccc2c(c1)OC1=NC(n3c(C1=C2)nc1c3cccc1)c1ccc(cc1)C(=O)O)CC	DMSO	14	10.1016/j.solener.2008.10.002
OC(=O)c1ccc(cc1)C1N=C2Oc3c(C=C2c2n1c1ccccc1n2)cc1c2c3CCCN2CCC1	DMSO	48	10.1016/j.solener.2008.10.002
CCCCCCSC1=C(SCCCCCC)SC(=Cc2ccc(cc2)c2ccc(s2)c2ccncc2)S1	THF	-107	10.1007/s40843-016-5119-1
CCCCCCSC1=C(SCCCCCC)SC(=Cc2ccc(s2)c2ccc(cc2)c2ccncc2)S1	THF	-90	10.1007/s40843-016-5119-1
CCCCCCSC1=C(SCCCCCC)SC(=C(C(=C2SC(=C(S2)SCCCCCC)SCCCCCC)c2ccc(cc2)c2ccc(s2)c2ccncc2)c2ccc(cc2)c2ccc(s2)c2ccncc2)S1	THF	-98	10.1007/s40843-016-5119-1
CCCCCCSC1=C(SCCCCCC)SC(=C(C(=C2SC(=C(S2)SCCCCCC)SCCCCCC)c2ccc(s2)c2ccc(cc2)c2ccncc2)c2ccc(s2)c2ccc(cc2)c2ccncc2)S1	THF	-87	10.1007/s40843-016-5119-1
CCCCCCN1c2ccc(cc2Sc2c1ccc(c2)C=C(C(=O)O)C#N)c1ccc(cc1)C=Cc1ccccc1	dichloromethane	-29	10.1039/c2ra20819a
CCCCCCN1c2ccc(cc2Sc2c1ccc(c2)C=C(C(=O)O)C#N)c1ccc(cc1)C=C(c1ccccc1)c1ccccc1	dichloromethane	-16	10.1039/c2ra20819a
CCCCCCN1c2ccc(cc2Sc2c1ccc(c2)C=C(C(=O)O)C#N)c1ccc(cc1)C(=C(c1ccccc1)c1ccccc1)c1ccccc1	dichloromethane	-26	10.1039/c2ra20819a
CCCCCCn1c2ccc(cc2c2c1ccc(c2)C=C(C(=O)O	dichloromethane	2	10.1039/c2jm30254c

SMILES	SOLVENT	SHIFT (nm)	DOI
)C#N)c1ccc(cc1)C=C(c1ccc(cc1)n1c2ccccc2c2c1cccc2)c1ccc(cc1)n1c2ccccc2c2c1cccc2			
CCCCCCn1c2ccc(cc2c2c1ccc(c2)C=C(C(=O)O)C#N)c1ccc(cc1)C=C(c1ccc(cc1)N1c2ccccc2Sc2c1cccc2)c1ccc(cc1)N1c2ccccc2Sc2c1cccc2	dichloromethane	2	10.1039/c2jm30254c
CCCCCCN1c2ccc(cc2Sc2c1ccc(c2)C=C(C(=O)O)C#N)c1ccc(cc1)C=C(c1ccc(cc1)n1c2ccccc2c2c1cccc2)c1ccc(cc1)n1c2ccccc2c2c1cccc2	dichloromethane	7	10.1039/c2jm30254c
CCCCCCN1c2ccc(cc2Sc2c1ccc(c2)C=C(C(=O)O)C#N)c1ccc(cc1)C=C(c1ccc(cc1)N1c2ccccc2Sc2c1cccc2)c1ccc(cc1)N1c2ccccc2Sc2c1cccc2	dichloromethane	6	10.1039/c2jm30254c
N#CC(=Cc1ccc(s1)c1sc(cc1c1ccc(s1)/C=C(\C(=O)O)/C#N)c1ccc(cc1)N(c1ccccc1)c1ccccc1)C(=O)O	THF	62	10.1039/C3TA14404F
N#CC(=Cc1ccc(s1)c1sc(cc1c1ccc(s1)/C=C(\C(=O)O)/C#N)c1ccc(s1)c1ccc(cc1)N(c1ccccc1)c1ccccc1)C(=O)O	THF	68	10.1039/C3TA14404F
N#CC(=Cc1ccc(cc1)c1sc(cc1c1ccc(cc1)/C=C(\C(=O)O)/C#N)c1ccc(s1)c1ccc(cc1)N(c1ccccc1)c1ccccc1)C(=O)O	THF	41	10.1039/C3TA14404F
CCCCCCOc1ccc(cc1)N(c1ccc(cc1)OCCCCCC)c1ccc(cc1)c1ccc(s1)c1cc(c(s1)c1ccc(cc1)C=C(C(=O)O)C#N)c1ccc(cc1)/C=C(\C(=O)O)/C#N	THF	52	10.1039/C3TA14404F
CCCCCCn1c2cc(ccc2c2c1cccc2)c1sc(c(c1)c1ccc(s1)/C=C(\C(=O)O)/C#N)c1ccc(s1)C=C(C(=O)O)C#N	THF	72	10.1039/C3TA14404F
Cc1ccc(cc1)N1C2CCCC2c2c1ccc(c2)c1ccc(s1)/C=C\1/SC(=NC1=O)N	dichloromethane	-9	10.1039/C6RA04915J
O=C1NC(=O)C(=Cc2ccc(s2)c2ccc3c(c2)C2CCCC2N3c2ccc(cc2)C)C(=O)N1	dichloromethane	28	10.1039/C6RA04915J
S=C1NC(=O)C(=Cc2ccc(s2)c2ccc3c(c2)N(c2ccc(cc2)C)C2C3CCC2)C(=O)N1	dichloromethane	40	10.1039/C6RA04915J
CCCCCCc1ccc(s1)c1cc(c(s1)c1ccc(s1)c1ccc(s1)c1sc(cc1CCCCCC)c1ccc(s1)P(=O)(O)O)CCCCCC	chloroform	-14	10.1021/am501515s
CCCCCCc1ccc(s1)c1sc(cc1CCCCCC)c1ccc(c2c1nsn2)c1cc(c(s1)c1ccc(s1)P(=O)	chloroform	-6	10.1021/am501515s

SMILES	SOLVENT	SHIFT (nm)	DOI
(O)O)CCCCCC			
C(C)C(CN1C(/C(/C2=CC=C(C=C12)C1=CC(=C(S1)C=1SC(=CC1)P(O)(O)=O)CCCCCC)=C\1/C(N(C2=CC(=CC=C12)C1=CC=C(S1)C=1SC(=CC1)CCCCCC)CC(CCCC)CC)=O)=O)CCCC	chloroform	0	10.1021/am501515s
N#C/C(=C\c1ccc2c(c1)c1c(n2C)ccc2c1c1c3ccccc3n(c1cc2)C)/C(=O)O	chloroform	25	10.1016/j.solmat.2009.11.014
CCCCCCCCn1c2ccccc2c2c1ccc1c2c2c3cc(ccc3n(c2cc1)CCCCCCCC)/C=C(/C(=O)O)\C#N	chloroform	15	10.1016/j.solmat.2009.11.014
C(#N)/C(/C(=O)O)=C\C1=CC=C(C=C1)N(C1=CC=CC=C1)C1=CC=2C(C3=CC(=CC=C3C2C=C1)N(C1=CC=CC=C1)C1=CC=CC=C1)(CC)CC	dichloromethane	-28	10.1021/jo200501b
C(#N)/C(/C(=O)O)=C\C1=CC=C(C=C1)N(C1=CC=CC2=CC=CC=C12)C1=CC=2C(C3=CC(=CC=C3C2C=C1)N(C1=CC=CC=C1)C1=CC=CC2=CC=CC=C12)(CC)CC	dichloromethane	-30	10.1021/jo200501b
C1=CC=CC=2C3=CC=CC=C3N(C12)C1=CC=C2C=3C=CC(=CC3C(C2=C1)(CC)CC)N1C2=CC=CC=C2C=2C=C(C=CC12)/C=C(/C(=O)O)\C#N	dichloromethane	-32	10.1021/jo200501b
N#CC(=Cc1ccc(cc1)c1ccc(s1)c1ccc(cc1)N(c1ccccc1)c1ccccc1)C(=O)O	THF	28	10.1016/j.tet.2009.09.036
N#CC(=Cc1ccc(cc1)c1ccc(s1)c1ccc(cc1)N(c1ccc(cc1)C)c1ccc(cc1)C)C(=O)O	THF	29	10.1016/j.tet.2009.09.036
COc1ccc(cc1)N(c1ccc(cc1)OC)c1ccc(cc1)c1ccc(s1)c1ccc(cc1)C=C(C(=O)O)C#N	THF	24	10.1016/j.tet.2009.09.036
N#CC(=Cc1ccc(s1)c1ccc(cc1)c1ccc(cc1)N(c1ccccc1)c1ccccc1)C(=O)O	THF	18	10.1016/j.tet.2009.09.036
N#CC(=Cc1ccc(s1)c1ccc(cc1)c1ccc(cc1)N(c1ccc(cc1)C)c1ccc(cc1)C)C(=O)O	THF	24	10.1016/j.tet.2009.09.036
COc1ccc(cc1)N(c1ccc(cc1)OC)c1ccc(cc1)c1ccc(cc1)c1ccc(s1)C=C(C(=O)O)C#N	THF	16	10.1016/j.tet.2009.09.036
N#CC(=Cc1ccc(n1c1c(F)c(F)c(c(c1F)F)F)/C=C/c1ccc(cc1)N(c1ccccc1)c1ccccc1)C(=O)O	dichloromethane	45	10.1016/j.dyepig.2013.05.030
N#CC(=Cc1ccc(n1c1ccc(cc1)C#N)/C=C/c1ccc(cc1)N(c1ccccc1)c1ccccc1)C(=O)O	dichloromethane	32	10.1016/j.dyepig.2013.05.030
COc1ccc(cc1)N(c1ccc(cc1)OC)c1ccc(cc1)c1ccc(s1)C=C(C(=O)O)C#N	dichloromethane	79	10.1021/jp2054519
CCCCCCOc1ccc(cc1)N(c1ccc(cc1)c1ccc(s1)C	dichloromethane	83	10.1021/jp2054519

SMILES	SOLVENT	SHIFT (nm)	DOI
=C(C(=O)O)C#N)c1ccc(cc1)OCCCCCC			
CCCCCCCCCCOc1ccc(cc1)N(c1ccc(cc1)c1ccc(s1)C=C(C(=O)O)C#N)c1ccc(cc1)OCCCCCCCCCC	dichloromethane	94	10.1021/jp2054519
CCCCCCn1c2ccc(cc2c2c1ccc(c2)c1ccc(cc1)/C=C(/C(=O)O)\C#N)c1ccc(cc1)/C=C(/C(=O)O)\C#N	DMF	-29	10.1016/j.orgel.2013.11.020
CCCCCCn1c2ccc(cc2c2c1ccc(c2)c1ccc(s1)/C=C(/C(=O)O)\C#N)c1ccc(s1)C=C(C(=O)O)C#N	DMF	-12	10.1016/j.orgel.2013.11.020
CCCCCCn1c2ccc(cc2c2c1ccc(c2)c1ccc(o1)/C=C(/C(=O)O)\C#N)c1ccc(o1)C=C(C(=O)O)C#N	DMF	31	10.1016/j.orgel.2013.11.020
N#C/C(=C\c1ccc(s1)c1ccc(cc1)N(c1ccc(cc1)c1cccs1)c1ccc(cc1)c1cccs1)/C(=O)O	dichloromethane	51	10.1016/j.synthmet.2013.02.010
CCCCCCc1ccc(s1)c1ccc(cc1)N(c1ccc(cc1)c1ccc(s1)/C=C(/C(=O)O)\C#N)c1ccc(cc1)c1ccc(s1)CCCCCC	dichloromethane	49	10.1016/j.synthmet.2013.02.010
N#C/C(=C\c1ccc(s1)c1ccc(cc1)N(c1ccc(cc1)c1ccccc1)c1ccc(cc1)c1ccccc1)/C(=O)O	dichloromethane	38	10.1016/j.synthmet.2013.02.010
N#C/C(=C\c1ccc(cc1)c1ccc(cc1)N(c1ccccc1)c1ccccc1)/C(=O)O	dichloromethane	41	10.1016/j.synthmet.2013.02.010
N#C/C(=C/c1ccc(s1)c1ccc(cc1)N(c1ccccc1)c1ccccc1)/C(=O)O	dichloromethane	57	10.1016/j.synthmet.2013.02.010
CCCCn1c2cc(ccc2c2c1cc(cc2)N(c1ccc2c(c1)C(CCC)(CCC)c1c2cccc1)c1ccccc1)c1ccc(s1)/C=C(/C(=O)O)\C#N	dichloromethane	28	10.1016/j.dyepig.2015.07.034
CCCCn1c2cc(ccc2c2c1cc(cc2)N(c1ccc2c(c1)C(CC)(CC)c1c2cccc1)c1ccc2c(c1)C(CC)(CC)c1c2cccc1)c1ccc(s1)/C=C(/C(=O)O)\C#N	dichloromethane	17	10.1016/j.dyepig.2015.07.034
CCCCn1c2cc(ccc2c2c1cc(cc2)c1ccc(s1)c1ccc(s1)/C=C(/C(=O)O)\C#N)N(c1ccc2c(c1)C(CCC)(CCC)c1c2cccc1)c1ccccc1	dichloromethane	50	10.1016/j.dyepig.2015.07.034
CCCCn1c2cc(ccc2c2c1cc(cc2)c1ccc(s1)c1ccc(s1)/C=C(/C(=O)O)\C#N)N(c1ccc2c(c1)C(CC)(CC)c1c2cccc1)c1ccc2c(c1)C(CC)(CC)c1c2cccc1	dichloromethane	7	10.1016/j.dyepig.2015.07.034
CCCCn1c2cc(ccc2c2c1cc(cc2)N(c1ccccc1)c1	dichloromethane	-32	10.1039/C4RA15234D

SMILES	SOLVENT	SHIFT (nm)	DOI
ccccc1)c1ccc(s1)c1ccc(cc1)/C=C(/C(=O)O)\C#N			
CCCCn1c2cc(ccc2c2c1cc(cc2)N(c1ccccc1)c1ccccc1)c1ccc(s1)c1ccc(s1)c1ccc(s1)/C=C(/C(=O)O)\C#N	dichloromethane	-47	10.1039/C4RA15234D
CCCCn1c2cc(ccc2c2c1cc(cc2)N(c1ccccc1)c1ccccc1)c1ccc(s1)c1ccc2c(c1)C(CC)(CC)c1c2ccc(c1)/C=C(/C(=O)O)\C#N	dichloromethane	-30	10.1039/C4RA15234D
CCCCn1c2cc(ccc2c2c1cc(cc2)N(c1ccccc1)c1ccccc1)c1ccc(s1)c1ccc2c(c1)n(CCCC)c1c2ccc(c1)/C=C(/C(=O)O)\C#N	dichloromethane	-26	10.1039/C4RA15234D
N#CC(=Cc1ccc(cc1)c1ccc(cc1)N(c1ccc(cc1C)C)c1ccc(cc1)c1ccc(cc1)C=C(C(=O)O)C#N)C(=O)O	chloroform	-17	10.1002/slct.201800977
N#CC(=Cc1ccc(s1)c1ccc(cc1)N(c1ccc(cc1C)C)c1ccc(cc1)c1ccc(s1)C=C(C(=O)O)C#N)C(=O)O	chloroform	-24	10.1002/slct.201800977
CCCCCCCCn1c2ccc(cc2c2c1ccc(c2)C=C(C(=O)O)C#N)c1ccc(cc1)N(c1ccc(cc1C)C)c1ccc(cc1)c1ccc2c(c1)c1cc(ccc1n2CCCCCCCC)C=C(C(=O)O)C#N	chloroform	-45	10.1002/slct.201800977
CCCCCCCCN1c2ccc(cc2Sc2c1ccc(c2)C=C(C(=O)O)C#N)c1ccc(cc1)N(c1ccc(cc1C)C)c1ccc(cc1)c1ccc2c(c1)Sc1c(N2CCCCCCCC)ccc(c1)C=C(C(=O)O)C#N	chloroform	5	10.1002/slct.201800977
COc1ccc(cc1)N(c1ccc(cc1)OC)c1ccc(cc1)c1c(C)c2n(c1C)[B-](F)(F)[N+]1=C(C(=C(C1=C2C)C)c1ccc(s1)C=C(C(=O)O)C#N)C	chloroform	28	10.1002/chem.201702285
COc1ccc(cc1)N(c1ccc(cc1)OC)c1ccc(cc1)c1c(C)c2n(c1C)[B-](F)(F)[N+]1=C(C(=C(C1=C2C)C)c1cc2c(s1)cc(s2)C=C(C(=O)O)C#N)C	chloroform	23	10.1002/chem.201702285
COc1ccc(cc1)N(c1ccc(cc1)OC)c1ccc(cc1)c1c(C)c2n(c1C)[B-]1(Oc3c(O1)cccc3)[N+]1=C(C(=C(C1=C2C)C)c1ccc(s1)C=C(C(=O)O)C#N)C	chloroform	16	10.1002/chem.201702285
COc1ccc(cc1)N(c1ccc(cc1)OC)c1ccc(cc1)c1c(C)c2n(c1C)[B-](F)(F)[N+]1=C(C(=C(C1=C2C)C)c1ccc(s1)c1cc[n+](cc1)[O-])C	chloroform	12	10.1002/chem.201702285
COc1ccc(cc1)N(c1ccc(cc1)OC)c1ccc(cc1)c1c(C)c2n(c1C)[B-](F)(F)[N+]1=C(C(=C(C1=C2C)C)c1cc2c(s1)cc(s2)c1	chloroform	18	10.1002/chem.201702285

SMILES	SOLVENT	SHIFT (nm)	DOI
cc[n+](cc1)[O-])C			
COc1ccc(cc1)N(c1ccc(s1)c1c(C)c2n(c1C)[B-](F)(F)[N+]1=C(C(=C(C1=C2C)C)c1ccc(s1)C=C(C(=O)O)C#N)C)c1ccc(cc1)OC	chloroform	0	10.1002/chem.201702285
N#CC(=Cc1ccc(s1)C1=C(C)C2=C(C)c3n([B-]([N+]2=C1C)(F)F)c(c(c3C)c1ccc(cc1)N(c1ccccc1)c1ccccc1)C)C(=O)O	chloroform	23	10.1002/chem.201702285
CCCCCCn1c2cc(ccc2c2c1cc(cc2)C=C(C(=O)O)C#N)c1ccc(cc1)N(c1ccc(cc1)C(C)(C)C)c1ccc(cc1)C(C)(C)C	dichloromethane	45	10.1016/j.dyepig.2015.09.004
CCCCCCn1c2cc(ccc2c2c1cc(cc2)c1ccc2c(c1)c1cc(ccc1n2c1ccc(cc1)C(C)(C)C)C(C)(C)C)C=C(C(=O)O)C#N	dichloromethane	37	10.1016/j.dyepig.2015.09.004
CCCCCCn1c2cc(ccc2c2c1cc(cc2)C=C(C(=O)O)C#N)c1ccc(cc1)n1c2ccc(cc2c2c1ccc(c2)C(C)(C)C)C(C)(C)C	dichloromethane	26	10.1016/j.dyepig.2015.09.004
CCCCCCn1c2cc(ccc2c2c1cc(cc2)c1ccc(c2c1nsn2)c1ccc(cc1)N(c1ccc(cc1)C(C)(C)C)c1ccc(cc1)C(C)(C)C)C=C(C(=O)O)C#N	dichloromethane	4	10.1016/j.dyepig.2015.09.004
CCCCCCCCn1nc2c(n1)c(ccc2c1ccc2c(c1)c1cccc3c1n2c1ccccc1C3(CC)CC)c1ccc(s1)/C=C(/C(=O)O)\C#N	THF	5	10.1039/c3ra43057j
CCCCCCCCn1nc2c(n1)c(ccc2c1ccc2c(c1)c1cccc3c1n2c1ccccc1C3(CC)CC)c1cc2c(s1)cc(s2)/C=C(/C(=O)O)\C#N	THF	12	10.1039/c3ra43057j
CCCCCCCCn1nc2c(n1)c(ccc2c1ccc(s1)c1ccc2c(c1)c1cccc3c1n2c1ccccc1C3(CC)CC)c1ccc(s1)/C=C(/C(=O)O)\C#N	THF	13	10.1039/c3ra43057j
CCCCCCc1cc(sc1/C=C(/C(=O)O)\C#N)c1ccc(c2c1nsn2)c1ccc2c(c1)c1cccc3c1n2c1ccccc1C3(CC)CC	THF	-6	10.1039/c3ra43057j
CCCCCCc1cc(sc1c1ccc2c(c1)c1cccc3c1n2c1ccccc1C3(CC)CC)c1ccc(c2c1nsn2)c1sc(c(c1)CCCCCC)/C=C(/C(=O)O)\C#N	THF	-2	10.1039/c3ra43057j
OC(=O)CN1C(=S)SC(=Cc2ccc(cc2)N(c2ccccc2)N=Cc2ccc(cc2)N(c2ccccc2)c2ccccc2)C1=O	chloroform	-6	10.1021/jp500527d
Cc1ccc(cc1)N(c1ccccc1)c1ccc(cc1)C=NN(c1ccc(cc1)C=C1SC(=S)N(C1=O)CC(=O)O)c1ccccc1	chloroform	-9	10.1021/jp500527d
CCCCCCc1ccc(cc1)N(c1ccc(cc1)CCCCCC)c1ccc(cc1)C=NN(c1ccc(cc1)C=C1SC(=S)N(C1=O)CC(=O)O)c1ccccc1	chloroform	-2	10.1021/jp500527d

SMILES	SOLVENT	SHIFT (nm)	DOI
CCCN(c1ccc(cc1)C=C1SC(=S)N(C1=O)CC(=O)O)N=Cc1ccc(cc1)N(c1ccc(cc1)C)c1ccccc1	chloroform	1	10.1021/jp500527d
CCCCCCCCCN(c1ccc(cc1)C=C1SC(=S)N(C1=O)CC(=O)O)N=Cc1ccc(cc1)N(c1ccc(cc1)C)c1ccccc1	chloroform	17	10.1021/jp500527d
CCCCCCCCCN(c1ccc(cc1)C=C1SC(=S)N(C1=O)CC(=O)O)N=Cc1ccc(cc1)N(c1ccc(cc1)C=NN(c1ccccc1)CCCCCCCCC)c1ccccc1	chloroform	0	10.1021/jp500527d
Cc1ccc(cc1)N(c1ccc(cc1)C=NN(c1ccc(cc1)C=C1SC(=S)N(C1=O)CC(=O)O)c1ccccc1)c1ccc(cc1)C=NN(c1ccc(cc1)C=C1SC(=S)N(C1=O)CC(=O)O)c1ccccc1	chloroform	-9	10.1021/jp500527d
N#C/C(=C/1\C=C(/C=C/c2ccc3c(c2)C(C)CC(N3C)(C)C)Oc2c1cccc2)/C(=O)O	ethanol	-38	10.1039/c3ra41583j
N#C/C(=C/1\C=C(/C=C/c2cc3C(C)CC(N(c3cc2OC)C)(C)C)Oc2c1cccc2)/C(=O)O	ethanol	-38	10.1039/c3ra41583j
CCCCCCC1CC(C)(CCCCCC)N(c2c1cc(/C=C/C1=C/C(=C(/C(=O)O)\C#N)/c3c(O1)cccc3)c(c2)OC)C	ethanol	-41	10.1039/c3ra41583j
N#CC(=Cc1ccc(s1)/C=C/c1ccc2c(c1)C(C)CC(N2C)(C)C)C(=O)O	ethanol	-38	10.1039/c3ra41583j
CCCCCCC1(CCCCCC)c2cc(sc2c2c1cc(s2)c1ccc2c(c1)nc(c(c2)/C=C(\C(=O)O)/C#N)Cl)c1ccc(cc1)N(c1ccc(cc1)OCCCCCC)c1ccc(cc1)OCCCCCC	dichloromethane	22	10.1021/jp5004352
CCCCCCC1(CCCCCC)c2cc(sc2c2c1cc(s2)c1ccc2c(c1)[nH]c(=O)c(c2)/C=C(\C(=O)O)/C#N)c1ccc(cc1)N(c1ccc(cc1)OCCCCCC)c1ccc(cc1)OCCCCCC	dichloromethane	49	10.1021/jp5004352
CCCCCCC1(CCCCCC)c2cc(sc2c2c1cc(s2)c1ccc2c(c1)[nH]c(=O)c(c2)C=C(C#N)C#N)c1ccc(cc1)N(c1ccc(cc1)OCCCCCC)c1ccc(cc1)OCCCCCC	dichloromethane	50	10.1021/jp5004352
CCCCCCC1(CCCCCC)c2cc(sc2c2c1cc(s2)c1ccc2c(c1)[nH]c(=O)c(c2)C(=O)O)c1ccc(cc1)N(c1ccc(cc1)OCCCCCC)c1ccc(cc1)OCCCCCC	dichloromethane	34	10.1021/jp5004352
CCCCCCC1(CCCCCC)c2cc(sc2c2c1cc(s2)c1ccc2c(c1)nc(c(c2)C(=O)O)Cl)c1ccc(cc1)N(c1ccc(cc1)OCCCCCC)c1ccc(cc1)OCCCCCC	dichloromethane	53	10.1021/jp5004352
N#CC(=Cc1ccc(cc1)N(c1ccc(cc1)c1nc(c([nH]1)c1ccccc1)c1ccccc1)c1ccccc1)c1ccc(cc1)	DMF	45	10.1016/j.matlet.2019.01.091

SMILES	SOLVENT	SHIFT (nm)	DOI
[N+](=O)[O-]			
OC(=O)CN1C(=S)SC(=Cc2ccc(cc2)N(c2ccc(cc2)c2nc(c([nH]2)c2ccccc2)c2ccccc2)c2ccccc2)C1=O	DMF	-3	10.1016/j.matlet.2019.01.091
N#CC(=Cc1ccc(cc1)N(c1ccccc1)c1ccc(cc1)c1nc(c([nH]1)c1ccccc1)c1ccccc1)C(=O)O	DMF	-30	10.1016/j.matlet.2019.01.091
CCCCC(Cn1c(c2ccc(s2)c2ccc3c(c2)C2CCCC2N3c2ccc(cc2)C=C(c2ccc(cc2)c2ccc(cc2OCCCCCC)OCCCCCC)c2ccc(cc2)c2ccc(cc2OCCCCCC)OCCCCCC)c2c(c1=O)c(n(c2=O)CC(CCCC)CC)c1ccc(cc1)c1ccc(cc1)C=C(C(=O)O)C#N)CC	dichloromethane	-40	10.1021/acssuschemeng.5b00332
CCCCC(Cn1c(c2ccc(s2)c2ccc3c(c2)C2CCCC2N3c2ccc(cc2)C=C(c2ccc(cc2)c2ccc(cc2OCCCCCC)OCCCCCC)c2ccc(cc2)c2ccc(cc2OCCCCCC)OCCCCCC)c2c(c1=O)c(n(c2=O)CC(CCCC)CC)c1ccc(cc1)c1ccc(cc1)C(=O)O)CC	dichloromethane	-47	10.1021/acssuschemeng.5b00332
CCCCC1(CCCC)C(=[N+](c2c1cccc2)CCC(=O)O)C=C1C(=C(C1=O)c1ccc2c(c1)C1CCCC1N2c1ccc2c(c1)C(C)(C)c1c2cccc1)[O-]	DMSO	-7	10.1021/ol300054a
CCCCC1(CCCC)C(=[N+](c2c1cccc2)CCC(=O)O)C=C1C(=C(C1=O)c1ccc2c(c1)C1CCCC1N2c1ccc(cc1)C=C(c1ccccc1)c1ccccc1)[O-]	DMSO	-7	10.1021/ol300054a
N#CC(=Cc1ccc(s1)c1ccc2c3c1cccc3c1c2c(c2ccccc2)c2c(c1c1ccccc1)cccc2)C(=O)O	dichloromethane	5	10.1016/j.dyepig.2009.02.006
OC(=O)CN1C(=S)SC(=Cc2ccc(s2)c2ccc3c4c2cccc4c2c3c(c3ccccc3)c3c(c2c2ccccc2)cccc3)C1=O	dichloromethane	-15	10.1016/j.dyepig.2009.02.006
N#CC(=Cc1ccc(cc1)C#Cc1ccc2c3c1cccc3c1c2c(c2ccccc2)c2c(c1c1ccccc1)cccc2)C(=O)O	dichloromethane	1	10.1016/j.dyepig.2009.02.006
CCCCCCn1c2ccc(cc2c2c1ccc(c2)/C=C(/c1ccc(cc1)c1ccc(cc1)/C=C\1/SC(=S)N(C1=O)CC(=O)O)\C#N)C=C(c1ccc(cc1)c1ccc(cc1)/C=C/1\SC(=S)N(C1=O)CC(=O)O)C#N	DMF	5	10.1016/j.dyepig.2013.01.014
CCCCCCN(c1ccc(cc1)/C=C(/c1ccc(s1)/C=C(/C(=O)O)\C#N)\C#N)c1ccc(cc1)/C=C(/c1ccc(s1)/C=C(/C(=O)O)\C#N)\C#N	DMF	12	10.1016/j.dyepig.2013.01.014
CCCCCCc1cc(sc1c1ccc(cc1)N(c1ccc(cc1)OCCCCCC)c1ccc(cc1)OCCCCCC)c1ccc(c2c1nsn2)c1sc(c(c1)CCCCCC)c1ccc(cc1)C=C(C(=O)O)C#N	dichloromethane	0	10.1016/j.electacta.2014.09.138
CCCCCCc1cc(sc1c1ccc(cc1)N(c1ccc(cc1)OCC	dichloromethane	25	10.1016/

SMILES	SOLVENT	SHIFT (nm)	DOI
CCCC)c1ccc(cc1)OCCCCCC)c1ccc(c2c1nsn2)c1sc(c(c1)CCCCCC)c1ccc(s1)/C=C(/C(=O)O)\C#N			j.electacta.2014.09.138
CCCC1(CCC)c2cc(ccc2c2c1c1c3ccccc3C(c1c1c2C(CCC)(CCC)c2c1cccc2)(CCC)CCC)N(c1ccc(cc1)c1sc(c2c1OCCO2)/C=C(/C(=O)O)\C#N)c1ccc(cc1)c1ccc(cc1)OC	dichloromethane	47	10.1016/j.tet.2013.10.041
CCCC1(CCC)c2cc(ccc2c2c1cccc2)N(c1ccc2c(c1)C(CCC)(CCC)c1c2c2c(c3c1c1ccccc1C3(CCC)CCC)c1c(C2(CCC)CCC)cccc1)c1ccc(cc1)c1sc(c2c1OCCO2)/C=C(/C(=O)O)\C#N	dichloromethane	50	10.1016/j.tet.2013.10.041
CCCCCCCCN1c2ccccc2C(C1=CC1=C([O-])C(=CC2=[N+](CCCCCCCC)c3c(C2(C)C)cc(cc3)C(=O)O)C1=O)(C)C	ethanol	-21	10.1016/ j.electacta.2014.06.073
CCCCCCCC[N+]1=C(C=C2C(=C(C2=O)C=C2N(C)c3c(C2(C)C)cccc3)[O-])C(c2c1ccc(c2)C(=O)O)(C)C	ethanol	-13	10.1016/ j.electacta.2014.06.073
CCCCCCN1c2ccc(cc2Sc2c1cccc2)/C=C(/C(=O)O)\C#N	methanol	-47	10.1016/j.orgel.2015.09.009
CCCCCCN1c2cc(ccc2Sc2c1ccc(c2)/C=C(/C(=O)O)\C#N)C=C1SC(=C(S1)SC)SC	methanol	-24	10.1016/j.orgel.2015.09.009
CCCCCCN1c2cc(ccc2Sc2c1ccc(c2)/C=C(/C(=O)O)\C#N)C=C1SC(=C(S1)SCCCCCC)SCCCCCC	methanol	-12	10.1016/j.orgel.2015.09.009
CCCCCCOc1ccc(cc1)N(c1ccc(cc1)OCCCCCC)c1ccc(cc1)c1ccc(c2c1nc(c1ccccc1)c(n2)c1ccccc1)c1sc2c(c1)sc(c2)/C=C(/C(=O)O)\C#N	DMF	1	10.1016/j.tet.2014.02.087
CCCCCCOc1ccc(cc1)N(c1ccc(cc1)OCCCCCC)c1ccc(cc1)c1ccc(c2c1nc(c1ccccc1)c(n2)c1ccccc1)c1ccc(s1)/C=C(/C(=O)O)\C#N	DMF	-3	10.1016/j.tet.2014.02.087
N#C/C(=C\c1ccc(s1)c1ccc(c2c1nc(c1ccccc1)c(n2)c1ccccc1)c1ccc(cc1)N(c1cccc2c1cccc2)c1ccccc1)/C(=O)O	DMF	-3	10.1016/j.tet.2014.02.087
N#C/C(=C\c1sc2c(c1)sc(c2)c1ccc(c2c1nc(c1ccccc1)c(n2)c1ccccc1)N(c1cccc2c1cccc2)c1ccccc1)/C(=O)O	DMF	3	10.1016/j.tet.2014.02.087
N#CC(=Cc1ccc(o1)c1ncc(c2c1nsn2)c1ccc(cc1)N(c1ccccc1)c1ccccc1)C(=O)O	THF	37	10.1016/j.orgel.2013.11.031
N#CC(=Cc1ccc(s1)c1ncc(c2c1nsn2)c1ccc(cc1)N(c1ccccc1)c1ccccc1)C(=O)O	THF	46	10.1016/j.orgel.2013.11.031

SMILES	SOLVENT	SHIFT (nm)	DOI
N#CC(=Cc1ccc(cc1)c1ncc(c2c1nsn2)c1ccc(cc1)N(c1ccccc1)c1ccccc1)C(=O)O	THF	26	10.1016/j.orgel.2013.11.031
N#CC(=Cc1ccc(cc1)c1ncc(c2c1nsn2)c1ccc(s1)c1ccc(cc1)N(c1ccccc1)c1ccccc1)C(=O)O	THF	38	10.1016/j.orgel.2013.11.031
N#CC(=Cc1ccc(o1)c1cnc(c2c1nsn2)c1ccc(cc1)N(c1ccccc1)c1ccccc1)C(=O)O	THF	28	10.1016/j.orgel.2013.11.031
N#CC(=Cc1ccc(s1)c1cnc(c2c1nsn2)c1ccc(cc1)N(c1ccccc1)c1ccccc1)C(=O)O	THF	44	10.1016/j.orgel.2013.11.031
N#CC(=Cc1ccc(cc1)c1cnc(c2c1nsn2)c1ccc(cc1)N(c1ccccc1)c1ccccc1)C(=O)O	THF	27	10.1016/j.orgel.2013.11.031
N#CC(=Cc1ccc(cc1)c1cnc(c2c1nsn2)c1ccc(s1)c1ccc(cc1)N(c1ccccc1)c1ccccc1)C(=O)O	THF	26	10.1016/j.orgel.2013.11.031
CCCCCCOc1ccc(cc1)N(c1ccc(cc1)OCCCCCC)c1ccc(cc1)c1ncc(c2c1nsn2)c1ccc(cc1)C=C(C(=O)O)C#N	THF	62	10.1016/j.orgel.2013.11.031
CCCCCCOc1ccc(cc1)N(c1ccc(cc1)OCCCCCC)c1ccc(cc1)c1ccc(s1)c1ncc(c2c1nsn2)c1ccc(s1)C=C(C(=O)O)C#N	THF	42	10.1016/j.orgel.2013.11.031
N#CC(=Cc1ccc(s1)c1ccc(c2c1nsn2)c1ccc(cc1)n1c2ccccc2c2c1cccc2)C(=O)O	THF	-5	10.1016/j.dyepig.2013.07.002
N#CC(=Cc1ccc(s1)c1ccc(c2c1nsn2)c1ccc2c(c1)c1ccccc1n2c1ccccc1)C(=O)O	THF	-6	10.1016/j.dyepig.2013.07.002
CCCCC(Cn1c2ccc(cc2c2c1cccc2)c1ccc(c2c1nsn2)c1ccc(s1)C=C(C(=O)O)C#N)CC	THF	-1	10.1016/j.dyepig.2013.07.002
CCCCC(COc1ccc(cc1)n1c2ccc(cc2c2c1cccc2)c1ccc(c2c1nsn2)c1ccc(s1)C=C(C(=O)O)C#N)CC	THF	1	10.1016/j.dyepig.2013.07.002
N#CC(=Cc1ccc(s1)c1ccc(c2c1nsn2)c1ccc2c(c1)n(c1ccccc1)c1c2cccc1)C(=O)O	THF	-3	10.1016/j.dyepig.2013.07.002
CCCCC(COc1ccc(cc1)n1c2ccccc2c2c1cc(cc2)c1ccc(c2c1nsn2)c1ccc(s1)C=C(C(=O)O)C#N)CC	THF	1	10.1016/j.dyepig.2013.07.002
CCCCCCc1cc(sc1c1ccc(cc1)N(c1ccc2c(c1)c1ccccc1n2c1ccccc1)c1ccc2c(c1)c1ccccc1n2c1ccccc1)/C=C(\C(=O)O)/C#N	THF	-53	10.5012/bkcs.2013.34.5.1533
CCCCCCc1cc(sc1c1ccc(cc1)N(c1ccc2c(c1)c1ccccc1n2c1ccccc1)c1ccc2c(c1)c1ccccc1n2c1ccccc1)c1sc(cc1CCCCCC)/C=C(\C(=O)O)/C#N	THF	-61	10.5012/bkcs.2013.34.5.1533
N#CC(=Cc1ccc2c(c1)c1cc(ccc1n2CC)c1ccc(cc1)C=C(c1ccc(cc1)N(C)C)c1ccc(cc1)N(C)C)C(=O)O	chloroform	-26	10.1016/j.renene.2011.03.003
N#CC(=Cc1ccc2c(c1)c1cc(ccc1n2CC)c1ccc(c	chloroform	-25	10.1016/

SMILES	SOLVENT	SHIFT (nm)	DOI
c1)C=C(c1ccc(cc1)N(C)C)c1ccc(cc1)N(C)C)C(=O)O			j.renene.2011.03.003
N#C/C(=C/c1ccc2c(c1)c1ccc(cc1n2CC)c1ccc(s1)c1ccc(cc1)C=C(c1ccc(cc1)N(C)C)c1ccc(cc1)N(C)C)/C(=O)O	chloroform	-22	10.1016/j.renene.2011.03.003
N#C/C(=C/c1ccc2c(c1)c1ccc(cc1n2CC)c1ccc(s1)c1ccc(s1)c1ccc(cc1)C=C(c1ccc(cc1)N(C)C)c1ccc(cc1)N(C)C)/C(=O)O	chloroform	35	10.1016/j.renene.2011.03.003
N#C/C(=C\c1ccc(s1)c1ccc(cc1)n1c2ccccc2c2c1cccc2)/C(=O)O	dichloromethane	-4	10.1016/j.dyepig.2013.09.025
N#C/C(=C\c1ccc(s1)c1ccc(s1)n1c2ccccc2c2c1cccc2)/C(=O)O	dichloromethane	5	10.1016/j.dyepig.2013.09.025
N#C/C(=C\c1ccc(s1)c1ccc(s1)c1ccc(s1)n1c2ccccc2c2c1cccc2)/C(=O)O	dichloromethane	37	10.1016/j.dyepig.2013.09.025
N#C/C(=C\c1ccc(s1)c1ccc(s1)c1ccc(s1)n1c2ccc(cc2c2c1ccc(c2)n1c2ccccc2c2c1cccc2)n1c2ccccc2c2c1cccc2)/C(=O)O	dichloromethane	10	10.1016/j.dyepig.2013.09.025
CCCCCCCCn1c2ccc(cc2c2c1c(c1ccc(cc1)CC)c1c(c2c2ccc(cc2)CC)n(c2c1cccc2)CCCCCCCC)c1ccc(s1)/C=C(/C(=O)O)\C#N	dichloromethane	56	10.1016/j.jpowsour.2016.04.043
CCCCCCCCn1c2ccc(cc2c2c1c(c1ccc(cc1)N(CC)CC)c1c(c2c2ccc(cc2)N(CC)CC)n(c2c1cccc2)CCCCCCCC)c1ccc(s1)/C=C(/C(=O)O)\C#N	dichloromethane	80	10.1016/j.jpowsour.2016.04.043
CCCCCCCCn1c2ccc(cc2c2c1c(c1ccc(cc1)OCC)c1c(c2c2ccc(cc2)OCC)n(c2c1cccc2)CCCCCCCC)c1ccc(s1)/C=C(/C(=O)O)\C#N	dichloromethane	85	10.1016/j.jpowsour.2016.04.043
CCCCCCCCOc1ccc(cc1)c1c2c3ccccc3n(c2c(c2c1n(CCCCCCCC)c1c2cc(cc1)c1ccc(s1)/C=C(/C(=O)O)\C#N)c1ccc(cc1)OCCCCCCCC)CCCCCCCC	dichloromethane	65	10.1016/j.jpowsour.2016.04.043
CCCCn1c2cc(ccc2c2c1cc(cc2)c1ccc(s1)/C=C(/C(=O)O)\C#N)N(c1ccc(cc1)OCCCC)c1ccc(cc1)OCCCC	dichloromethane	5	10.1002/ajoc.201402235
CCCCOc1ccc(cc1)N(c1ccc2c(c1)n(CCCC)c1c2ccc(c1)c1ccc(s1)c1ccc(s1)/C=C(/C(=O)O)\C#N)c1ccc(cc1)OCCCC	dichloromethane	-5	10.1002/ajoc.201402235
CCCCn1c2cc(ccc2c2c1cc(cc2)N(c1ccc(cc1)c1ccc2c(c1)C(CC)	dichloromethane	10	10.1002/ajoc.201402235

SMILES	SOLVENT	SHIFT (nm)	DOI
(CC)c1c2cccc1)c1ccc(cc1)c1ccc2c(c1)C(CC)(CC)c1c2cccc1)c1ccc(s1)/C=C(/C(=O)O)\C#N			
CCCCn1c2cc(ccc2c2c1cc(cc2)N(c1ccccc1)c1ccccc1)c1ccc(s1)c1ccc(s1)/C=C(/C(=O)O)\C#N	dichloromethane	-2	10.1002/ajoc.201402235
CCCCC(COc1c2c3sc(cc3sc2c2c(c1OCC(CCCC)CC)c1sc(cc1s2)/C=C(\C(=O)O)/C#N)c1ccc2c(c1)c1ccccc1n2CC(CCCC)CC)CC	THF	-26	10.1016/j.orgel.2016.12.044
CCCCC(COc1c2c3sc(cc3sc2c2c(c1OCC(CCCC)CC)c1sc(cc1s2)/C=C(\C(=O)O)/C#N)c1ccc(cc1)N(c1ccc(cc1)OCCCCCCCC)c1ccc(cc1)OCCCCCCCC)CC	THF	-32	10.1016/j.orgel.2016.12.044
CCCCC(COc1c2c3sc(cc3sc2c2c(c1OCC(CCCC)CC)c1sc(cc1s2)/C=C(\C(=O)O)/C#N)c1ccc(c2c1nsn2)c1ccc2c(c1)c1ccccc1n2CC(CCCC)CC)CC	THF	-6	10.1016/j.orgel.2016.12.044
CCCCC(COc1c2c3sc(cc3sc2c2c(c1OCC(CCCC)CC)c1sc(cc1s2)/C=C(\C(=O)O)/C#N)c1ccc(c2c1nc(c1csc(c1)C)c(n2)c1ccc(s1)C)c1ccc2c(c1)c1ccccc1n2CC(CCCC)CC)CC	THF	31	10.1016/j.orgel.2016.12.044
CCCCC(COc1c2c3sc(cc3sc2c2c(c1OCC(CCCC)CC)c1sc(cc1s2)/C=C(\C(=O)O)/C#N)c1ccc2c(c1)n(CC(CCCC)CC)c1c2cc(cc1)c1sc2c(c1)sc1c2c(OCC(CCCC)CC)c(OCC(CCCC)CC)c2c1sc1c2sc(c1)/C=C(\C(=O)O)/C#N)CC	THF	17	10.1016/j.orgel.2016.12.044
CCCCn1c2cc(ccc2c2c1cc(cc2)c1ccc(s1)C1=C2C=CC(=[N+]2[B-](n2c1ccc2c1ccc(s1)c1ccncc1)(F)F)c1ccc(s1)c1ccncc1)N(c1ccccc1)c1ccccc1	dioxane	23	10.1039/C3NJ00456B
CCCCCCn1c2ccc(cc2c2c1cccc2)c1ccc(cc1)C=C(C(=O)O)C#N	chloroform	-60	10.1039/C7CP05226J
CCCCCCn1c2ccc(cc2c2c1cccc2)c1ccc(c(c1)F)C=C(C(=O)O)C#N	chloroform	-24	10.1039/C7CP05226J
CCCCCCn1c2ccc(cc2c2c1cccc2)c1ccc(cc1F)C=C(C(=O)O)C#N	chloroform	-50	10.1039/C7CP05226J
CCCCCCn1c2ccc(cc2c2c1cccc2)c1ccc(c(c1F)F)C=C(C(=O)O)C#N	chloroform	-23	10.1039/C7CP05226J
CCCCn1c2cc(ccc2c2c1cc(cc2)/C=C(/C(=O)O)\C#N)n1c2ccccc2c2c1cccc2	dichloromethane	-73	10.1021/am404948w
CCCCn1c2cc(ccc2c2c1cc(cc2)n1c2ccccc2c2c1cccc2)c1ccc(s1)/C=C(/C(=O)O)\C#N	dichloromethane	-80	10.1021/am404948w

SMILES	SOLVENT	SHIFT (nm)	DOI
CCCCn1c2cc(ccc2c2c1cc(cc2)n1c2ccc(cc2c2c1ccc(c2)C(C)(C)C)C(C)(C)C)c1ccc(s1)/C=C(/C(=O)O)\C#N	dichloromethane	-77	10.1021/am404948w
CCCCn1c2cc(ccc2c2c1cc(cc2)n1c2ccccc2c2c1cccc2)c1ccc(s1)c1ccc(s1)/C=C(/C(=O)O)\C#N	dichloromethane	-74	10.1021/am404948w
CCCCn1c2cc(ccc2c2c1cc(cc2)n1c2ccc(cc2c2c1ccc(c2)C(C)(C)C)C(C)(C)C)c1ccc(s1)c1ccc(s1)/C=C(/C(=O)O)\C#N	dichloromethane	-72	10.1021/am404948w
CCCCn1c2cc(ccc2c2c1cc(cc2)/C=C(/C(=O)O)\C#N)n1c2ccccc2c2c1cccc2	toluene	-74	10.1021/am404948w
CCCCn1c2cc(ccc2c2c1cc(cc2)n1c2ccccc2c2c1cccc2)c1ccc(s1)/C=C(/C(=O)O)\C#N	toluene	-86	10.1021/am404948w
CCCCn1c2cc(ccc2c2c1cc(cc2)n1c2ccc(cc2c2c1ccc(c2)C(C)(C)C)C(C)(C)C)c1ccc(s1)/C=C(/C(=O)O)\C#N	toluene	-81	10.1021/am404948w
CCCCn1c2cc(ccc2c2c1cc(cc2)n1c2ccccc2c2c1cccc2)c1ccc(s1)c1ccc(s1)/C=C(/C(=O)O)\C#N	toluene	-84	10.1021/am404948w
CCCCn1c2cc(ccc2c2c1cc(cc2)n1c2ccc(cc2c2c1ccc(c2)C(C)(C)C)C(C)(C)C)c1ccc(s1)c1ccc(s1)/C=C(/C(=O)O)\C#N	toluene	-82	10.1021/am404948w
CCCCn1c2cc(ccc2c2c1cc(cc2)/C=C(/C(=O)O)\C#N)n1c2ccccc2c2c1cccc2	chloroform	-59	10.1021/am404948w
CCCCn1c2cc(ccc2c2c1cc(cc2)n1c2ccccc2c2c1cccc2)c1ccc(s1)/C=C(/C(=O)O)\C#N	chloroform	-78	10.1021/am404948w
CCCCn1c2cc(ccc2c2c1cc(cc2)n1c2ccc(cc2c2c1ccc(c2)C(C)(C)C)C(C)(C)C)c1ccc(s1)/C=C(/C(=O)O)\C#N	chloroform	-71	10.1021/am404948w
CCCCn1c2cc(ccc2c2c1cc(cc2)n1c2ccccc2c2c1cccc2)c1ccc(s1)c1ccc(s1)/C=C(/C(=O)O)\C#N	chloroform	-70	10.1021/am404948w
CCCCn1c2cc(ccc2c2c1cc(cc2)n1c2ccc(cc2c2c1ccc(c2)C(C)(C)C)C(C)(C)C)c1ccc(s1)c1ccc(s1)/C=C(/C(=O)O)\C#N	chloroform	-69	10.1021/am404948w
CCCCn1c2cc(ccc2c2c1cc(cc2)/C=C(/C(=O)O)\C#N)n1c2ccccc2c2c1cccc2	THF	-87	10.1021/am404948w
CCCCn1c2cc(ccc2c2c1cc(cc2)n1c2ccccc2c2c1cccc2)c1ccc(s1)/C=C(/C(=O)O)\C#N	THF	-96	10.1021/am404948w
CCCCn1c2cc(ccc2c2c1cc(cc2)n1c2ccc(cc2c2c1ccc(c2)C(C)(C)C)C(C)(C)C)c1ccc(s1)/C=C(/C(=O)O)\C#N	THF	-92	10.1021/am404948w
CCCCn1c2cc(ccc2c2c1cc(cc2)n1c2ccccc2c2c1cccc2)c1ccc(s1)c1ccc(s1)/C=C(/C(=O)O)\	THF	-92	10.1021/am404948w

SMILES	SOLVENT	SHIFT (nm)	DOI
C#N			
CCCCn1c2cc(ccc2c2c1cc(cc2)n1c2ccc(cc2c2c1ccc(c2)C(C)(C)C)C(C)(C)C)c1ccc(s1)c1ccc(s1)/C=C(/C(=O)O)\C#N	THF	-93	10.1021/am404948w
CCCCn1c2cc(ccc2c2c1cc(cc2)/C=C(/C(=O)O)\C#N)n1c2ccccc2c2c1cccc2	DMF	-116	10.1021/am404948w
CCCCn1c2cc(ccc2c2c1cc(cc2)n1c2ccccc2c2c1cccc2)c1ccc(s1)/C=C(/C(=O)O)\C#N	DMF	-127	10.1021/am404948w
CCCCn1c2cc(ccc2c2c1cc(cc2)n1c2ccc(cc2c2c1ccc(c2)C(C)(C)C)C(C)(C)C)c1ccc(s1)/C=C(/C(=O)O)\C#N	DMF	-124	10.1021/am404948w
CCCCn1c2cc(ccc2c2c1cc(cc2)n1c2ccccc2c2c1cccc2)c1ccc(s1)c1ccc(s1)/C=C(/C(=O)O)\C#N	DMF	-124	10.1021/am404948w
CCCCn1c2cc(ccc2c2c1cc(cc2)n1c2ccc(cc2c2c1ccc(c2)C(C)(C)C)C(C)(C)C)c1ccc(s1)c1ccc(s1)/C=C(/C(=O)O)\C#N	DMF	-124	10.1021/am404948w
CCCCn1c2cc(ccc2c2c1cc(cc2)/C=C(/C(=O)O)\C#N)n1c2ccccc2c2c1cccc2	acetonitrile	-91	10.1021/am404948w
CCCCn1c2cc(ccc2c2c1cc(cc2)n1c2ccccc2c2c1cccc2)c1ccc(s1)/C=C(/C(=O)O)\C#N	acetonitrile	-98	10.1021/am404948w
CCCCn1c2cc(ccc2c2c1cc(cc2)n1c2ccc(cc2c2c1ccc(c2)C(C)(C)C)C(C)(C)C)c1ccc(s1)/C=C(/C(=O)O)\C#N	acetonitrile	-98	10.1021/am404948w
CCCCn1c2cc(ccc2c2c1cc(cc2)n1c2ccccc2c2c1cccc2)c1ccc(s1)c1ccc(s1)/C=C(/C(=O)O)\C#N	acetonitrile	-111	10.1021/am404948w
CCCCn1c2cc(ccc2c2c1cc(cc2)n1c2ccc(cc2c2c1ccc(c2)C(C)(C)C)C(C)(C)C)c1ccc(s1)c1ccc(s1)/C=C(/C(=O)O)\C#N	acetonitrile	-93	10.1021/am404948w
CCCCn1c2cc(ccc2c2c1cc(cc2)/C=C(/C(=O)O)\C#N)n1c2ccccc2c2c1cccc2	ethanol	-119	10.1021/am404948w
CCCCn1c2cc(ccc2c2c1cc(cc2)n1c2ccccc2c2c1cccc2)c1ccc(s1)/C=C(/C(=O)O)\C#N	ethanol	-120	10.1021/am404948w
CCCCn1c2cc(ccc2c2c1cc(cc2)n1c2ccc(cc2c2c1ccc(c2)C(C)(C)C)C(C)(C)C)c1ccc(s1)/C=C(/C(=O)O)\C#N	ethanol	-116	10.1021/am404948w
CCCCn1c2cc(ccc2c2c1cc(cc2)n1c2ccccc2c2c1cccc2)c1ccc(s1)c1ccc(s1)/C=C(/C(=O)O)\C#N	ethanol	-118	10.1021/am404948w
CCCCn1c2cc(ccc2c2c1cc(cc2)n1c2ccc(cc2c2c1ccc(c2)C(C)(C)C)C(C)(C)C)c1ccc(s1)c1ccc(s1)/C=C(/C(=O)O)\C#N	ethanol	-117	10.1021/am404948w

SMILES	SOLVENT	SHIFT (nm)	DOI
N#C/C(=C/C=C/c1cc2cc3c4c(c2oc1=O)C(C)(C)CCN4CCC3(C)C)/C(=O)O	ethanol	6	10.1021/jp026963x
N#C/C(=C/C=C/C=C/c1cc2cc3c4c(c2oc1=O)C(C)(C)CCN4CCC3(C)C)/C(=O)O	ethanol	43	10.1021/la0357615
N#C/C(=C/c1ccc(s1)c1ccc(s1)c1cc2cc3c4c(c2oc1=O)C(C)(C)CCN4CCC3(C)C)/C(=O)O	THF	36	10.1016/j.dyepig.2011.01.009
N#C/C(=C\c1sc(c2c1OCCO2)c1ccc(s1)c1cc2cc3c4c(c2oc1=O)C(C)(C)CCN4CCC3(C)C)/C(=O)O	THF	12	10.1016/j.dyepig.2011.01.009
N#C/C(=C\c1ccc(cc1)c1sc(c2c1OCCO2)c1ccc(s1)c1cc2cc3c4c(c2oc1=O)C(C)(C)CCN4CCC3(C)C)/C(=O)O	THF	9	10.1016/j.dyepig.2011.01.009
N#C/C(=C\c1ccc(s1)c1sc(c2c1OCCO2)c1ccc(s1)c1cc2cc3c4c(c2oc1=O)C(C)(C)CCN4CCC3(C)C)/C(=O)O	THF	27	10.1016/j.dyepig.2011.01.009
N#CC(=Cc1ccc(s1)c1ccc(s1)c1c(c2ccccc2)n(c2c1cccc2)c1ccc2c(c1)C(C)(C)c1c2cccc1)C(=O)O	ethanol	-12	10.1016/j.tet.2008.08.045
N#CC(=Cc1ccc(s1)c1ccc(s1)c1ccc2c(c1)cc(n2c1ccc2c(c1)C(C)(C)c1c2cccc1)c1ccccc1)C(=O)O	ethanol	-16	10.1016/j.tet.2008.08.045
N#C/C(=C\c1ccc(s1)c1ccc2c(c1)c1ccccc1n1c2cc2c1cc(cc2)C)/C(=O)O	THF	8	10.1016/j.tet.2009.04.077
N#C/C(=C\c1ccc(s1)c1ccc(s1)c1ccc2c(c1)c1ccccc1n1c2cc2c1cc(cc2)C)/C(=O)O	THF	-40	10.1016/j.tet.2009.04.077
CCCC1(CCC)c2ccc(cc2c2c1cccc2)N1C2CCCC2c2c1ccc(c2)c1ccc(c2c1nsn2)c1ccc(s1)/C=C(\C(=O)O)/C#N	dichloromethane	28	10.1016/j.dyepig.2013.10.016
N#C/C(=C/c1ccc(s1)c1ccc(c2c1nsn2)c1ccc2c(c1)C1CCCC1N2c1ccc(cc1)C(C)(C)C)/C(=O)O	dichloromethane	34	10.1016/j.dyepig.2013.10.016
CCCC1(CCC)c2ccc(cc2c2c1cccc2)N1C2CCCC2c2c1ccc(c2)c1ccc(s1)/C=C(\C(=O)O)/C#N	dichloromethane	53	10.1016/j.dyepig.2013.10.016
COc1ccc(cc1)N1c2ccc(cc2C2C1CCC2)/C=C/1\SC(=S)N(C1=O)CC(=O)O	ethanol	-117	10.1016/j.jphotochem.2003.12.018
COc1ccc(cc1)N1C2CCCC2c2c1ccc(c2)/C=C(/C(=O)O)\C#N	ethanol	-25	10.1016/j.jphotochem.2003.12.018
N#C/C(=C/1\C=C(/C=C/	acetonitrile	24	10.1039/b820964b

SMILES	SOLVENT	SHIFT (nm)	DOI
c2ccc(cc2)N(C)C)CC(C1)(C)C)/C(=O)O			
N#C/C(=C/1\C=C(/C=C/c2ccc(cc2)N(c2ccccc2)c2ccccc2)CC(C1)(C)C)/C(=O)O	acetonitrile	0	10.1039/b820964b
N#C/C(=C/1\C=C(/C=C/c2ccc3c(c2)C2CCCC2N3c2ccc(cc2)C)CC(C1)(C)C)/C(=O)O	acetonitrile	57	10.1039/b820964b
CCCCCCn1cc(c2c1ccc(c2)OC)/C=C(\c1ccc(s1)C=C(C(=O)O)C#N)/C#N	chloroform	68	10.1016/j.electacta.2015.07.079
CCCCCCn1cc(c2c1ccc(c2)OC)/C=C(\c1ccc(s1)/C=C/1\SC(=S)N(C1=O)CC(=O)O)/C#N	chloroform	82	10.1016/j.electacta.2015.07.079
CCCCCCn1cc(c2c1ccc(c2)OC)/C=C(\c1ccc(s1)/C=N/c1ccc(cc1)C(=O)O)/C#N	chloroform	32	10.1016/j.electacta.2015.07.079
CCCCCCN1c2ccc(cc2Sc2c1ccc(c2)N(c1ccccc1)c1ccccc1)c1ccc(s1)/C=C(\C(=O)O)/C#N	THF	-19	10.1002/cssc.201402160
CCCCCCN1c2ccc(cc2S(=O)(=O)c2c1ccc(c2)c1ccc(s1)/C=C(\C(=O)O)/C#N)c1ccc(cc1)N(c1ccccc1)c1ccccc1	THF	-32	10.1002/cssc.201402160
CCCCCCN1c2ccc(cc2S(=O)(=O)c2c1ccc(c2)c1ccc(s1)/C=C(\C(=O)O)/C#N)c1ccc(s1)c1ccc(cc1)N(c1ccccc1)c1ccccc1	THF	-39	10.1002/cssc.201402160
CCCCCCN1c2ccc(cc2S(=O)(=O)c2c1ccc(c2)c1ccc(s1)/C=C(\C(=O)O)/C#N)c1ccc(cc1)N(c1ccc(cc1)OCCCCCC)c1ccc(cc1)OCCCCCC	THF	-9	10.1002/cssc.201402160
CCCCCCN1c2ccc(cc2S(=O)(=O)c2c1ccc(c2)c1csc2c1sc(c2)[C@@H](C(=O)O)C#N)c1ccc(cc1)N(c1ccc(cc1)OCCCCCC)c1ccc(cc1)OCCCCCC	THF	4	10.1002/cssc.201402160
CCCCCCN1c2ccc(cc2Sc2c1ccc(c2)/C=C(/C(=O)O)\C#N)c1ccc2c(c1)C(=[N+]1C2Cc2n([B-]1(F)F)c(c1c2cccc1)c1ccccc1OC)c1ccccc1OC	THF	-22	10.1039/C7NJ01735A
CCCCC(Cn1c2c3n(CC(CCCC)CC)c4c(c3sc2c2c1sc(c2)/C=C(\C(=O)O)/C#N)sc(c4)/C=C(/C(=O)O)\C#N)CC	THF	5	10.1016/j.orgel.2015.01.005
CCCCC(Cn1c2c3sc4c(c3sc2c2c1cc(s2)/C=C(\C(=O)O)/C#N)n(c1c4sc(c1)/C=C(/C(=O)O)\C#N)CC(CCCC)CC)CC	THF	-26	10.1016/j.orgel.2015.01.005
CCCCCCCCC(n1c2c3sc4c(c3sc2c2c1cc(s2)/C=C(\C(=O)O)/C#N)n(c1c4sc(c1)/C=C(/C(=O)O)\	THF	-4	10.1016/j.orgel.2015.01.005

SMILES	SOLVENT	SHIFT (nm)	DOI
C#N)C(CCCCCCCC)CCCCCCCC)CCCCCCCC			
CCN(c1ccc(cc1)c1nc2c([nH]1)cc(cc2)C(=O)O)CC	DMF	-48	10.1016/j.dyepig.2014.02.010
OC(=O)c1ccc2c(c1)[nH]c(n2)c1ccc(cc1)N(c1ccccc1)c1ccccc1	DMF	-38	10.1016/j.dyepig.2014.02.010
CCN(c1ccc(cc1)C=C(c1nc2c([nH]1)cc(cc2)C(=O)O)C#N)CC	DMF	-9	10.1016/j.dyepig.2014.02.010
N#CC(=Cc1ccc(cc1)N(c1ccccc1)c1ccccc1)c1nc2c([nH]1)cc(cc2)C(=O)O	DMF	-17	10.1016/j.dyepig.2014.02.010
N#CC(=Cc1ccc(s1)c1cc(C(=O)O)c(cc1C(=O)O)c1ccc(s1)/C=C(/c1ccc(cc1)[N+](=O)[O-])\C#N)c1ccc(cc1)[N+](=O)[O-]	THF	15	10.1016/j.electacta.2011.04.011
N#C/C(=C\c1ccc(s1)c1ccc(s1)c1ccc(cc1)c1cc2CCCN3c2c(c1)CCC3)/C(=O)O	ethanol	32	10.1016/j.dyepig.2013.06.023
OC(=O)CN1C(=S)S/C(=C\c2ccc(s2)c2ccc(s2)c2ccc(cc2)c2cc3CCCN4c3c(c2)CCC4)/C1=O	ethanol	16	10.1016/j.dyepig.2013.06.023
CCCCCCSC1=C(SCCCCCC)SC(=c2c3ccccc3c(=C3SC(=C(S3)SCCCCCC)SCCCCCC)c3c2ccc(c3)c2ccc(s2)/C=C(/C(=O)O)\C#N)S1	dichloromethane	21	10.1039/c6qm00070c
CCCCCCSC1=C(SCCCCCC)SC(=c2c3cc(C#Cc4ccc(s4)/C=C(/C(=O)O)\C#N)ccc3c(=C3SC(=C(S3)SCCCCCC)SCCCCCC)c3c2cccc3)S1	dichloromethane	17	10.1039/c6qm00070c
CCCCCCCCOc1c2cc(c3ccc(s3)/C=C(/C(=O)O)\C#N)c3c4c2c2c5c1cccc5c(cc2c(c4ccc3)OCCCCCCCC)c1ccc(s1)/C=C(/C(=O)O)\C#N	THF	64	10.1039/c5ra21917e
CCCCCCCCOc1c2cccc3c2c2c4c1cc(c1ccc(s1)/C=C(/C(=O)O)\C#N)c1c4c(c(c2cc3c2ccc(cc2)N(c2ccc(cc2)C)c2ccc(cc2)C)OCCCCCCCC)ccc1	THF	61	10.1039/c5ra21917e
CCCCC(COc1ccc(cc1)c1c2cccc3c2c2c4c1cc(c1ccc(s1)/C=C(/C(=O)O)\C#N)c1c4c(c(c2cc3c2ccc(s2)/C=C(/C(=O)O)\C#N)c2ccc(cc2)OCC(CCCC)CC)ccc1)CC	THF	-7	10.1039/c5ra21917e
CCCCC(COc1ccc(cc1)c1c2cccc3c2c2c4c1cc(c1ccc(s1)/C=C(/C(=O)O)\C#N)c1c4c(c(c2cc3c2ccc(cc2)N(c2ccc(cc2)C)c2ccc(cc2)C)c2ccc(cc2)OCC(CCCC)CC)ccc1)CC	THF	4	10.1039/c5ra21917e
CCCCCCc1cc(sc1c1sc(c(c1)CCCCCC)c1scc(c1)CCCCCC)c1sc(cc1CCCCCC)c1ccc(s1)C=C(C(	dichloromethane	0	10.1039/c5ta09133k

SMILES	SOLVENT	SHIFT (nm)	DOI
=O)O)C#N			
CCCCCCc1cc(sc1c1sc(c(c1)CCCCCC)c1scc(c1)CCCCCC)c1sc(cc1CCCCCC)c1sc(cc1CCCCCC)c1ccc(s1)C=C(C(=O)O)C#N	dichloromethane	0	10.1039/c5ta09133k
CCO[Si](c1ccccc1)(c1ccc(cc1)N=Nc1ccc(cc1)N(C)C)OCC	acetonitrile	13	10.1007/s12633-013-9174-y
CCO[Si](c1ccccc1)(c1ccc(cc1)N=Nc1ccccc1)OCC	acetonitrile	34	10.1007/s12633-013-9174-y
O[Si](c1ccc(cc1)N=Nc1ccccc1)(c1ccc(cc1)N=Nc1ccccc1)O	acetonitrile	20	10.1002/aoc.1612
OC(=O)c1ccc(cc1)N=Nc1ccccc1	acetonitrile	19	10.1002/aoc.1612
CCCCCCCCOc1cc2c3cc(sc3c3c(c2cc1OCCCCCCCC)cc(s3)C=C(C(=O)O)C#N)c1ccc(cc1)N(c1ccc(cc1)OC)c1ccc(cc1)OC	THF	11	10.1039/c3cc44258f
CCCCCCCCOc1cc2c3cc(sc3c3c(c2cc1OCCCCCCCC)cc(s3)C=C(C(=O)O)C#N)c1sc(c2c1OCCO2)c1ccc(cc1)N(c1ccc(cc1)OC)c1ccc(cc1)OC	THF	1	10.1039/c3cc44258f
CCCCCCCCOc1cc2c3cc(sc3c3c(c2cc1OCCCCCCCC)cc(s3)CC(C(O)O)CN)c1ccc(c2c1nsn2)c1ccc(cc1)N(c1ccc(cc1)OC)c1ccc(cc1)OC	THF	0	10.1039/c3cc44258f
OC(=O)CN1C(=S)S/C(=C/c2ccc(cc2)N(c2ccccc2)c2ccccc2)/C1=O	methanol	-17	10.1021/jp067930a
OC(=O)CN1C(=S)S/C(=C/C=C/c2ccc(cc2)N(c2ccccc2)c2ccccc2)/C1=O	methanol	-15	10.1021/jp067930a
C=Cc1ccc(cc1)N(c1ccccc1)c1ccc(cc1)/C=C\1/SC(=S)N(C1=O)CC(=O)O	methanol	-6	10.1021/jp067930a
C=Cc1ccc(cc1)N(c1ccccc1)c1ccc(cc1)/C=C/C=C\1/SC(=S)N(C1=O)CC(=O)O	methanol	-27	10.1021/jp067930a
N#C/C(=C\c1sc(c2c1OCC(CO2)(C)C)c1sc(c2c1OCC(CO2)(C)C)c1ccc(cc1)N(c1ccccc1)c1ccccc1)/C(=O)O	THF	-31	10.1021/ol902973r
CCCC1(CCC)COc2c(OC1)c(sc2c1ccc(cc1)N(c1ccccc1)c1ccccc1)c1sc(c2c1OCC(CO2)(CCC)CCC)/C=C(/C(=O)O)\C#N	THF	-27	10.1021/ol902973r
CCCCCCC1(CCCCCC)COc2c(OC1)c(sc2c1sc(c2c1OCC(CO2)(CCCCCC)CCCCCC)/C=C(/C(=O)O)\C#N)c1ccc(cc1)N(c1ccccc1)c1ccccc1	THF	2	10.1021/ol902973r
N#CC(=Cc1ccc(s1)c1ccc(s1)c1ccc(cc1)N(c1ccccc1)c1ccccc1)C(=O)O	THF	-28	10.1021/ol902973r
CCCCCCOc1ccc(cc1)N(c1ccc(cc1)OCCCCCC)c1ccc(cc1)c1ccc(s1)c1scc(c1)c1nc2cccc(c2n	toluene	9	10.1039/c3ta11398a

SMILES	SOLVENT	SHIFT (nm)	DOI
c1c1ccc(s1)/C=C(/C(=O)O)\ C#N)c1ccc(s1)c1ccc(cc1)N(c1ccc(cc1)OCCC CCC)c1ccc(cc1)OCCCCCC			
CCCCCCOc1ccc(cc1)N(c1ccc(cc1)OCCCCCC) c1ccc(cc1)c1ccc(s1)c1ccc(c2c1nc(c1ccc(s1)/ C=C(\C(=O)O)/C#N)c(n2)c1ccc(s1)/C=C(\ C(=O)O)/ C#N)c1ccc(s1)c1ccc(cc1)N(c1ccc(cc1)OCCC CCC)c1ccc(cc1)OCCCCCC	toluene	3	10.1039/c3ta11398a
CCCCCCCCOc1ccc(cc1)c1ccc(cc1)N(c1ccc(cc 1)c1ccc(c2c1nsn2)c1ccc(cc1)C(=O)O)c1ccc(cc1)c1ccc(cc1)OCCCCCCCC	THF	8	10.1039/c6cp05173a
CCCCCCCCOc1ccc(cc1)c1ccc(cc1)N(c1ccc(cc 1)c1ccc(c2c1nsn2)C#Cc1ccc(cc1)C(=O)O)c1 ccc(cc1)c1ccc(cc1)OCCCCCCCC	THF	5	10.1039/c6cp05173a
CCCCCCCCOc1ccc(cc1)c1ccc(cc1)N(c1ccc(cc 1)c1ccc(cc1)OCCCCCCCC)c1ccc(cc1)c1ccc(c 2c1nsn2)C#Cc1sc2c(c1)c(OCCCCCCCC)c1c(c 2OCCCCCCCC)cc(s1)c1ccc(cc1)C(=O)O	THF	-7	10.1039/c6cp05173a
CCCCCCCCOc1ccc(cc1)c1ccc(cc1)N(c1ccc(cc 1)c1ccc(cc1)OCCCCCCCC)c1ccc(cc1)c1ccc(c 2c1nsn2)C#Cc1sc2c(c1)c(OCCCCCCCC)c1c(c 2OCCCCCCCC)cc(s1)c1ccc(s1)C(=O)O	THF	-7	10.1039/c6cp05173a
CCCCCCc1ccc(s1)c1ccc(s1)c1ccc(cc1)N(c1cc c(cc1)c1ccc(s1)c1ccc(s1)CCCCCC)c1ccc(cc1) /C=C(\C(=O)O)/C#N	chloroform	68	10.1016/ j.electacta.2015.04.071
CCCCCCc1ccc(s1)c1ccc(s1)c1ccc(cc1)N(c1cc c(cc1)c1ccc(s1)c1ccc(s1)CCCCCC)c1ccc(cc1) /C=C\1/SC(=S)N(C1=O)CC(=O)O	chloroform	94	10.1016/ j.electacta.2015.04.071
N#C/C(=C/c1ccc(s1)/C=C/ c1ccc(cc1)N(c1ccc(cc1)c1ccc(cc1)C(C) (C)C)c1ccc(cc1)c1ccc(cc1)C(C)(C)C)/C(=O)O	ethanol	-4	10.1016/ j.dyepig.2012.03.027
N#C/C(=C/c1ccc(s1)c1ccc(s1)/C=C/ c1ccc(cc1)N(c1ccc(cc1)c1ccc(cc1)C(C) (C)C)c1ccc(cc1)c1ccc(cc1)C(C)(C)C)/C(=O)O	ethanol	-27	10.1016/ j.dyepig.2012.03.027
N#C/C(=C/c1sc(c2c1OCCO2)/C=C/ c1ccc(cc1)N(c1ccc(cc1)c1ccc(cc1)C(C) (C)C)c1ccc(cc1)c1ccc(cc1)C(C)(C)C)/C(=O)O	ethanol	-6	10.1016/ j.dyepig.2012.03.027
N#C/C(=C/c1ccc(o1)/C=C/ c1ccc(cc1)N(c1ccc(cc1)c1ccc(cc1)C(C) (C)C)c1ccc(cc1)c1ccc(cc1)C(C)(C)C)/C(=O)O	ethanol	-19	10.1016/ j.dyepig.2012.03.027
OC(=O)CN1C(=S)S/C(=C\ c2ccc(cc2)N(c2ccc(cc2)/C=N/ N(c2ccccc2)c2ccccc2)c2ccccc2)/C1=O	chloroform	32	10.1016/ j.dyepig.2014.11.012

SMILES	SOLVENT	SHIFT (nm)	DOI
Cc1ccc(cc1)N(c1ccc(cc1)/C=C/1\SC(=S)N(C1=O)CC(=O)O)c1ccc(cc1)/C=N/N(c1ccccc1)c1ccccc1	chloroform	27	10.1016/j.dyepig.2014.11.012
COc1ccc(cc1)N(c1ccc(cc1)/C=C/1\SC(=S)N(C1=O)CC(=O)O)c1ccc(cc1)/C=N/N(c1ccccc1)c1ccccc1	chloroform	32	10.1016/j.dyepig.2014.11.012
CCCCCCCCCN(c1ccccc1)/N=C/c1ccc(cc1)N(c1ccc(cc1)/C=C/1\SC(=S)N(C1=O)CC(=O)O)c1ccc(cc1)C	chloroform	18	10.1016/j.dyepig.2014.11.012
CCCCCCc1ccc(cc1)N(c1ccc(cc1)/C=C/1\SC(=S)N(C1=O)CC(=O)O)c1ccc(cc1)/C=N/N(c1ccccc1)CCCCCCCCC	chloroform	23	10.1016/j.dyepig.2014.11.012
COc1ccc(cc1)N(c1ccc(cc1)OC)c1ccc(cc1)/C=C/c1nc2cc(ccc2nc1/C=C/c1ccc(cc1)N(c1ccc(cc1)OC)c1ccc(cc1)OC)C(=O)O	chloroform	22	10.1016/j.dyepig.2014.11.012
COc1cccc(c1)c1ccc(cc1)N(c1ccc(cc1)/C=C(\C(=O)O)/[N+]#[C-])c1ccc(cc1)c1nc2c([nH]1)c(ccc2c1cccc(c1)OC)c1cccc(c1)OC	toluene	0	10.1016/j.saa.2015.04.074
COc1cccc(c1)c1ccc(c2c1[nH]c(n2)c1ccc(cc1)N(c1ccc(cc1)c1cccc(c1)N(C)C)c1ccc(cc1)/C=C(\C(=O)O)/[N+]#[C-])c1cccc(c1)OC	toluene	0	10.1016/j.saa.2015.04.074
COc1cccc(c1)c1ccc(c2c1[nH]c(n2)c1ccc(cc1)N(c1ccc(cc1)/C=C(\C(=O)O)/[N+]#[C-])c1ccc(cc1)Br)c1cccc(c1)OC	toluene	12	10.1016/j.saa.2015.04.074
COc1cccc(c1)c1ccc(cc1)N(c1ccc(cc1)/C=C(\C(=O)O)/[N+]#[C-])c1ccc(cc1)c1nc2c([nH]1)c(ccc2c1cccc(c1)OC)c1cccc(c1)OC	chloroform	0	10.1016/j.saa.2015.04.074
COc1cccc(c1)c1ccc(c2c1[nH]c(n2)c1ccc(cc1)N(c1ccc(cc1)c1cccc(c1)N(C)C)c1ccc(cc1)/C=C(\C(=O)O)/[N+]#[C-])c1cccc(c1)OC	chloroform	0	10.1016/j.saa.2015.04.074
COc1cccc(c1)c1ccc(c2c1[nH]c(n2)c1ccc(cc1)N(c1ccc(cc1)/C=C(\C(=O)O)/[N+]#[C-])c1ccc(cc1)Br)c1cccc(c1)OC	chloroform	2	10.1016/j.saa.2015.04.074
CCCCCCOc1ccc(cc1)N(c1ccc2c(c1)C(CCCCCC)(CCCCCC)c1c2c2c(c3c1c1ccccc1C3(CCCCCC)CCCCCC)c1c(C2(CCCCCC)CCCCCC)cccc1)c1ccc(cc1)c1cc2c(s1)c1c(n2c2ccc(cc2)OCCCCCC)c(cs1)C=C(C(=O)O)C#N	dichloromethane	59	10.1002/cssc.201301155
CCCCCCOc1ccc(cc1)N(c1ccc2c(c1)C(CCCCCC)	dichloromethane	47	10.1002/cssc.201301155

SMILES	SOLVENT	SHIFT (nm)	DOI
(CCCCCC)c1c2c2c(c3c1c1ccccc1C3(CCCCCC)CCCCCC)c1c(C2(CCCCCC)CCCCCC)cccc1)c1ccc(cc1)c1cc2c(s1)c1c(n2CCCCCC)c(cs1)C=C(C(=O)O)C#N			
CCCCCCOc1ccc(cc1)N(c1ccc(cc1)OCCCCCC)c1ccc(cc1)c1cc2c(s1)c1c(n2CCCCCC)c(cs1)C=C(C(=O)O)C#N	dichloromethane	74	10.1002/cssc.201301155
OC(=O)CN1C(=S)SC(=Cc2ccc(cc2)N(c2ccc(cc2)C=C(c2ccccc2)c2ccccc2)c2ccccc2)C1=O	methanol	-8	10.1016/j.jpowsour.2008.11.028
N#CC(=Cc1cc2c(s1)c1c(C2=O)cc(s1)c1ccc(cc1)N(c1ccc(cc1)C)c1ccc(cc1)C)C(=O)O	DMF	-12	10.1016/j.dyepig.2012.03.002
N#CC(=Cc1cc2c(s1)c1c(C2=O)cc(s1)c1ccc(s1)c1ccc(cc1)N(c1ccc(cc1)C)c1ccc(cc1)C)C(=O)O	DMF	7	10.1016/j.dyepig.2012.03.002
N#CC(=Cc1cc2c(s1)c1c(C2=O)cc(s1)c1sc(c2c1OCCO2)c1ccc(cc1)N(c1ccc(cc1)C)c1ccc(cc1)C)C(=O)O	DMF	10	10.1016/j.dyepig.2012.03.002
CCC(CCCOc1ccccc1N(c1ccccc1OCCCC(CC)CC)c1ccc(cc1)c1ccc2c(c1)C=C1C2=CC=C(C1=O)c1ccc(s1)C=C(C(=O)O)C#N)CC	DMF	4	10.1039/c2jm33105e
N#CC(=Cc1ccc(s1)C1=CC=C2C(=Cc3c2ccc(c3)c2ccc(cc2)N(c2ccc(cc2)C)c2ccc(cc2)C)C1=O)C(=O)O	DMF	8	10.1039/c2jm33105e
N#C/C(=C/c1ccc(s1)c1ccc2c(c1)C(CC)(CC)c1c2ccc(c1)c1ccc(cc1)N(c1ccccc1)c1ccccc1)/C(=O)O	chloroform	7	10.1016/j.orgel.2010.10.016
CCCCCCc1cc(sc1/C=C(\C(=O)O)/C#N)c1ccc2c(c1)n(CC)c1c2ccc(c1)c1ccc(cc1)N(c1ccccc1)c1ccccc1	chloroform	-11	10.1016/j.orgel.2010.10.016
CCCCCCc1cc(sc1/C=C(\C(=O)O)/C#N)c1ccc2c(c1)C1(c3c2ccc(c3)c2ccc(cc2)N(c2ccccc2)c2ccccc2)c2ccccc2c2c1cccc2	chloroform	-6	10.1016/j.orgel.2010.10.016
N#C/C(=C\c1ccc(cc1)[N+](=O)[O-])/c1ccc(cc1)N(c1ccc(cc1)/C=C\C(=O)O)c1ccc(cc1)/C=C\C(=O)O	THF	-24	10.1021/jp210971u
N#C/C(=C\c1ccc(cc1)N(c1ccc(cc1)/C=C(/c1ccc(cc1)[N+](=O)[O-])\C#N)c1ccc(cc1)/C=C/C(=O)O)/c1ccc(cc1)[N+](=O)[O-]	THF	1	10.1021/jp210971u
N#C/C(=C\c1cc(n(c1c1ccc(s1)c1ccc(cc1)N(c1ccccc1)c1ccccc1)c1c(cccc1C(C)C)C(C)C)c1ccc(s1)c1ccc(cc1)N(c1ccccc1)c1ccccc1)/C(=O)O	THF	-45	10.1016/j.tet.2012.04.104
N#CC(=Cc1cc(c(s1)c1ccc(cc1)N(c1ccccc1)c1ccccc1)C)C(=O)O	chloroform	6	10.1016/j.dyepig.2010.03.029

SMILES	SOLVENT	SHIFT (nm)	DOI
N#CC(=Cc1cc(c(s1)C=Cc1ccc(cc1)N(c1ccccc1)c1ccccc1)C)C(=O)O	chloroform	32	10.1016/j.dyepig.2010.03.029
CCCCCCc1cc(sc1C=Cc1ccc(cc1)N(c1ccc(cc1)C)c1ccc(cc1)C)C=C(C(=O)O)C#N	chloroform	31	10.1016/j.dyepig.2010.03.029
N#C/C(=C\c1ccc(cc1)N(c1ccccc1)c1ccccc1)/C(=O)O	methanol	-28	10.1016/j.orgel.2013.05.011
N#C/C(=C\c1ccc(cc1)N(c1ccc(cc1)C=C1SC(=C(S1)SC)SC)c1ccc(cc1)C=C1SC(=C(S1)SC)SC)/C(=O)O	methanol	33	10.1016/j.orgel.2013.05.011
CCCCCCOc1ccc(cc1)N(c1ccc(cc1)OCCCCCC)c1ccc(cc1)c1cc2c(s1)c1c(n2CCCCCC)c2c(s1)c1c(n2CCCCCC)cc(s1)/C=C(/C(=O)O)\C#N	dichloromethane	87	10.1039/c4ta06705c
CCCCCCOc1ccc(cc1)n1c2cc(sc2c2c1c1c(s2)c2c(n1c1ccc(cc1)OCCCCCC)cc(s2)/C=C(/C(=O)O)\C#N)c1ccc(cc1)N(c1ccc(cc1)OCCCCCC)c1ccc(cc1)OCCCCCC	dichloromethane	70	10.1039/c4ta06705c
CCCCCCOc1ccc(cc1)N(c1ccc(cc1)OCCCCCC)c1ccc(cc1)c1cc2c(s1)c1c(n2CCCCCC)cc(s1)/C=C(\C(=O)O)/C#N	dichloromethane	65	10.1039/c4ta06705c
N#C/C(=C/c1ccc(cc1)N(c1ccc(cc1)/C=C(/C(=O)O)\C#N)c1ccccc1)/C(=O)O	chloroform	20	10.1016/j.dyepig.2014.01.012
OC(=O)CN1C(=S)S/C(=C\c2ccc(cc2)N(c2ccc(cc2)/C=C\2/SC(=S)N(C2=O)CC(=O)O)c2ccccc2)/C1=O	chloroform	6	10.1016/j.dyepig.2014.01.012
CCCCCCCCN1C(=S)S/C(=c\2/s/c(=C/c3ccc(cc3)N(c3ccc(cc3)/C=c/3\s/c(=C\4/SC(=S)N(C4=O)CCCCCCCC)/n(c3=O)CC(=O)O)c3ccccc3)/c(=O)n2CC(=O)O)/C1=O	chloroform	0	10.1016/j.dyepig.2014.01.012
N#C/C(=C\c1cc2c(s1)cc(s2)c1ccc(cc1)N(c1ccccc1)c1ccccc1)/C(=O)O	chloroform	73	10.1021/jp808275z
N#C/C(=C\c1cc2c(s1)cc(s2)c1ccc(cc1)N(c1ccc(cc1)OC)c1ccc(cc1)OC)/C(=O)O	chloroform	77	10.1021/jp808275z
N#C/C(=C\c1cc2c(s1)cc(s2)c1cc(OC)c(cc1OC)N(c1ccc(cc1)OC)c1ccc(cc1)OC)/C(=O)O	chloroform	77	10.1021/jp808275z
CCCCCCOc1ccc(cc1)N(c1ccc(cc1)OCCCCCC)c1ccc(cc1)c1sc2c(c1)sc(c2)/C=C(/C(=O)O)\C#N	chloroform	101	10.1021/jp808275z
CCCCC(Cn1nc2c(n1)c(ccc2/C=C(/C(=O)O)\C#N)c1ccc(cc1)N(c1ccccc1)c1ccccc1)CC	THF	-26	10.1039/c5cc07105d

SMILES	SOLVENT	SHIFT (nm)	DOI
CCCCC(Cn1nc2c(n1)c1cc(sc1c1c2cc(s1)c1ccc(cc1)N(c1ccccc1)c1ccccc1)/C=C(/C(=O)O)\C#N)CC	THF	-6	10.1039/c5cc07105d
CCCCCCn1c2cc(sc2c2c1c1n(CCCCCC)c3c(c1c1c2nn(n1)CC(CCCC)CC)sc(c3)/C=C(/C(=O)O)\C#N)c1ccc(cc1)N(c1ccccc1)c1ccccc1	THF	-3	10.1039/c5cc07105d
CCCOc1c2Cc3cc(cc(c3OCCCCC(=O)COc3cc(sc3c3ccc(cc3)N(c3ccccc3)c3ccccc3)/C=C(\C(=O)O)/C#N)Cc3cc(cc(Cc4c(c(Cc1cc(c2)C(C)(C)C)cc(c4)C(C)(C)C)OCCC)c3OCCC)C(C)(C)C)C(C)(C)C	dichloromethane	35	10.1039/c5ra15184h
CCCOc1c2Cc3cc(cc(c3OCCCCCC(=O)COc3cc(sc3c3ccc(cc3)N(c3ccccc3)c3ccccc3)/C=C(\C(=O)O)/C#N)Cc3cc(cc(Cc4c(c(Cc1cc(c2)C(C)(C)C)cc(c4)C(C)(C)C)OCCCCC(=O)COc1cc(sc1c1ccc(cc1)N(c1ccccc1)c1ccccc1)/C=C(\C(=O)O)/C#N)c3OCCC)C(C)(C)C)C(C)(C)C	dichloromethane	33	10.1039/c5ra15184h
N#CC(=Cc1ccc(c(c1)C#Cc1ccc(cc1)N(c1ccccc1)c1ccccc1)C#C)C(=O)O	chloroform	-50	10.1039/c5ra25938j
N#CC(=Cc1ccc(c(c1)C#C)C#Cc1ccc(cc1)N(c1ccccc1)c1ccccc1)C(=O)O	chloroform	4	10.1039/c5ra25938j
N#CC(=Cc1ccc(c(c1)C#Cc1ccc(cc1)N(c1ccccc1)c1ccccc1)C#Cc1ccc(cc1)N(c1ccccc1)c1ccccc1)C(=O)O	chloroform	8	10.1039/c5ra25938j
N#C/C(=C\c1ccc(cc1)c1ccc(cc1)N(c1ccc(cc1)c1cccs1)c1ccc(cc1)c1cccs1)/C(=O)O	chloroform	-25	10.1039/c2cp42993d
OC(=O)CN1C(=S)S/C(=C\c2ccc(cc2)N(c2ccc(cc2)/C=C\2/SC(=S)N(C2=O)CC(=O)O)c2ccccc2)/C1=O	ethanol	33	10.1016/j.jpowsour.2008.12.026
CCCCCCOc1ccc(cc1)N(c1ccc(cc1)OCCCCCC)c1ccc(cc1)c1sc2c(c1)sc(c2)c1sc2c(c1)sc(c2)C=C(C(=O)O)C#N	chloroform	84	10.1039/b817990e
N#C/C(=C/c1ccc(cc1)N(c1ccccc1)c1ccc(cc1)c1nc(c([nH]1)c1ccccc1)c1ccccc1)/C(=O)O	ethanol	-31	10.1016/j.orgel.2013.04.004
N#C/C(=C/c1ccc(cc1)N(c1ccccc1)c1ccc(cc1)c1nc2c([nH]1)c1ccccc1c1c2cccc1)/C(=O)O	ethanol	-29	10.1016/j.orgel.2013.04.004
N#CC(=Cc1ccc(cc1)N(c1ccccc1)c1ccccc1)C(	dichloromethane	9	10.1016/j.tet.2011.09.008

SMILES	SOLVENT	SHIFT (nm)	DOI
=O)O			
CCCCCCCCC1(CCCCCCCC)c2cc(/C=C/c3ccc(cc3)N(c3ccccc3)c3ccccc3)ccc2c2c1cc(cc2)/C=C(\C(=O)O)/C#N	dichloromethane	14	10.1016/j.tet.2011.09.008
CCCCCCCCC1(CCCCCCCC)c2cc(ccc2c2c1cc(cc2)/C=C/c1ccc(cc1)N(c1ccccc1)c1ccccc1)/C=C/c1ccc2c(c1)C(CCCCCCCC)(CCCCCCCC)c1c2ccc(c1)C=C(C(=O)O)C#N	dichloromethane	7	10.1016/j.tet.2011.09.008
CCCCCCCCOc1c(OCCCCCCCC)c(c2ccc(s2)N(c2ccccc2)c2ccccc2)c2c(c1c1ccc(cc1)/C=C(\C(=O)O)/C#N)nsn2	dichloromethane	16	10.1039/c4tc00169a
CCCCC(COc1cc(OCC(CCCC)CC)ccc1c1ccc(cc1)N(c1ccc(cc1)c1ccc(cc1OCC(CCCC)CC)OCC(CCCC)CC)c1ccc(cc1)c1ccc(s1)c1ccc(c2c1nc(c1ccccc1)c(n2)c1ccccc1)c1ccc(cc1)C=C(C(=O)O)C#N)CC	dichloromethane	-3	10.1039/c5ta07254a
CCCCC(COc1cc(OCC(CCCC)CC)ccc1c1ccc(cc1)N(c1ccc(cc1)c1ccc(cc1OCC(CCCC)CC)OCC(CCCC)CC)c1ccc(cc1)c1sc(c2c1OCCO2)c1ccc(c2c1nc(c1ccccc1)c(n2)c1ccccc1)c1ccc(cc1)C=C(C(=O)O)C#N)CC	dichloromethane	2	10.1039/c5ta07254a
CCCCC(CC1(CCC(CCC)CC)c2cc(sc2c2c1cc(s2)c1ccc(c2c1nc(c1ccccc1)c(n2)c1ccccc1)c1ccc(cc1)C=C(C(=O)O)C#N)c1ccc(cc1)N(c1ccc(cc1)c1ccc(cc1OCC(CCCC)CC)OCC(CCCC)CC)c1ccc(cc1)c1ccc(cc1OCC(CCCC)CC)OCC(CCCC)CC)CC	dichloromethane	17	10.1039/c5ta07254a
CCCCCCCCn1c2ccc(cc2c2c1ccc(c2)c1ccc(s1)/C=C(/C(=O)O)\C#N)c1ccc(s1)/C=C(/C(=O)O)\C#N	THF	56	10.1016/j.dyepig.2013.02.012
CCCCCCCCn1c2ccc(cc2c2c1ccc(c2)c1ccc(s1)c1ccc(s1)/C=C(/C(=O)O)\C#N)c1ccc(s1)c1ccc(s1)/C=C(/C(=O)O)\C#N	THF	49	10.1016/j.dyepig.2013.02.012
CCCCCCC1(CCCCCC)c2cc(ccc2c2c1cc(cc2)c1ccc(cc1)n1c(/C=C/c2ccc(cc2)N(c2ccccc2)c2ccccc2)ccc1/C=C(/C(=O)O)\C#N)c1ccc(cc1)n1c(/C=C/c2ccc(cc2)N(c2ccccc2)c2ccccc2)ccc1/C=C(/C(=O)O)\C#N	dichloromethane	-30	10.1039/C2JM30200D
CCCCCCn1c2cc(ccc2c2c1cc(cc2)c1ccc(cc1)n1c(/C=C/c2ccc(cc2)N(c2ccccc2)c2ccccc2)ccc1/C=C(/C(=O)O)\C#N)c1ccc(cc1)n1c(/C=C/c2ccc(cc2)N(c2ccccc2)c2ccccc2)ccc1/C=C(/C(=O)O)\C#N	dichloromethane	-45	10.1039/C2JM30200D

SMILES	SOLVENT	SHIFT (nm)	DOI
CCCCCCOc1cc(c(cc1c1ccc(cc1)n1c(/C=C/c2ccc(cc2)N(c2ccccc2)c2ccccc2)ccc1/C=C(/C(=O)O)\C#N)OCCCCCC)c1ccc(cc1)n1c(/C=C/c2ccc(cc2)N(c2ccccc2)c2ccccc2)ccc1/C=C(/C(=O)O)\C#N	dichloromethane	-34	10.1039/C2JM30200D
N#CC(=Cc1ccc(cc1)N(c1ccccc1)c1ccccc1)C(=O)O	methanol	-20	10.1021/jp076992d
C=Cc1ccc(cc1)N(c1ccccc1)c1ccc(cc1)C=C(C(=O)O)C#N	methanol	-12	10.1021/jp076992d
N#C/C(=C/c1ccc(cc1)N(c1ccc(cc1)/C=C(/C(=O)O)\C#N)c1ccccc1)/C(=O)O	methanol	-4	10.1021/jp076992d
C=Cc1ccc(cc1)N(c1ccccc1)c1ccc(cc1)C=CC=C(C(=O)O)C#N	methanol	-5	10.1021/jp076992d
CCCCCCc1cc(sc1c1ccc(cc1)N(c1ccccc1)c1ccccc1)c1cc(c(s1)/C=C(/C(=O)O)\C#N)CCCCCC	dichloromethane	-112	10.1016/j.jpowsour.2013.01.127
CCCCCCc1cc(sc1c1sc(cc1CCCCCC)/C=C(/C(=O)O)\C#N)c1ccc(cc1)N(c1ccccc1)c1ccccc1	dichloromethane	-106	10.1016/j.jpowsour.2013.01.127
CCCCCCc1cc(sc1c1ccc(cc1)N(c1ccccc1)c1ccccc1)c1sc(cc1CCCCCC)/C=C(/C(=O)O)\C#N	dichloromethane	-90	10.1016/j.jpowsour.2013.01.127
CCCCCCc1cc(sc1c1cc(c(s1)/C=C(/C(=O)O)\C#N)CCCCCC)c1ccc(cc1)N(c1ccccc1)c1ccccc1	dichloromethane	-83	10.1016/j.jpowsour.2013.01.127
N#CC(=Cc1ccc(cc1)N(c1ccccc1)c1ccccc1)C(=O)O	acetonitrile	-14	10.1021/jo701592x
N#CC(=Cc1ccc(s1)c1ccc(cc1)N(c1ccccc1)c1ccccc1)C(=O)O	acetonitrile	0	10.1021/jo701592x
N#CC(=Cc1ccc(s1)/C=C/c1ccc(s1)/C=C/c1ccc(cc1)N(c1ccccc1)c1ccccc1)C(=O)O	acetonitrile	33	10.1021/jo701592x
N#C/C(=C\c1ccc(s1)C=Cc1ccc(cc1)N(c1ccccc1)c1ccccc1)/C(=O)O	acetonitrile	48	10.1021/jo701592x
CCCCCCc1cc(sc1c1ccc(cc1)N(c1ccc2c(c1)C(C)(C)c1c2cccc1)c1ccc2c(c1)C(C)(C)c1c2cccc1)c1sc(cc1CCCCCC)c1sc(cc1CCCCCC)/C=C(/C(=O)O)\C#N	THF	20	10.1002/ange.200703852
CCCCCCC1=CC(=c2sc(=C3SC(C=C3CCCCCC)(C=O)/C=C(/C(=O)O)\C#N)cc2CCCCCC)SC1c1cc2c(s1)cc(cc2)N(c1ccc2c(c1)C(C)(C)c1c2cccc1)c1ccc2c(c1)C(C)(C)c1c2cccc1	THF	20	10.1002/ange.200703852
N#C/C(=C\c1cc(c(s1)c1ccc(s1)c1ccc(cc1)N(c1ccccc1)c1ccccc1)c1ccc(s1)c1ccc(cc1)N(c1ccccc1)c1cc	THF	62	10.1021/cm702631r

SMILES	SOLVENT	SHIFT (nm)	DOI
ccc1)/C(=O)O			
N#C/C(=C\ c1ccc(s1)c1ccc(s1)c1ccc(cc1)N(c1ccccc1)c1ccccc1)/C(=O)O	THF	48	10.1021/cm702631r
CCCCCCCCc1ccc(cc1)C1(c2ccc(cc2)CCCCCCCC)c2cc3c4ccc(cc4C(c3cc2c2c1cc1c3cc4Cc5c(c4cc3C(c1c2)(c1ccc(cc1)CCCCCCCC)c1ccc(cc1)CCCCCCCC)ccc(c5)/C=C(/C(=O)O)\C#N)(CCCCCCCC)CCCCCCCC)N(c1ccc(cc1)C)c1ccc(cc1)C	dichloromethane	37	10.1021/cm703459p
CCCCCCCCc1ccc(cc1)C1(c2ccc(cc2)CCCCCCCC)c2cc3c4cc5c(cc4C(c3cc2c2c1cc1c(c2)Cc2c1ccc(c2)/C=C(/C(=O)O)\C#N)(c1ccc(cc1)CCCCCCCC)c1ccc(cc1)CCCCCCCC)c1c(C5(CCCCCCCC)CCCCCCCC)cc(cc1)N1c2ccccc2Sc2c1cccc2	dichloromethane	37	10.1021/cm703459p
CCCCCCCCc1ccc(cc1)C1(c2ccc(cc2)CCCCCCCC)c2cc3c4cc5Cc6c(c5cc4C(c3cc2c2c1cc1c3ccc(cc3C(c1c2)(CCCCCCCC)CCCCCCCC)N(c1ccc(cc1)N(C)C)c1ccc(cc1)N(C)C)(c1ccc(cc1)CCCCCCCC)c1ccc(cc1)CCCCCCCC)ccc(c6)/C=C(/C(=O)O)\C#N	dichloromethane	23	10.1021/cm703459p
N#CC(=Cc1ccc(s1)c1c2ccccc2c(c2c1cccc2)c1ccc(cc1)N(c1ccccc1)c1ccccc1)C(=O)O	dichloromethane	4	10.1021/jp101238k
N#CC(=Cc1ccc(cc1)C=Cc1c2ccccc2c(c2c1cccc2)c1ccc(cc1)N(c1ccccc1)c1ccccc1)C(=O)O	dichloromethane	2	10.1021/jp101238k
N#CC(=Cc1ccc(cc1)c1c2ccccc2c(c2c1cccc2)c1ccc(cc1)N(c1ccccc1)c1ccccc1)C(=O)O	dichloromethane	24	10.1021/jp101238k
N#CC(=Cc1ccc(s1)C=Cc1c2ccccc2c(c2c1cccc2)c1ccc(cc1)N(c1ccccc1)c1ccccc1)C(=O)O	dichloromethane	6	10.1021/jp101238k
N#C/C(=C\ c1ccc(s1)C#Cc1c2ccccc2c(c2c1cccc2)c1ccc(cc1)N(c1ccccc1)c1ccccc1)/C(=O)O	dichloromethane	24	10.1021/jp101238k
N#C/C(=C\ c1ccc(cc1)C#Cc1c2ccccc2c(c2c1cccc2)c1ccc(cc1)N(c1ccc(cc1)OC)c1ccc(cc1)OC)/C(=O)O	dichloromethane	-3	10.1021/jp101238k
N#C/C(=C\ c1ccc(s1)C#Cc1c2ccccc2c(c2c1cccc2)c1ccc(cc1)N(c1ccc(cc1)OC)c1ccc(cc1)OC)/C(=O)O	dichloromethane	29	10.1021/jp101238k
N#C/C(=C\ c1ccc(cc1)c1c2ccccc2c(c2c1cccc2)C#Cc1ccc(cc1)N(c1ccccc1)c1ccccc1)/C(=O)O	dichloromethane	-5	10.1021/jp101238k

SMILES	SOLVENT	SHIFT (nm)	DOI
N#C/C(=C\c1ccc(cc1)c1c2ccccc2c(c2c1cccc2)C#Cc1ccc(cc1)N(c1ccc(cc1)OC)c1ccc(cc1)OC)/C(=O)O	dichloromethane	-1	10.1021/jp101238k
N#CC(=Cc1ccc2c(c1)C(C)(C)c1c2ccc(c1)c1ccc(cc1)N(c1ccccc1)c1ccccc1)C(=O)O	THF	51	10.1002/cplu.201200127
N#CC(=Cc1ccc2c(c1)C(C)(C)c1c2ccc(c1)c1ccc(s1)c1ccc(cc1)N(c1ccccc1)c1ccccc1)C(=O)O	THF	32	10.1002/cplu.201200127
N#CC(=Cc1ccc2c(c1)C(C)(C)c1c2ccc(c1)c1ccc(s1)c1ccc(s1)c1ccc(cc1)N(c1ccccc1)c1ccccc1)C(=O)O	THF	32	10.1002/cplu.201200127
CCCCCCCCc1cc(sc1c1sc(c(c1)CCCCCCCC)c1ccc(cc1)N(c1ccccc1)c1ccccc1)c1ccc2c(c1)C(C)(C)c1c2ccc(c1)C=C(C(=O)O)C#N	THF	34	10.1002/cplu.201200127
N#CC(=Cc1ccc2c(c1)C(C)(C)c1c2ccc(c1)c1sc(c2c1OCCO2)c1ccc(cc1)N(c1ccccc1)c1ccccc1)C(=O)O	THF	33	10.1002/cplu.201200127
N#CC(=Cc1ccc(s1)c1ccc(s1)c1sc(c2c1OCCO2)c1ccc(cc1)N(c1ccccc1)c1ccccc1)C(=O)O	THF	55	10.1002/cplu.201200127
N#CC(=Cc1ccc2c(c1)C(C)(C)c1c2ccc(c1)c1ccc(s1)c1sc(c2c1OCCO2)c1ccc(cc1)N(c1ccccc1)c1ccccc1)C(=O)O	THF	37	10.1002/cplu.201200127
Cc1ccc(cc1)C1(c2ccc(cc2)C)c2cc(ccc2c2c1cc(s2)c1ccc(c2c1nsn2)c1ccc(cc1)C(=O)O)N(c1ccc(cc1)C)c1ccc(cc1)C	chloroform	17	10.1016/j.jpowsour.2016.06.099
Cc1ccc(cc1)C1(c2ccc(cc2)C)c2cc(sc2c2c1cc(cc2)N(c1ccc(cc1)C)c1ccc(cc1)C)c1c(F)c(F)c(c2c1nsn2)c1ccc(cc1)C(=O)O	chloroform	5	10.1016/j.jpowsour.2016.06.099
CCCCCCC1(CCCCCC)c2cc(ccc2c2c1c1c3ccc(cc3C(c1c1c2C(CCCCCC)(CCCCCC)c2c1ccc(c2)c1ccc(s1)/C=C(/C(=O)O)\C#N)(CCCCCC)CCCCCC)c1ccc(cc1)N(c1ccccc1)c1ccccc1)c1ccc(cc1)N(c1ccccc1)c1ccccc1	chloroform	58	10.1016/j.cclet.2015.03.008
CCCCCCC1(CCCCCC)c2cc(ccc2c2c1c1c3ccc(cc3C(c1c1c2C(CCCCCC)(CCCCCC)c2c1ccc(c2)c1ccc(s1)/C=C(/C(=O)O)\C#N)(CCCCCC)CCCCCC)c1ccc(s1)/C=C(/C(=O)O)\C#N)c1ccc(cc1)N(c1ccccc1)c1ccccc1	chloroform	2	10.1016/j.cclet.2015.03.008
CCCCCCC1(CCCCCC)c2cc(ccc2c2c1c1c3ccc(cc3C(c1c1c2C(CCCCCC)(CCCCCC)c2c1ccc(c2)c1ccc(cc1)N(c1ccccc1)	chloroform	110	10.1016/j.cclet.2015.03.008

SMILES	SOLVENT	SHIFT (nm)	DOI
c1ccccc1)(CCCCCC)CCCCCC)c1ccc(cc1)N(c1ccccc1)c1ccccc1)c1ccc(s1)/C=c\1/sc(=S)n(o1)CC(=O)O			
OC(=O)CN1C(=S)SC(=Cc2ccc(cc2)Nc2ccc(cc2)c2ccccc2)C1=O	ethanol	-5	10.1016/j.matchemphys.2011.07.038
OC(=O)CN1C(=S)SC(=Cc2ccc(cc2)NC2=CCC(C=C2)(C=C2SC(=S)N(C2=O)CC(=O)O)c2ccccc2)C1=O	ethanol	-52	10.1016/j.matchemphys.2011.07.038
OC(=O)CN1C(=S)SC(=Cc2ccc(cc2)N(c2ccc(cc2)c2ccc3c(c2)[nH]c2c3cccc2)c2ccccc2)C1=O	ethanol	-34	10.1016/j.matchemphys.2011.07.038
OC(=O)CN1C(=S)SC(=Cc2ccc(cc2)N(c2ccc(cc2)c2ccc3c(c2)[nH]c2c3cccc2)c2ccc(cc2)C=C2SC(=S)N(C2=O)CC(=O)O)C1=O	ethanol	-48	10.1016/j.matchemphys.2011.07.038
N#C/C(=C\c1ccc(s1)c1ccc2c(c1)c1ccccc1n2c1ccc(cc1)N(c1ccc(cc1)n1c2ccccc2c2c1cccc2)c1ccc(cc1)n1c2ccccc2c2c1cccc2)/C(=O)O	THF	-20	10.1039/C5RA04283F
OC(=O)CN1C(=S)S/C(=C\c2ccc(s2)c2ccc3c(c2)c2ccccc2n3c2ccc(cc2)N(c2ccc(cc2)n2c3ccccc3c3c2cccc3)c2ccc(cc2)n2c3ccccc3c3c2cccc3)/C1=O	THF	-13	10.1039/C5RA04283F
N#C/C(=C/c1ccc(s1)/C=C/c1ccc(cc1)N(c1ccc(cc1)c1ccc2c(c1)C(C)(C)c1c2cccc1)c1ccc(cc1)c1ccc2c(c1)C(C)(C)c1c2cccc1)/C(=O)O	ethanol	6	10.1016/j.tet.2007.08.058
N#C/C(=C/c1cc(c(s1)/C=C/c1ccc(cc1)N(c1ccc(cc1)c1ccc2c(c1)C(C)(C)c1c2cccc1)c1ccc(cc1)c1ccc2c(c1)C(C)(C)c1c2cccc1)C)/C(=O)O	ethanol	6	10.1016/j.tet.2007.08.058
CCCCCCc1cc(sc1/C=C/c1ccc(cc1)N(c1ccc(cc1)c1ccc2c(c1)C(C)(C)c1c2cccc1)c1ccc(cc1)c1ccc2c(c1)C(C)(C)c1c2cccc1)/C=C(\C(=O)O)/C#N	ethanol	6	10.1016/j.tet.2007.08.058
N#C/C(=C\c1ccc(s1)c1ccc(n1C)c1ccc(cc1)N(c1ccccc1)c1ccccc1)/C(=O)O	THF	-48	10.1021/jp801036s
CCN(c1ccc(c(c1)O)/C=C/c1sc2c([n+]1CC(=O)[O-])cccc2)CC	methanol	40.5	10.1039/B418906J
CCN(c1ccc(cc1)/C=C/c1sc2c([n+]1CC(=O)[O-])cccc2)CC	methanol	31.5	10.1039/B418906J

SMILES	SOLVENT	SHIFT (nm)	DOI
CCN(c1ccc(c(c1)O)/C=C/c1sc2c([n+]1CCC(=O)[O-])cccc2)CC	methanol	59	10.1039/B418906J
CCN(c1ccc(cc1)/C=C/c1sc2c([n+]1CCC(=O)[O-])cccc2)CC	methanol	40.5	10.1039/B418906J
CCN(c1ccc(c(c1)O)/C=C/c1sc2c([n+]1CCCS(=O)(=O)[O-])cccc2)CC	methanol	62	10.1039/B418906J
CCN(c1ccc(c(c1)O)/C=C/c1sc2c([n+]1CCCS(=O)(=O)[O-])ccc1c2cccc1)CC	methanol	33	10.1039/B418906J
[O-]C1=C(/C=C\2/N(Cc3ccc(cc3)C(=O)O)c3c(C2(C)C)cccc3)C(=O)/C/1=C\C1=[N+](Cc2ccc(cc2)C(=O)O)c2c(C1(C)C)cccc2	methanol	-18	10.1016/j.jphotochem.2004.10.016
[O-]C1=C(/C=C\2/N(Cc3ccc(cc3)C(=O)O)c3c(C2(C)C)cccc3)C(=O)/C/1=C\C1=[N+](Cc2ccc(cc2)C(=O)O)c2c(C1(C)C)cccc2	ethanol	-14	10.1016/j.jphotochem.2004.10.016
[O-]C1=C(/C=C\2/N(Cc3ccc(cc3)C(=O)O)c3c(C2(C)C)cccc3)C(=O)/C/1=C\C1=[N+](Cc2ccc(cc2)C(=O)O)c2c(C1(C)C)cccc2	acetonitrile	-16	10.1016/j.jphotochem.2004.10.016
[O-]C1=C(/C=C\2/N(Cc3ccc(cc3)C(=O)O)c3c(C2(C)C)cccc3)C(=O)/C/1=C\C1=[N+](Cc2ccc(cc2)C(=O)O)c2c(C1(C)C)cccc2	chloroform	-13	10.1016/j.jphotochem.2004.10.016
CCN1c2ccccc2C(/C/1=C/C=C/1\SC(=S)N(C1=O)CC(=O)O)(C)C	ethanol	-30	10.1039/b001517m
CCn1/c(=C\C=C\2/SC(=S)N(C2=O)CC(=O)O)/sc2c1cccc2	ethanol	-40	10.1039/b001517m
CCCCCn1/c(=C\C=C\2/SC(=S)N(C2=O)CC(=O)O)/sc2c1cccc2	ethanol	-40	10.1039/b001517m
CCCCCCCCCCn1/c(=C\C=C\2/SC(=S)N(C2=O)CC(=O)O)/sc2c1cccc2	ethanol	-50	10.1039/b001517m
CCCCCCCCCCCCCCCCCCn1/c(=C\C=C\2/SC(=S)N(C2=O)CC(=O)O)/sc2c1cccc2	ethanol	-70	10.1039/b001517m
OC(=O)CN1C(=S)S/C(=C\C=C\2/N(CC(=O)O)c3c(C2(C)C)cccc3)/C1=O	ethanol	-30	10.1039/b001517m
OC(=O)Cc1ccc2c(c1)C(C)(C)/C(=C/C=C/C1=[N+](C)c3c(C1(C)C)cccc3)/N2C	ethanol	-15	10.1016/S0927-0248(03)00113-2
OC(=O)Cc1ccc2c(c1)C(C)(C)/C(=C\C=C\C=C/C1=[N+](C)c3c(C1(C)C)cccc3)/N2C	ethanol	70	10.1016/S0927-0248(03)00113-2
CN(c1ccc(cc1)/C=C/c1cc[n+](cc1)CCCS(=O)(=O)[O-])C	methanol	40	10.1021/jp001580p

SMILES	SOLVENT	SHIFT (nm)	DOI
CN(c1ccc(cc1)/C=C/c1cc[n+](c2c1cccc2)CCCS(=O)(=O)[O-])C	methanol	44	10.1021/jp001580p
CCCCCCCCCCCCCCCCN(c1ccc(cc1)/C=C/c1cc[n+](cc1)CCCS(=O)(=O)[O-])C	methanol	26	10.1021/jp001580p
CCCCCCCCCCCCCCCCN(c1ccc(cc1)/C=C/c1cc[n+](c2c1cccc2)CCCS(=O)(=O)[O-])C	methanol	16	10.1021/jp001580p
CN(c1ccc(cc1)/C=C/C1=[N+](CCCS(=O)(=O)[O-])c2c(C1(C)C)cccc2)C	chloroform	96	10.1021/jp010667n
CN(c1ccc(cc1)/C=C/c1sc2c([n+]1CCCS(=O)(=O)[O-])cccc2)C	chloroform	25	10.1021/jp010667n
CN(c1ccc(cc1)/C=C/C1=[N+](CCCS(=O)(=O)[O-])c2c(C1(C)C)cccc2)C	methanol	68	10.1021/jp010667n
CN(c1ccc(cc1)/C=C/c1sc2c([n+]1CCCS(=O)(=O)[O-])cccc2)C	methanol	11	10.1021/jp010667n
CCN(c1ccc(cc1)/C=C/C1=[N+](C)c2c(C1(C)C)cccc2)CCC(=O)O	ethanol	11	10.1039/b300083b
OC(=O)CCN(c1ccc(cc1)/C=C/C1=[N+](C)c2c(C1(C)C)cccc2)CCC(=O)O	ethanol	-15	10.1039/b300083b
CCN(c1ccc(cc1)/C=C/C1=[N+](C)C2=C(C1(C)C)C1C=CC=CC1C=C2)CCC(=O)O	ethanol	37	10.1039/b300083b
OC(=O)CCN(c1ccc(cc1)/C=C/C1=[N+](C)C2=C(C1(C)C)C1C=CC=CC1C=C2)CCC(=O)O	ethanol	-32	10.1039/b300083b
CCCCCC[N+]1=C(/C=C/2\C([O-])C(C2=O)c2ccc(cc2)N(CCCC)CCCC)c2c3c1cccc3c(cc2)C(=O)O	DMF	-37	10.1016/j.dyepig.2015.06.022
CCCCCC[N+]1=C(/C=C/2\C([O-])C(C2=O)c2ccc(cc2)N(CCCC)CCCC)c2c3c1cccc3c(cc2)C(=O)O	DMSO	-31	10.1016/j.dyepig.2015.06.022
CCCCCC[N+]1=C(/C=C/2\C([O-])C(C2=O)c2ccc(cc2)N(CCCC)CCCC)c2c3c1cccc3c(cc2)C(=O)O	ethanol	-48	10.1016/j.dyepig.2015.06.022
CCCCCC[N+]1=C(/C=C/2\C([O-])C(C2=O)c2ccc(cc2)N(CCCC)CCCC)c2c3c1cccc3c(cc2)C(=O)O	chloroform	31	10.1016/j.dyepig.2015.06.022
CCCCCC[N+]1=C(/C=C/2\C([O-])C(C2=O)c2ccc(cc2)N(c2ccc3c(c2)C(CCCCCC)(CCCCCC)c2c3cccc2)c2ccc3c(c2)C(CCCCCC)(CCCCCC)c2c3cccc2)c2c3c1cccc3c(cc2)C(=O)O	DMF	-39	10.1016/j.dyepig.2015.06.022
CCCCCC[N+]1=C(/C=C/2\C([O-])C(C2=O)c2ccc(cc2)N(c2ccc3c(c2)C(C	ethanol	-31	10.1016/j.dyepig.2015.06.022

SMILES	SOLVENT	SHIFT (nm)	DOI
CCCCC)(CCCCCC)c2c3cccc2)c2ccc3c(c2)C(CCCCCC)(CCCCCC)c2c3cccc2)c2c3c1cccc3c(cc2)C(=O)O			
CCCCCC[N+]1=C(/C=C/2\C([O-])C(C2=O)c2ccc(cc2)N(c2ccc3c(c2)C(CCCCCC)(CCCCCC)c2c3cccc2)c2ccc3c(c2)C(CCCCCC)(CCCCCC)c2c3cccc2)c2c3c1cccc3c(cc2)C(=O)O	chloroform	50	10.1016/j.dyepig.2015.06.022
CCCCCCOc1ccc(cc1)N(c1ccc(cc1)OCCCCCC)c1ccc(cc1)c1sc(c2c1OCCO2)C1=C([O-])/C(=C\c2ccc3c([n+]2C)ccc(c3)/C=C/C(=O)O)/C1=O	ethanol	56	10.1021/ol302481k
CCCCCCOc1ccc(cc1)N(c1ccc(cc1)OCCCCCC)c1ccc(cc1)c1sc(c2c1OCCO2)C1=C([O-])/C(=C\c2ccc3c([n+]2C)ccc(c3)C(=O)O)/C1=O	ethanol	23	10.1021/ol302481k
CC[N+]1=C(/C=C/2\C(=C(C2=O)c2ccc(s2)n2c3ccccc3c3c2cccc3)[O-])Cc2c1ccc(c2)C(=O)O	dichloromethane	5	10.1039/C5CP03371C
CC[N+]1=C(/C=C/2\C(=C(C2=O)c2ccc(s2)n2c3ccc(cc3c3c2ccc(c3)C(C)(C)C)C(C)(C)C)[O-])Cc2c1ccc(c2)C(=O)O	dichloromethane	-3	10.1039/C5CP03371C
CCCCCCCCCCC1(CCCCCCCC)C(=[N+](c2c1cccc2)CCCCCC)/C=C/1\C(=C(C1=O)/C=C/1\N(C)c2c(C1(C)C)cc(cc2)c1ccc(cc1)C=C(C(=O)O)C#N)[O-]	chloroform	-15	10.1021/acsami.7b08346
CCCCCCCCCCC1(CCCCCCCC)c2cc(ccc2N(/C/1=C\C1=C([O-])/C(=C\C2=[N+](C)c3c(C2(C)C)cccc3)/C1=O)CCCCCCC)c1ccc(cc1)C=C(C(=O)O)C#N	chloroform	-13	10.1021/acsami.7b08346
CCCCCCCCCCC1(CCCCCCCC)C(=[N+](c2c1cccc2)CCCCCC)/C=C/1\C(=C(C1=O)/C=C/1\N(C)c2c(C1(C)C)cc(cc2)c1ccc(s1)C=C(C(=O)O)C#N)[O-]	chloroform	-13	10.1021/acsami.7b08346
CCCCCCCCCCC1(CCCCCCCC)c2cc(ccc2N(/C/1=C\C1=C([O-])/C(=C\C2=[N+](C)c3c(C2(C)C)cccc3)/C1=O)CCCCCC)c1ccc(s1)C=C(C(=O)O)C#N	chloroform	-15	10.1021/acsami.7b08346
CCCCCCCCCCC1(CCCCCCCC)C(=[N+](c2c1cccc2)CCCCCCCCCCC)/C=C/1\C(=C(C1=O)/C=C/1\	chloroform	-15	10.1021/acsami.7b08346

SMILES	SOLVENT	SHIFT (nm)	DOI
N(C)c2c(C1(C)C)cc(cc2)c1ccc(s1)C=C(C(=O)O)C#N)[O-]			
CCCCCCCCOc1ccc(cc1)N(c1ccc(cc1)C=c1sc(=C(C#N)C#N)[nH]c1=O)c1ccc(cc1)OCCCCCCCC	dichloromethane	11	10.1002/anie.201204948
CCCCCCCCOc1ccc(cc1)N(c1ccc(cc1)OCCCCCCCC)c1ccc(cc1)c1ccc(s1)C=c1sc(=C(C#N)C#N)[nH]c1=O	dichloromethane	42	10.1002/anie.201204948
CCCCCCCCOc1ccc(cc1)N(c1ccc(cc1)C=C(C(=O)O)C#N)c1ccc(cc1)OCCCCCCCC	dichloromethane	17	10.1002/anie.201204948
CCCCCCCCOc1ccc(cc1)N(c1ccc(cc1)c1ccc(s1)C=C(C(=O)O)C#N)c1ccc(cc1)OCCCCCCCC	dichloromethane	19	10.1002/anie.201204948
N#C/C(=C/1\c2sc(cc2c2c1cccc2)c1ccc(cc1)N(c1ccccc1)c1ccccc1)/C(=O)O	THF	-11	10.1002/ajoc.201600100
CCCCCCOc1ccc(cc1)N(c1ccc(cc1)OCCCCCC)c1ccc(cc1)c1ccc(s1)c1sc2c(c1)c1c(/C/2=C(\C(=O)O)/C#N)cccc1	THF	-36	10.1002/ajoc.201600100
CCCCCCOc1ccc(cc1)N(c1ccc(cc1)OCCCCCC)c1ccc(cc1)c1ccc(s1)c1sc2c(c1)c1c(/C/2=C\c2ccc(s2)/C=C(/C(=O)O)\C#N)cccc1	THF	-14	10.1002/ajoc.201600100
CCCCCCOc1ccc(cc1)N(c1ccc(cc1)OCCCCCC)c1ccc(cc1)c1ccc(s1)c1sc2c(c1)c1c(/C/2=C\c2ccc(s2)/C=C(/C(=O)O)\C#N)cccc1	THF	-52	10.1002/ajoc.201600100
N#C/C(=C\c1ccc(s1)c1ccc(s1)/C=C/1\c2sc(cc2c2c1cc(cc2)c1ccc(cc1)N(c1ccccc1)c1ccccc1)c1ccc(cc1)N(c1ccccc1)c1ccccc1)/C(=O)O	THF	-20	10.1002/ajoc.201600100
COCCOC#C[B-]1(C#COCCOC)[N+]2=C(C)C(C(=C2C(=C2N1C(C)C(=C2C)CC)c1ccc(cc1)C(=O)O)C)CC	acetonitrile	-5	10.1002/chem.200900518
COCCOCCOc1ccc(cc1)/C=C/C1C(=C(C2=C(c3ccc(cc3)C(=O)O)C3=C(C(C(=[N+]3[B-](N12)(C#COCCOC)C#COCCOC)C)CC)C)C)CC	acetonitrile	-10	10.1002/chem.200900518
COCCOCCOc1ccc(cc1)/C=C/C1C(=C(C2=C(c3ccc(cc3)C(=O)O)C3=C(C(C(=[N+]3[B-](N12)(C#COCCOC)C#COCCOC)/C=C/c1ccc(cc1)OCCOCCOC)CC)C)C)CC	acetonitrile	-21	10.1002/chem.200900518
CCCCCCCCc1cc(sc1c1nc2c(s1)nc(s2)c1ccc(s1)c1ccc(cc1)N(c1ccccc1)c1ccccc1)C=C(C(=O)O)C#N	THF	21	10.1002/asia.201201202
CCCCCCCCc1cc(sc1c1nc2c(s1)nc(s2)c1ccc(s1)c1ccc(cc1)N(c1ccc(cc1)OC)c1ccc(cc1)OC)	THF	21	10.1002/asia.201201202

SMILES	SOLVENT	SHIFT (nm)	DOI
C=C(C(=O)O)C#N			
CCCCCCCCc1cc(sc1c1sc2c(n1)sc(n2)c1ccc(s1)c1ccc(cc1)N(c1ccc(cc1)OCCCC)c1ccc(cc1)OCCCC)C=C(C(=O)O)C#N	THF	23	10.1002/asia.201201202
CCCCCCCCOc1ccc(cc1)N(c1ccc(cc1)OCCCCCCCC)c1ccc(cc1)c1ccc(s1)c1nc2c(s1)nc(s2)c1sc(cc1CCCCCCCC)C=C(C(=O)O)C#N	THF	26	10.1002/asia.201201202
CCCCn1c(c2ccc(cc2)c2ccc(cc2)N(c2ccccc2)c2ccccc2)c2c(c1=O)c(n(c2=O)CCCC)c1ccc(cc1)c1ccc(o1)/C=C(/C(=O)O)\C#N	dichloromethane	-32	10.1002/asia.201200648
CCCCn1c(c2ccc(cc2)c2ccc(cc2)N(c2ccccc2)c2ccccc2)c2c(c1=O)c(n(c2=O)CCCC)c1ccc(cc1)c1ccc(cc1)C=C(C(=O)O)C#N	dichloromethane	-5	10.1002/asia.201200648
CCCCn1c(c2ccc(cc2)c2ccc3c(c2)C2CCCC2N3c2ccc(cc2)C)c2c(c1=O)c(n(c2=O)CCCC)c1ccc(cc1)c1ccc(o1)/C=C(/C(=O)O)\C#N	dichloromethane	-7	10.1002/asia.201200648
CCCCn1c(c2ccc(cc2)c2ccc3c(c2)C2CCCC2N3c2ccc(cc2)C)c2c(c1=O)c(n(c2=O)CCCC)c1ccc(cc1)c1ccc(cc1)C=C(C(=O)O)C#N	dichloromethane	-16	10.1002/asia.201200648
CCCCCCN1C(=O)c2c(C1=O)c(sc2c1ccc(s1)c1ccc(cc1)N(c1ccccc1)c1ccccc1)c1ccc(s1)C=C(C(=O)O)C#N	THF	50	10.1002/asia.201200720
CCCCCCN1C(=O)c2c(C1=O)c(sc2c1ccc(s1)c1cc2CCCN3c2c(c1)CCC3)c1ccc(s1)C=C(C(=O)O)C#N	THF	71	10.1002/asia.201200720
CCCCC(CN1C(=O)c2c(C1=O)c(sc2c1ccc(s1)c1ccc(cc1)N(c1ccccc1)c1ccccc1)c1ccc(s1)C=C(C(=O)O)C#N)CC	THF	50	10.1002/asia.201200720
CCCCC(CN1C(=O)c2c(C1=O)c(sc2c1ccc(s1)c1cc2CCCN3c2c(c1)CCC3)c1ccc(s1)C=C(C(=O)O)C#N)CC	THF	58	10.1002/asia.201200720
CCCc1nc2c(n1C)c(cnc2c1ccc(s1)c1ccc(cc1)N(c1ccc(cc1)OCCCCCC)c1ccc(cc1)OCCCCCC)c1ccc(s1)C=C(C(=O)O)C#N	toluene	-38	10.1002/asia.201700039
CCCc1nc2c(n1C)c(ncc2c1ccc(s1)C=C(C(=O)O)C#N)c1ccc(s1)c1ccc(cc1)N(c1ccc(cc1)OCCCCCC)c1ccc(cc1)OCCCCCC	toluene	-23	10.1002/asia.201700039
CCCCCCOc1ccc(cc1)N(c1ccc(cc1)OCCCCCC)c1ccc(cc1)c1ccc(s1)c1ccc(c2c1n(C)c(n2)CCC)c1ccc(s1)C=C(C(=O)O)C#N	toluene	-7	10.1002/asia.201700039
CCCCCCOc1ccc(cc1)N(c1ccc(cc1)OCCCCCC)c1ccc(cc1)c1ccc(s1)c1ccc(c2c1n(CCCCCC)c(n2)c1ccc(cc1)OCCCCCC)c1cc(c(s1)C=C(C(=O)O)C#N)CCCCCC	toluene	-3	10.1002/asia.201700039

SMILES	SOLVENT	SHIFT (nm)	DOI
CCCCCCOc1ccc(cc1)N(c1ccc(cc1)OCCCCCC)c1ccc(cc1)c1ccc(s1)c1ccc(c2c1n(C)c(n2)c1ccc(cc1)OCCCCCC)c1cc(c(s1)C=C(C(=O)O)C#N)CCCCCC	toluene	38	10.1002/asia.201700039
CCCCn1c(nc2c1c1cccc3c1c1c2cccc1cc3)c1ccc(cc1)c1ccc(s1)/C=C(/C(=O)O)\C#N	THF	-80	10.1002/ajoc.201402214
CCCCn1c(nc2c1c1cccc3c1c1c2cccc1cc3)c1ccc(s1)c1ccc(s1)/C=C(/C(=O)O)\C#N	THF	-92	10.1002/ajoc.201402214
CCCCn1c(nc2c1c1cccc3c1c1c2cccc1cc3)c1ccc(s1)c1ccc(s1)c1ccc(s1)/C=C(/C(=O)O)\C#N	THF	-116	10.1002/ajoc.201402214
CCCCn1c(nc2c1c1cccc3c1c1c2cccc1cc3)c1ccc2c(c1)C(CC)(CC)c1c2ccc(c1)c1ccc(s1)/C=C(/C(=O)O)\C#N	THF	-86	10.1002/ajoc.201402214
CCCCn1c(nc2c1c1cccc3c1c1c2cccc1cc3)c1ccc(cc1)c1ccc(s1)c1ccc(s1)/C=C(/C(=O)O)\C#N	THF	-76	10.1002/ajoc.201402214
CCCCn1c(nc2c1c1cccc3c1c1c2cccc1cc3)c1ccc2c(c1)C(CC)(CC)c1c2ccc(c1)c1ccc(s1)c1ccc(s1)/C=C(/C(=O)O)\C#N	THF	-104	10.1002/ajoc.201402214
CCCCn1c(nc2c1c1cccc3c1c1c2cccc1cc3)c1ccc(cc1)c1ccc(s1)/C=C(/C(=O)O)\C#N	dichloromethane	-81	10.1002/ajoc.201402214
CCCCn1c(nc2c1c1cccc3c1c1c2cccc1cc3)c1ccc(s1)c1ccc(s1)/C=C(/C(=O)O)\C#N	dichloromethane	-108	10.1002/ajoc.201402214
CCCCn1c(nc2c1c1cccc3c1c1c2cccc1cc3)c1ccc(s1)c1ccc(s1)c1ccc(s1)/C=C(/C(=O)O)\C#N	dichloromethane	-115	10.1002/ajoc.201402214
CCCCn1c(nc2c1c1cccc3c1c1c2cccc1cc3)c1ccc2c(c1)C(CC)(CC)c1c2ccc(c1)c1ccc(s1)/C=C(/C(=O)O)\C#N	dichloromethane	-78	10.1002/ajoc.201402214
CCCCn1c(nc2c1c1cccc3c1c1c2cccc1cc3)c1ccc(cc1)c1ccc(s1)c1ccc(s1)/C=C(/C(=O)O)\C#N	dichloromethane	-63	10.1002/ajoc.201402214
CCCCn1c(nc2c1c1cccc3c1c1c2cccc1cc3)c1ccc2c(c1)C(CC)(CC)c1c2ccc(c1)c1ccc(s1)c1ccc(s1)/C=C(/C(=O)O)\C#N	dichloromethane	-91	10.1002/ajoc.201402214
CCCCn1c(nc2c1c1cccc3c1c1c2cccc1cc3)c1ccc(cc1)c1ccc(s1)/C=C(/C(=O)O)\C#N	DMF	-96	10.1002/ajoc.201402214
CCCCn1c(nc2c1c1cccc3c1c1c2cccc1cc3)c1ccc(s1)c1ccc(s1)/C=C(/C(=O)O)\C#N	DMF	-121	10.1002/ajoc.201402214
CCCCn1c(nc2c1c1cccc3c1c1c2cccc1cc3)c1ccc(s1)c1ccc(s1)c1ccc(s1)/C=C(/C(=O)O)\	DMF	-138	10.1002/ajoc.201402214

SMILES	SOLVENT	SHIFT (nm)	DOI
C#N			
CCCCn1c(nc2c1c1cccc3c1c1c2cccc1cc3)c1ccc2c(c1)C(CC)(CC)c1c2ccc(c1)c1ccc(s1)/C=C(/C(=O)O)\C#N	DMF	-106	10.1002/ajoc.201402214
CCCCn1c(nc2c1c1cccc3c1c1c2cccc1cc3)c1ccc(cc1)c1ccc(s1)c1ccc(s1)/C=C(/C(=O)O)\C#N	DMF	-86	10.1002/ajoc.201402214
CCCCn1c(nc2c1c1cccc3c1c1c2cccc1cc3)c1ccc2c(c1)C(CC)(CC)c1c2ccc(c1)c1ccc(s1)c1ccc(s1)/C=C(/C(=O)O)\C#N	DMF	-128	10.1002/ajoc.201402214
CCCCn1c(nc2c1c1cccc3ccc4cccc2c4c13)-c1ccc(cc1)-c1ccc(\C=C(/C#N)C(O)=O)s1	acetonitrile	-100	10.1002/ajoc.201402214
CCCCn1c(nc2c1c1cccc3c1c1c2cccc1cc3)c1ccc2c(c1)C(CC)(CC)c1c2ccc(c1)c1ccc(s1)/C=C(/C(=O)O)\C#N	acetonitrile	-88	10.1002/ajoc.201402214
CCCCn1c(nc2c1c1cccc3c1c1c2cccc1cc3)c1ccc2c(c1)C(CC)(CC)c1c2ccc(c1)c1ccc(s1)c1ccc(s1)/C=C(/C(=O)O)\C#N	acetonitrile	-103	10.1002/ajoc.201402214
CCCCn1c(nc2c1c1cccc3c1c1c2cccc1cc3)c1ccc(cc1)c1ccc(s1)/C=C(/C(=O)O)\C#N	methanol	-99	10.1002/ajoc.201402214
CCCCn1c(nc2c1c1cccc3c1c1c2cccc1cc3)c1ccc(s1)c1ccc(s1)/C=C(/C(=O)O)\C#N	methanol	-131	10.1002/ajoc.201402214
CCCCn1c(nc2c1c1cccc3c1c1c2cccc1cc3)c1ccc(s1)c1ccc(s1)c1ccc(s1)/C=C(/C(=O)O)\C#N	methanol	-144	10.1002/ajoc.201402214
CCCCn1c(nc2c1c1cccc3c1c1c2cccc1cc3)c1ccc2c(c1)C(CC)(CC)c1c2ccc(c1)c1ccc(s1)/C=C(/C(=O)O)\C#N	methanol	-100	10.1002/ajoc.201402214
CCCCn1c(nc2c1c1cccc3c1c1c2cccc1cc3)c1ccc(cc1)c1ccc(s1)c1ccc(s1)/C=C(/C(=O)O)\C#N	methanol	-86	10.1002/ajoc.201402214
CCCCn1c(nc2c1c1cccc3c1c1c2cccc1cc3)c1ccc2c(c1)C(CC)(CC)c1c2ccc(c1)c1ccc(s1)c1ccc(s1)/C=C(/C(=O)O)\C#N	methanol	-127	10.1002/ajoc.201402214
CCCCn1c(nc2c1c1cccc3c1c1c2cccc1cc3)c1ccc(cc1)c1ccc(s1)/C=C(/C(=O)O)\C#N	toluene	-84	10.1002/ajoc.201402214
CCCCn1c(nc2c1c1cccc3c1c1c2cccc1cc3)c1ccc2c(c1)C(CC)(CC)c1c2ccc(c1)c1ccc(s1)/C=C(/C(=O)O)\C#N	toluene	-73	10.1002/ajoc.201402214
CCCCn1c(nc2c1c1cccc3c1c1c2cccc1cc3)c1ccc(cc1)c1ccc(s1)c1ccc(s1)/C=C(/C(=O)O)\	toluene	-71	10.1002/ajoc.201402214

SMILES	SOLVENT	SHIFT (nm)	DOI
C#N			
CCCCn1c(nc2c1c1cccc3c1c1c2cccc1cc3)c1ccc2c(c1)C(CC)(CC)c1c2ccc(c1)c1ccc(s1)c1ccc(s1)/C=C(/C(=O)O)\C#N	toluene	-94	10.1002/ajoc.201402214

Supplementary Information II

Molecular Screening of Absorption Spectrum Shifts in Dyes Adsorbed on Titania

Vishwesh Venkatraman[*a], Amsalu Efrem Yemene*[a], and John de Mello*[a]

[a] *Department of Chemistry, Norwegian University of Science and Technology (NTNU), 7491 Trondheim, Norway*

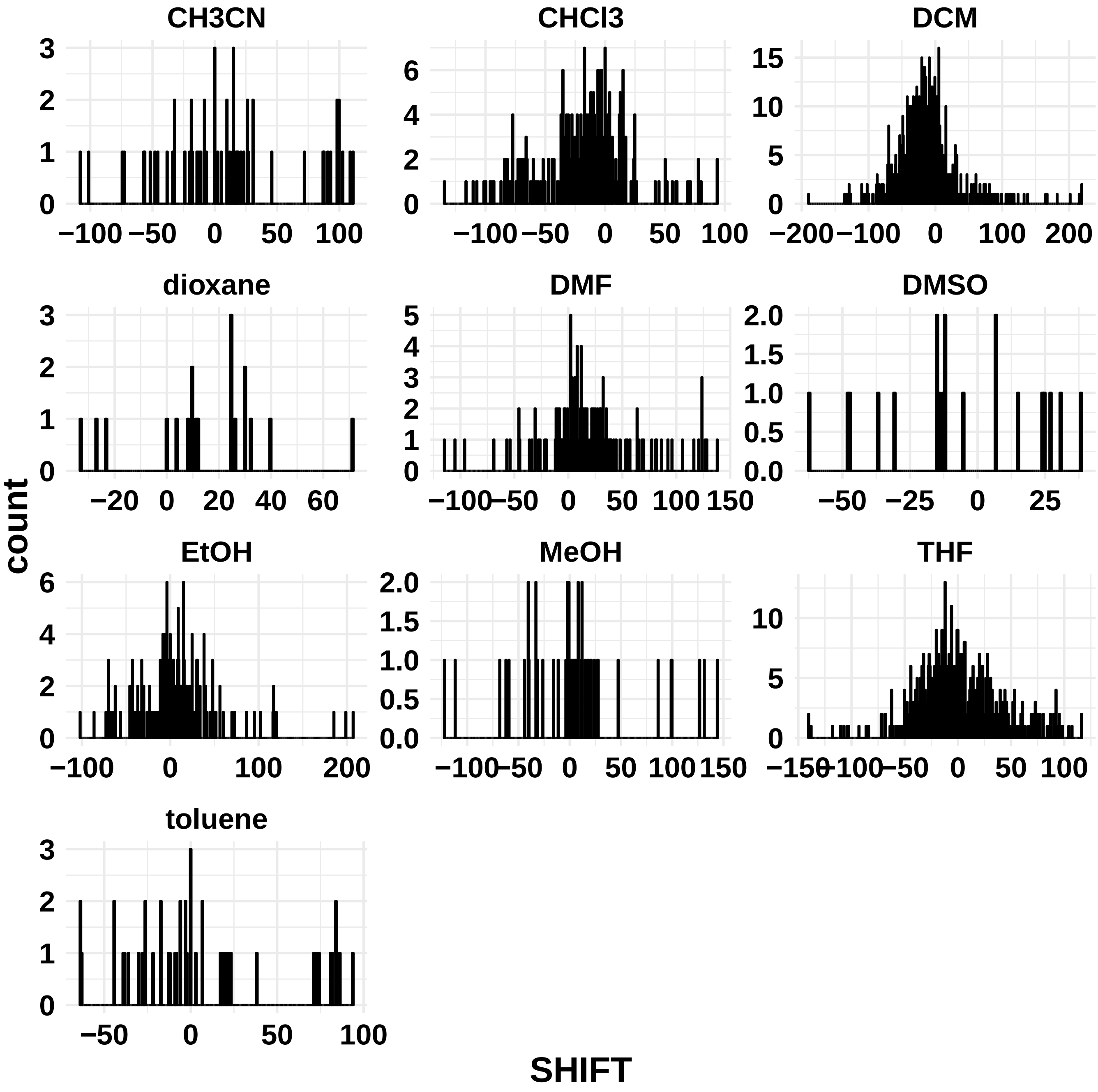

Figure F1: Histogram of the absorption peak shifts for different solvents.

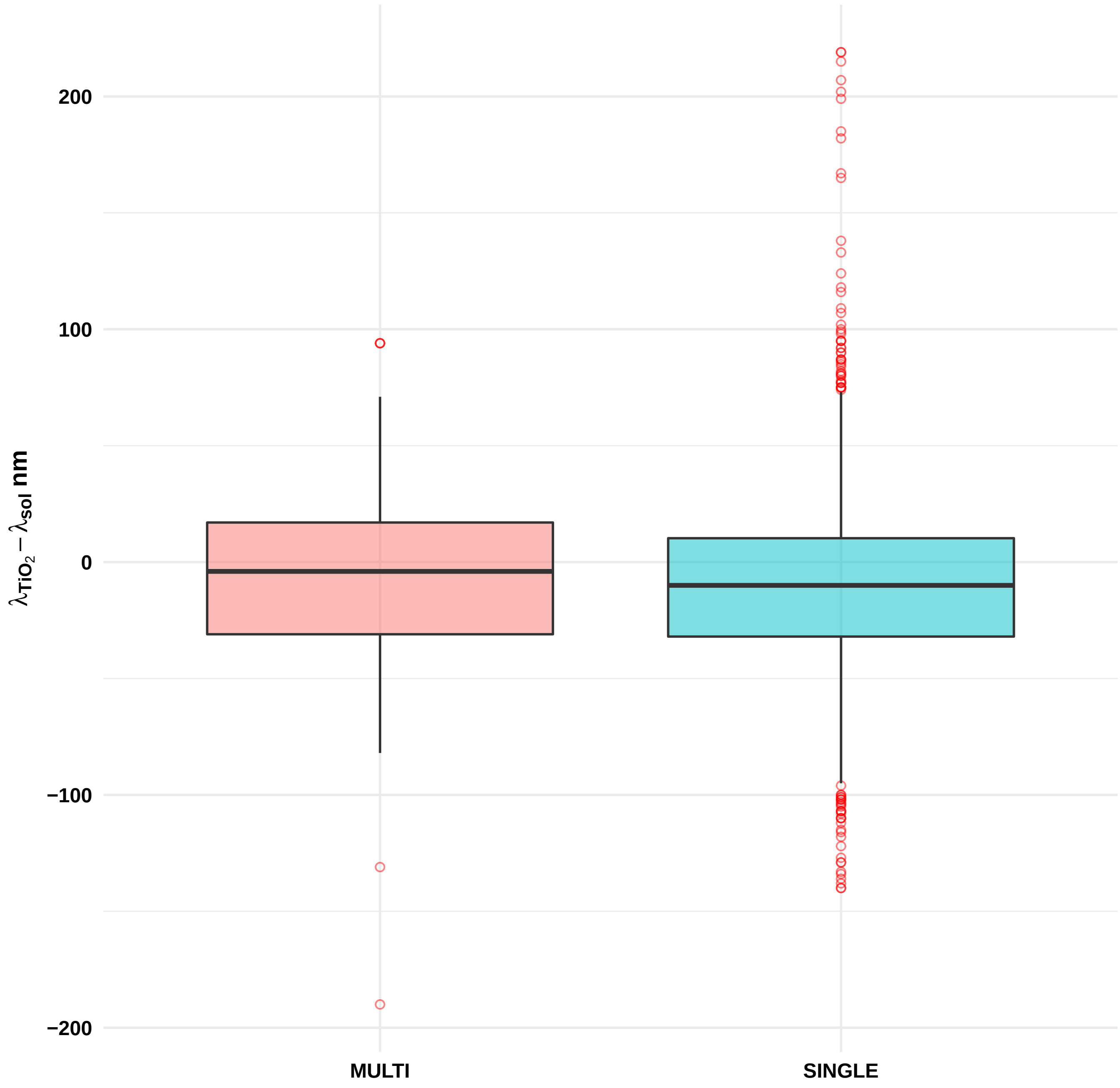

Figure F2: Bar plot comparing the range of shifts for dyes containing single and multiple anchoring groups.

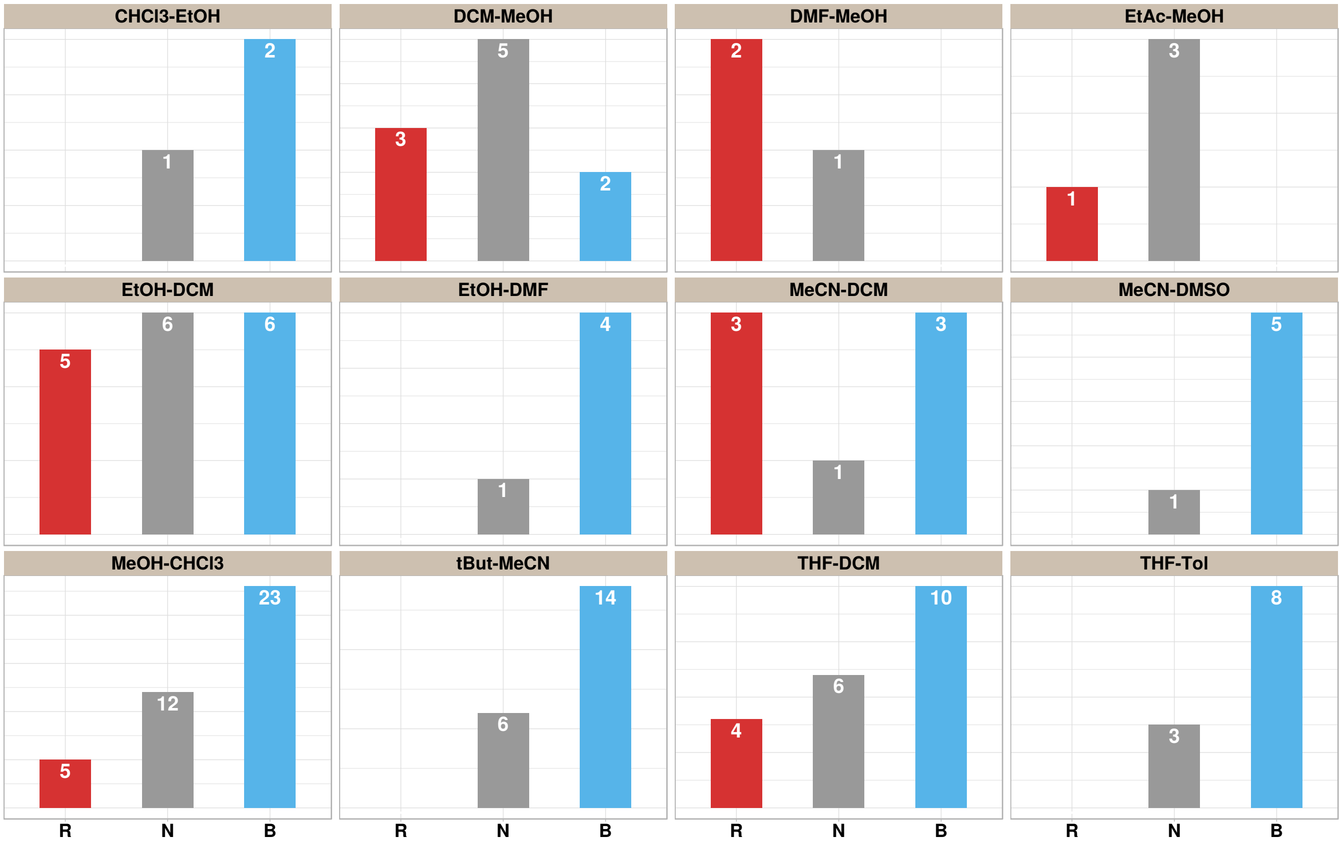

Figure F3: Plot comparing the types of shifts for dyes in mixed solvents. For ease of analysis, an equimolar (1:1) concentration ratio has been considered.

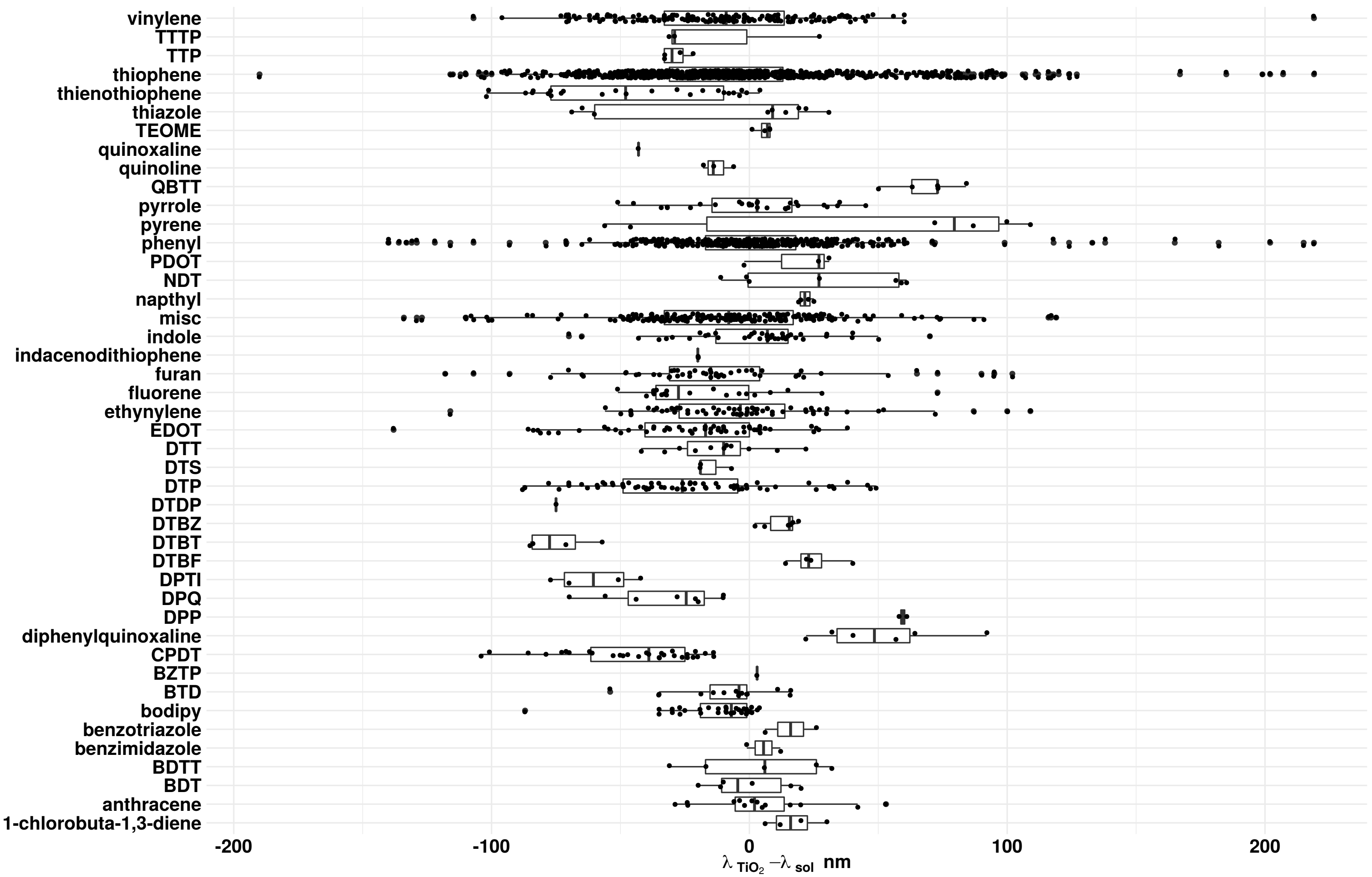

Figure F4: Boxplot showing the distribution of the absorption shifts with respect to the π-bridges/linkers present in the dye. The "misc" category refers to cases containing additional donors as a bridging group or in others is merged with the donor system.

π-bridges/linkers

The various π-bridges/linkers used in the dyes include:
dipentyldithieno[3,2-f:2,3-h]quinoxaline – DPQ
3,4-ethylenedioxythiophene – EDOT
3,4-Propylenedioxythiophene – PDOT
dithieno[3,2-b:2,3-d]pyrrole – DTP
8H-thieno[2,3:4,5]thieno[3,2-b]thieno[2,3-d]pyrrole – TTP
4,5-dihexyl-4,5-dihydrothieno[2,3:4,5]pyrrolo[2,3:4,5]thieno[3,2-b]indole – DPTI
9H-thieno[2,3':4,5]thieno[3,2-b]thieno[2,3':4,5]thieno[2,3-d]pyrrole – TTTP
benzo[1,2-b:4,5-b]dithiophene – BDT
dithieno[3,2-b;2,3-d]thiophene – DTT
dithieno[2,3-d:2,3-d]thieno[3,2-b:3,2-b]dipyrrole – DTDP
4-bis(2-ethylhexyl)-4H-silolo[3,2-b:4,5-b]dithiophene – DTS
diketopyrrolopyrrole – DPP
dithieno[3,2:3,4;2,3:5,6]benzo[1,2-d][1,2,3]triazole – DTBZ
dithieno[3,2:3,4;2,3:5,6]benzo[1,2-c]furazan – DTBF
triethylene oxide methyl ether – TEOME
naphthodithiophene – NDT
quinoxalinedithienothiophene – QBTT
Dithienopyrrolobenzotriazole – DTBT
benzodithienothiophene – BDTT
dithieno[3,2-b]pyrrolobenzotriazole – BZTP
dithieno[3,2-b]pyrrolobenzothiadiazole – BTTP

Experimental Details

Three dyes quercetin, 2,5-dihydroxytetraphthalic acid and carminic acid were obtained commercially from Sigma-Aldrich. UV-Vis experiments were performed with a Hitachi U-1900 UV-Vis spectrophotometer using quart cuvettes of 1cm path length and scanning from 190-700 nm. Extinction coefficients (ϵ) were calculated using Beer-Lambert's law. Absorption spectra of all dyes from THF and ethanol solution of 10^{-4} M concentration are presented in Figure F5and F6. Figure F7shows the absorption spectra for dye sensitized TiO_2 films on FTO glass substrate (prepared from 0.5 mM solution of dyes). The measurements were carried out using the same spectrophotometer with a non-stained TiO_2/FTO glass substrate as a background.

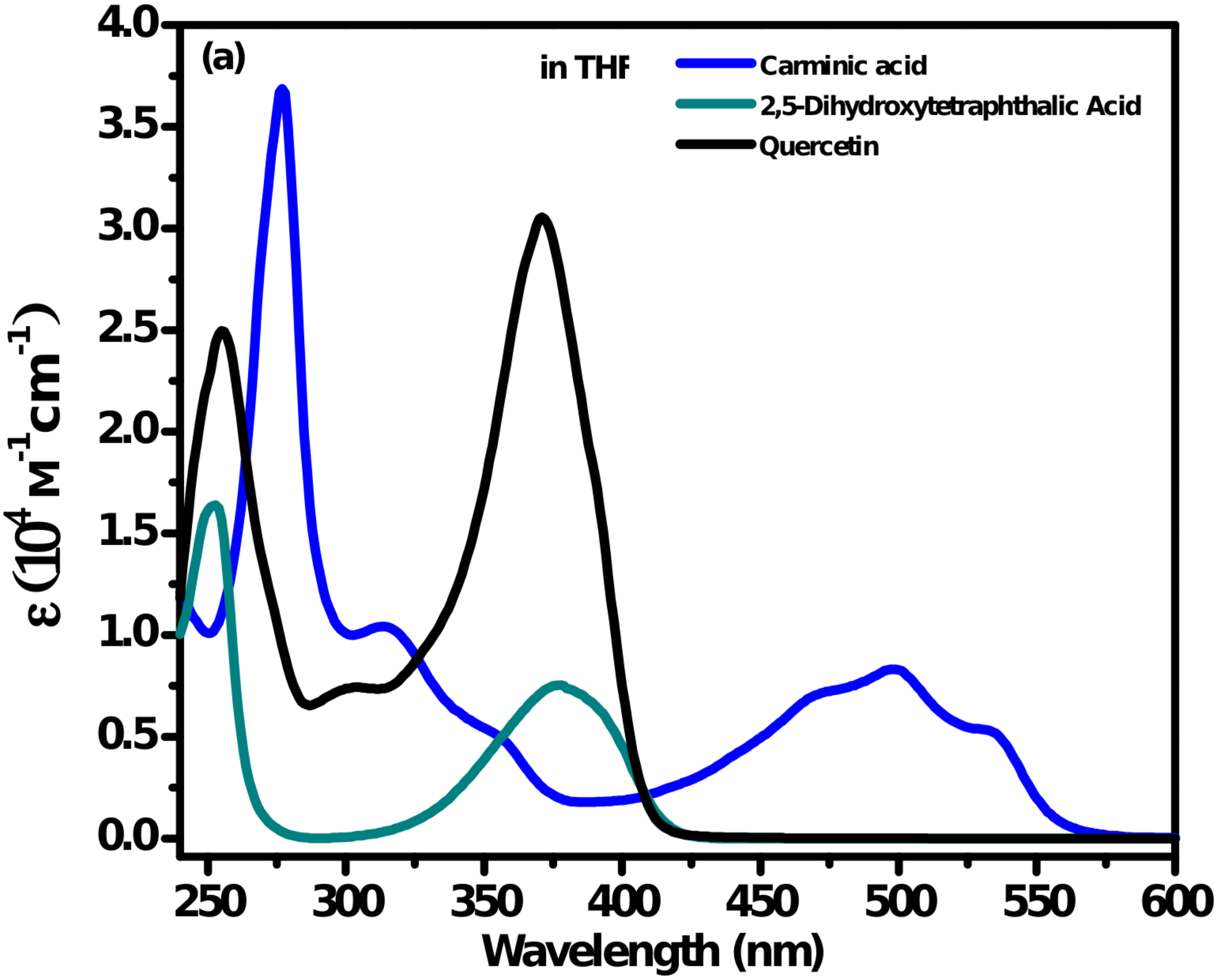

Figure F5: Absorption spectra of the dyes in THF.

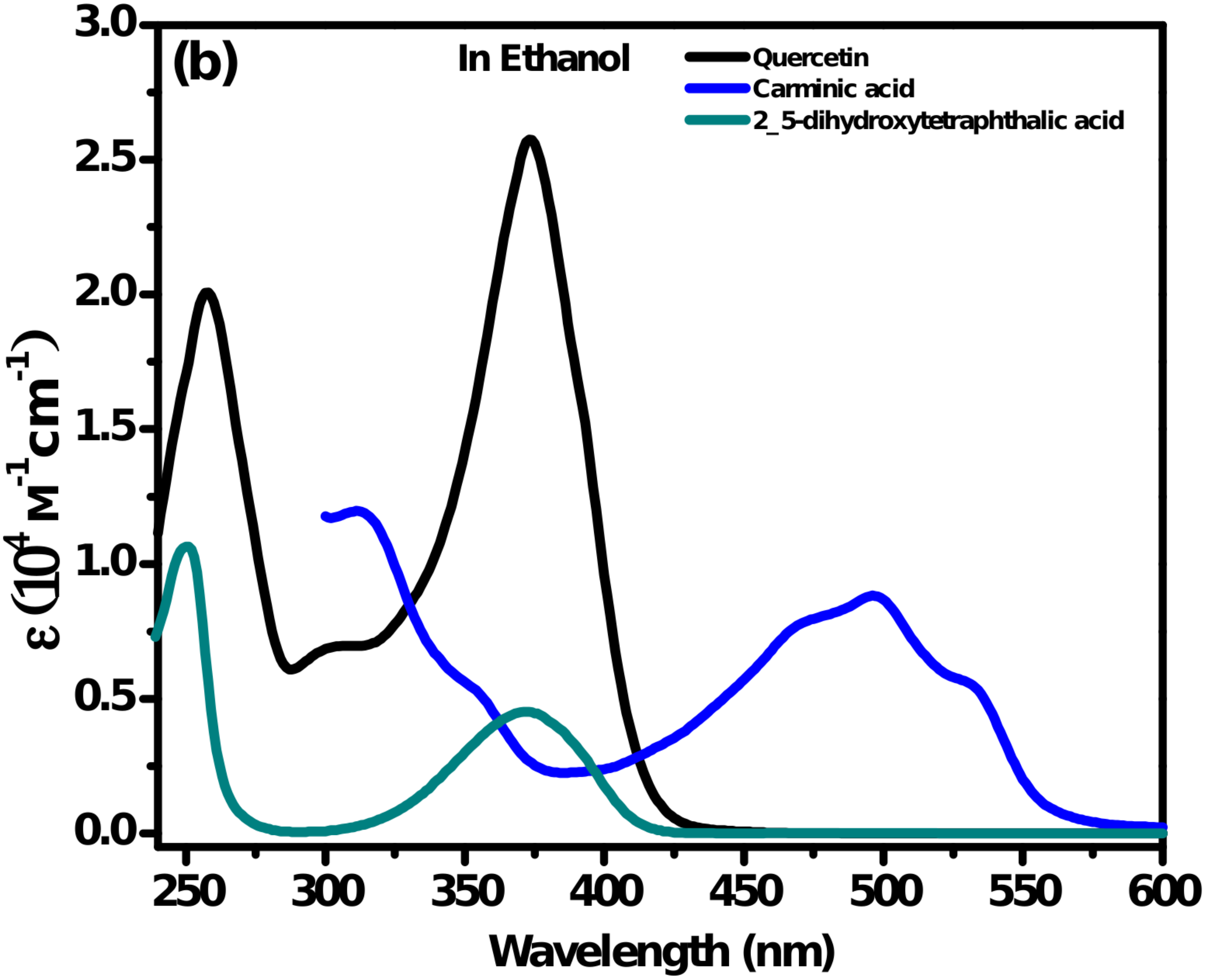

Figure F6: Absorption spectra of the dyes in ethanol.

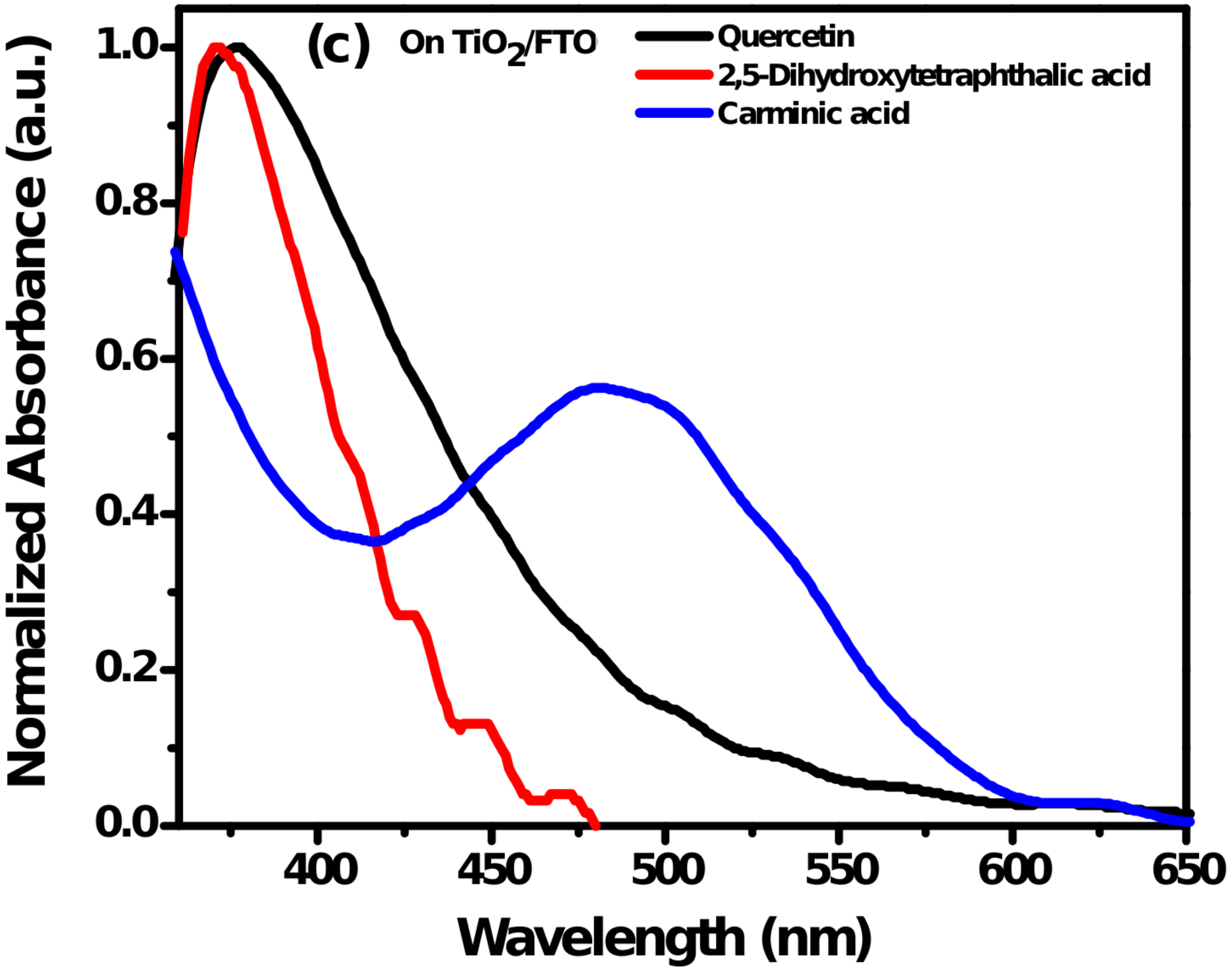

Figure F7: Absorption spectra of the dye-sensitized film.

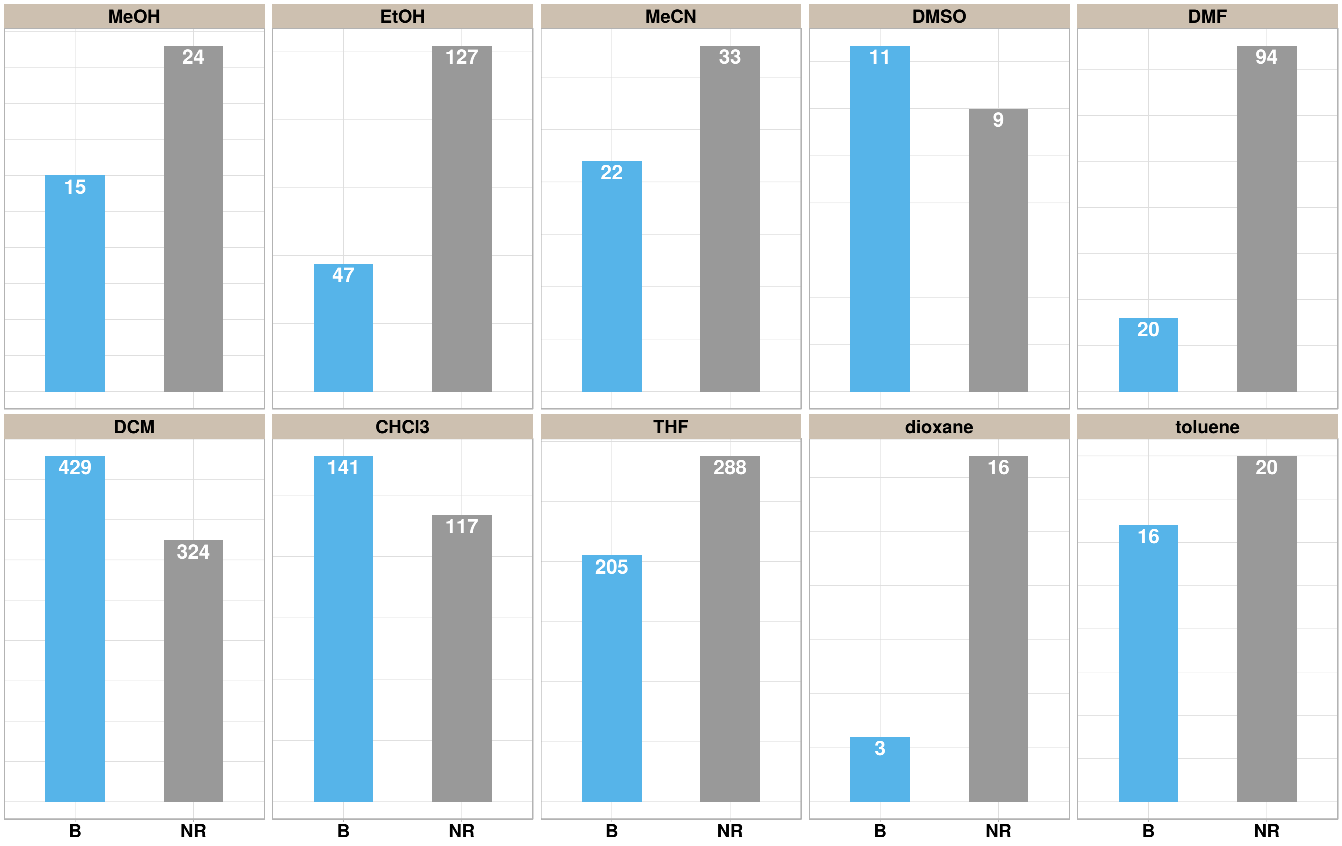

Figure F8: Distribution of the absorption shifts with respect to the solvents when cases are merged. The letter ”B” indicates a blue shift, while ”N” and ”R” correspond to no change or red shift respectively. Here, ”N” and ”R” are merged into one group. The solvents are sorted in increasing order of polarity from left to right.

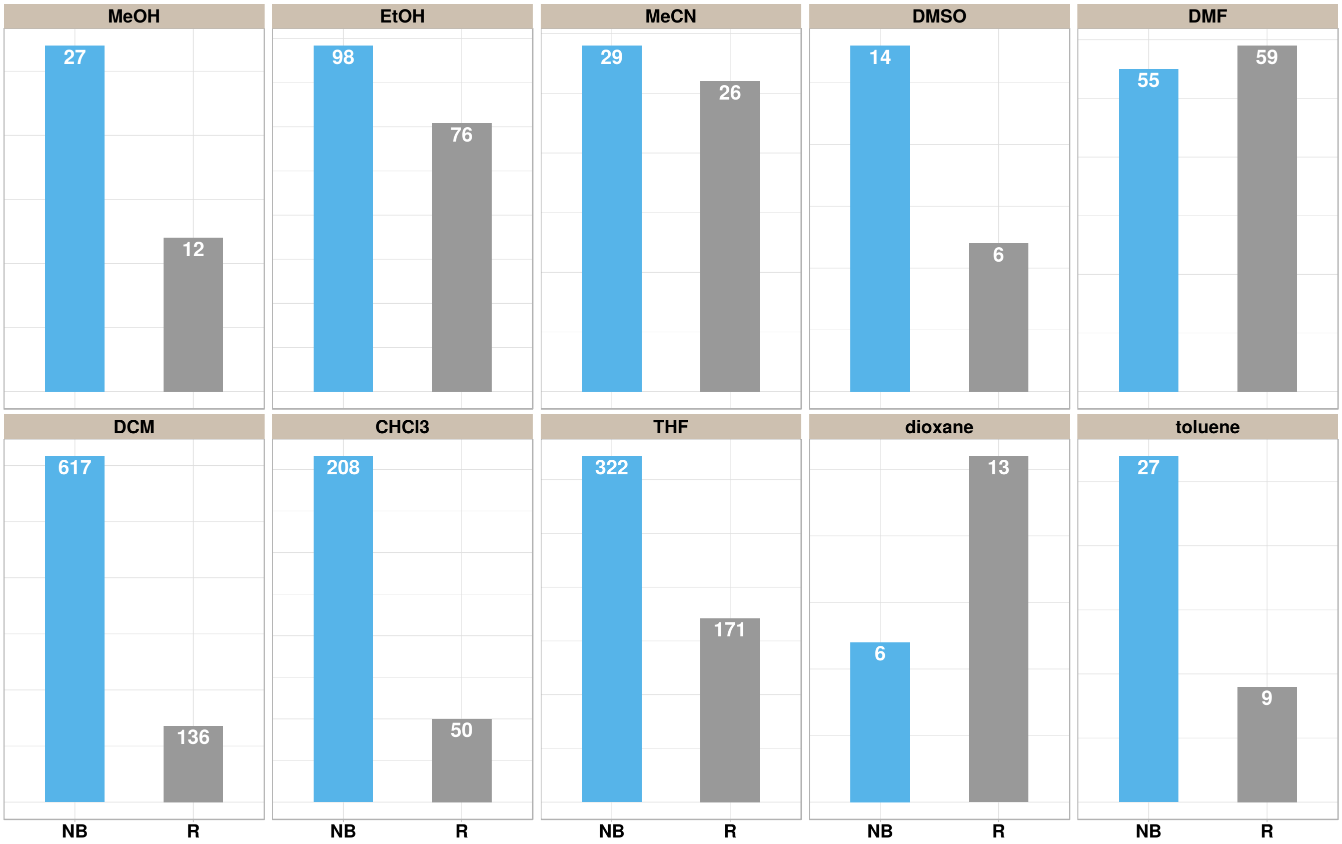

Figure F9: Distribution of the absorption shifts with respect to the solvents when cases are merged. The letter "B" indicates a blue shift, while "N" and "R" correspond to no change or red shift respectively. Here, "N" and "B" are merged into one group. The solvents are sorted in increasing order of polarity from left to right.
